# Comparison analysis of full-spectrum metabolomics revealed on the variation of potential metabolites of unscented, *Chloranthus spicatus* scented, and *Osmanthus fragrans (Thunb.)* Lour. scented *Congou* black teas

**DOI:** 10.3389/fnut.2023.1234807

**Published:** 2023-08-14

**Authors:** Ping Tang, Jie-Qiong Wang, Yong-Feng Wang, Jian-Chang Jin, Xin Meng, Yan Zhu, Ying Gao, Yong-Quan Xu

**Affiliations:** ^1^Hangzhou Vocational & Technical College, Hangzhou, China; ^2^Key Laboratory of Biology, Genetics and Breeding of Special Economic Animals and Plants, Ministry of Agriculture and Rural Affairs, Tea Research Institute Chinese Academy of Agricultural Sciences, Hangzhou, China; ^3^College of Food Science, Southwest University, Chongqing, China; ^4^Jingdezhen Jin Gui Yuan Agricultural Development Co Ltd, Jingdezhen, China; ^5^College of Biological and Environmental Engneering, Zhejiang Shuren University, Hangzhou, China; ^6^College of Food and Health, Zhejiang A&F University, Hangzhou, China

**Keywords:** scented black teas, *Osmanthus fragrans (Thunb.)* Lour., *Chloranthus spicatus*, non-volatile metabolites, volatile metabolites

## Abstract

**Introduction:**

In recent years, scented black tea has attracted much attention due to its pleasant floral aroma and mellow flavor, but little research has been carried out on its flavor metabolic profile.

**Methods:**

In this study, the flavor metabolic profiles of unscented, *Chloranthus spicatus* scented, and *Osmanthus fragrans (Thunb.)* Lour. scented *Congou* black teas were investigated using full-spectrum metabolomics analysis method, the first time that the flavor profiles of scented black tea were characterized in detail.

**Results and Discussion:**

The results revealed that a total of 3,128 metabolites were detected in the three teas. Based on the criteria of variable importance in the project >1 and fold change ≥2 or ≤  0.5, 761 non-volatile metabolites and 509 volatile metabolites were filtered as differential metabolites. Many differential non-volatile metabolites belonged to flavonoids, phenolic acids, and terpenoids. Floral, fruity and herbaceous volatile metabolites were significantly up-regulated in *Chloranthus spicatus* scented *Congou* black tea while sweet and fruity volatile metabolites were significantly down-regulated in *Osmanthus fragrans (Thunb.)* Lour. scented *Congou* black tea. The results contribute to a better understanding of the scenting techniques on the flavor quality of scented black teas and provide some information on the flavor chemistry theory of scented black teas.

## 1. Introduction

Black tea, a fully fermented tea, is loved by consumers around the world for its health benefits (1–4) and mellow taste. Black tea is widely produced in countries such as China, India, Sri Lanka, Kenya and Indonesia (5, 6). It is popular and ranks first in the world in terms of production and consumption. The world black tea production accounted for around 65% of total tea production in 2017 (7). The *Congou* black tea (CK), a unique Chinese black tea variety, are with tightly striped shape, sweet and mellow taste, and floral and fruity aroma (8). The aroma of CK can be classified as sweet, floral or fruity, based on sensory characteristics (9). The general processes of producing CK are divided into two phases. The enzymatic phase includes the withering, rolling and fermentation processes, while the non-enzymatic phase include the drying process (10). The flavour quality is mainly formed in the enzymatic phase by the conversion of non-volatile metabolites, such as flavonoids, amino acids, fatty acids, and volatile metabolites derived from fatty acids, amino acids, terpenoids, carotenoids, and glycosidically bound volatiles (11).

In recent years, scented teas have been gaining attention for their pleasant floral aroma and mellow flavour. As a material with both ornamental and health benefits, such as protecting stomach and liver, dispersing phlegm, and antioxidant activity, *Osmanthus fragrans (Thunb.)* Lour. is ideal to be the raw material for the production of scented tea (12). Linalool, ionone and ocimene are aroma-contributing substances in it (13). Chloranthus spicatus is well-known for its elegance and is one of the “*The Four Famous Chinese Camellias*,” along with jasmine, *Michelia alba DC.* and *Citrus aurantium*. As one of the traditional Chinese scented teas, Chloranthus spicatus was also ideal for the production of scented tea.

The changes in various volatile metabolites during the scenting process are crucial to the formation of scented tea aroma. The scenting process decreased alcohols but increased ketones and esters in *Osmanthus* scented black tea (14). Nonyl aldehyde, γ-decalactone, 1-octene-3-ol, trans-2-hexenyl pentanoate, and phenethyl butyrate were key odorants contributing to its *Osmanthus* aroma. Currently, the changes of volatile metabolites during the scenting process of producing *Chloranthus spicatus* scented black tea is less studied. The changes of non-volatiles metabolites during the scenting process should have an effect on the formation of scented tea quality, but still largely remain unveiled.

Based on these facts, the present study characterized the flavor metabolites of CK, *Osmanthus* scented *Congou* black tea (OF), and *Chloranthus spicatus* scented *Congou* black tea (CH) by ultra-performance liquid chromatography tandem mass spectrometry (UPLC-MS/MS) and gas chromatography–mass spectrometry (GC–MS). The results aim to understand the effect of the scenting process on the metabolic profile of black tea and help broaden the ideas for the study of flavor quality of scented black tea.

## 2. Materials and methods

### 2.1. Chemicals

Methanol, acetonitrile and hexane (all chromatography grade) for the tests were obtained from Merck (Darmstadt, Germany). Formic acid was purchased from Aladdin (Shanghai, China). Sodium chloride was produced from Sinopharm (Shanghai, China). Authentic standards used in the full-spectrum metabolomics analysis were obtained from MetWare (Wuhan, China).

### 2.2. Preparation of tea samples

The CK used in the experiment was purchased from Jingdezhen Jin Gui Yuan Agricultural Development Co (Jiangxi, China). The black tea was made according to the traditional *Congou* black teas process, which consisted mainly of indoor withering, rolling, fermentation and drying. The CH and OF were scented with CK as the raw material, and the specific scenting process was as follows (15):

1. Flower spreading: Pick *Chloranthus* spicatus and *Osmanthus* buds, ensure their integrity during the picking process, remove impurities such as flower tips, stalks and leaves and then stall them indoors. Indoor environment: ventilation, humidity of 65–75%, temperature of 20–24°C, thickness of 15 cm, spreading time of 30 min.
2. Mixing and resting of tea flowers: 1/3 of the CK was used to produce CH and the other 1/3 was used to produce OF. For CH, the flowers are mixed with CK in a ratio of 3:100 (flowers: dried tea, w:w) for 100 d. For OF, the flowers are mixed with CK in a ratio of 3:10 (flowers: dried tea, w: w) for 2 days, with 1 renewal, the process lasts about 4 days in total.
3. Drying: The scented teas were baked at 85°C (6CTH-30, Zhejiang Chunjiang Tea Machinery Co., Ltd.) for 412 min, cooled for 40 min, baked at 75°C for 90 min and cooled for 120 min before removing the petals and debris, resulting in scented *Congou* black teas. Three replicates were set for each treatment. The samples from three treatments were stored in a refrigerator at −80°C until assayed. The three samples were mixed and sampled three times for subsequent testing to ensure homogeneity and traceability.

### 2.3. UPLC-MS/MS based analysis of non-volatile metabolites

#### 2.3.1. Sample pretreatment

The three samples were vacuum freeze-dried in a lyophilizer (Scientz-100F) before being ground (30 Hz, 1.5 min) into powder by a grinder (MM 400, Retsch). Afterwards, 50 mg of sample powder was accurately weighed and 1.2 mL of 70% methanolic aqueous internal standard extraction solution pre-chilled at −20°C was added. The sample was vortexed once every 30 min for 30 s, for a total of 6 times. Then centrifuged (12,000 rpm, 3 min), the supernatant was aspirated and filtered through 0.22 μm microporous membrane for determination.

#### 2.3.2. UPLC-MS/MS conditions

An UPLC system equipped with a tandem mass spectrometer was used to analyze the non-volatile metabolites in the samples. (1) Liquid phase conditions: An Agilent SB-C18 1.8 μm, 2.1 mm * 100 mm column was equipped with a flow rate of 0.35 mL/min, a column temperature of 40°C, and an injection volume of 2 μL. The mobile phase A was 0.1% formic acid/ultrapure water and mobile phase B was 0.1% formic acid/acetonitrile. The gradient elution method was as follows: mobile phase B was 5% at 0 min; increased linearly to 95% at 9 min, maintained for 1 min; decreased to 5% at 10–11.10 min and equilibrated at that ratio until 14 min. (2) Mass spectrometry conditions: electrospray ionization temperature (ESI) of 500°C, ion spray (IS) voltage of 5,500 V (positive ion mode) and − 4,500 V (negative ion mode); ion source gases I (GSI), gases II (GS II), and curtain gas (CUR) of 50, 60 and 25 psi, respectively; collision-induced ionization parameters of high. The multiple reaction monitoring (MRM) mode was used for triple quadrupole (QQQ) scanning, and the collision gas (nitrogen) was medium. The declustering potential (DP) and collision energy (CE) of individual MRM ion pairs were evaluated by further DP and CE optimization. A specific set of MRM ion pairs was monitored in each period depending on the metabolites eluted during the period (16).

#### 2.3.3. Identification of non-volatile metabolites

Based on the MWDB (metware database), the substance characterization was performed based on the secondary spectrum information of the substance, and the analysis removed the isotopic signals, the duplicate signals containing K^+^, Na^+^, NH_4_^+^, and the duplicate signals of fragment ions that were themselves other larger molecular weight substances. After obtaining the metabolite spectral analysis data of different samples, the peaks of all substances were integrated by peak area using MultiQuant software, and the mass spectra of the same metabolite in different samples among them were corrected for the integration of the peaks.

### 2.4. GC–MS based analysis of volatile metabolites

#### 2.4.1. Sample pretreatment

The sample was removed from the refrigerator at −80°C and firstly ground by liquid nitrogen, followed by vortexing to make a homogeneous mixture. The 500 mg of the sample was accurately weighed in a headspace vial, subsequently 2 mL of saturated sodium chloride solution were added, the vial was sealed, and then the sample was extracted by fully automated headspace solid phase microextraction (HS-SPME) for GC–MS analysis.

#### 2.4.2. GC–MS conditions

The headspace vial containing the tea samples was shaken and equilibrated at 60°C for 5 min, followed by insertion of a 120 μm divinylbenzene/carboxen/polydimethylsiloxane (DVB/CWR/PDMS) extraction head into the vial for 15 min (stirring while extracting was used in the extraction process with a stirring rate of 1,000 rpm, thus stability and homogeneity of the extraction process and reducing human error), followed by desorption at the inlet end at 250°C for 5 min for subsequent GC–MS separation and identification. It should be noted that the extraction head should be heated at the inlet end at 250°C for 5 min before sampling. (1) Chromatographic conditions: DB-5MS capillary column (30 m × 0.25 mm × 0.25 μm, Agilent J&W Scientific, Folsom, CA, United States) supplemented with high purity helium (purity not less than 99.999%) as carrier gas. The program temperature was set as follows: 40°C for 3.5 min, then 10°C/min to 100°C, then 7°C/min to 180°C, and finally 25°C/min to 280°C for 5 min. (2) Mass spectrometry conditions: electron bombardment ion source (EI); ion source, quadrupole and mass spectrometry interface temperatures of 230°C, 150°C and 280°C, respectively; electron energy of 70 eV, scanning mode of selected ion detection mode (SIM), qualitative and quantitative ion precision scanning (GB 23200.8–2016).

#### 2.4.3. Identification of volatile metabolites

Based on multi-species, literatures, authentic standards, and retention index (RI), the database was established independently, containing the identified retention time (RT) as well as qualitative and quantitative ions for the selected ion detection mode for precise scanning, with one quantitative ion and two to three qualitative ions selected for each compound, respectively. All ions to be detected in each group were detected separately in the order of of their peaks, in separate time periods. If the retention time of the detected peaks was consistent with the authentic standard and the selected ions all appeared in the mass spectra of the samples after background deduction, the substance would be ascertained. The odor descriptions for the qualitative aroma substances were taken from websites such as http://www.thegoodscentscompany.com, http://perflavory.com/, http://www.odour.org.uk, http://foodflavorlab.cn. The quantitative ions were selected by MassHunter software for the integration and calibration of the peaks in order to enhance the accuracy of quantification.

### 2.5. Statistical analysis

All samples were set up with three biological replicates and statistical analysis was performed using IBM SPSS statistics (version 25.0, SPSS Inc., Chicago, IL). The principal component analysis (PCA), partial least squares-discriminant analysis (PLS-DA), clustering heat map, K-Means plot, and key metabolite kyoto encyclopedia of genes and genomes (KEGG) enrichment pathway maps were done by R software.^1^ The identified metabolites were first annotated through the KEGG compound database,^2^ and subsequently the annotated completed metabolites were mapped to the KEGG pathway database.^3^ The pathways mapped to metabolites with significant regulation were subsequently sent to metabolite set enrichment analysis (MSEA), whose significance was determined by *p*-values of hypergeometric tests.

## 3. Results and discussion

### 3.1. Overall profile of metabolites in the three black teas

Metabolomics allows the detection and screening of significant differential metabolites from different biological samples, which can be used as a basis to further elucidate the metabolic processes and mechanisms of change in biological samples. In this study, a full-spectrum metabolomics analysis based on dual platforms of UPLC-MS/MS and GC–MS was carried out. A total of 3,128 metabolites, including 2,396 non-volatile metabolites and 732 volatile metabolites, were detected in three black tea samples.

**Figure 1:**
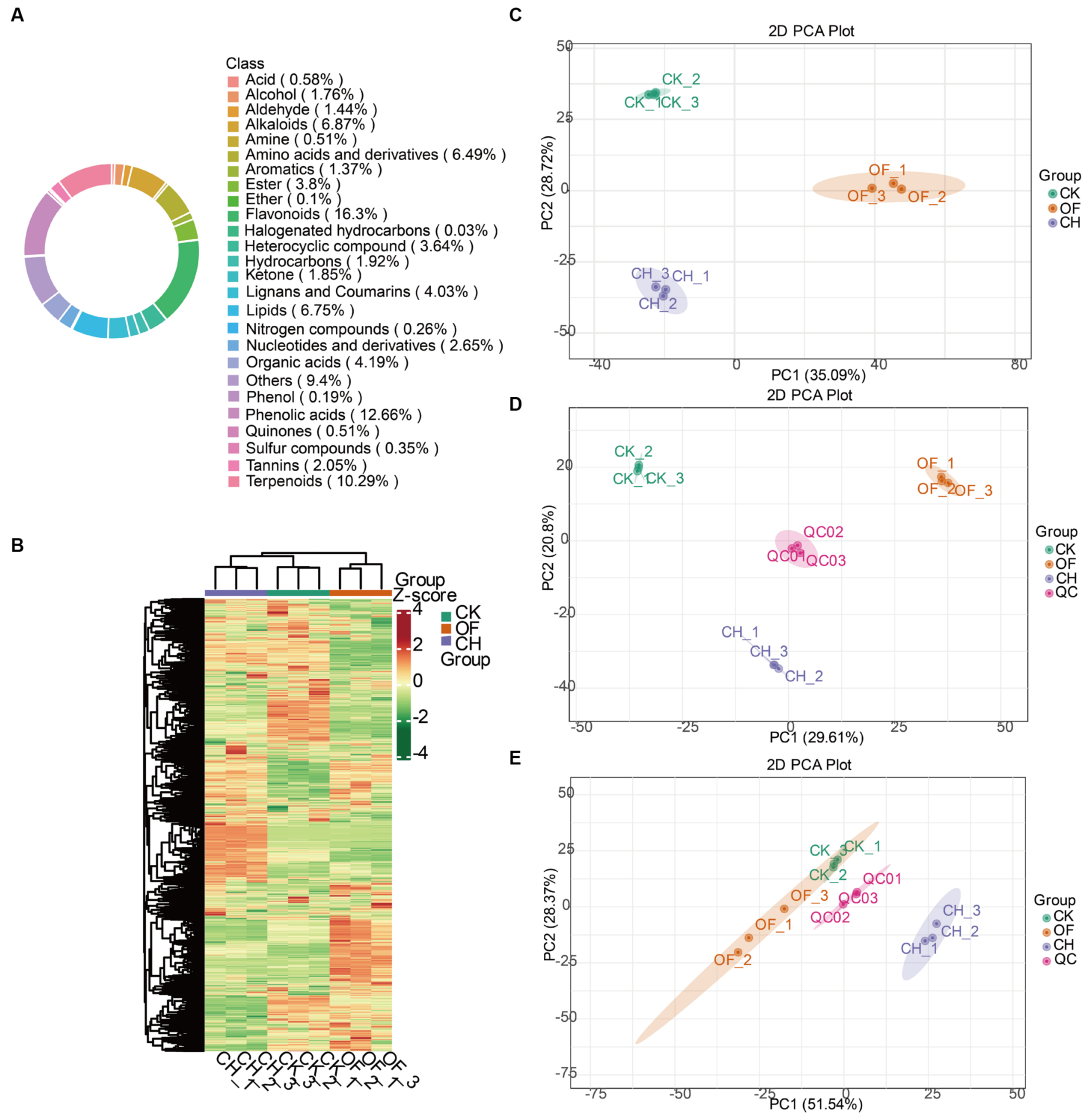
Analysis of all metabolites in three types of black tea (CK, CH and OF). **(A)** Ring diagram of total metabolite class composition; **(B)** Heat map of total metabolite content clustering (processed by Z-score); **(C)** PCA analysis of total metabolites; **(D)** PCA analysis of non-volatile metabolites; **(E)** PCA analysis of volatile metabolites. CK, Congou black tea; OF, Osmanthus black tea; CH, Chloranthus spicatus black tea. Same below.

As shown in Figures 1A, a total of 26 categories of metabolites were detected in black teas. Flavonoids, phenolic acids, terpenoids, others, alkaloids, lipids, amino acids and derivatives together occupied 68.76% of all metabolites. Lignans and coumarins (4.03%), organic acids (4.19%), esters (3.8%), heterocyclic compounds (3.64%), nucleotides and derivatives (2.65%), tannins (2.05%), hydrocarbons (1.92%), ketones (1.85%), alcohols (1.76%), aldehydes (1.44%), aromatics (1.37%) were minor metabolites. Acids, quinones, amines, sulfur compounds, nitrogen compounds, phenols, ethers, and halogenated hydrocarbons accounted for less than 1%. A previous study also pointed out that flavonoids played an important role in the quality and flavour of black tea during black tea processing (17), which was similar to the above results.

The heatmap distribution in Figure 1B indicated that the metabolite contents in the three black tea samples were significantly different. To further validate the above results, PCA, as an unsupervised pattern recognition method for multidimensional data statistical analysis, was introduced to analyze the differences of overall metabolites (Figure 1C), non-volatile metabolites (Figure 1D), and volatile metabolites (Figure 1E) among the three black tea samples. The results revealed that the three samples achieved good separation in each PCA model, and the sum of the first and second principal components of the above three data sets explained more than 50% of the whole data set, reaching 63.81, 50.41 and 79.91%, respectively, indicating that the three black tea metabolites reached significant differences among them. The results provided some data support for further screening the important differential metabolites.

### 3.2. Important differential metabolites among the three black teas

1,270 differential metabolites were initially identified based on the screening criteria of variable importance in the project (VIP)>1 and fold change (FC) ≥2 or ≤ 0.5, among which 761 non-volatile metabolites (Supplementary Table S1) and 509 volatile metabolites (Supplementary Table S2) were identified.

**Figure 2:**
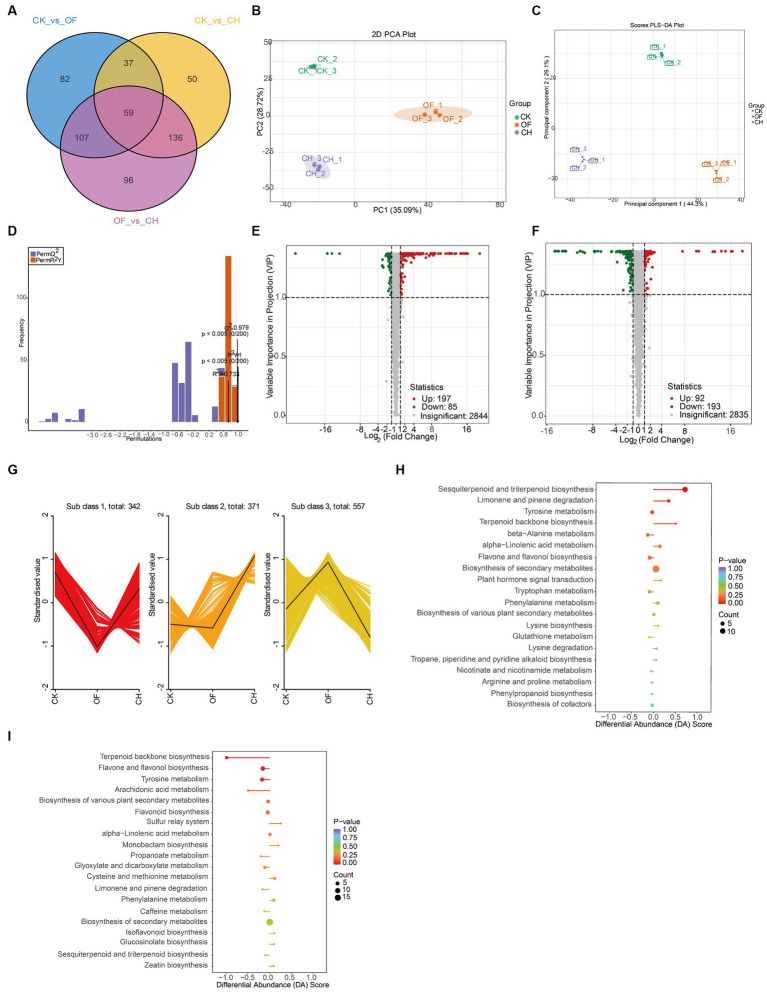
Analysis of important differential metabolites in three types of black tea (CK, CH and OF). **(A)** Venn plot; **(B)** PCA plot; **(C)** PLS-DA plot; **(D)** PLS-DA 200-permutation plot; **(E,F)** Volcano plot (E. CK vs. CH; F. CK vs. OF); **(G)** K-means plot; **(H,I)** KEGG differential abundance score plot (**H**. CK vs. CH; **I**. CK vs. OF; where the color and size of the dots indicated the size of the *p*-values and number of metabolites, respectively; DA score indicated the overall change of all metabolites in the metabolic pathway, 1: up-regulation, −1: down-regulation, and the length of the line segment indicated the DA score).

The Venn plot indicated the relationships of differential metabolites between different black tea samples. The results showed that there were 59 differential metabolites between the three comparison groups (CK vs. OF, CK vs. CH, OF vs. CH) and 82, 50, 96 differential metabolites were unique to each group (Figure 2A). To further verify whether the differential metabolites among the three black tea samples reached significant differences, an unsupervised model statistical method, i.e., PCA (Figure 2B), and a supervised model statistical method, i.e., PLS-DA (Figure 2C), were used simultaneously to screen for differential metabolites. It was found that both models could achieve good separation. In 200 permutations of validation of the PLS-DA model (Figure 2D), R2X = 0.733, R2Y = 1 (*p* < 0.005), Q2 = 0.979 (*p* < 0.005), indicating a good model fit.

The Volcano plot was mainly used to show the relative abundances of metabolites between two samples. Each dot in the plot indicated a differential metabolite. Green dots indicated down-regulated substances, red dots indicated up-regulated substances, and gray indicated substances with insignificant differences. The results showed that compared to CK, 197 substances were up-regulated and 85 substances were down-regulated in CH (Figure 2E), while 92 substances were up-regulated and 193 substances were down-regulated in OF (Figure 2F), which might be related to the different flowers used in the scenting process.

The K-means plot focused on classifying the trends of relative abundances of important differential metabolites under different comparison groups to facilitate subsequent targeted analysis. According to Figure 2G, three different trends in the levels of these important differential metabolites were detected in CK, CH and OF. Among them, 342 metabolites were lower in OF than in CK and 371 metabolites were higher in CH than in CK. It was obvious that more substances were up-regulated in CH and down-regulated in OF after CK was scented, suggesting that the type of flower had a great influence on the metabolites of CK.

Subsequently, the top 20 KEGG metabolic pathways in the three samples were screened based on differential abundance (DA) scores and *p*-values sizes (Figures 2H,I). The sesquiterpenoid and triterpenoid biosynthesis, limonene and pinene degradation, and tyrosine metabolism reached significant levels in the metabolite expression of CH (compared to CK), with the first two pathways being significantly up-regulated and the last one remaining basically stable. Sesquiterpenoids and triterpenoids were generally biosynthesized *via* the cytoplasmic mevalonate (MVA) pathway in plants (18–20). Triterpenoids usually had a variety of stereoisomers and positional isomers, but the main compounds were the madecassic acid isomer and asiatic acid isomer (21). In OF, the expression of metabolic pathways such as terpenoid backbone biosynthesis, flavone and flavonol biosynthesis, and tyrosine metabolism were significantly down-regulated overall. Terpenoids were derived from the same C5 isoprene backbone produced from isopentenyl pyrophosphates (IPP) and dimethyl allyl pyrophosphates (DMAPP) and were used in the synthesis of terpene backbones by rearrangement and cyclisation reactions (22), where linalool, nerol and alpha-terpineol could be produced by further reactions in the biosynthesis of terpene backbones (23). The results revealed differences in flavor quality between different scented black teas (CH and OF) from a metabolomics perspective. However, the most critical contributors to this difference were still unknown, both in terms of non-volatile and volatile substances.

### 3.3. Analysis of key differential non-volatile metabolites among the three black teas

#### 3.3.1. Analysis of key differential categories

Based on the percentage of metabolite categories, all non-volatile metabolites were further screened and a total of 173 metabolites were selected (Table 1). In terms of categories, the metabolites in the three black teas mainly consisted of amino acids and derivatives, phenolic acids, flavonoids, lipids, accounting for 7.69, 20.12, 52.66 and 19.53%, respectively. In terms of peak area, flavonoids accounted for a relatively large proportion of all key metabolites, reaching 96.30% in CK, and compared with CK, the percentage of peak area of flavonoids increased by 0.39 and 6.72% in CH and OF, respectively. The variation of flavonoids was determined by the thermal stability during the drying stage (17). Compared with CK, the peak area of lipids in CH was reduced by 0.43%. The lipids were important precursors of volatile metabolites in tea leaves (24). It was reported that lipids were degraded by enzymatic hydrolysis or oxidation of glycolipids and phospholipids to produce flavor volatiles during black tea processing (25), which was in accordance with our experimental results. The total peak area of amino acids and derivatives in OF was reduced by 5.18% compared to CK (Supplementary Table S1), which indicated that the Maillard reaction, deamidation polymerization and caramelization might occur during the scenting process (26).

**Table 1:** Key differential non-volatile metabolites changes among the three black teas.

No.	Molecular weight (Da)	Q1 (Da)^a^	Q3 (Da)^b^	Formula	Ionization model^c^	Compounds^d^	Level^e^	CH_FC^f^	CH_Log_2_FC^g^	CH_Type^h^	OF_FC ^f^	OF_Log_2_FC^g^	OF_Type^h^
*Amino acids and derivatives (13)*													
1	145.0739	144.07	126.06	C_6_H_11_NO_3_	[M-H]^−^	Allysine	3	2.19	1.13	Up			
2	149.0510	150.06	61.01	C_5_H_11_NO_2_S	[M + H]^+^	DL-Methionine	3				4.18	2.06	Up
3	163.0667	164.07	56.05	C_6_H_13_NO_2_S	[M + H]^+^	L-Homomethionine	3				2.63	1.39	Up
4	188.1273	189.13	144.10	C_7_H_16_N_4_O_2_	[M + H]^+^	Homoarginine	3				3.16	1.66	Up
5	191.0616	192.07	56.05	C_7_H_13_NO_3_S	[M + H]^+^	N-Acetyl-L-Methionine	3				5.16	2.37	Up
6	215.0946	214.09	153.07	C_13_H_13_NO_2_	[M-H]-	3-(2-Naphthyl)-L-alanine	3				2.98	1.57	Up
7	222.1004	223.11	120.09	C_11_H_14_N_2_O_3_	[M + H]^+^	L-Glycyl-L-phenylalanine*	2				2.04	1.03	Up
8	246.1004	245.09	203.08	C_13_H_14_N_2_O_3_	[M-H]-	N-acetyl-tryptophan	1	0.47	−1.09	Down			
9	294.1216	295.13	120.08	C_14_H_18_N_2_O_5_	[M + H]^+^	γ-Glutamylphenylalanine	2				2.07	1.05	Up
10	321.0995	322.11	130.05	C_11_H_19_N_3_O_6_S	[M + H]^+^	S-(Methyl)glutathione	1				2.07	1.05	Up
11	384.1216	385.13	250.00	C_14_H_20_N_6_O_5_S	[M + H]^+^	S-(5′-Adenosy)-L-homocysteine	3				2.64	1.40	Up
12	398.1372	399.14	250.09	C_15_H_22_N_6_O_5_S	[M + H]^+^	S-(5′-Adenosyl)-L-methionine	3				4.01	2.00	Up
13	399.1445	399.14	250.09	C_15_H_23_N_6_O_5_S^+^	[M]+	S-Adenosylmethionine	3				4.01	2.00	Up
*Phenolic acids (34)*													
14	110.0368	109.03	81.03	C_6_H_6_O_2_	[M-H]-	Hydroquinone	3				0.35	−1.52	Down
15	120.0575	121.06	77.04	C_8_H_8_O	[M + H]^+^	Phenylacetaldehyde	3	9503.89	13.21	Up	1052.15	10.04	Up
16	126.0317	125.02	79.02	C_6_H_6_O_3_	[M-H]-	Pyrogallol	2				0.50	−1.01	Down
17	150.0681	149.06	131.05	C_9_H_10_O_2_	[M-H]-	p-Coumaryl alcohol	3				2.19	1.13	Up
18	152.0473	153.05	123.04	C_8_H_8_O_3_	[M + H]^+^	Methyl salicylate	3	0.49	−1.03	Down			
19	153.0426	152.04	108.05	C_7_H_7_NO_3_	[M-H]^−^	3-Aminosalicylic acid	3	0.29	−1.80	Down	0.30	−1.73	Down
20	153.0426	152.03	108.05	C_7_H_7_NO_3_	[M-H]^−^	4-Aminosalicylic acid	3	0.39	−1.36	Down	0.31	−1.69	Down
21	154.0266	153.02	109.03	C_7_H_6_O_4_	[M-H]^−^	Gentisic Acid*	1				0.34	−1.58	Down
22	154.0266	153.02	109.03	C_7_H_6_O_4_	[M-H]^−^	Protocatechuic acid*	1				0.34	−1.57	Down
23	154.0266	153.02	109.03	C_7_H_6_O_4_	[M-H]^−^	Methyl cumalate	1				0.34	−1.57	Down
24	168.0423	167.03	123.05	C_8_H_8_O_4_	[M-H]^−^	3,4-Dihydroxybenzeneacetic acid	3	0.48	−1.05	Down			
25	180.0786	179.07	92.03	C_10_H_12_O_3_	[M-H]^−^	Propyl 4-hydroxybenzoate	3	2.63	1.39	Up			
26	208.1099	209.12	179.07	C_12_H_16_O_3_	[M + H]^+^	alpha-Asarone	2	69747.14	16.09	Up	79115.94	16.27	Up
27	212.0685	213.07	195.07	C_10_H_12_O_5_	[M + H]^+^	2,4,5-Trimethoxybenzoic acid	3				0.30	−1.74	Down
28	238.0841	237.08	163.04	C_12_H_14_O_5_	[M-H]^−^	1*-O-*p-Cumaroylglycerol	3	2.26	1.18	Up			
29	268.0947	269.10	177.05	C_13_H_16_O_6_	[M + H]^+^	2-Feruloyl-sn-glycerol	3				0.45	−1.15	Down
30	286.0325	285.03	133.02	C_11_H_10_O_9_	[M-H]^−^	L-Malic acid-2-*O*-gallate	3	2.05	1.04	Up	2.17	1.12	Up
31	308.0896	309.10	147.04	C_15_H_16_O_7_	[M + H]^+^	1-*O*-(p-coumaroyl) 3-Hydroxy-3-methylglutaric acid	1	0.45	−1.17	Down			
32	308.0896	309.10	147.04	C_15_H_18_O_10_	[M + H]^+^	5-*O*-Galloyl-methyl quinine ester	1	0.37	−1.43	Down			
33	326.1002	325.09	145.03	C_15_H_18_O_8_	[M-H]^−^	1-*O*-p-Coumaroyl-β-D-glucose*	2	0.45	−1.15	Down			
34	338.1002	337.09	163.04	C_16_H_18_O_8_	[M-H]^−^	1-*O*-p-Coumaroylquinic acid	1				0.47	−1.08	Down
35	346.0900	345.08	139.04	C_14_H_18_O_10_	[M-H]^−^	1-*O*-(3,4-Dihydroxy-5-methoxy-benzoyl)-glucoside	3	0.32	−1.63	Down			
36	356.1107	355.10	121.03	C_16_H_20_O_9_	[M-H]^−^	Homovanilloylquinic acid	3				0.36	−1.47	Down
37	360.0845	359.08	161.02	C_18_H_16_O_8_	[M-H]^−^	Rosmarinic acid	2	0.12	−3.01	Down	0.00	−9.73	Down
38	360.0851	359.08	197.05	C_18_H_16_O_8_	[M-H]^−^	Salvianic acid B	2	0.12	−3.08	Down	0.00	−7.68	Down
39	360.0851	359.08	197.05	C_29_H_36_O_15_	[M-H]^−^	Magnoloside D	2	345.65	8.43	Up			
40	418.1839	477.20	181.09	C_19_H_30_O_10_	[M + CH3COO]^−^	2-[4-(3-Hydroxypropyl)-2-methoxyphenoxy]-1,3-propanediol 1-glucoside	3				267109.16	18.03	Up
41	466.1264	467.13	147.04	C_25_H_22_O_9_	[M + H]^+^	3,4*-O-*Di-p-Coumaroylshikimic acid	3				0.48	−1.07	Down
42	474.0434	473.04	169.01	C_21_H_14_O_13_	[M-H]^−^	Trigallic acid	2				0.37	−1.45	Down
43	481.0600	481.06	275.00	C_20_H_18_O_14_	[M-H]^−^	4,6-(S)-Hexahydroxydiphenoyl-D-glucose	2				2.20	1.14	Up
44	624.2054	623.20	461.17	C_29_H_36_O_15_	[M-H]^−^	Acteoside	2	13.27	3.73	Up			
45	624.2054	623.20	161.02	C_29_H_36_O_15_	[M-H]^−^	Forsythoside H*	3	465047.40	18.83	Up	1396.47	10.45	Up
46	624.2054	623.20	161.03	C_29_H_36_O_15_	[M-H]^−^	Forsythoside I*	3	337.38	8.40	Up			
47	798.1491	797.14	645.10	C_33_H_34_O_23_	[M-H]^−^	2-*O*-Trigalloyl-glucose–glucose	3				0.00	−14.62	Down
*Flavonoids (89)*													
48	254.0579	255.07	137.02	C_15_H_10_O_4_	[M + H]^+^	6,7-Dihydroxyflavone	3	3.85	1.95	Up			
49	270.0528	271.06	215.07	C_15_H_10_O_5_	[M + H]^+^	4′,6,7-Trihydroxyisoflavone	3				2.07	1.05	Up
50	274.0841	275.09	107.05	C_15_H_14_O_5_	[M + H]^+^	Epiafzelechin	1				2.06	1.05	Up
51	288.0634	287.06	125.03	C_15_H_12_O_6_	[M-H]^−^	2-hydroxynaringenin	2	2.15	1.10	Up	2.29	1.19	Up
52	302.0427	301.04	151.00	C_15_H_10_O_7_	[M-H]^−^	Tricetin	2				0.44	−1.20	Down
53	306.0740	305.07	125.02	C_15_H_14_O_7_	[M-H]^−^	Leucocyanidin	1	0.45	−1.16	Down	0.41	−1.30	Down
54	306.0740	307.08	139.04	C_15_H_14_O_7_	[M + H]^+^	Gallocatechin	2	0.47	−1.09	Down	0.49	−1.04	Down
55	314.1154	315.12	181.05	C_18_H_18_O_5_	[M + H]^+^	Flavokawain A	1	35.05	5.13	Up	0.07	−3.93	Down
56	328.0947	329.10	299.05	C_18_H_16_O_6_	[M + H]^+^	5-Hydroxy-7,3′,4′-trimethoxyflavone	3				0.32	−1.62	Down
57	344.0896	345.10	284.07	C_18_H_16_O_7_	[M + H]^+^	5,7-Dihydroxy-3′,4′,5′-trimethoxyflavone	3				0.33	−1.58	Down
58	360.0845	359.08	161.02	C_18_H_16_O_8_	[M-H]^−^	Myricetin-3,7,3′-trimethyl ether	2	0.14	−2.84	Down	0.00	−9.39	Down
59	366.0046	365.00	285.04	C_15_H_10_O_9_S	[M-H]^−^	Kaempferol-3*-O-*sulfonate	3	0.43	−1.23	Down			
60	381.9995	380.99	301.04	C_15_H_10_O_10_S	[M-H]^−^	Quercetin-3-*O*-Sulfonate	3	0.33	−1.59	Down			
61	397.9944	396.99	317.03	C_15_H_10_O_11_S	[M-H]^−^	Myricetin-3-*O*-sulfonate	3	0.40	−1.33	Down			
62	402.0951	403.10	367.08	C_20_H_18_O_9_	[M + H]^+^	Apigenin-6-C-arabinoside*	2				0.30	−1.71	Down
63	402.0951	403.10	367.08	C_20_H_18_O_9_	[M + H]^+^	Apigenin-8-C-Arabinoside*	2				0.34	−1.54	Down
64	402.1315	403.14	373.09	C_21_H_22_O_8_	[M + H]^+^	3,5,6,7,8,4’-Hexamethoxyflavone	3	0.45	−1.14	Down			
65	418.0900	419.10	383.07	C_20_H_18_O_10_	[M + H]^+^	Luteolin-8-C-arabinoside	3				0.47	−1.09	Down
66	418.1264	419.13	383.10	C_21_H_22_O_9_	[M + H]^+^	*O*-MethylNaringenin-8-C-arabinoside	3				0.50	−1.00	Down
67	432.1056	431.10	311.05	C_21_H_20_O_10_	[M-H]^−^	Isovitexin	1				0.37	−1.43	Down
68	432.1056	433.11	313.07	C_21_H_20_O_10_	[M + H]^+^	Vitexin	1				0.46	−1.13	Down
69	432.1056	433.11	283.06	C_21_H_20_O_10_	[M + H]^+^	Genistein-8-C-glucoside	3	0.49	−1.04	Down	0.32	−1.63	Down
70	432.1420	433.15	403.11	C_22_H_24_O_9_	[M + H]^+^	3,5,6,7,8,3′,4’-Heptamethoxyflavone	3	0.32	−1.65	Down			
71	434.0849	435.09	303.05	C_20_H_18_O_11_	[M + H]^+^	Morin-3-*O*-xyloside	3				0.35	−1.50	Down
72	434.1213	433.11	271.06	C_21_H_22_O_10_	[M-H]^−^	Choerospondin	2	0.46	−1.12	Down			
73	436.1369	435.13	315.09	C_21_H_24_O_10_	[M-H]^−^	Trilobatin	3	0.48	−1.05	Down			
74	448.1006	449.11	303.05	C_21_H_20_O_11_	[M + H]^+^	Quercitrin	3	0.20	−2.31	Down	0.33	−1.59	Down
75	450.1162	449.11	287.06	C_21_H_22_O_11_	[M-H]^−^	Dihydrokaempferol-7-*O-*glucoside	3	59587.87	15.86	Up	69886.92	16.09	Up
76	452.1319	451.12	289.07	C_21_H_24_O_11_	[M-H]^−^	Catechin-5-*O*-glucoside	3	2.99	1.58	Up	2.86	1.52	Up
77	456.1056	455.10	289.07	C_23_H_20_O_10_	[M-H]^−^	Epicatechin-3-(3”-*O*-methyl) gallate	2				0.35	−1.52	Down
78	458.0849	459.09	139.04	C_22_H_18_O_11_	[M + H]^+^	Gallocatechin gallate*	1				0.48	−1.07	Down
79	464.0955	465.10	303.05	C_21_H_20_O_12_	[M + H]^+^	Rhodiolgin	1	0.43	−1.23	Down			
80	464.0955	465.10	303.05	C_21_H_20_O_12_	[M + H]^+^	Quercetin-5-*O*-β-D-glucoside*	1	0.48	−1.06	Down			
81	464.0955	465.10	333.07	C_21_H_20_O_12_	[M + H]^+^	6-Methoxyquercetin-3-*O*-Xyloside	3	0.31	−1.68	Down	0.21	−2.26	Down
82	472.1006	471.10	183.03	C_23_H_20_O_11_	[M-H]^−^	Epigallocatechin 3-*O*-(3*-O*-Methyl) Gallate	3				0.43	−1.21	Down
83	474.1162	475.12	271.06	C_23_H_22_O_11_	[M + H]^+^	Apigenin-7-*O*-(6″-acetyl) glucoside	3	10.78	3.43	Up			
84	478.1475	477.14	315.09	C_23_H_26_O_11_	[M-H]^−^	Persicoside	3				0.38	−1.41	Down
85	480.0904	481.10	319.05	C_21_H_20_O_13_	[M + H] ^+^	Gossypetin-3-*O*-glucoside	2	0.40	−1.34	Down			
86	480.1268	481.13	319.05	C_22_H_24_O_12_	[M + H]^+^	3′,5′,5,7-Tetrahydroxy-4′-methoxyflavanone-3’-*O*-glucoside	1	0.42	−1.25	Down			
87	492.1268	493.13	331.08	C_23_H_24_O_12_	[M + H]^+^	Flavoyadorinin A	3				0.50	−1.01	Down
88	492.1268	493.13	331.08	C_23_H_24_O_12_	[M + H]^+^	Tricin-4’-*O*-glucoside	3				0.43	−1.23	Down
89	492.1268	493.13	331.08	C_23_H_24_O_12_	[M + H]^+^	Iristectorin A	3				0.42	−1.24	Down
90	494.0697	495.08	319.04	C_21_H_18_O_14_	[M + H]^+^	Myricetin-3-*O*-glucuronide	3	3.82	1.93	Up	4.92	2.30	Up
91	494.1060	495.11	333.07	C_22_H_22_O_13_	[M + H]^+^	Laricitrin-3-*O*-glucoside	2	0.40	−1.32	Down	0.49	−1.02	Down
92	494.1060	493.10	331.05	C_22_H_22_O_13_	[M-H]^−^	Mearnsetin-3-*O*-glucoside	2				0.50	−1.01	Down
93	506.1060	505.10	300.03	C_23_H_22_O_13_	[M-H]^−^	Quercetin-3-*O*-(6”-*O*-acetyl) galactoside	3	0.43	−1.23	Down			
94	506.1060	507.12	303.05	C_23_H_22_O_13_	[M + H]^+^	Quercetin-3-*O*-(6”-*O*-acetyl) glucoside	3	0.35	−1.51	Down	0.31	−1.68	Down
95	508.1217	509.13	347.08	C_23_H_24_O_13_	[M + H]^+^	5,6,3′,4’-Tetrahydroxy-3,7-dimethoxyflavone-6-*O*-glucoside	3	0.00	−15.40	Down			
96	508.1217	509.13	347.08	C_23_H_24_O_13_	[M + H]^+^	Limocitrin 3-Glucoside	3	0.00	−15.40	Down			
97	534.1010	535.11	287.05	C_24_H_22_O_14_	[M + H]^+^	Kaempferol-3-*O*-(6″-malonyl) glucoside*	1	0.38	−1.41	Down			
98	536.1166	535.11	287.05	C_24_H_24_O_14_	[M-H]^−^	Eriodictyol-7-*O*-(6″-malonyl) glucoside	2				2.24	1.16	Up
99	550.1323	551.14	497.10	C_25_H_26_O_14_	[M + H]^+^	Luteolin-6,8-di-C-arabinoside	3				0.41	−1.30	Down
100	564.1115	565.12	317.08	C_25_H_24_O_15_	[M + H]^+^	Isorhamnetin-3-*O*-(6″-malonyl) glucoside	3				7621.45	12.90	Up
101	564.1479	565.16	433.10	C_26_H_28_O_14_	[M + H] ^+^	Genistein-8-C-apiosyl (1 → 6) glucoside	3				0.36	−1.47	Down
102	564.1479	565.16	433.11	C_26_H_28_O_14_	[M + H]^+^	Apigenin-6-C-(2″-xylosyl) glucoside	3				0.45	−1.15	Down
103	564.1479	565.15	433.11	C_26_H_28_O_14_	[M + H]^+^	Isovitexin-2”-*O*-xyloside*	3				0.44	−1.17	Down
104	566.1272	567.13	303.05	C_25_H_26_O_15_	[M + H]^+^	Quercetin-3-*O*-xylosyl (1 → 2) arabinoside	3	2280.49	11.16	Up			
105	578.1424	579.15	271.06	C_30_H_26_O_12_	[M + H]^+^	Apigenin-7-*O*-(6″-p-Coumaryl) glucoside	3	0.43	−1.21	Down			
106	578.1636	579.17	433.11	C_27_H_30_O_14_	[M + H]^+^	Vitexin-2”-*O*-rhamnoside	1				0.38	−1.40	Down
107	580.1428	581.15	287.06	C_26_H_28_O_15_	[M + H]^+^	Kaempferol-3-*O*-sambubioside	3	0.47	−1.08	Down			
108	592.1792	593.18	447.13	C_28_H_32_O_14_	[M + H]^+^	Chrysoeriol-6-C-rhamnoside-7-*O*-rhamnoside	3				0.44	−1.20	Down
109	594.1585	593.15	285.04	C_27_H_30_O_15_	[M-H]^−^	Nicotiflorin*	1	0.47	−1.08	Down			
110	594.1585	595.17	287.06	C_27_H_30_O_15_	[M + H]^+^	Kaempferol-3-*O*-glucorhamnoside*	1	0.00	−22.58	Down	0.40	−1.33	Down
111	594.1585	595.16	313.07	C_27_H_30_O_15_	[M + H]^+^	Vitexin-2”-*O*-glucoside	1				0.41	−1.29	Down
112	594.1585	595.17	313.07	C_27_H_30_O_15_	[M + H]^+^	Isosaponarin	1				0.48	−1.05	Down
113	594.1585	595.17	433.11	C_27_H_30_O_15_	[M + H]^+^	Saponarin*	1				0.46	−1.13	Down
114	594.1585	595.17	433.12	C_27_H_30_O_15_	[M + H]^+^	Vitexin-2”-*O*-galactoside*	1				0.40	−1.33	Down
115	594.1585	595.17	271.06	C_27_H_30_O_15_	[M + H]^+^	Apigenin-7-*O*-Gentiobioside	3				0.44	−1.20	Down
116	594.1585	595.17	433.12	C_27_H_30_O_15_	[M + H]^+^	4’-*O*-Glucosylvitexin	3				0.47	−1.09	Down
117	594.1585	595.16	449.11	C_27_H_30_O_15_	[M + H]^+^	Orientin-2”-*O*-rhamnoside	2				0.46	−1.13	Down
118	594.1585	595.17	449.11	C_27_H_30_O_15_	[M + H]^+^	Luteolin-6-C-glucoside-7-*O*-rhamnoside	3				0.43	−1.22	Down
119	598.1898	599.20	431.13	C_27_H_34_O_15_	[M + H]^+^	Phloretin 3′,5’-Di-C-Glucoside	1	2.27	1.18	Up			
120	600.1115	601.12	287.06	C_28_H_24_O_15_	[M + H]^+^	Kaempferol-3-*O*-(2″-galloyl) galactoside*	1	3.73	1.90	Up			
121	600.1115	601.12	287.06	C_28_H_24_O_15_	[M + H]^+^	Kaempferol-3-*O*-(6″-galloyl) galactoside*	1	3.73	1.90	Up			
122	610.1528	611.16	449.11	C_27_H_30_O_16_	[M + H]^+^	Orientin-2”-*O*-galactoside	3				0.48	−1.07	Down
123	614.0908	615.10	153.02	C_28_H_22_O_16_	[M + H]^+^	Kaempferol-3-*O*-(2”-*O*-galloyl) glucuronide	3	2.65	1.41	Up	2.33	1.22	Up
124	624.1690	625.18	317.06	C_28_H_32_O_16_	[M + H]^+^	6-C-Methylquercetin-3-*O*-rutinoside	3	0.50	−1.00	Down	0.49	−1.02	Down
125	624.1690	625.17	301.07	C_28_H_32_O_16_	[M + H]^+^	Chrysoeriol-5,7-di-*O*-glucoside	3				0.42	−1.24	Down
126	624.1690	625.18	317.07	C_28_H_32_O_16_	[M + H]^+^	Isorhamnetin-3-*O*-glucoside-7-*O*-rhamnoside	3				0.46	−1.12	Down
127	624.1690	625.18	487.12	C_28_H_32_O_16_	[M + H]^+^	Chrysoeriol-6,8-di-C-glucoside	3				0.00	−14.14	Down
128	626.1483	627.16	303.05	C_27_H_30_O_17_	[M + H]^+^	6-Hydroxykaempferol-6,7-*O*-Diglucoside*	2	2.43	1.28	Up			
129	626.1847	627.19	465.10	C_28_H_34_O_16_	[M + H]^+^	Hesperetin-8-C-glucoside-3’-*O*-glucoside	3	37838.48	15.21	Up	34878.58	15.09	Up
130	626.1847	627.19	465.20	C_28_H_34_O_16_	[M + H]^+^	Hesperetin-6-C-glucoside-7-*O*-glucoside	3	2.26	1.17	Up			
131	740.2164	741.22	287.06	C_33_H_40_O_19_	[M + H]^+^	Kaempferol-3-*O*-rutinoside-7-*O*-rhamnoside	2	0.00	−14.38	Down			
132	756.2113	757.22	577.20	C_33_H_40_O_20_	[M + H]^+^	Apigenin-6,8-di-C-glucoside-4’-*O*-glucoside	3	0.50	−1.01	Down			
133	772.2062	773.21	303.05	C_33_H_40_O_21_	[M + H]^+^	Quercetin-3-*O*-sophoroside-7-*O*-rhamnoside	3	0.44	−1.20	Down			
134	786.2007	787.21	339.11	C_37_H_38_O_19_	[M + H]^+^	Kaempferol-3-*O*-(6”-Feruloyl) glucosyl-(1 → 4)-galactoside	3	0.35	−1.50	Down	0.30	−1.73	Down
135	802.1956	803.20	339.11	C_37_H_38_O_20_	[M + H]^+^	Quercetin-3-*O*-(2″‘-*O*-feruloyl) sophoroside	3	0.39	−1.36	Down			
136	902.2481	903.26	147.04	C_42_H_46_O_22_	[M + H]^+^	Vitexin-2”-*O*-(6″‘-p-coumaroyl) glucoside-4’-*O*-glucoside	2	2.95	1.56	Up	4.29	2.10	Up
*Lipids (33)*													
137	276.2089	277.22	93.07	C_18_H_28_O_2_	[M + H]^+^	14,15-Dehydrocrepenynic acid	3				2.56	1.36	Up
138	336.2664	337.27	263.24	C_21_H_36_O_3_	[M + H]^+^	(Oxiran-2-yl) methyl octadeca-9,12-dienoate	1				0.34	−1.57	Down
139	338.2457	339.25	121.10	C_20_H_34_O_4_	[M + H]^+^	5,6-DiHETrE [(±)5,6-dihydroxy-8Z,11Z,14Z-eicosatrienoic acid]	3				0.48	−1.05	Down
140	356.2927	357.30	265.25	C_21_H_40_O_4_	[M + H] ^+^	1-Oleoyl-Sn-Glycerol	1				0.41	−1.28	Down
141	447.2386	448.25	307.23	C_21_H_38_NO_7_P	[M + H]^+^	LysoPE 16:3	3				0.39	−1.37	Down
142	465.2855	466.29	325.27	C_22_H_44_NO_7_P	[M + H]^+^	LysoPE 17:1*	3				0.39	−1.36	Down
143	475.2699	476.28	335.26	C_23_H_42_NO_7_P	[M + H]^+^	LysoPE 18:3	1				0.43	−1.22	Down
144	475.2699	476.28	335.26	C_23_H_42_NO_7_P	[M + H]^+^	LysoPE 18:3(2n isomer)	1				0.37	−1.44	Down
145	477.2855	478.29	337.27	C_23_H_44_NO_7_P	[M + H]^+^	LysoPE 18:2	2				0.35	−1.52	Down
146	477.2855	478.29	337.27	C_23_H_44_NO_7_P	[M + H]^+^	LysoPE 18:2(2n isomer)	2				0.36	−1.48	Down
147	479.3012	480.31	339.29	C_23_H_46_NO_7_P	[M + H]^+^	LysoPE 18:1*	3				0.46	−1.13	Down
148	479.3012	480.31	339.29	C_23_H_46_NO_7_P	[M + H]^+^	LysoPE 18:1(2n isomer) *	3				0.46	−1.11	Down
149	481.3168	482.32	341.31	C_23_H_48_NO_7_P	[M + H]^+^	LysoPE 18:0(2n isomer)	3				0.43	−1.22	Down
150	482.2624	481.26	253.22	C_22_H_43_O_9_P	[M-H]^−^	LysoPG 16:1	3				0.19	−2.39	Down
151	493.3168	494.32	184.07	C_24_H_48_NO_7_P	[M + H]^+^	LysoPC 16:1(2n isomer) *	1				0.50	−1.01	Down
152	505.3168	504.31	279.23	C_25_H_48_NO_7_P	[M-H]^−^	1-Linoleoyl-2-Lysophosphatidic Acid Monobutylamine Ester	1				0.41	−1.28	Down
153	516.3298	515.32	279.23	C_27_H_48_O_9_	[M-H]^−^	1-*O*-Linoleoyl-3-*O*-galactopyranosyl-L-glycerol	1				0.23	−2.13	Down
154	517.3168	518.32	184.07	C_26_H_48_NO_7_P	[M + H]^+^	LysoPC 18:3	2				0.41	−1.27	Down
155	519.3325	520.34	184.07	C_26_H_50_NO_7_P	[M + H]^+^	LysoPC 18:2(2n isomer)	2				0.39	−1.37	Down
156	521.3481	522.36	184.07	C_26_H_52_NO_7_P	[M + H]^+^	LysoPC 18:1*	1				0.38	−1.39	Down
157	521.3481	522.36	184.07	C_26_H_52_NO_7_P	[M + H]^+^	LysoPC 18:1(2n isomer) *	1				0.40	−1.33	Down
158	533.3481	534.36	184.07	C_27_H_52_NO_7_P	[M + H]^+^	LysoPC 19:2(2n isomer)	2				0.15	−2.69	Down
159	553.3380	552.33	253.22	C_26_H_52_NO_9_P	[M-H]^−^	2-(2,3-dihydroxypropoxy)-3-(((2-(dimethylamino) ethoxy) (hydroxy)phosphoryl)oxy)propan-2-yl (E)-hexadec-9-enoate	2				0.48	−1.05	Down
160	577.3380	576.33	277.22	C_28_H_52_NO_9_P	[M-H]^−^	2-(2,3-dihydroxypropoxy)-3-(((2-(dimethylamino) ethoxy) (hydroxy) phosphoryl) oxy) propyl (8E,11Z,14Z)-octadeca-8,11,14-trienoate	1				0.41	−1.30	Down
161	581.3693	580.36	281.25	C_28_H_56_NO_9_P	[M-H]^−^	1-(2,3-dihydroxypropoxy)-3-(((2-(dimethylamino) ethoxy) (hydroxy) phosphoryl) oxy) propan-2-yl (Z)-14-Octadecenoic Acid	1				0.48	−1.06	Down
162	581.3693	580.36	281.25	C_28_H_56_NO_9_P	[M-H]^−^	2-(2,3-dihydroxypropoxy)-3-(((2-(dimethylamino) ethoxy) (hydroxy) phosphoryl) oxy) propan-2-yl (Z)-14-Octadecenoic Acid	1				0.47	−1.10	Down
163	654.3827	653.38	397.14	C_31_H_58_O_14_	[M-H]^−^	1-Palmitoyl-Sn-Glycerol 3-*O*-Diglucoside	2				0.46	−1.12	Down
164	654.3827	653.38	397.14	C_31_H_58_O_14_	[M-H]^−^	2-Palmitoyl-Sn-Glycerol 3-*O*-Diglucoside	2				0.42	−1.26	Down
165	676.3670	677.37	261.22	C_33_H_56_O_14_	[M + H]^+^	1-α-Linolenoyl-glycerol-2,3-di-*O*-glucoside	2				0.29	−1.77	Down
166	676.3670	677.37	261.22	C_33_H_56_O_14_	[M + H]^+^	2-α-Linolenoyl-glycerol-1,3-di-*O*-glucoside	1				0.26	−1.95	Down
167	676.3670	675.36	397.14	C_33_H_56_O_14_	[M-H]^−^	Gingerglycolipid A	3				0.29	−1.81	Down
168	678.3827	677.38	397.13	C_33_H_58_O_14_	[M-H]^−^	Gingerglycolipid B	3				0.18	−2.48	Down
169	680.3983	679.39	397.13	C_33_H_60_O_14_	[M-H]^−^	Gingerglycolipid C	3	2.40	1.27	Up			

a Molecular weight of the parent ion of the substance after ionization by the electrospray ion source.

b Characteristic fragment ions.

c Ionization mode (M + H is positively charged, M-H is negatively charged).

d An * indicates the possible presence of isomers.

e Metabolite identification criteriawhere 1 indicates a score of 0.7 or higher for the sample substance secondary mass spectrum (all fragment daughter ions of the substance), RT and database substance match; 2indicates a score of 0.5–0.7 for the sample substance secondary mass spectrum (all fragment daughter ions of the substance), RT and database substance match; 3: sample substance Q1, Q3, RT, DP, CE and database substance match.

f CH_FC and OF_FC indicate the fold change of CK vs FC and CK vs OF metabolites, respectively.

g Fold change were logarithmic with a base of 2.

h Metabolite up-and down-regulation type.

Overall, there was a downward trend in the non-volatile metabolites of black tea after scenting. Compared to CK, phenolic acids were significantly up-regulated in CH (Log2FC = 31.66) whilst flavonoids were significantly down-regulated (Log2FC = −39.00). Amino acids and derivatives were significantly up-regulated in OF (Log2FC = 17.60) whilst flavonoids were significantly down-regulated (Log2FC = −30.43).

#### 3.3.2. Analysis of key differential compounds

**Figure 3:**
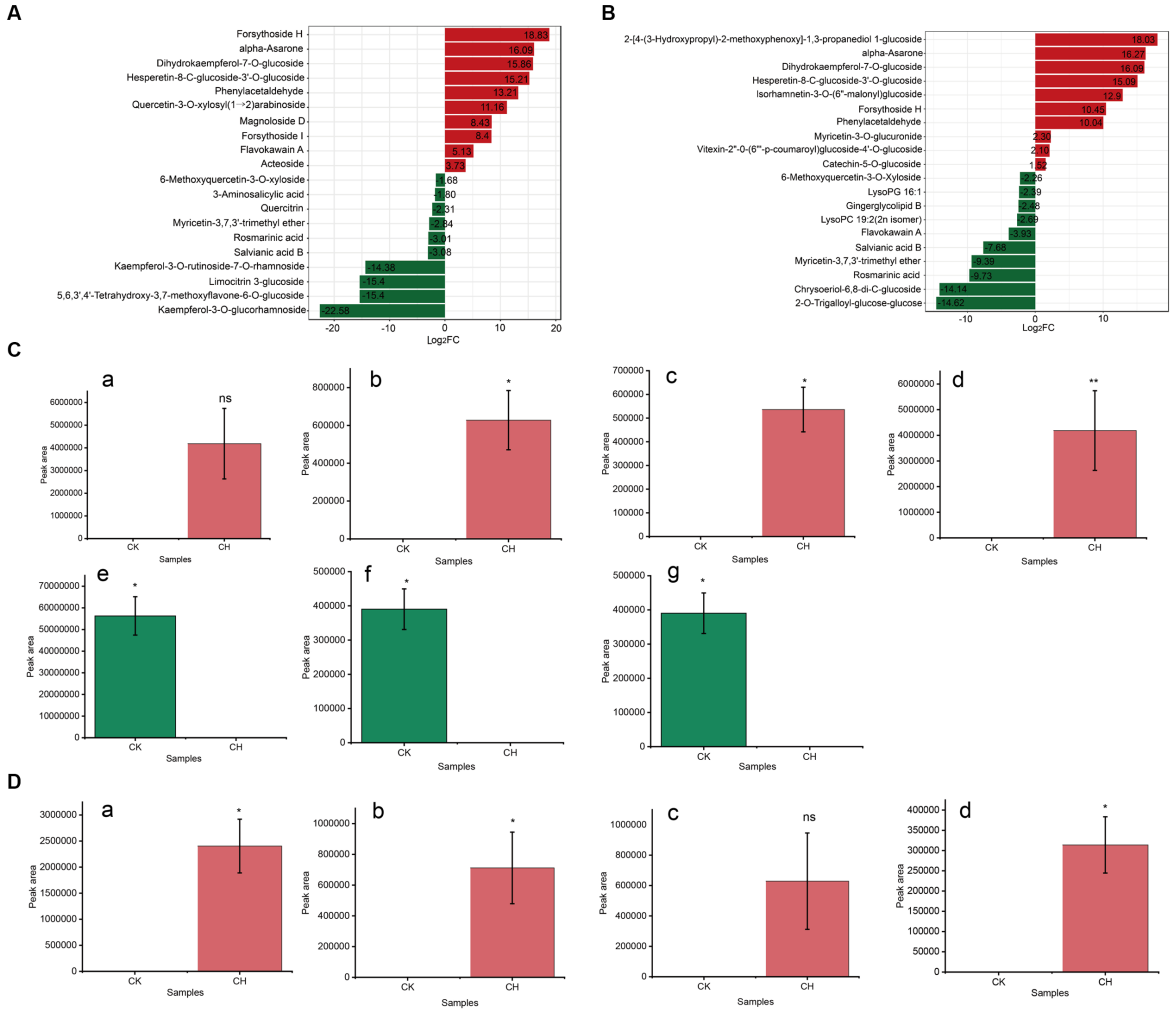
Analysis of key differential non-volatile metabolites in three types of black tea (CK, CH and OF). **(A,B)** Bar charts of the multiplicity of differences between different comparison groups (A. CK vs. CH, B. CK vs. OF); **(C,D)** Abundance histograms of key metabolites (|Log2FC| > 15, C. CK vs. CH; D. CK vs. OF). C-a, Forsythoside H; C-b, alpha-Asarone; C-c, Dihydrokaempferol-7-O-glucoside; C-d, Hes-peretin-8-C-glucoside-3’-O-glucoside; C-e, Kaempferol-3-O-glucorhamnoside; C-f, 5,6,3′,4’-Tetrahydroxy-3,7-dimethoxyflavone-6-O-glucoside; C-g, Limocitrin 3-Glucoside; D-a, 2-[4-(3-Hydroxypropyl)-2-methoxyphenoxy]-1,3-propanediol 1-glucoside; D-b, alpha-Asarone; D-c, Dihydrokaempferol-7-O-glucoside; D-d, Hesperetin-8-C-glucoside-3’-O-glucoside. * Indicated a significant difference between treated samples at the 0.05 level; ** indicated a significant difference at the 0.01 level and ‘ns’ indicated a non-significant difference.

Based on the values of magnitude of the difference multiplier (Log2FC) in each comparison group (CK vs. CH, CK vs. OF), the substances ranked the top 20 of the key non-volatile differential metabolites were prioritized for further discussion. As illustrated in Figures 3A,B, the Log2FC of the selected metabolites ranged from −22.58 to 18.83 in CK vs. CH and − 14.62 to 18.03 in CK vs. OF. Based on |Log2FC| > 15, 7 and 4 substances were screened as key differential compounds in CH (Figure 3C) and OF (Figure 3D), respectively.

The results revealed that the 11 substances were divided into two main groups, which were flavonoids and phenolic acids. Combined with the relevant information in Table 1, it was concluded that compared to CK, forsythoside H, alpha-asarone, dihydrokaempferol-7-*O*-glucoside, and hesperetin-8-C-glucoside-3’-*O*-glucoside in CH were increased, with FC levels of 465047.40, 69747.14, 59587.87, and 37838.48, respectively. Forsythoside H, a caffeoyl phenylethanoid glycoside (CPG) isolated from Forsythia suspense (Thunb.) Vahl, was a type of forsythoside and might possess anti-inflammatory activity. Alpha-asarone was a polyphenol with anti-bacterial and anti-inflammatory activities. It was worth noting that the level of dihydrokaempferol-7-*O*-glucoside was reported to reduce during the withering process of CK (17), which was inconsistent with our results. It suggested that reactions during different stages of black tea processing were not the same. On the contrary, the relative abundances of three substances, including kaempferol-3-*O*-glucorhamnoside, 5,6,3′,4′-tetrahydroxy-3,7-dimethoxyflavone-6-*O*-glucoside, and limocitrin 3-glucoside, decreased significantly and the metabolites were significantly down-regulated compared to CK. Limocitrin 3-glucoside, as a flavonoid, which was previously detected in hickory seeds (27). In OF, the relative abundances of 2-[4-(3-Hydroxypropyl)-2-methoxyphenoxy]-1,3-propanediol 1-glucoside, alpha-asarone, dihydrokaempferol-7-*O*-glucoside, and hesperetin-8-C-glucoside-3’-*O*-glucoside were significantly increased compared to CK and the FC values ranged from 34878.58 to 267109.16. Among them, the latter three substances were common to both scented teas.

In summary, flavonoids and phenolic acids were key differential non-volatile metabolites between unscented and scented black teas. The relative abundances of dihydrokaempferol-7-*O*-glucoside, hesperetin-8-C-glucoside-3’-*O*-glucoside, and alpha-asarone significantly increased in scented teas (CH & OF). There was a significant decrease in kaempferol-3-*O*-glucorhamnoside, 5,6,3′,4′-tetrahydroxy-3,7-dimethoxyflavone-6-*O*-glucoside, and limocitrin 3-glucoside in CH, while the relative abundance of 2-[4-(3-Hydroxypropyl)-2-methoxyphenoxy]-1,3-propanediol 1-glucoside was significantly increased in OF. This result was in general concordance with the results of KEGG metabolic pathways analysis (Figures 2H,I).

### 3.4. Analysis of key differential volatile metabolites among the three black teas

#### 3.4.1. Analysis of key differential categories

Based on the percentage of metabolite categories, all volatile metabolites were further screened and 110 metabolites with aroma contribution were selected, including 11 alcohols, 9 ketones, 29 esters, 31 terpenoids, 14 heterocyclic compounds, 4 aldehydes, 5 aromatics, 3 acids, 3 nitrogen compounds, and 1 phenol (Table 2). It was consistent with a previous study which revealed that terpenoids were the most important aroma components in CK (28). The sum of relative abundances of esters, terpenoids, heterocyclic compounds, alcohols, ketones and aromatics was 97.08% in CK. Compared to CK, the relative abundances of heterocyclic compounds and alcohols in CH decreased by 19.62 and 11.49%, respectively, while aromatics increased by 21.23%. In OF, the relative abundances of heterocyclic compounds and esters increased by 17.21 and 13.59%, respectively, while the relative abundances of terpenoids and ketones showed a decreasing trend, reaching 14.87 and 10.31%, respectively (Supplementary Table S2). A previous study found a significant increase in both ketones and esters during the scenting of *Osmanthus* black tea (14), which was not consistent with the results of this study and may be influenced by the variety of tea leaves, tea processing parameters and scenting process.

**Table 2:** Key differential volatile metabolites changes among the three black teas.

RI^a^	Quan. Ion^b^	Qual. Ion^c^	Compounds	Odor^d^	CH_FC^e^	CH_Log_2_FC^f^	CH_Type^g^	OF_FC^e^	OF_Log_2_FC^f^	OF_Type^g^
*Alcohols (11)*										
766	42	55	1-Pentanol	Plastic, moderately strong, green, fusel oil-like	0.32	−1.66	Down			
851	98	41	Furfuryl alcohol	Alcoholic, chemical, musty, sweet, caramel, bread, coffee				2.10	1.07	Up
862	57	41	(E)-2-Hexen-1-ol	Fresh, green, leafy, fruity, unripe banana				2.01	1.01	Up
970	70	56	1-Heptanol	Grassy	0.26	−1.97	Down			
1,004	79	77	(E, E)-2,4-Heptadien-1-ol	Green, fruity, nutty, cheese				2.06	1.04	Up
1,030	57	41	2-Ethyl-1-hexanol	Citrus, fresh, floral, oily, sweet	2.49	1.32	Up			
1,143	55	41	(E)-3-Nonen-1-ol	Green, waxy, melon, cucumber	2.11	1.08	Up			
1,371	55	69	1-Undecanol	Fresh, waxy, rose, soapy, clean, clothes, floral, citrus				2.20	1.14	Up
1,378	41	55	2-Butyl-2-octenal	Aldehydic, green, watery, fruity, metallic, oily, tropical, fatty, sweaty, goaty	0.42	−1.27	Down			
1,382	91	73	3-Methyl-1-phenyl-3-pentanol	Sweet, fresh, cayloxol, lilac, anisic, earthy, narcissus, peony	6.04	2.59	Up			
1,474	55	69	1-Dodecanol	Earthy, soapy, waxy, fatty, honey, coconut	3.91	1.97	Up	0.16	−2.65	Down
*Ketones (9)*										
745	100	71	2-Methyl-3-pentanone	Mint	0.28	−1.84	Down			
798	83	55	4-Methyl-3-penten-2-one	Pungent, earthy, vegetable, acrylic	0.43	−1.22	Down			
881	55	70	1-Hepten-3-one	Metallic				3.16	1.66	Up
895	58	71	2-Heptanone	Fruity, spicy, sweet, herbal, coconut, woody	0.34	−1.55	Down			
972	69	41	5-Methyl-(E)-2-hepten-4-one	Hazelnut, nutty	0.24	−2.04	Down			
1,035	111	55	(E)-3-Octen-2-one	Herbaceous, mushroom	0.46	−1.13	Down			
1,374	105	77	1-Phenyl-1-pentanone	Balsam, valerian				0.46	−1.13	Down
1,433	121	161	Dihydro-beta-ionone	Earthy, woody, mahogany, orris, dry, amber	45.10	5.50	Up			
1,513	135	77	1-(4-methoxyphenyl)-1-propanone	Musty, anisic	7.17	2.84	Up			
*Esters (29)*										
876	70	55	3-Methyl-1-butanol acetate	Sweet, fruity, banana, green, ripe				2.60	1.38	Up
910	70	61	Acetic acid pentyl ester	Fruity, ba	0.37	−1.44	Down	2.18	1.12	Up
925	74	87	Hexanoic acid methyl ester	Ethereal, fruity, pineapple, apricot, strawberry, tropical, fruit, banana, bacon	0.30	−1.73	Down			
1,009	82	67	(Z)-acetate 3-Hexen-1-ol	Fresh, green, sweet, fruity, banana, apple, grassy				2.04	1.03	Up
1,107	70	85	3-Methyl-butanoic acid 2-methylbutyl ester	Herbal, fruity, earthy, cheese, apple, green	7.50	2.91	Up			
1,129	70	43	Acetic acid 2-ethylhexyl ester	Earthy, herbal, humus, undergrowth				2.92	1.55	Up
1,136	83	55	Cyclohexanecarboxylic acid ethyl ester	Fruity, cheese, winey	2.02	1.01	Up			
1,236	103	57	2-Methyl-butanoic acid hexyl ester	Green, waxy, fruity, apple, spicy, tropical				2.02	1.01	Up
1,299	91	108	2-Methyl-propanoic acid phenylmethyl ester	Jasmin, oily, fruity, sweet, rose, tropical	2.01	1.01	Up	2.08	1.06	Up
1,301	69	41	Geranyl formate	Fresh, rose, neroli, tea, rose, green				2.40	1.27	Up
1,347	108	91	Butanoic acid phenylmethyl ester	Fresh, fruity, jasmin, apricot, loganberry	3.40	1.77	Up			
1,379	131	103	(E)-3-Phenyl-2-propenoic acid methyl ester	Sweet, balsam, strawberry, cherry				0.12	−3.07	Down
1,393	91	136	Benzeneacetic acid 2-methylpropyl ester	Sweet, floral, honey, chocolate, amber	5.95	2.57	Up			
1,404	164	132	2-(Dimethylamino)-benzoic acid methyl ester	Fruity, orange, leaf, petitgrain				2.25	1.17	Up
1,408	165	105	2-(Methylamino)-Benzoic acid methyl ester	Fruity, musty, sweet, neroli, powdery, phenolic, wine				2.05	1.03	Up
1,435	95	55	Cyclohexanepropanoic acid 2-propenyl ester	Sweet, pineapple, tropical, fruity, candy, waxy	30.783	4.944	Up			
1,444	104	105	beta-Phenylethyl butyrate	Musty, sweet, floral, yeast, strawberry	2.005	1.004	Up			
1,463	131	103	(E)-3-Phenyl-2-propenoic acid ethyl ester	Floral, honey, balsamic				0.34	−1.55	Down
1,464	129	100	Diisopropyl adipate	Mild, estery, fatty, sour				0.42	−1.26	Down
1,466	91	119	Myrtenyl isobutyrate	Fruity, woody, pine	0.336	−1.572	Down	0.09	−3.46	Down
1,471	85	29	gamma-Decalactone	Fresh, oily, waxy, peach, coconut, buttery, sweet	400.922	8.647	Up			
1,475	69	93	Neryl isobutyrate	Sweet, fresh, fruity, raspberry, strawberry, green				0.00	−13.07	Down
1,476	71	95	Bornyl butyrate	Herbal, woody	4183.310	12.030	Up			
1,488	115	71	2-Phenoxyethyl isobutyrate	Green, fruity, waxy, apple, nuances	10.170	3.346	Up			
1,491	191	131	4-(1,1-dimethylethyl)-benzeneacetic acid methyl ester	Dairy, buttermilk, leafy, creamy, green, waxy, fatty, hyacinth, melon, rind, rubbery	47.075	5.557	Up			
1,514	69	68	Geranyl isobutyrate	Sweet, floral, fruity, green, peach, apricot, rose	10.971	3.456	Up	0.41	−1.30	Down
1,546	56	57	n-Capric acid isobutyl ester	Oily, sweet, brandy, apricot, fermented, cognac	35.991	5.170	Up			
1,656	83	82	Methyl dihydrojasmonate	Floral, oily, jasmin, green, lactonic, tropical, natural	7.996	2.999	Up	0.09	−3.41	Down
1926	74	87	Hexadecanoic acid methyl ester	Oily, waxy, fatty, orris				0.46	−1.12	Down
*Terpenoids (31)*										
937	93	91	alpha-Pinene	Fresh, camphor, sweet, pine, earthy, woody	5.137	2.361	Up	0.22	−2.19	Down
972	93	91	Sabinene	Woody, terpene, citrus, pine, spice	8.945	3.161	Up	0.00	−14.55	Down
1,138	108	67	trans-Limonene oxide	Fresh, citrus				0.46	−1.11	Down
1,212	81	67	Pulegone	Minty	21.364	4.417	Up			
1,285	95	93	Bornyl acetate	Woody, pine, herbal, cedar, spice				2.21	1.14	Up
1,306	136	121	Dihydrocarvyl acetate	Floral, rose, cuminseed, sweet, minty				2.29	1.19	Up
1,355	69	41	Geranic acid	Green	2.757	1.463	Up			
1,376	161	119	Copaene	Woody, spicy, honey	6.841	2.774	Up			
1,384	81	80	(−)-beta-Bourbonene	Herbal, woody, floral, balsamic	2.407	1.267	Up			
1,406	41	93	Isocaryophyllene	Woody, spicy				2.36	1.24	Up
1,422	121	93	Ionone	Violet, sweet, floral, woody	11.222	3.488	Up	3.33	1.74	Up
1,435	119	93	trans-alpha-Bergamotene	Woody, warm, tea	64.573	6.013	Up			
1,444	41	69	cis-beta-Farnesene	CItrus, green	51533.833	15.653	Up			
1,457	41	69	(E)-beta-Famesene	Woody, citrus, herbal, sweet	2.462	1.300	Up			
1,473	161	81	(+)-gamma-Gurjunene	Musty	0.302	−1.729	Down	0.04	−4.78	Down
1,473	123	41	beta-Ionone epoxide	Fruity, sweet, berry, woody, violet, orris, powdery	2.719	1.443	Up	0.00	−10.16	Down
1,479	189	133	gamma-Selinene	Woody	64.683	6.015	Up			
1,483	119	132	alpha-curcumene	Herbal	8.013	3.002	Up			
1,486	41	105	β-selinene	Herbal	8.151	3.027	Up			
1,490	161	105	beta-Guaiene	Sweet, woody, dry, guaiacwood, spicy, powdery	9.407	3.234	Up			
1,495	119	93	Zingiberene	Spice, fresh, sharp	5.804	2.537	Up	0.31	−1.69	Down
1,495	121	93	(+)-Bicyclogermacrene	Green, woody, weedy	7.295	2.867	Up	0.15	−2.71	Down
1,508	41	93	alpha-Farnesene	Citrus, herbal, lavender, bergamot, myrrh, neroli, green	15.290	3.935	Up			
1,513	161	105	gamma-Cadinene	Herbal, woody	14.136	3.821	Up	0.44	−1.17	Down
1,542	157	142	alpha-Calacorene	Woody	2.161	1.112	Up			
1,544	69	41	d-Nerolidol	Mild, floral	116.773	6.868	Up			
1,600	95	150	Cedrol	Cedarwood, woody, dry, sweet, soft	54.332	5.764	Up			
1,656	69	41	Farnesal	Floral, minty	4.910	2.296	Up	0.48	−1.06	Down
1,658	81	83	Citronellyl tiglate	Rose, fruity, floral, geranium, leafy, tea	6.324	2.661	Up	0.32	−1.62	Down
1,671	82	93	beta-Bisabolol	Citrus, floral, tangy, lemon, fresh, sweet, herbal	7.603	2.927	Up	0.11	−3.15	Down
1,695	93	55	beta-Sinensal	Orange, sweet, fresh, waxy, juicy	9.257	3.211	Up			
*Heterocyclic compounds (14)*										
816	93	66	2-Methyl-pyridine	Sweat				0.43	−1.23	Down
829	94	67	Methyl pyrazine	Nutty, cocoa, roasted, chocolate, peanut, green				0.49	−1.03	Down
904	110	40	Methoxy pyrazine	Sweet, nutty, cocoa	0.422	−1.245	Down	0.40	−1.31	Down
910	95	110	1-(2-Furanyl)-ethanone	Nut-like, sweet, roast	0.422	−1.245	Down			
966	83	55	5-Ethyl-2(5H)-furanone	Spice	0.485	−1.043	Down			
976	107	51	4-Pyridinecarboxaldehyde	Fruity	6.444	2.688	Up	0.37	−1.42	Down
1,065	81	82	2-Ethoxy-3-methylpyrazine	Hazelnut, roasted, almonds, pineapple, earthy				2.43	1.28	Up
1,081	135	136	3-Ethyl-2,5-dimethyl-pyrazine	Potato, cocoa, roasted, nutty				0.43	−1.20	Down
1,084	135	136	2-Ethyl-3,5-dimethyl-pyrazine	Burnt, almonds, roasted, nuts, coffee				0.38	−1.38	Down
1,277	97	98	2-Hexyl-thiophene	Floral, fruity, gassy, green, meaty				2.30	1.20	Up
1,282	125	168	2-Butyl-5-ethyl-thiophene	Fruity, berry, earthy				2.28	1.19	Up
1,317	122	135	2,5-Dimethyl-3-(3-methylbutyl)-pyrazine	Fruity				0.36	−1.48	Down
1,440	146	118	Coumarin	Sweet, hay, tonka, new mown hay	21.458	4.423	Up	0.01	−6.60	Down
1,501	97	68	Massoia lactone	Coconut, driedfigs	5.658	2.500	Up			
*Aldehydes (4)*										
1,283	79	107	alpha-Terpinen-7-al	Fatty, spicy				2.53	1.34	Up
1,430	81	41	(E, E)-2,4-Undecadienal	Oily, caramellic, spicy, citrus, buttery, baked	48.858	5.611	Up			
1,449	69	70	2,6-Dodecadien-1-al	Citrus, mandarin, orange, melon	4.100	2.036	Up			
1,468	41	70	(E)-2 Dodecenal	Citrus, metallic, mandarin, orange, waxy, aldehydic	2.016	1.012	Up	0.11	−3.25	Down
*Aromatics (5)*										
763	91	92	Toluene	Sweet	0.343	−1.544	Down			
1,148	138	95	1,2-Dimethoxy-benzene	Sweet, creamy, vanilla, phenolic, musty	2.385	1.254	Up	3.41	1.77	Up
1,430	112	41	Geosmin	Fresh, musty, earthy, soil	26.482	4.727	Up			
1,492	178	163	(E)-Methyl isoeugenol	Spicy, clove, blossom, cartion, woody	26.994	4.755	Up			
1,513	205	57	Butylated hydroxytoluene	Mild, phenolic, camphor	6.030	2.592	Up			
*Acids (3)*										
974	41	69	2-Methyl-2-pentenoic acid	Dry, acid, sweaty, strawberry, woody, fruity, jam	22.103	4.466	Up	0.00	−10.24	Down
1,356	91	104	Hydrocinnamic acid	Sweet, fatty, rose, musk, cinnamon	2.268	1.182	Up			
1,446	115	134	Acetic acid cinnamyl ester	Sweet, floral, spicy, balsam, cinnamon	45.323	5.502	Up			
*Nitrogen compounds (3)*										
1,283	121	136	p-Menthene-8-thiol	Sulfury, aromatic, grapefruit, naphthyl, resinous, woody	0.458	−1.127	Down			
1,328	182	75	Dipropyltrisulfane	Sulfurous, green, onion, garlic, tropical	0.410	−1.285	Down	0.43	−1.22	Down
1,490	97	41	Dodecanenitrile	Dry, citrus, orange, peel, metallic, spicy	5.906	2.562	Up			
*Phenols (1)*										
1,282	137	152	4-Ethyl-2-methoxy-phenol	Clove, candy	3.531	1.820	Up	3.84	1.94	Up

a Retention index of metabolites on a non-polar column.

b Quantitative ions.

c Qualitative ions.

d Aroma description of the substance (http://www.thegoodscentscompany.com, http://perflavory.com/, http://www.odour.org.uk, http://foodflavorlab.cn).

e CH_FC and OF_FC indicate the fold change of CK vs FC and CK vs OF metabolites, respectively.

f Fold change were logarithmic with a base of 2.

g Metabolite up-and down-regulation type.

Overall, compared to CK, the sum of key volatile metabolites was significantly up-regulated (Log2FC = 187.05) in CH and down-regulated (Log2FC = −77.28) in OF. In particular, esters, terpenoids, heterocyclic compounds, aldehydes, acids, nitrogen compounds were down-regulated, while alcohols, ketones, aromatics and phenols were up-regulated.

#### 3.4.2. Analysis of key differential compounds

**Figure 4:**
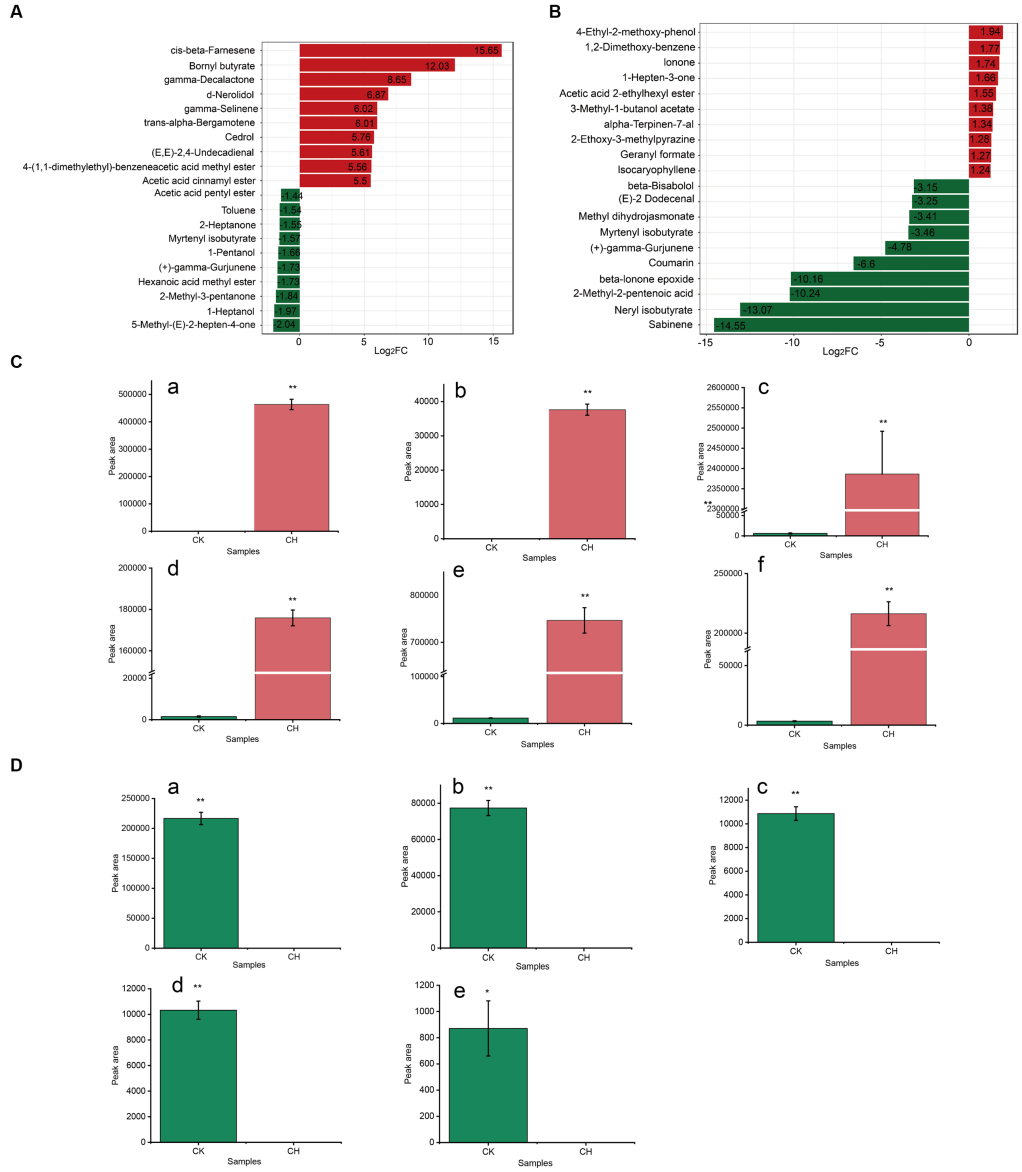
Analysis of key differential volatile metabolites in three types of black tea (CK, CH and OF). **(A,B)** Bar charts of the multiplicity of differences between different comparison groups (A. CK vs. CH, B. CK vs. OF); **(C,D)** Abundance histograms of key metabolites (|Log2FC| > 6, C. CK vs. CH; D. CK vs. OF). C-a, cis-beta-Farnesene; C-b, Bornyl butyrate; C-c, gamma-Decalactone; C-d, d-Nerolidol; C-e, gamma-Selinene; C-f, trans-alpha-Bergamotene; D-a, Sabinene; D-b, Neryl isobutyrate; D-c, 2-Methyl-2-pentenoic acid; D-d, beta-Ionone epoxide; D-e, Coumarin. * Indicated a significant difference between treated samples at the 0.05 level; ** indicated a significant difference at the 0.01 level.

Based on the magnitude of Log2FC in each comparison group (CK vs. CH, CK vs. OF), the top 20 substances in the key volatile differential metabolites were selected preferentially and their FCs were shown in Figures 4A,B. It was found that the selected metabolites were significantly up-regulated in CH compared to CK, while those in OF were significantly down-regulated. Based on |Log2FC| > 6, six and five substances were screened as key differential compounds in CH (Figure 4C) and OF (Figure 4D), respectively.

Among them, the terpenoids and ester accounted for a greater number, with six and three varieties, respectively. Acids and heterocyclic compounds each accounted for one. Combined with the relevant information in Table 2, the relative abundances of cis-beta-farnesene, bornyl butyrate, gamma-decalactone, d-nerolidol, gamma-selinene, and trans-alpha-bergamotene were significantly increased in CH (compared to CK) and FC values reached 51533.83, 4183.31, 400.92, 116.77, 64.68, 64.57, respectively. The cis-beta-farnesene and trans-alpha-bergamotene were significantly associated with floral aroma (28, 29). Trans-alpha-bergamotene was a citrus odorant which was considered to be the most important sesquiterpene in the characteristic odour of *Polygonum minus* (30). Bornyl butyrate, which presented an herbaceous aroma (31), usually presented in the *Aloysia triphylla* and *Aloysia citrodora*.^4^ Gamma-decalactone, which delivered a pleasant peachy aroma, was regarded as one of the characteristic components contributing to the *Osmanthus* aroma of *Osmanthus* black tea (14). Nerolidol was considered to be one of the skeleton aroma components of CK (32). The decrease of nerolidol after scenting might be related to the drying process, as it was previously reported that there was a significant positive correlation between the content of nerolidol and the degree of roasting (33). Gamma-selinene, as a sesquiterpene with floral aroma, was detected in the essential oils from *Chloranthus spicatus* flowers (34).

Nevertheless, in OF, sabinene, neryl isobutyrate, 2-methyl-2-pentenoic acid, beta-ionone epoxide, and coumarin were significantly down-regulated (FC ≤ 0.01). Sabinene, a representative of the bicyclic monoterpenes, conveyed a typical citrus note (35). Beta-ionone epoxide (sweet berry aroma), an apo carotenoid monoterpenoid, was found decreased in summer green tea (*Camellia sinensis L.*) after yellowing (36) and steamed green tea after baking (37), which was consistent with the results of this study. Neryl isobutyrate rendered a sweet rose scent and 2-methyl-2-pentenoic acid offered a fruity note (31). Chen (38) and Su et al. (32) both revealed that coumarin was one of the key substances in the formation of the typical aroma of special grade *Keemun* black tea. The coumarin was described as a sweet grass and cherry blossom-like aroma, which was formed by intramolecular esterification of hydroxycinnamic acid following hydrolysis of 2-coumaric acid primeveroside (18). The reduction of coumarin in OF might be related to the drying process (39).

In conclusion, terpenes, including monoterpenes and sesquiterpenes, were the main differential category. The increase of cis-beta-farnesene and bornyl butyrate might contribute to an increased in floral, fruity and herbaceous aromas in CH. The decrease of sabinene and neryl isobutyrate might be related to the insignificant sweet fruity aroma in OF. The result was generally consistent with the results of KEGG metabolic pathways analysis (Figures 2H,I). As such, potential differential metabolites between the CK vs. CH and CK vs. OF comparator groups were characterized in scented teas with CK as the tea raw material.

## 4. Conclusion

The full-spectrum metabolomics (UPLC-MS/MS and GC–MS) adopted in this study permits a more comprehensive and objective characterization of the differences in non-volatile and volatile flavor metabolites among unscented and scented Congou black teas. The results indicated that the scenting process and the types of flowers had a significant effect on the flavor quality of Congou black tea. Flavonoids, phenolic acids and terpenoids were screened as key differential categories by PCA and PLS-DA, with most significant changes in terpenoids in different scented teas. Floral, fruity and herbaceous volatiles were significantly up-regulated in CH, whilst the sweet fruity volatiles were significantly down-regulated. This study is the first to characterize the flavor profiles of CH and OF in some detail, the results enrich the research on flavor metabolisms of scented black tea and has certain implications for the processing and quality control of scented black tea. Future research needs to broaden the range of other types of flowers and to extend this study to the other five major tea categories, so as to find the most suitable flowers for each tea category to better guide the production of scented teas.

## Data availability statement

The original contributions presented in the study are included in the article/Supplementary material, further inquiries can be directed to the corresponding authors.

## Author contributions

PT, J-QW, YG, and Y-QX: conceptualization. PT and J-QW: data curation and formal analysis and writing—review and editing. PT and Y-QX: funding acquisition. Y-FW, J-CJ, XM, and YZ: investigation. PT, J-QW, YG, and Y-QX: methodology. Y-QX: project administration and validation. YG and Y-QX: supervision. All authors have read and agreed to the published version of the manuscript.

## Funding

This work was supported by the Agricultural and Social Development General Program of Hangzhou (20201203B100), the Key Research and Development Program of Zhejiang (2022C02033), the Central Public-interest Scientific Institution Basal Research Fund (Y2023XK11), and the China Agriculture Research System of MOF and MARA (CARS-19).

## Conflict of interest

Y-FW was employed by Jingdezhen Jin Gui Yuan Agricultural Development Co Ltd.

The remaining authors declare that the research was conducted in the absence of any commercial or financial relationships that could be construed as a potential conflict of interest.

## Publisher’s note

All claims expressed in this article are solely those of the authors and do not necessarily represent those of their affiliated organizations, or those of the publisher, the editors and the reviewers. Any product that may be evaluated in this article, or claim that may be made by its manufacturer, is not guaranteed or endorsed by the publisher.

## Footnotes

^1^www.r-project.org/

^2^http://www.kegg.jp/kegg/compound/

^3^http://www.kegg.jp/kegg/pathway.html

^4^https://lotus.naturalproducts.net/

## Glossary

CK: *Congou* black tea
CH: *Chloranthus spicatus* black tea
OF: *Osmanthus* black tea
UPLC-MS/MS: ultra-performance liquid chromatography tandem mass spectrometry
GC–MS: gas chromatography–mass spectrometry
ESI: electrospray ionization temperature
IS: ion spray
GSI I (II): ion source gases I (II)
CUR: curtain gas
MRM: multiple reaction monitoring
QQQ: triple quadrupole
DP: declustering potential
CE: collision energy
MWDB: metware database
HS-SPME: headspace solid phase microextraction
DVB/CWR/PDMS: divinylbenzene/carboxen/polydimethylsiloxane
EI: electron bombardment ion source
SIM: selected ion detection mode
RI: retention index
RT: retention time
PCA: principal component analysis
PLS-DA: partial least squares-discriminant analysis
KEGG: kyoto encyclopedia of genes and genomes
MSEA: metabolite set enrichment analysis
VIP: variable importance in the project
FC: fold change
DA: differential abundance
MVA: cytoplasmic mevalonate
IPP: isopentenyl pyrophosphates
DMAPP: dimethyl allyl pyrophosphates
CPG: caffeoyl phenylethanoid glycoside
