# Supplementary material for: Comparison analysis of full-spectrum metabolomics revealed on the variation of potential metabolites of unscented, Chloranthus spicatus scented, and Osmanthus fragrans (Thunb.) Lour. scented Congou black teas
Source: Front Nutr. 2023 Aug 14;10:1234807. doi: 10.3389/fnut.2023.1234807 (PMC10461629; doi:10.3389/fnut.2023.1234807)
Supplement: Supplementary file 1 [file Data_Sheet_1.docx]

*Supplementary materials*

Comparison Analysis of Full-Spectrum Metabolomics Revealed on the Variation of Potential Metabolites of Unscented, *Chloranthus Spicatus* Scented, and *Osmanthus Fragrans (Thunb.)* Lour. Scented *Congou* Black Teas

Ping Tang ^1, a^, Jie-Qiong Wang ^2, 3, a^, Yong-Feng Wang ^4^, Jian-Chang Jin ^5^, Xin Meng ^6^, Yan Zhu ^6^, Ying Gao ^2, *^, Yong-Quan Xu ^2, *^

^1^ Hangzhou Vocational & Technical College, 68 Xueyuan Road, Hangzhou 310018, China; 21009359@qq.com (P.T.)

^2^ Tea Research Institute Chinese Academy of Agricultural Sciences, Key Laboratory of Biology, Genetics and Breeding of Special Economic Animals and Plants, Ministry of Agriculture and Rural Affairs, 9 South Meiling Road, Hangzhou 310008, China; wangjieqiong@tricaas.com (J.-Q.W.)

^3^ College of Food Science, Southwest University, Chongqing 400715, China; wangjieqiong@tricaas.com (J.-Q.W.)

^4^ Jindezhen Jin Gui Yuan Agricultural Development Co Ltd, Jindezhen 333000, China; 1340438775@qq.com (Y.-F.W.)

^5^ College of Biological and Environmental Engneering, Zhejiang Shuren University, Hangzhou 310015, China; jinjianchang2004@163.com (J.-C.J.)

^6^ College of Food and health, Zhejiang A & F University, Hangzhou 310015, China; mengxin@tricaas.com (X.-M.); 36833873@qq.com (Y.Z.)

^*^ Correspondence: yinggao@tricaas.com (Y.G.); yqx33@126.com (Y.-Q.X.); Tel.: 0571-86650594 (Y.G.); +86-571-86650594 (Y.-Q.X.); Fax: +86-571-86650056 (Y.-Q.X.)

^a^ These authors have contributed equally to this work.

**TABLE S1.** Comparison of the abundance of important non-volatile differential metabolites in three samples.

| Index^a^ | Q1 (Da)^b^ | Q3 (Da) ^c^ | Compounds | Level^d^ | CK_1^e^ | CK_2 ^e^ | CK_3 ^e^ | OF_1 ^e^ | OF_2 ^e^ | OF_3 ^e^ | CH_1 ^e^ | CH_2 ^e^ | CH_3 ^e^ | QC01^f^ | QC02^f^ | QC03 ^f^ |
| --- | --- | --- | --- | --- | --- | --- | --- | --- | --- | --- | --- | --- | --- | --- | --- | --- |
| 1 | 147.08 | 115.05 | Dihydronaphthalenone | 2 | 208716 | 211394 | 210678 | 212842 | 201105 | 201657 | 185977 | 163852 | 161268 | 189002 | 170910 | 213513 |
| 2 | 179.07 | 91.06 | Coniferaldehyde | 1 | 535741 | 648441 | 559764 | 752595 | 663953 | 710916 | 502910 | 556201 | 588143 | 616195 | 636200 | 610870 |
| 3 | 195.06 | 93.03 | Vanillin acetate | 3 | 1E+06 | 1E+06 | 1E+06 | 1E+06 | 1E+06 | 1E+06 | 988965 | 999714 | 981186 | 1E+06 | 1E+06 | 1E+06 |
| 4 | 177.1 | 160.08 | Serotonin; 5-Hydroxytryptamine* | 2 | 531235 | 594999 | 560803 | 402171 | 447457 | 438226 | 530403 | 529741 | 488024 | 441376 | 439633 | 458952 |
| 5 | 283.07 | 151.03 | Xanthosine | 2 | 2E+07 | 2E+07 | 2E+07 | 1E+07 | 1E+07 | 1E+07 | 2E+07 | 2E+07 | 2E+07 | 2E+07 | 2E+07 | 2E+07 |
| 6 | 328.05 | 134.05 | Cyclic 3',5'-Adenylic acid | 1 | 7E+06 | 6E+06 | 7E+06 | 9E+06 | 9E+06 | 9E+06 | 6E+06 | 6E+06 | 6E+06 | 6E+06 | 7E+06 | 7E+06 |
| 7 | 165.09 | 91.05 | Ethyl phenylacetate | 2 | 192834 | 230867 | 220811 | 341167 | 379668 | 341394 | 228357 | 255807 | 272370 | 261526 | 258899 | 229039 |
| 8 | 179.07 | 92.03 | Propyl 4-hydroxybenzoate | 3 | 65053 | 61080 | 59354 | 58872 | 49249 | 56520 | 159690 | 157643 | 170470 | 97010 | 93965 | 90991 |
| 9 | 179.07 | 146.04 | 4-Methoxyphenylpropionic acid | 3 | 73189 | 85798 | 83030 | 104823 | 115373 | 105644 | 63795 | 64482 | 81879 | 72865 | 79603 | 70155 |
| 10 | 213.08 | 151.04 | Methyl Syringate | 2 | 449028 | 543461 | 394733 | 267685 | 231672 | 220472 | 439860 | 375348 | 437837 | 345623 | 355151 | 353480 |
| 11 | 213.08 | 154.06 | Eudesmic acid (3,4,5-trimethoxybenzoic acid) | 3 | 48542 | 47746 | 52121 | 73406 | 86292 | 99007 | 46780 | 41904 | 33278 | 74804 | 49041 | 53593 |
| 12 | 213.07 | 195.07 | 2,4,5-Trimethoxybenzoic acid | 3 | 93370 | 200495 | 212423 | 34445 | 52111 | 65020 | 239269 | 71690 | 146611 | 77269 | 54644 | 136398 |
| 13 | 252.11 | 136.06 | 2'-Deoxyadenosine* | 3 | 560453 | 614315 | 447072 | 425498 | 456087 | 378743 | 644437 | 607983 | 673512 | 498287 | 533893 | 553944 |
| 14 | 268.11 | 136.06 | Vidarabine | 3 | 7E+06 | 7E+06 | 6E+06 | 6E+06 | 6E+06 | 6E+06 | 8E+06 | 8E+06 | 8E+06 | 7E+06 | 6E+06 | 6E+06 |
| 15 | 268.1 | 136.06 | Adenosine* | 1 | 6E+06 | 6E+06 | 6E+06 | 6E+06 | 6E+06 | 6E+06 | 7E+06 | 8E+06 | 7E+06 | 7E+06 | 6E+06 | 6E+06 |
| 16 | 268.11 | 136.07 | 9-Alpha-Ribofuranosyladenine* | 1 | 7E+06 | 7E+06 | 6E+06 | 7E+06 | 6E+06 | 6E+06 | 8E+06 | 8E+06 | 8E+06 | 7E+06 | 6E+06 | 6E+06 |
| 17 | 196.1 | 91.05 | L-Tyrosine methyl ester | 3 | 455668 | 475155 | 453550 | 502875 | 451520 | 405943 | 314859 | 291245 | 282417 | 400087 | 407919 | 407380 |
| 18 | 239.11 | 181.07 | Proxyphylline | 3 | 5283.3 | 12375 | 27460 | 9 | 9 | 9 | 6670 | 15159 | 21675 | 18836 | 30385 | 19772 |
| 19 | 426.02 | 158.93 | Adenosine 5'-diphosphate | 3 | 2E+06 | 2E+06 | 2E+06 | 2E+06 | 2E+06 | 2E+06 | 2E+06 | 2E+06 | 2E+06 | 2E+06 | 2E+06 | 2E+06 |
| 20 | 197.13 | 70.07 | Cyclo(D-Val-L-Pro) | 3 | 3E+06 | 3E+06 | 3E+06 | 3E+06 | 3E+06 | 4E+06 | 3E+06 | 3E+06 | 3E+06 | 3E+06 | 3E+06 | 3E+06 |
| 21 | 245.1 | 227.08 | Biotin | 3 | 854091 | 1E+06 | 1E+06 | 1E+06 | 1E+06 | 1E+06 | 944119 | 810156 | 862025 | 1E+06 | 1E+06 | 892615 |
| 22 | 277.1 | 148.06 | L-α-Glutamyl-L-Glutamic Acid | 3 | 550504 | 544113 | 502531 | 730473 | 745829 | 670459 | 607987 | 555162 | 560610 | 636197 | 663454 | 602844 |
| 23 | 153.13 | 83.05 | cis-Citral | 3 | 709325 | 703486 | 684819 | 732810 | 731411 | 699226 | 675811 | 640866 | 643639 | 593846 | 594902 | 638545 |
| 24 | 169.12 | 123.12 | Eucamalol | 3 | 2E+06 | 2E+06 | 2E+06 | 2E+06 | 2E+06 | 2E+06 | 2E+06 | 2E+06 | 2E+06 | 2E+06 | 2E+06 | 2E+06 |
| 25 | 247.13 | 184.1 | γ-Glutamyl-L-valine | 3 | 2E+06 | 2E+06 | 2E+06 | 2E+06 | 2E+06 | 2E+06 | 2E+06 | 2E+06 | 2E+06 | 2E+06 | 2E+06 | 2E+06 |
| 26 | 262.14 | 84.08 | Asp-Lys | 2 | 4E+06 | 5E+06 | 6E+06 | 3E+06 | 4E+06 | 4E+06 | 5E+06 | 4E+06 | 5E+06 | 4E+06 | 4E+06 | 4E+06 |
| 27 | 217.15 | 144.08 | Val-Val | 1 | 5E+06 | 4E+06 | 4E+06 | 4E+06 | 4E+06 | 4E+06 | 4E+06 | 4E+06 | 5E+06 | 4E+06 | 4E+06 | 4E+06 |
| 28 | 233.15 | 84.05 | 1',2',3',4'-tetrahydro-5'-deoxypinnatanine | 3 | 6E+06 | 6E+06 | 6E+06 | 6E+06 | 6E+06 | 6E+06 | 6E+06 | 6E+06 | 5E+06 | 5E+06 | 5E+06 | 5E+06 |
| 29 | 187.13 | 141.13 | 10-Hydroxydecanoic acid | 3 | 108048 | 87522 | 92372 | 67582 | 72233 | 64072 | 97544 | 89963 | 99348 | 82786 | 90183 | 80921 |
| 30 | 172.04 | 128.05 | Quinoline-4-carboxylic acid | 2 | 277146 | 288222 | 303215 | 219399 | 279783 | 197708 | 338233 | 323113 | 297954 | 302377 | 234377 | 284983 |
| 31 | 177.05 | 121.06 | 7-Methoxycoumarin | 1 | 2E+07 | 2E+07 | 2E+07 | 1E+07 | 1E+07 | 1E+07 | 1E+07 | 1E+07 | 1E+07 | 1E+07 | 1E+07 | 1E+07 |
| 32 | 193.05 | 153.02 | Noreugenin; 5,7-Dihydroxy-2-Methylchromone | 2 | 1E+06 | 924786 | 999528 | 757740 | 681921 | 689307 | 932250 | 880890 | 837022 | 785764 | 793447 | 766106 |
| 33 | 207.07 | 192.04 | 5,7-Dimethoxycoumarin (Limettin)(Citropten) | 3 | 107648 | 78556 | 343946 | 244375 | 325436 | 104512 | 84025 | 69266 | 102954 | 251838 | 283592 | 261038 |
| 34 | 223.06 | 190.03 | isofraxidin | 3 | 37083 | 39169 | 71174 | 21675 | 11880 | 11259 | 43345 | 46889 | 67720 | 25722 | 23068 | 27752 |
| 35 | 285.03 | 133.02 | L-Malic acid-2-O-gallate | 3 | 752111 | 907808 | 894530 | 2E+06 | 2E+06 | 2E+06 | 2E+06 | 2E+06 | 2E+06 | 1E+06 | 1E+06 | 1E+06 |
| 36 | 190.09 | 118.07 | 3-Indolepropionic acid | 2 | 2E+06 | 2E+06 | 2E+06 | 2E+06 | 2E+06 | 2E+06 | 2E+06 | 2E+06 | 2E+06 | 2E+06 | 2E+06 | 2E+06 |
| 37 | 206.08 | 145.05 | Methoxyindoleacetic acid | 3 | 2E+07 | 2E+07 | 2E+07 | 2E+07 | 2E+07 | 2E+07 | 1E+07 | 1E+07 | 1E+07 | 1E+07 | 2E+07 | 1E+07 |
| 38 | 221.09 | 130.06 | 5-Hydroxy-DL-tryptophan(5-HTP) | 1 | 2E+06 | 2E+06 | 2E+06 | 1E+06 | 1E+06 | 1E+06 | 2E+06 | 2E+06 | 2E+06 | 2E+06 | 2E+06 | 2E+06 |
| 39 | 177.09 | 131.05 | Ethyl cinnamate | 3 | 121384 | 114274 | 152225 | 107780 | 157367 | 162667 | 78921 | 91485 | 71936 | 127228 | 106202 | 137923 |
| 40 | 209.08 | 121.06 | (R)-3-ethyl-7-hydroxy-6-methoxyphthalide | 2 | 4E+06 | 3E+06 | 3E+06 | 4E+06 | 4E+06 | 4E+06 | 4E+06 | 4E+06 | 3E+06 | 4E+06 | 4E+06 | 3E+06 |
| 41 | 204.11 | 159.09 | L-Tryptophanamide | 2 | 204677 | 209157 | 162932 | 194798 | 190201 | 224213 | 142513 | 141181 | 137268 | 159346 | 167306 | 181524 |
| 42 | 223.11 | 120.09 | L-Glycyl-L-phenylalanine* | 2 | 1E+06 | 2E+06 | 1E+06 | 3E+06 | 3E+06 | 3E+06 | 2E+06 | 2E+06 | 2E+06 | 2E+06 | 2E+06 | 2E+06 |
| 43 | 335.06 | 123.06 | β-Nicotinamide mononucleotide | 3 | 340918 | 373868 | 350928 | 581969 | 524590 | 515117 | 412804 | 388674 | 163844 | 497828 | 547285 | 550372 |
| 44 | 298.1 | 136.06 | 5'-Deoxy-5'-(methylthio)adenosine | 1 | 8E+06 | 7E+06 | 7E+06 | 1E+07 | 1E+07 | 1E+07 | 6E+06 | 6E+06 | 6E+06 | 9E+06 | 8E+06 | 8E+06 |
| 45 | 282.12 | 136.06 | 2'-O-Methyladenosine | 3 | 2E+07 | 2E+07 | 2E+07 | 3E+07 | 3E+07 | 3E+07 | 3E+07 | 3E+07 | 3E+07 | 3E+07 | 3E+07 | 3E+07 |
| 46 | 193.13 | 105.03 | N-(4-Aminobutyl)benzamide | 3 | 9 | 9 | 9 | 238543 | 162798 | 174130 | 263464 | 216864 | 251158 | 191307 | 174015 | 302006 |
| 47 | 181.12 | 107.09 | Dihydroactinidiolide | 1 | 2E+07 | 2E+07 | 2E+07 | 1E+07 | 1E+07 | 1E+07 | 2E+07 | 2E+07 | 2E+07 | 1E+07 | 1E+07 | 1E+07 |
| 48 | 181.12 | 121.1 | oxyphyllone F | 3 | 9E+06 | 9E+06 | 9E+06 | 8E+06 | 7E+06 | 7E+06 | 9E+06 | 1E+07 | 1E+07 | 8E+06 | 8E+06 | 8E+06 |
| 49 | 181.12 | 135.12 | 2,5-dimethyl-2,3,3a,7a-tetrahydro-1H-indene-1,3-diol | 3 | 1E+07 | 1E+07 | 1E+07 | 1E+07 | 1E+07 | 1E+07 | 1E+07 | 1E+07 | 1E+07 | 1E+07 | 1E+07 | 1E+07 |
| 50 | 181.12 | 135.12 | 5,6,7,7a-tetrahydro-4,4,7a-trimethyl-2(4H)-benzofuranone | 1 | 1E+07 | 1E+07 | 1E+07 | 1E+07 | 1E+07 | 1E+07 | 1E+07 | 1E+07 | 1E+07 | 1E+07 | 1E+07 | 1E+07 |
| 51 | 197.12 | 133.1 | Loliolide | 1 | 2E+07 | 2E+07 | 2E+07 | 1E+07 | 1E+07 | 2E+07 | 2E+07 | 2E+07 | 2E+07 | 1E+07 | 2E+07 | 2E+07 |
| 52 | 290.09 | 128.04 | N-Fructosyl Pyroglutamate | 1 | 2E+07 | 2E+07 | 2E+07 | 1E+07 | 1E+07 | 1E+07 | 3E+07 | 3E+07 | 2E+07 | 1E+07 | 2E+07 | 2E+07 |
| 53 | 180.14 | 123.07 | Candicine | 3 | 362852 | 473112 | 333485 | 560459 | 453607 | 509756 | 261667 | 295052 | 210461 | 329172 | 341345 | 326632 |
| 54 | 197.12 | 153.12 | Robinlin | 1 | 434301 | 416536 | 438369 | 435023 | 423992 | 408362 | 398229 | 379043 | 396375 | 389520 | 392340 | 390168 |
| 55 | 197.12 | 153.13 | 5-hydroxy-3,4-dimethyl-5-pentylfuran-2(5H)-one | 1 | 487656 | 457887 | 458583 | 477690 | 456791 | 452209 | 420134 | 424035 | 417878 | 443265 | 463016 | 441350 |
| 56 | 197.12 | 153.13 | Hydroxydihydrobovolide | 1 | 455788 | 428838 | 448107 | 451820 | 432356 | 439666 | 403739 | 393430 | 392637 | 426575 | 422441 | 414558 |
| 57 | 322.11 | 130.05 | S-(Methyl)glutathione | 1 | 591974 | 573990 | 591134 | 1E+06 | 1E+06 | 1E+06 | 687847 | 595398 | 663003 | 763221 | 791213 | 737123 |
| 58 | 229.15 | 116.07 | 1-(2-Amino-4-methylpentanoyl)pyrrolidine-2-carboxylic acid | 1 | 8E+06 | 9E+06 | 8E+06 | 1E+07 | 1E+07 | 1E+07 | 9E+06 | 9E+06 | 9E+06 | 9E+06 | 9E+06 | 9E+06 |
| 59 | 275.12 | 145.1 | L-Saccharopine | 3 | 249189 | 233108 | 241459 | 303269 | 276744 | 287159 | 194184 | 240424 | 223623 | 232134 | 261706 | 231588 |
| 60 | 307.11 | 145.06 | L-Glutamine-O-glycoside | 3 | 1E+07 | 1E+07 | 1E+07 | 1E+07 | 1E+07 | 1E+07 | 8E+06 | 8E+06 | 8E+06 | 9E+06 | 1E+07 | 9E+06 |
| 61 | 283.05 | 167.04 | Vnilloylmalic acid | 2 | 1E+06 | 1E+06 | 1E+06 | 1E+06 | 2E+06 | 1E+06 | 1E+06 | 1E+06 | 1E+06 | 1E+06 | 1E+06 | 1E+06 |
| 62 | 250.07 | 88.04 | N-carboxy-N-(2-oxo-2-phenylethyl)-L-alanine | 1 | 2E+06 | 2E+06 | 3E+06 | 2E+06 | 2E+06 | 2E+06 | 3E+06 | 3E+06 | 3E+06 | 2E+06 | 2E+06 | 3E+06 |
| 63 | 219.11 | 160.08 | N-Acetyl-5-hydroxytryptamine | 3 | 728618 | 901760 | 868821 | 796755 | 909344 | 733068 | 581008 | 640671 | 621624 | 835707 | 769506 | 682068 |
| 64 | 237.08 | 163.04 | 1-O-p-Cumaroylglycerol | 3 | 52950 | 36025 | 43151 | 43017 | 37270 | 65378 | 97060 | 102265 | 99014 | 74950 | 83521 | 69024 |
| 65 | 301.06 | 168.01 | Gallic acid-1-O-xyloside | 3 | 9 | 9 | 9 | 123275 | 40411 | 93886 | 268738 | 230179 | 139090 | 120529 | 264590 | 325512 |
| 66 | 286.09 | 124.04 | N-(beta-D-Glucosyl)nicotinate | 3 | 2E+06 | 2E+06 | 2E+06 | 2E+06 | 2E+06 | 2E+06 | 2E+06 | 2E+06 | 2E+06 | 2E+06 | 2E+06 | 2E+06 |
| 67 | 286.09 | 124.04 | Pyridine-4-formyl-O-β-D-glucopyranoside | 1 | 1E+06 | 1E+06 | 1E+06 | 2E+06 | 2E+06 | 2E+06 | 1E+06 | 1E+06 | 1E+06 | 1E+06 | 1E+06 | 1E+06 |
| 68 | 193.12 | 105.04 | Senkyunolide A | 2 | 483409 | 677189 | 726784 | 113297 | 77370 | 109754 | 2E+06 | 2E+06 | 2E+06 | 1E+06 | 912786 | 929924 |
| 69 | 209.12 | 179.07 | alpha-Asarone | 2 | 9 | 9 | 9 | 805092 | 391976 | 939062 | 408950 | 765767 | 708456 | 836438 | 348887 | 525749 |
| 70 | 225.11 | 151.07 | (E)-2,3,4-Trimethoxy-5-(1-propenyl)phenol | 2 | 4E+06 | 3E+06 | 4E+06 | 343697 | 376564 | 355146 | 2E+07 | 2E+07 | 2E+07 | 8E+06 | 8E+06 | 8E+06 |
| 71 | 271.08 | 108.02 | Arbutin | 2 | 552055 | 578517 | 607679 | 576847 | 578539 | 548401 | 532481 | 504388 | 495414 | 512207 | 509847 | 540185 |
| 72 | 287.08 | 125.02 | Phloroglucinol-1-O-β-D-glucopyranoside | 3 | 126246 | 144610 | 105770 | 160480 | 145733 | 173860 | 126321 | 120895 | 103600 | 107391 | 61685 | 141834 |
| 73 | 265.11 | 122.07 | Thiamine (Vitamin B1) | 3 | 56720 | 38815 | 37214 | 97511 | 82713 | 72868 | 33644 | 33051 | 34180 | 46735 | 55891 | 32519 |
| 74 | 272.11 | 110.06 | 3-pyridine-methanol-O-β-D-glucopyranosyl | 3 | 599173 | 647450 | 621403 | 636563 | 605601 | 587884 | 556448 | 502477 | 536396 | 450529 | 497230 | 520821 |
| 75 | 337.08 | 277.06 | 2-O-α-D-Glucopyranosyl-L-ascorbic acid | 1 | 6E+06 | 7E+06 | 7E+06 | 6E+06 | 6E+06 | 6E+06 | 4E+06 | 4E+06 | 4E+06 | 5E+06 | 5E+06 | 5E+06 |
| 76 | 209.12 | 59.02 | Jasmonic acid | 3 | 5E+06 | 5E+06 | 5E+06 | 3E+06 | 3E+06 | 3E+06 | 2E+07 | 1E+07 | 2E+07 | 8E+06 | 7E+06 | 7E+06 |
| 77 | 211.13 | 133.1 | orientalol P | 3 | 2E+06 | 2E+06 | 2E+06 | 1E+06 | 1E+06 | 1E+06 | 6E+06 | 5E+06 | 6E+06 | 3E+06 | 3E+06 | 3E+06 |
| 78 | 213.1485 | 111.0441 | 6-Hydroxy-3,9-Undecadienoic acid Methyl Ester | 3 | 417361 | 385553 | 346237 | 248080 | 192702 | 246953 | 369882 | 574688 | 434202 | 329341 | 339297 | 337657 |
| 79 | 227.13 | 183.14 | glycoric acid | 1 | 1E+07 | 1E+07 | 1E+07 | 1E+07 | 1E+07 | 1E+07 | 1E+07 | 1E+07 | 1E+07 | 1E+07 | 1E+07 | 1E+07 |
| 80 | 304.15 | 185.09 | Nicotianamine | 2 | 4E+06 | 3E+06 | 4E+06 | 7E+06 | 6E+06 | 6E+06 | 5E+06 | 4E+06 | 5E+06 | 5E+06 | 5E+06 | 5E+06 |
| 81 | 325.11 | 59.01 | Rutinose | 3 | 37706 | 41843 | 39113 | 59696 | 49203 | 59739 | 41844 | 46442 | 39974 | 41875 | 43223 | 43256 |
| 82 | 337.17 | 70.07 | N-(1-deoxy-1-fructosyl)arginine | 2 | 4E+06 | 4E+06 | 4E+06 | 3E+06 | 3E+06 | 4E+06 | 4E+06 | 4E+06 | 4E+06 | 3E+06 | 4E+06 | 3E+06 |
| 83 | 247.02 | 191.03 | 3,5,9-Trihydroxy-7,8-dihydrocyclopenta[g]chromene-2,6-dione | 2 | 2E+07 | 2E+07 | 2E+07 | 2E+07 | 2E+07 | 2E+07 | 3E+07 | 3E+07 | 3E+07 | 2E+07 | 2E+07 | 2E+07 |
| 84 | 247.03 | 191.04 | Brevifolin[Geranium] | 2 | 2E+07 | 2E+07 | 2E+07 | 2E+07 | 2E+07 | 2E+07 | 3E+07 | 3E+07 | 3E+07 | 2E+07 | 2E+07 | 2E+07 |
| 85 | 183.08 | 105.03 | Benzophenone | 3 | 520666 | 607476 | 619412 | 611370 | 671007 | 567950 | 475607 | 455596 | 526498 | 547577 | 503482 | 512704 |
| 86 | 295.05 | 179.03 | 2-O-Caffeoylmalic acid | 2 | 2E+07 | 2E+07 | 3E+07 | 2E+07 | 2E+07 | 2E+07 | 3E+07 | 3E+07 | 3E+07 | 2E+07 | 2E+07 | 2E+07 |
| 87 | 311.04 | 179.04 | 2-Caffeoyl-L-tartaric acid (Caftaric acid) | 2 | 2E+06 | 2E+06 | 2E+06 | 1E+06 | 1E+06 | 1E+06 | 2E+06 | 2E+06 | 2E+06 | 1E+06 | 1E+06 | 1E+06 |
| 88 | 214.09 | 153.07 | 3-(2-Naphthyl)-L-alanine | 3 | 46680 | 33340 | 47368 | 152716 | 88659 | 137786 | 72303 | 58196 | 62456 | 53350 | 119122 | 89507 |
| 89 | 245.09 | 203.08 | N-acetyl-tryptophan | 1 | 6E+06 | 6E+06 | 1E+07 | 8E+06 | 5E+06 | 8E+06 | 4E+06 | 4E+06 | 3E+06 | 5E+06 | 5E+06 | 8E+06 |
| 90 | 315.07 | 153.02 | Salicylacyl Glucuronide | 2 | 7E+06 | 5E+06 | 8E+06 | 1E+07 | 1E+07 | 1E+07 | 1E+07 | 1E+07 | 1E+07 | 1E+07 | 1E+07 | 1E+07 |
| 91 | 281.11 | 166.09 | L-Aspartyl-L-Phenylalanine | 2 | 3E+06 | 3E+06 | 3E+06 | 3E+06 | 3E+06 | 3E+06 | 3E+06 | 2E+06 | 3E+06 | 2E+06 | 3E+06 | 2E+06 |
| 92 | 269.1 | 177.05 | 2-Feruloyl-sn-glycerol | 3 | 80432 | 80456 | 110429 | 63941 | 26841 | 31642 | 101552 | 34688 | 53043 | 59216 | 84844 | 72114 |
| 93 | 235.15 | 147.05 | p-Coumaroylputrescine | 3 | 655022 | 558029 | 546445 | 431480 | 395760 | 365095 | 540885 | 574912 | 459330 | 537671 | 521301 | 553983 |
| 94 | 193.16 | 109.06 | Damascenone | 1 | 984510 | 1E+06 | 1E+06 | 932053 | 923909 | 851347 | 4E+07 | 4E+07 | 4E+07 | 1E+07 | 1E+07 | 1E+07 |
| 95 | 193.16 | 109.07 | β-Ionone | 1 | 855955 | 918247 | 890726 | 805382 | 831086 | 755864 | 3E+07 | 3E+07 | 3E+07 | 1E+07 | 1E+07 | 1E+07 |
| 96 | 193.16 | 175.15 | α-Ionone | 1 | 848596 | 909208 | 893592 | 746457 | 807924 | 807579 | 3E+07 | 3E+07 | 3E+07 | 9E+06 | 9E+06 | 9E+06 |
| 97 | 209.15 | 149.1 | (3R,6R,7E)-3-hydroxy-4,7-megastigmadien-9-one | 3 | 2E+06 | 2E+06 | 2E+06 | 956927 | 980196 | 928649 | 2E+06 | 2E+06 | 2E+06 | 2E+06 | 2E+06 | 2E+06 |
| 98 | 209.15 | 149.1 | 9-Hydroxy-5-megastigmen-4-one | 1 | 2E+06 | 2E+06 | 2E+06 | 968778 | 942392 | 890477 | 2E+06 | 2E+06 | 2E+06 | 2E+06 | 2E+06 | 2E+06 |
| 99 | 225.15 | 123.08 | Blumenol A | 2 | 89118 | 129626 | 83802 | 129213 | 124538 | 121026 | 73355 | 78360 | 67220 | 81923 | 161518 | 110885 |
| 100 | 225.15 | 151.11 | Methyl jasmonate | 3 | 704017 | 659887 | 710065 | 55997 | 54830 | 44588 | 4E+06 | 4E+06 | 4E+06 | 2E+06 | 1E+06 | 1E+06 |
| 101 | 291.01 | 247.03 | Phyllanthusiin E | 1 | 5E+06 | 5E+06 | 5E+06 | 6E+06 | 6E+06 | 7E+06 | 9E+06 | 9E+06 | 9E+06 | 6E+06 | 7E+06 | 7E+06 |
| 102 | 321.03 | 125.02 | Digallic Acid | 2 | 4E+07 | 4E+07 | 4E+07 | 4E+07 | 4E+07 | 4E+07 | 5E+07 | 5E+07 | 5E+07 | 4E+07 | 4E+07 | 4E+07 |
| 103 | 307.03 | 227.07 | Resveratrol-3-O-sulfate | 3 | 77047 | 62423 | 55612 | 30454 | 33222 | 46680 | 60276 | 67690 | 65805 | 63030 | 57006 | 49653 |
| 104 | 312.11 | 132.04 | Dhurrin | 2 | 1E+06 | 1E+06 | 728998 | 2E+06 | 2E+06 | 2E+06 | 1E+06 | 1E+06 | 1E+06 | 1E+06 | 1E+06 | 1E+06 |
| 105 | 279.13 | 120.08 | Phe-Hyp | 2 | 330277 | 317112 | 330369 | 310441 | 307531 | 305418 | 311182 | 325256 | 328106 | 295589 | 324379 | 303985 |
| 106 | 295.13 | 120.08 | Glu-Phe | 2 | 6E+06 | 4E+06 | 5E+06 | 8E+06 | 7E+06 | 8E+06 | 5E+06 | 6E+06 | 6E+06 | 6E+06 | 6E+06 | 6E+06 |
| 107 | 295.13 | 120.08 | γ-Glutamylphenylalanine | 2 | 1E+06 | 1E+06 | 1E+06 | 2E+06 | 3E+06 | 2E+06 | 1E+06 | 1E+06 | 1E+06 | 1E+06 | 1E+06 | 2E+06 |
| 108 | 345.08 | 139.04 | 1-O-(3,4-Dihydroxy-5-methoxy-benzoyl)-glucoside | 3 | 2E+06 | 2E+06 | 2E+06 | 1E+06 | 1E+06 | 1E+06 | 747553 | 760493 | 688381 | 1E+06 | 1E+06 | 1E+06 |
| 109 | 425.04 | 241 | methyl 3,5-dihydroxy-4-((3,4,5-trihydroxy-6-((sulfooxy)methyl)tetrahydro-2H-pyran-2-yl)oxy)benzoate | 3 | 1E+06 | 1E+06 | 1E+06 | 754378 | 741021 | 766983 | 1E+06 | 1E+06 | 1E+06 | 929178 | 972473 | 950964 |
| 110 | 425.0394 | 241.0028 | Methylgallic Acid 3-(6''-Sulfate)Glucoside | 3 | 1E+06 | 1E+06 | 2E+06 | 852542 | 746801 | 771249 | 1E+06 | 1E+06 | 1E+06 | 1E+06 | 1E+06 | 1E+06 |
| 111 | 297.1 | 135.07 | Picein (4-Acetylphenyl-glucoside) | 3 | 168066 | 153523 | 135519 | 113324 | 117593 | 95299 | 155442 | 143079 | 136367 | 216136 | 139081 | 151056 |
| 112 | 294.14 | 120.08 | Phe-Gln | 2 | 2E+06 | 2E+06 | 2E+06 | 2E+06 | 2E+06 | 2E+06 | 2E+06 | 2E+06 | 2E+06 | 1E+06 | 2E+06 | 2E+06 |
| 113 | 265.15 | 177.06 | N-Feruloylputrescine | 3 | 1E+06 | 1E+06 | 1E+06 | 842038 | 879896 | 820326 | 1E+06 | 1E+06 | 1E+06 | 914435 | 972969 | 1E+06 |
| 114 | 281.15 | 177.05 | N-Feruloylhydroxyputrescine | 3 | 41378 | 55187 | 80247 | 9 | 9 | 9 | 9 | 9 | 9 | 63056 | 85084 | 82112 |
| 115 | 385.13 | 250 | S-(5'-Adenosy)-L-homocysteine | 3 | 1E+06 | 1E+06 | 1E+06 | 3E+06 | 3E+06 | 3E+06 | 2E+06 | 2E+06 | 2E+06 | 2E+06 | 2E+06 | 2E+06 |
| 116 | 299.11 | 137.02 | Salidroside | 1 | 120847 | 448179 | 461907 | 193638 | 123123 | 252325 | 551186 | 505520 | 602595 | 480153 | 439945 | 482832 |
| 117 | 535.04 | 323.03 | Uridine-5'-Diphosphate-D-Xylose | 3 | 1E+06 | 1E+06 | 1E+06 | 2E+06 | 2E+06 | 2E+06 | 811747 | 797987 | 872427 | 1E+06 | 2E+06 | 1E+06 |
| 118 | 267.17 | 179.07 | Dihydroferuloylputrescine | 3 | 151552 | 166821 | 99972 | 9 | 9 | 9 | 144139 | 107942 | 178043 | 121288 | 193776 | 117236 |
| 119 | 353.15 | 173.08 | Glucosyl 6-methylhept-6-ene-1,2,3,4,5-pentaol | 2 | 924244 | 945023 | 1E+06 | 800796 | 379164 | 719447 | 1E+06 | 1E+06 | 987747 | 870468 | 758232 | 777954 |
| 120 | 337.15 | 179.06 | Dihydroxyoctanoic acid glucoside | 3 | 86628 | 49290 | 48926 | 139508 | 126122 | 139434 | 48044 | 55015 | 86946 | 78499 | 111774 | 98804 |
| 121 | 380.99 | 301.04 | Quercetin-3-O-Sulfonate | 3 | 1E+06 | 2E+06 | 2E+06 | 944040 | 949477 | 923500 | 521766 | 503840 | 530429 | 857703 | 902473 | 921425 |
| 122 | 396.99 | 317.03 | Myricetin-3-O-sulfonate | 3 | 149096 | 131027 | 119725 | 83352 | 62215 | 71526 | 62088 | 48863 | 48448 | 79987 | 97540 | 89643 |
| 123 | 255.07 | 137.02 | 6,7-Dihydroxyflavone | 3 | 12709 | 18550 | 16835 | 10894 | 16886 | 15802 | 41647 | 108641 | 35041 | 33890 | 23287 | 21356 |
| 124 | 271.06 | 215.07 | 4',6,7-Trihydroxyisoflavone | 3 | 1E+06 | 2E+06 | 2E+06 | 3E+06 | 3E+06 | 3E+06 | 2E+06 | 3E+06 | 3E+06 | 2E+06 | 2E+06 | 3E+06 |
| 125 | 287.06 | 153.02 | Thunberginol B | 3 | 7E+07 | 9E+07 | 8E+07 | 3E+07 | 8E+07 | 7E+07 | 2E+07 | 3E+07 | 5E+07 | 7E+07 | 7E+07 | 8E+07 |
| 126 | 301.04 | 149.02 | Hypolaetin* | 2 | 731015 | 795622 | 1E+06 | 599792 | 480678 | 571574 | 830144 | 919419 | 905752 | 772210 | 791124 | 741617 |
| 127 | 301.04 | 149.03 | Isoetin (5,7,2',4',5'-Pentahydroxyflavone)* | 2 | 1E+06 | 1E+06 | 1E+06 | 690279 | 599409 | 731836 | 870476 | 936856 | 913700 | 803175 | 791741 | 863799 |
| 128 | 301.04 | 151 | Tricetin (5,7,3',4',5'-Pentahydroxyflavone) | 2 | 2E+06 | 2E+06 | 2E+06 | 593710 | 372402 | 2E+06 | 2E+06 | 1E+06 | 2E+06 | 787440 | 2E+06 | 2E+06 |
| 129 | 365 | 285.04 | Kaempferol-3-O-sulfonate | 3 | 5E+06 | 6E+06 | 6E+06 | 4E+06 | 3E+06 | 4E+06 | 3E+06 | 2E+06 | 2E+06 | 3E+06 | 4E+06 | 3E+06 |
| 130 | 209.1 | 77.04 | Chalcone | 3 | 160214 | 129898 | 130616 | 88423 | 91449 | 115135 | 130139 | 119253 | 132543 | 153909 | 138587 | 140412 |
| 131 | 255.07 | 119.05 | 3,9-Dihydroxypterocarpan | 3 | 142579 | 142577 | 136901 | 127592 | 121806 | 120577 | 133124 | 132951 | 136664 | 137351 | 124626 | 130622 |
| 132 | 257.08 | 153.02 | Pinocembrin (Dihydrochrysin) | 1 | 1E+06 | 1E+06 | 1E+06 | 2E+06 | 2E+06 | 2E+06 | 1E+06 | 1E+06 | 1E+06 | 1E+06 | 1E+06 | 1E+06 |
| 133 | 273.0763 | 123.0443 | 2-(4-hydroxyphenyl)-2H-chromene-3,5,7-triol | 3 | 3E+07 | 3E+07 | 3E+07 | 3E+07 | 3E+07 | 3E+07 | 4E+07 | 4E+07 | 4E+07 | 3E+07 | 3E+07 | 3E+07 |
| 134 | 273.08 | 153.02 | Naringenin (5,7,4'-Trihydroxyflavanone)* | 1 | 2E+07 | 2E+07 | 2E+07 | 3E+07 | 3E+07 | 3E+07 | 2E+07 | 2E+07 | 2E+07 | 2E+07 | 3E+07 | 2E+07 |
| 135 | 273.08 | 153.02 | Naringenin chalcone; 2',4,4',6'-Tetrahydroxychalcone | 1 | 2E+07 | 2E+07 | 2E+07 | 3E+07 | 3E+07 | 3E+07 | 3E+07 | 2E+07 | 3E+07 | 2E+07 | 2E+07 | 2E+07 |
| 136 | 287.06 | 125.03 | 2-hydroxynaringenin | 2 | 2E+06 | 2E+06 | 2E+06 | 4E+06 | 4E+06 | 4E+06 | 3E+06 | 5E+06 | 2E+06 | 3E+06 | 3E+06 | 3E+06 |
| 137 | 287.0561 | 135.0452 | Eriodictyol (5,7,3',4'-Tetrahydroxyflavanone) | 3 | 6E+06 | 6E+06 | 6E+06 | 4E+06 | 4E+06 | 4E+06 | 5E+06 | 5E+06 | 5E+06 | 4E+06 | 5E+06 | 5E+06 |
| 138 | 287.06 | 149.02 | Fustin | 3 | 204185 | 240505 | 246777 | 174886 | 187587 | 145062 | 276764 | 295476 | 220206 | 193530 | 377193 | 327713 |
| 139 | 289.07 | 153.02 | Okanin | 1 | 4E+06 | 4E+06 | 4E+06 | 3E+06 | 3E+06 | 3E+06 | 3E+06 | 4E+06 | 3E+06 | 3E+06 | 3E+06 | 3E+06 |
| 140 | 319.05 | 193.01 | Dihydromyricetin (Ampelopsin) | 2 | 580632 | 598644 | 535344 | 343174 | 276161 | 270311 | 519980 | 453140 | 446055 | 471161 | 437114 | 442365 |
| 141 | 275.09 | 107.05 | Epiafzelechin | 1 | 4E+06 | 6E+06 | 4E+06 | 1E+07 | 1E+07 | 8E+06 | 7E+06 | 7E+06 | 4E+06 | 8E+06 | 5E+06 | 8E+06 |
| 142 | 275.09 | 139.04 | Afzelechin (3,5,7,4'-Tetrahydroxyflavan) | 2 | 338523 | 398895 | 268732 | 470835 | 469949 | 482741 | 289983 | 339326 | 234236 | 482490 | 296804 | 237940 |
| 143 | 273.08 | 167.03 | Phloretin | 3 | 255529 | 266637 | 252396 | 254092 | 247694 | 237375 | 185018 | 180385 | 169486 | 237008 | 206062 | 228450 |
| 144 | 291.09 | 123.04 | Leucopelargonidin; 3,4,5,7,4'-Pentahydroxyflavan | 1 | 4E+06 | 4E+06 | 4E+06 | 3E+06 | 3E+06 | 4E+06 | 5E+06 | 5E+06 | 5E+06 | 4E+06 | 4E+06 | 4E+06 |
| 145 | 289.0718 | 151.0401 | 5,7,3',4',5'-Pentahydroxyflavan (Tricetiflavan) | 3 | 5E+06 | 5E+06 | 4E+06 | 4E+06 | 4E+06 | 4E+06 | 4E+06 | 5E+06 | 5E+06 | 4E+06 | 4E+06 | 4E+06 |
| 146 | 305.07 | 125.02 | Leucocyanidin | 1 | 3E+06 | 3E+06 | 3E+06 | 1E+06 | 1E+06 | 1E+06 | 1E+06 | 1E+06 | 1E+06 | 2E+06 | 2E+06 | 2E+06 |
| 147 | 307.08 | 139.04 | Gallocatechin | 2 | 4E+06 | 4E+06 | 4E+06 | 2E+06 | 2E+06 | 2E+06 | 2E+06 | 2E+06 | 2E+06 | 2E+06 | 3E+06 | 3E+06 |
| 148 | 309.1 | 147.04 | 1-O-(p-coumaroyl) 3-Hydroxy-3-methylglutaric acid | 1 | 6E+07 | 8E+07 | 1E+08 | 5E+07 | 6E+07 | 7E+07 | 6E+07 | 3E+07 | 3E+07 | 4E+07 | 5E+07 | 5E+07 |
| 149 | 325.09 | 163.04 | Skimmin (7-Hydroxycoumarin-7-O-glucoside) | 2 | 212159 | 221729 | 164947 | 183811 | 194464 | 188617 | 257238 | 231778 | 236451 | 172076 | 215948 | 200353 |
| 150 | 339.0722 | 177.0195 | Esculin (6,7-Dihydroxycoumarin-6-O-glucoside)* | 3 | 394932 | 427396 | 399106 | 224581 | 254964 | 202470 | 352611 | 324965 | 342035 | 396232 | 354472 | 373905 |
| 151 | 339.07 | 177.02 | Esculetin-7-O-glucoside* | 3 | 414756 | 347414 | 437057 | 262674 | 284055 | 230333 | 415142 | 408895 | 410628 | 394069 | 323572 | 341050 |
| 152 | 339.07 | 177.02 | Daphnin* | 3 | 427484 | 362245 | 355104 | 261360 | 262071 | 243154 | 378862 | 336215 | 390066 | 371556 | 376075 | 367954 |
| 153 | 341.09 | 207.07 | Sinapoyl malate | 3 | 383564 | 352704 | 309730 | 279573 | 274343 | 274977 | 387473 | 345594 | 419742 | 270187 | 320722 | 361396 |
| 154 | 357.08 | 173.04 | 5-O-Galloyl-methyl quinine ester | 2 | 1E+06 | 1E+06 | 906118 | 909702 | 887244 | 718213 | 249671 | 431003 | 464898 | 733444 | 723069 | 775495 |
| 155 | 247.1324 | 131.0848 | Zaluzanin C | 3 | 3E+06 | 3E+06 | 3E+06 | 3E+06 | 3E+06 | 3E+06 | 2E+06 | 2E+06 | 2E+06 | 3E+06 | 2E+06 | 3E+06 |
| 156 | 247.1338 | 145.0997 | Dehydrosantamarin | 3 | 4E+06 | 4E+06 | 4E+06 | 4E+06 | 4E+06 | 4E+06 | 3E+06 | 3E+06 | 3E+06 | 4E+06 | 3E+06 | 3E+06 |
| 157 | 247.13 | 187.11 | Leucodin | 3 | 1E+07 | 1E+07 | 2E+07 | 1E+07 | 1E+07 | 1E+07 | 1E+07 | 1E+07 | 1E+07 | 1E+07 | 1E+07 | 1E+07 |
| 158 | 247.133 | 187.1108 | Mansonone S | 3 | 2E+07 | 2E+07 | 2E+07 | 2E+07 | 2E+07 | 1E+07 | 1E+07 | 1E+07 | 1E+07 | 1E+07 | 1E+07 | 1E+07 |
| 159 | 325.09 | 119.05 | Phenylpropionic acid-O-β-D-glucopyranoside | 1 | 3E+06 | 3E+06 | 4E+06 | 3E+06 | 3E+06 | 3E+06 | 2E+06 | 2E+06 | 2E+06 | 2E+06 | 3E+06 | 3E+06 |
| 160 | 325.09 | 145.03 | 1-O-p-Coumaroyl-β-D-glucose* | 2 | 358738 | 521924 | 311828 | 484656 | 505133 | 186833 | 177539 | 146801 | 211785 | 215142 | 217341 | 226118 |
| 161 | 325.09 | 163.04 | p-Coumaric acid-4-O-glucoside | 1 | 3E+06 | 4E+06 | 5E+06 | 4E+06 | 4E+06 | 3E+06 | 2E+06 | 2E+06 | 2E+06 | 3E+06 | 3E+06 | 3E+06 |
| 162 | 341.09 | 163.04 | 1'-O-Galactoyl p-Coumaric acid* | 1 | 5E+06 | 5E+06 | 5E+06 | 5E+06 | 5E+06 | 5E+06 | 3E+06 | 3E+06 | 3E+06 | 4E+06 | 4E+06 | 4E+06 |
| 163 | 341.09 | 163.04 | 4-O-Galactoyl p-Coumaric acid* | 1 | 4E+06 | 5E+06 | 5E+06 | 5E+06 | 4E+06 | 5E+06 | 3E+06 | 3E+06 | 3E+06 | 4E+06 | 4E+06 | 4E+06 |
| 164 | 341.09 | 179.03 | 6-O-Caffeoyl-D-glucose* | 2 | 990760 | 974656 | 939549 | 1E+06 | 1E+06 | 2E+06 | 853898 | 878668 | 930720 | 1E+06 | 1E+06 | 1E+06 |
| 165 | 359.1 | 182.02 | Glucosyringic Acid | 3 | 476058 | 466972 | 400423 | 306275 | 323271 | 318927 | 418725 | 412864 | 419708 | 391641 | 387417 | 369843 |
| 166 | 439.05 | 241.01 | 3,5-dimethoxy-4-((3,4,5-trihydroxy-6-((sulfooxy)methyl)tetrahydro-2H-pyran-2-yl)oxy)benzoic acid | 3 | 1E+07 | 1E+07 | 1E+07 | 8E+06 | 9E+06 | 9E+06 | 1E+07 | 1E+07 | 1E+07 | 1E+07 | 9E+06 | 1E+07 |
| 167 | 249.15 | 145.1 | Santamarin | 2 | 16233 | 11023 | 10642 | 15090 | 41417 | 11202 | 106199 | 286750 | 235589 | 95086 | 52690 | 77079 |
| 168 | 263.13 | 204.12 | Abscisic acid | 1 | 7E+06 | 7E+06 | 7E+06 | 7E+06 | 7E+06 | 7E+06 | 6E+06 | 6E+06 | 6E+06 | 6E+06 | 6E+06 | 7E+06 |
| 169 | 327.11 | 165.05 | Demethyl coniferin | 3 | 2E+06 | 2E+06 | 2E+06 | 2E+06 | 2E+06 | 2E+06 | 2E+06 | 2E+06 | 2E+06 | 2E+06 | 2E+06 | 2E+06 |
| 170 | 400.15 | 136.06 | Ribosyladenosine | 3 | 2E+06 | 2E+06 | 2E+06 | 2E+06 | 2E+06 | 2E+06 | 2E+06 | 2E+06 | 2E+06 | 2E+06 | 2E+06 | 2E+06 |
| 171 | 399.14 | 250.09 | S-(5'-Adenosyl)-L-methionine | 3 | 61690 | 88610 | 76634 | 291569 | 312902 | 306308 | 106875 | 97321 | 99459 | 120121 | 136539 | 117921 |
| 172 | 235.17 | 133.1 | Procurcumenol | 2 | 53983 | 232586 | 53981 | 386283 | 231708 | 226386 | 181740 | 172738 | 252803 | 354421 | 230136 | 155088 |
| 173 | 235.17 | 179.11 | polygodial | 3 | 5E+06 | 5E+06 | 5E+06 | 5E+06 | 4E+06 | 4E+06 | 4E+06 | 4E+06 | 4E+06 | 4E+06 | 4E+06 | 4E+06 |
| 174 | 265.14 | 125.1 | 1β,3β-dihydroxy-eudesmane-5,11(13)-dien-12-oic acid | 2 | 244041 | 244721 | 249257 | 965978 | 933158 | 879935 | 967730 | 910332 | 1E+06 | 670107 | 680056 | 662556 |
| 175 | 281.14 | 124.02 | Octyl gallate | 2 | 421122 | 461891 | 436486 | 441313 | 471998 | 448678 | 412879 | 402984 | 389465 | 425933 | 422974 | 427911 |
| 176 | 345.12 | 183.07 | Aucubin | 2 | 3E+06 | 3E+06 | 3E+06 | 3E+06 | 3E+06 | 3E+06 | 3E+06 | 4E+06 | 4E+06 | 4E+06 | 3E+06 | 3E+06 |
| 177 | 399.14 | 250.09 | S-Adenosylmethionine | 3 | 61690 | 88610 | 76634 | 291569 | 312902 | 306308 | 106875 | 97321 | 99459 | 120121 | 136539 | 117921 |
| 178 | 221.19 | 119.09 | Spathulenol | 1 | 593210 | 621062 | 612831 | 657024 | 623691 | 596286 | 579291 | 577575 | 559844 | 579336 | 584890 | 590102 |
| 179 | 408.99 | 329.03 | 3,4-Dimethylellagic acid 4'-sulfate | 3 | 644995 | 672601 | 704798 | 314389 | 325251 | 288778 | 469335 | 485833 | 1E+06 | 481691 | 391785 | 443168 |
| 180 | 327.04 | 171.06 | Orange I | 2 | 4E+06 | 4E+06 | 4E+06 | 3E+06 | 3E+06 | 3E+06 | 4E+06 | 4E+06 | 4E+06 | 4E+06 | 4E+06 | 4E+06 |
| 181 | 237.09 | 135.05 | 6-Methylflavone | 3 | 46375 | 50190 | 45686 | 63875 | 65070 | 68690 | 21547 | 46642 | 32800 | 56203 | 59473 | 74252 |
| 182 | 285.08 | 270.05 | Calycosin | 3 | 86654 | 87427 | 66685 | 63908 | 65370 | 40877 | 97928 | 162739 | 87622 | 88448 | 81549 | 86622 |
| 183 | 294.13 | 128.07 | 1-methyl-9-[2-(1,2,4-triazol-1-yl)ethyl]-2H-pyrido[3,4-b]indol-7-one | 2 | 133961 | 155047 | 181094 | 340142 | 257124 | 294462 | 133549 | 153792 | 106944 | 167597 | 171627 | 75808 |
| 184 | 335.08 | 135.05 | 5-O-Caffeoylshikimic acid | 3 | 153205 | 124334 | 135411 | 90433 | 122875 | 87071 | 167074 | 128731 | 141978 | 115768 | 160085 | 136665 |
| 185 | 335.08 | 179.03 | 4-caffeoylshikimic acid | 3 | 125455 | 127991 | 124835 | 125431 | 136095 | 123512 | 196818 | 174317 | 172387 | 143273 | 130298 | 149211 |
| 186 | 337.09 | 163.04 | 1-O-p-Coumaroylquinic acid | 1 | 2E+07 | 2E+07 | 2E+07 | 6E+06 | 1E+07 | 1E+07 | 1E+07 | 1E+07 | 1E+07 | 1E+07 | 1E+07 | 1E+07 |
| 187 | 337.09 | 163.04 | 4-O-p-Coumaroylquinic acid | 3 | 5E+07 | 4E+07 | 4E+07 | 3E+07 | 3E+07 | 3E+07 | 4E+07 | 4E+07 | 4E+07 | 3E+07 | 4E+07 | 4E+07 |
| 188 | 353.09 | 191.06 | 1-Caffeoylquinic acid | 1 | 6E+06 | 7E+06 | 7E+06 | 4E+06 | 4E+06 | 4E+06 | 7E+06 | 7E+06 | 7E+06 | 5E+06 | 5E+06 | 5E+06 |
| 189 | 353.09 | 191.06 | Cryptochlorogenic acid (4-O-Caffeoylquinic acid)* | 1 | 9E+06 | 9E+06 | 9E+06 | 8E+06 | 9E+06 | 8E+06 | 1E+07 | 1E+07 | 1E+07 | 9E+06 | 8E+06 | 1E+07 |
| 190 | 355.1 | 121.03 | Homovanilloylquinic acid | 3 | 3E+06 | 4E+06 | 3E+06 | 1E+06 | 1E+06 | 1E+06 | 5E+06 | 4E+06 | 4E+06 | 3E+06 | 3E+06 | 3E+06 |
| 191 | 341.12 | 179.07 | Coniferin | 2 | 889367 | 788219 | 810379 | 679268 | 565733 | 538050 | 773527 | 762990 | 757255 | 659400 | 711796 | 738556 |
| 192 | 308.2 | 163.04 | Caffeoylspermidine | 3 | 506444 | 575083 | 538694 | 1E+06 | 1E+06 | 1E+06 | 685877 | 553399 | 648833 | 627212 | 653246 | 613351 |
| 193 | 235.21 | 81.07 | Callicarpenal | 3 | 716017 | 712338 | 736604 | 460734 | 428570 | 484020 | 680190 | 712791 | 611697 | 568147 | 572436 | 570938 |
| 194 | 358.2 | 116.07 | Glu-Pro-Leu | 2 | 327662 | 322622 | 216685 | 508289 | 566935 | 512007 | 395811 | 280257 | 171771 | 390058 | 331112 | 338512 |
| 195 | 253.22 | 81.07 | dalodorin B | 3 | 590268 | 579576 | 645281 | 411004 | 389217 | 433950 | 502459 | 615786 | 586636 | 550669 | 486986 | 509204 |
| 196 | 333.19 | 135.12 | 2,6-Dimethyl-6-hydroxy-2,7-octadienyl-β-D-glucoside (Betulalbuside A; Betulabuside A) | 2 | 8E+06 | 8E+06 | 9E+06 | 1E+07 | 1E+07 | 9E+06 | 7E+06 | 8E+06 | 9E+06 | 7E+06 | 8E+06 | 8E+06 |
| 197 | 333.19 | 153.13 | (3R,4R)-p-Menth-1-ene-3,4-diol-3-O-β-D-glucoside* | 1 | 9E+06 | 9E+06 | 7E+06 | 1E+07 | 8E+06 | 9E+06 | 7E+06 | 7E+06 | 6E+06 | 9E+06 | 7E+06 | 7E+06 |
| 198 | 333.19 | 153.13 | (3R,4S,6R)-p-Menth-1-ene-3,6-diol-3-O-β-D-glucoside* | 1 | 1E+07 | 1E+07 | 8E+06 | 1E+07 | 1E+07 | 1E+07 | 9E+06 | 7E+06 | 8E+06 | 9E+06 | 8E+06 | 9E+06 |
| 199 | 253.22 | 235.21 | (7Z)-Hexadecenoic acid* | 1 | 104966 | 94568 | 102911 | 86704 | 83108 | 85140 | 120225 | 112344 | 102685 | 95056 | 94131 | 86378 |
| 200 | 287.22 | 241.22 | 10,16-Dihydroxypalmitic acid | 1 | 501837 | 529209 | 547970 | 336095 | 350072 | 326173 | 514796 | 519299 | 526209 | 463957 | 454370 | 456545 |
| 201 | 329.06 | 299.02 | (2Z)-4,6-Dihydroxy-2-[(4-Hydroxy-3,5-Dimethoxyphenyl)Methylidene]-1-Benzofuran-3-One | 3 | 5E+06 | 5E+06 | 5E+06 | 3E+06 | 3E+06 | 3E+06 | 4E+06 | 4E+06 | 4E+06 | 4E+06 | 4E+06 | 4E+06 |
| 202 | 331.08 | 316.05 | Iristectorigenin A | 3 | 6E+06 | 6E+06 | 6E+06 | 3E+06 | 3E+06 | 3E+06 | 5E+06 | 5E+06 | 5E+06 | 4E+06 | 4E+06 | 5E+06 |
| 203 | 331.0821 | 316.0899 | Demethoxysudachitin | 3 | 6E+06 | 6E+06 | 6E+06 | 3E+06 | 3E+06 | 3E+06 | 5E+06 | 5E+06 | 5E+06 | 4E+06 | 4E+06 | 5E+06 |
| 204 | 298.11 | 136.06 | (E)-3-(3',5'-Dimethoxy-4'-hydroxy-benzylidene')-2-indolinone | 2 | 459090 | 486124 | 497697 | 451337 | 394404 | 416508 | 518707 | 537764 | 533641 | 414240 | 422885 | 462469 |
| 205 | 283.1 | 135.04 | Phenethyl caffeate | 2 | 51024 | 55361 | 54742 | 53457 | 57717 | 52420 | 70101 | 70038 | 71639 | 61102 | 59255 | 60631 |
| 206 | 287.14 | 121.03 | 4'-Hydroxy-3,5-dimethoxydihydrochalcone | 2 | 2E+06 | 2E+06 | 2E+06 | 2E+06 | 2E+06 | 2E+06 | 2E+06 | 2E+06 | 2E+06 | 2E+06 | 2E+06 | 2E+06 |
| 207 | 367.1035 | 193.0506 | 3-O-Feruloylquinic acid | 3 | 2E+06 | 2E+06 | 2E+06 | 1E+06 | 1E+06 | 1E+06 | 2E+06 | 2E+06 | 2E+06 | 2E+06 | 2E+06 | 2E+06 |
| 208 | 365.13 | 203.08 | N-(1-Deoxy-1-fructosyl)Tryptophan | 1 | 2E+06 | 2E+06 | 2E+06 | 2E+06 | 2E+06 | 1E+06 | 2E+06 | 2E+06 | 2E+06 | 2E+06 | 2E+06 | 2E+06 |
| 209 | 387.13 | 147.04 | 10-Dehydrogeniposide | 3 | 64684 | 38345 | 79247 | 44652 | 51789 | 23815 | 196982 | 123390 | 74211 | 47867 | 81695 | 57205 |
| 210 | 385.11 | 151.1 | 1-O-Eudesmoylquinic acid | 3 | 320653 | 325887 | 404858 | 360249 | 311731 | 275688 | 613671 | 474467 | 562111 | 344012 | 365933 | 478373 |
| 211 | 277.18 | 137.06 | Lycoposerramine E | 3 | 2E+06 | 3E+06 | 2E+06 | 3E+06 | 3E+06 | 3E+06 | 3E+06 | 3E+06 | 3E+06 | 2E+06 | 2E+06 | 2E+06 |
| 212 | 371.13 | 209.08 | Syringin | 3 | 4E+06 | 4E+06 | 2E+06 | 4E+06 | 7E+06 | 8E+06 | 2E+06 | 2E+06 | 3E+06 | 4E+06 | 5E+06 | 4E+06 |
| 213 | 263.2 | 59.01 | Norlinolenic acid | 3 | 45610 | 47021 | 45302 | 33675 | 38007 | 39306 | 54195 | 50573 | 54033 | 48611 | 45691 | 45007 |
| 214 | 325.07 | 193.05 | 2-(7-Dihydroxyl)-benzofuranyl-ferulic acid | 3 | 1E+06 | 1E+06 | 1E+06 | 1E+06 | 1E+06 | 1E+06 | 2E+06 | 2E+06 | 2E+06 | 1E+06 | 1E+06 | 1E+06 |
| 215 | 329.1 | 299.05 | 5-Hydroxy-7,3',4'-trimethoxyflavone | 3 | 74370 | 46981 | 41996 | 14868 | 19472 | 18693 | 44441 | 39894 | 42407 | 41525 | 41209 | 39267 |
| 216 | 345.1 | 284.07 | 5,7-Dihydroxy-3',4',5'-trimethoxyflavone | 3 | 90878 | 84674 | 81236 | 25394 | 23221 | 36995 | 54379 | 52363 | 62568 | 49972 | 48206 | 57953 |
| 217 | 359.08 | 161.02 | Rosmarinic acid | 2 | 2E+07 | 2E+07 | 4E+07 | 41896 | 30135 | 20465 | 5E+06 | 1E+06 | 4E+06 | 1E+07 | 1E+07 | 1E+07 |
| 218 | 359.08 | 161.02 | Myricetin-3,7,3'-trimethyl ether | 2 | 2E+07 | 2E+07 | 2E+07 | 44341 | 35990 | 10005 | 5E+06 | 1E+06 | 2E+06 | 2E+07 | 1E+07 | 1E+07 |
| 219 | 359.08 | 197.05 | Salvianic acid B | 2 | 5E+06 | 4E+06 | 9E+06 | 34583 | 31600 | 23554 | 1E+06 | 297467 | 743373 | 3E+06 | 3E+06 | 3E+06 |
| 220 | 315.12 | 181.05 | 2'-Hydroxy-4,4',6'-Trimethoxychalcone; Flavokawain A | 1 | 147669 | 125626 | 182226 | 8288.4 | 11425 | 10110 | 5E+06 | 6E+06 | 5E+06 | 2E+06 | 2E+06 | 2E+06 |
| 221 | 301.14 | 245.09 | 5-O-Methyllatifolin | 1 | 87765 | 82772 | 96553 | 45433 | 30576 | 49177 | 38124 | 26548 | 29710 | 70052 | 77442 | 43746 |
| 222 | 447.12 | 152.01 | Protocatechuic acid glucosyl xyloside | 1 | 915609 | 975341 | 934415 | 921120 | 982552 | 927582 | 1E+06 | 1E+06 | 1E+06 | 887211 | 917983 | 948464 |
| 223 | 368.17 | 131.05 | N-(2-hydroxyethyl)-N-methyl-3-phenylacrylamideglucoside | 3 | 92694 | 263580 | 171466 | 73594 | 56888 | 64901 | 115031 | 128580 | 102883 | 84023 | 66779 | 55940 |
| 224 | 275.2 | 105.07 | 3-(Cyclohexen-1-yl)-2-hydroxy-4a,5-dimethyl-2,3,4,5,6,8a-hexahydronaphthalen-1-one | 3 | 3E+06 | 3E+06 | 3E+06 | 3E+06 | 3E+06 | 3E+06 | 3E+06 | 3E+06 | 3E+06 | 3E+06 | 3E+06 | 3E+06 |
| 225 | 275.2 | 119.08 | 3β,12-dihydroxy-13-methyl-podocarpane-8,10,13-tiene | 3 | 2E+06 | 2E+06 | 2E+06 | 3E+06 | 4E+06 | 4E+06 | 2E+06 | 2E+06 | 3E+06 | 3E+06 | 3E+06 | 3E+06 |
| 226 | 275.2 | 133.1 | (10Z,14E,16E)-10,14,16-Octadecatrien-12-Ynoic Acid | 2 | 1E+06 | 1E+06 | 1E+06 | 2E+06 | 2E+06 | 2E+06 | 1E+06 | 970381 | 1E+06 | 1E+06 | 1E+06 | 1E+06 |
| 227 | 275.2 | 149.1 | 3-(4-hydroxycyclohex-1-en-1-yl)-4a,5-dimethyl-3,4,4a,5,6,8a-hexahydronaphthalen-1(2H)-one | 3 | 1E+06 | 2E+06 | 2E+06 | 2E+06 | 2E+06 | 2E+06 | 1E+06 | 1E+06 | 1E+06 | 1E+06 | 2E+06 | 2E+06 |
| 228 | 291.19 | 151.07 | Normelanothyrsin A | 3 | 390612 | 486888 | 398241 | 729342 | 698808 | 607621 | 412093 | 508299 | 377501 | 456486 | 414313 | 466271 |
| 229 | 277.22 | 93.07 | 14,15-Dehydrocrepenynic acid | 3 | 3E+07 | 7E+06 | 8E+06 | 4E+07 | 4E+07 | 3E+07 | 2E+07 | 2E+07 | 8E+06 | 8E+06 | 8E+06 | 9E+06 |
| 230 | 277.22 | 93.07 | 3-(Cyclohexen-1-yl)-2-hydroxy-4a,5-dimethyl-2,3,4,5,6,7,8,8a-octahydronaphthalen-1-one | 3 | 1E+07 | 1E+07 | 1E+07 | 2E+07 | 2E+07 | 2E+07 | 1E+07 | 1E+07 | 1E+07 | 1E+07 | 1E+07 | 1E+07 |
| 231 | 277.22 | 93.07 | Macrophypene B | 3 | 1E+07 | 1E+07 | 1E+07 | 2E+07 | 2E+07 | 2E+07 | 1E+07 | 1E+07 | 1E+07 | 1E+07 | 1E+07 | 1E+07 |
| 232 | 277.216 | 93.0701 | Moroctic Acid | 3 | 1E+07 | 1E+07 | 1E+07 | 2E+07 | 2E+07 | 2E+07 | 1E+07 | 1E+07 | 1E+07 | 1E+07 | 1E+07 | 1E+07 |
| 233 | 277.22 | 235.18 | 15,16-Bisnor-13-oxo-8(17),11-labdadien-19-ol | 1 | 644760 | 751944 | 703988 | 1E+06 | 1E+06 | 1E+06 | 807205 | 771829 | 770804 | 868540 | 849899 | 851008 |
| 234 | 293.21 | 55.05 | 2-Methoxy-6-undecyl-1,4-benzoquinone | 1 | 1E+06 | 1E+06 | 1E+06 | 1E+06 | 1E+06 | 1E+06 | 951864 | 961646 | 980448 | 988608 | 1E+06 | 1E+06 |
| 235 | 293.21 | 149.1 | JiangxiBaiyingsu I | 1 | 4E+06 | 4E+06 | 4E+06 | 5E+06 | 5E+06 | 4E+06 | 3E+06 | 3E+06 | 3E+06 | 3E+06 | 3E+06 | 4E+06 |
| 236 | 387.17 | 207.11 | Tuberonic acid glucoside | 3 | 1E+06 | 1E+06 | 1E+06 | 1E+06 | 2E+06 | 1E+06 | 2E+06 | 2E+06 | 2E+06 | 1E+06 | 1E+06 | 2E+06 |
| 237 | 263.24 | 81.07 | Octadeca-2,9,12,15-tetraen-1-ol | 1 | 3E+06 | 3E+06 | 3E+06 | 3E+06 | 3E+06 | 3E+06 | 4E+06 | 4E+06 | 4E+06 | 3E+06 | 3E+06 | 3E+06 |
| 238 | 277.2173 | 59.0139 | Crepenynic acid | 3 | 3E+06 | 3E+06 | 3E+06 | 2E+06 | 2E+06 | 2E+06 | 4E+06 | 3E+06 | 3E+06 | 3E+06 | 3E+06 | 3E+06 |
| 239 | 279.23 | 81.07 | Elaidolinolenic acid | 1 | 739665 | 604328 | 602950 | 765296 | 724421 | 755988 | 591172 | 545776 | 557016 | 697143 | 639155 | 581589 |
| 240 | 279.23 | 95.09 | Punicic acid (9Z,11E,13Z-octadecatrienoic acid) | 3 | 925303 | 836706 | 860328 | 1E+06 | 1E+06 | 1E+06 | 842170 | 793830 | 734745 | 970973 | 894616 | 811636 |
| 241 | 277.22 | 277.22 | 4-[3-(4,8-dimethylnona-3,7-dienyl)-3-methyloxiran-2-yl]butan-2-one | 1 | 9E+07 | 9E+07 | 9E+07 | 8E+07 | 8E+07 | 8E+07 | 1E+08 | 1E+08 | 1E+08 | 9E+07 | 9E+07 | 9E+07 |
| 242 | 277.22 | 277.22 | α-Linolenic Acid* | 1 | 9E+07 | 9E+07 | 9E+07 | 8E+07 | 8E+07 | 8E+07 | 1E+08 | 1E+08 | 1E+08 | 9E+07 | 9E+07 | 9E+07 |
| 243 | 277.22 | 277.22 | γ-Linolenic Acid* | 1 | 9E+07 | 9E+07 | 9E+07 | 8E+07 | 8E+07 | 8E+07 | 1E+08 | 1E+08 | 1E+08 | 9E+07 | 9E+07 | 9E+07 |
| 244 | 293.21 | 113.1 | 17-Hydroxylinolenic acid | 2 | 3E+07 | 3E+07 | 3E+07 | 3E+07 | 3E+07 | 3E+07 | 3E+07 | 3E+07 | 3E+07 | 3E+07 | 3E+07 | 3E+07 |
| 245 | 295.23 | 151.11 | 3-Hydroxy-3,7,11-trimethyldodeca-1,6E,10-trien-9-yl isobutyrate | 1 | 5E+06 | 5E+06 | 5E+06 | 6E+06 | 6E+06 | 6E+06 | 5E+06 | 5E+06 | 5E+06 | 5E+06 | 5E+06 | 5E+06 |
| 246 | 293.21 | 235.17 | 9-Hydroxy-10,12,15-octadecatrienoic acid* | 3 | 263762 | 262949 | 280457 | 328236 | 313071 | 309868 | 275056 | 252648 | 285158 | 287219 | 276035 | 286677 |
| 247 | 293.21 | 293.21 | Machilusolide D | 3 | 2E+07 | 2E+07 | 3E+07 | 3E+07 | 3E+07 | 3E+07 | 2E+07 | 2E+07 | 2E+07 | 3E+07 | 2E+07 | 2E+07 |
| 248 | 309.21 | 171.1 | 9-Hydroxy-12-oxo-10(E),15(Z)-octadecadienoic acid | 2 | 914790 | 911600 | 981131 | 1E+06 | 1E+06 | 1E+06 | 1E+06 | 962313 | 984549 | 1E+06 | 1E+06 | 1E+06 |
| 249 | 309.2 | 227.2 | 13(s)-hydroperoxy-(9z,11e,15z)-octadecatrienoic acid | 1 | 3E+06 | 3E+06 | 3E+06 | 3E+06 | 3E+06 | 3E+06 | 3E+06 | 2E+06 | 3E+06 | 3E+06 | 2E+06 | 3E+06 |
| 250 | 373.19 | 331.17 | p-Menth-4(5)-ene-1,2-diol-1-O-β-D-(2-O-acetyl)-glucoside | 1 | 40010 | 25787 | 36777 | 12552 | 24062 | 29200 | 46641 | 40010 | 58409 | 44037 | 28821 | 25158 |
| 251 | 487.17 | 341.11 | Solatriose | 3 | 117384 | 131116 | 113203 | 342080 | 165787 | 197907 | 118797 | 115841 | 113624 | 106517 | 132445 | 113134 |
| 252 | 279.23 | 59.01 | Linoleic acid* | 1 | 624048 | 574029 | 598046 | 539790 | 553267 | 541948 | 648628 | 637268 | 624217 | 598149 | 595676 | 573559 |
| 253 | 279.233 | 59.0133 | (9Z,11E)-Octadecadienoic acid* | 1 | 624048 | 574029 | 598046 | 539790 | 553267 | 541948 | 648628 | 637268 | 624217 | 598149 | 595676 | 573559 |
| 254 | 279.23 | 279.24 | (5Z,10E,14S)-14-hydroxy-2,6,10-trimethylpentadeca-5,10-dien-4-one | 3 | 7E+07 | 7E+07 | 7E+07 | 6E+07 | 5E+07 | 6E+07 | 8E+07 | 7E+07 | 7E+07 | 6E+07 | 7E+07 | 7E+07 |
| 255 | 295.23 | 171.1 | 9(10)-EpOME;(9R,10S)-(12Z)-9,10-Epoxyoctadecenoic acid | 2 | 1E+06 | 1E+06 | 1E+06 | 2E+06 | 2E+06 | 2E+06 | 1E+06 | 1E+06 | 1E+06 | 2E+06 | 1E+06 | 1E+06 |
| 256 | 295.23 | 195.14 | 13(S)-HODE;13(S)-Hydroxyoctadeca-9Z,11E-dienoic acid* | 1 | 3E+06 | 3E+06 | 3E+06 | 3E+06 | 3E+06 | 3E+06 | 3E+06 | 3E+06 | 3E+06 | 3E+06 | 3E+06 | 3E+06 |
| 257 | 295.23 | 195.14 | 9S-Hydroxy-10E,12Z-octadecadienoic acid* | 1 | 3E+06 | 3E+06 | 2E+06 | 3E+06 | 3E+06 | 3E+06 | 2E+06 | 3E+06 | 2E+06 | 3E+06 | 3E+06 | 3E+06 |
| 258 | 295.23 | 195.14 | alpha-Hydroxylinoleic acid* | 1 | 3E+06 | 2E+06 | 2E+06 | 3E+06 | 3E+06 | 3E+06 | 3E+06 | 3E+06 | 2E+06 | 3E+06 | 3E+06 | 3E+06 |
| 259 | 311.22 | 171.14 | 13S-Hydroperoxy-9Z,11E-octadecadienoic acid | 3 | 6E+06 | 6E+06 | 7E+06 | 1E+07 | 1E+07 | 1E+07 | 6E+06 | 6E+06 | 6E+06 | 8E+06 | 7E+06 | 7E+06 |
| 260 | 311.22 | 223.17 | Hydroperoxylinoleic acid | 2 | 889225 | 878903 | 895091 | 769170 | 775140 | 708535 | 1E+06 | 1E+06 | 1E+06 | 1E+06 | 974513 | 948404 |
| 261 | 311.22 | 293.21 | 9S-Hydroperoxy-10E,12Z-octadecadienoic acid | 3 | 3E+06 | 3E+06 | 3E+06 | 4E+06 | 4E+06 | 4E+06 | 3E+06 | 3E+06 | 3E+06 | 3E+06 | 3E+06 | 3E+06 |
| 262 | 281.25 | 281.25 | 11-Octadecanoic acid(Vaccenic acid)* | 1 | 5E+07 | 5E+07 | 5E+07 | 4E+07 | 4E+07 | 3E+07 | 5E+07 | 6E+07 | 5E+07 | 4E+07 | 4E+07 | 5E+07 |
| 263 | 281.25 | 281.25 | Elaidic Acid* | 1 | 7E+07 | 6E+07 | 6E+07 | 5E+07 | 5E+07 | 4E+07 | 7E+07 | 7E+07 | 7E+07 | 6E+07 | 6E+07 | 6E+07 |
| 264 | 281.25 | 281.25 | Petroselinic acid* | 1 | 6E+07 | 6E+07 | 6E+07 | 4E+07 | 5E+07 | 4E+07 | 5E+07 | 5E+07 | 6E+07 | 5E+07 | 5E+07 | 6E+07 |
| 265 | 313.24 | 183.14 | Hydroxy ricinoleic acid | 2 | 637578 | 635264 | 636880 | 618615 | 637228 | 610081 | 886267 | 910479 | 828543 | 748934 | 713517 | 707045 |
| 266 | 329.23 | 211.13 | Tianshic acid | 1 | 2E+07 | 2E+07 | 2E+07 | 2E+07 | 2E+07 | 2E+07 | 2E+07 | 2E+07 | 2E+07 | 2E+07 | 2E+07 | 2E+07 |
| 267 | 329.23 | 211.14 | 9,12,13-TriHOME; 9(S),12(S),13(S)-Trihydroxy-10(E)-octadecenoic acid | 2 | 2E+06 | 2E+06 | 2E+06 | 1E+06 | 1E+06 | 1E+06 | 2E+06 | 2E+06 | 2E+06 | 2E+06 | 2E+06 | 2E+06 |
| 268 | 329.23 | 229.15 | 9,10,13-Trihydroxy-11-Octadecenoic Acid | 3 | 1E+07 | 1E+07 | 1E+07 | 2E+07 | 2E+07 | 2E+07 | 1E+07 | 1E+07 | 1E+07 | 1E+07 | 1E+07 | 1E+07 |
| 269 | 329.23 | 311.22 | 9,10,11-Trihydroxy-12-octadecenoic acid | 1 | 1E+06 | 1E+06 | 1E+06 | 1E+06 | 1E+06 | 1E+06 | 1E+06 | 1E+06 | 1E+06 | 1E+06 | 1E+06 | 1E+06 |
| 270 | 331.25 | 157.12 | 9,10,18-Trihydroxystearic acid | 2 | 97411 | 98757 | 116610 | 51680 | 47035 | 62282 | 149403 | 155500 | 145122 | 112661 | 90641 | 113267 |
| 271 | 318.3 | 60.04 | 4-Hydroxysphinganine; Phytosphingosine | 3 | 368317 | 377620 | 319242 | 243618 | 245655 | 251436 | 311624 | 360789 | 359956 | 588728 | 597021 | 541950 |
| 272 | 391.1 | 149.03 | 5,7,4'-Trihydroxy-3,6,3',5'-Tetramethoxyflavone | 3 | 212058 | 191187 | 213379 | 152030 | 179045 | 161891 | 235749 | 235977 | 182384 | 161605 | 173771 | 194437 |
| 273 | 423.09 | 169.01 | 4-O-Galloylarbutin | 1 | 433106 | 496140 | 435309 | 563100 | 622780 | 510972 | 810411 | 879627 | 873111 | 656426 | 625190 | 620842 |
| 274 | 327.12 | 147.05 | Uvafzelic acid | 3 | 4E+06 | 4E+06 | 4E+06 | 4E+06 | 4E+06 | 4E+06 | 5E+06 | 4E+06 | 5E+06 | 1E+07 | 1E+07 | 4E+06 |
| 275 | 363.14 | 121.06 | Ligstral | 3 | 12987 | 18276 | 16963 | 16675 | 16660 | 28340 | 114896 | 53611 | 73246 | 35482 | 35208 | 55015 |
| 276 | 299.16 | 117.04 | Biondinin C | 1 | 754354 | 829367 | 807826 | 946419 | 858866 | 842305 | 2E+06 | 2E+06 | 2E+06 | 1E+06 | 1E+06 | 1E+06 |
| 277 | 299.17 | 145.03 | 7-O-Geranyl Umbelliferone | 2 | 67619 | 68671 | 63810 | 56489 | 62591 | 58771 | 134774 | 139436 | 129739 | 94976 | 84966 | 85932 |
| 278 | 429.19 | 267.14 | N-(gamma-L-glutamyl)tyramine O-glucoside | 3 | 58598 | 217814 | 183176 | 117174 | 125778 | 56207 | 217455 | 298826 | 317487 | 262843 | 162570 | 221731 |
| 279 | 287.2 | 269.21 | Trans-dehydrorosinone | 2 | 901365 | 909897 | 940327 | 598905 | 582333 | 585341 | 860419 | 912242 | 865910 | 780184 | 760408 | 768386 |
| 280 | 477.1983 | 181.0866 | 2-[4-(3-Hydroxypropyl)-2-methoxyphenoxy]-1,3-propanediol 1-glucoside | 3 | 9 | 9 | 9 | 3E+06 | 3E+06 | 2E+06 | 9 | 9 | 9 | 2E+06 | 5E+06 | 4E+06 |
| 281 | 307.2256 | 219.1724 | 3β,6β-Dihydroxy-15-nor-14-oxo8(17),12-labdadien-14-a | 3 | 54384 | 64307 | 59924 | 57826 | 55460 | 60694 | 44663 | 52931 | 48607 | 51586 | 57413 | 57534 |
| 282 | 309.24 | 179.14 | Sessilifol O | 3 | 64596 | 74312 | 58232 | 72231 | 89982 | 78653 | 53348 | 58235 | 54696 | 58456 | 68442 | 67545 |
| 283 | 375.24 | 177.16 | Megastigm-5-en-3,9-diol glucoside | 1 | 146540 | 145352 | 97522 | 131735 | 147231 | 97985 | 273600 | 257040 | 208542 | 143410 | 205155 | 148415 |
| 284 | 331.28 | 313.27 | 2-Palmitoylglycerol* | 1 | 78102 | 83269 | 94740 | 80013 | 84205 | 92266 | 99346 | 109360 | 133195 | 144076 | 250184 | 125657 |
| 285 | 426.26 | 285.24 | LysoPE 14:0* | 3 | 332598 | 289347 | 327691 | 213910 | 247623 | 213657 | 342373 | 372631 | 398772 | 280837 | 296876 | 248856 |
| 286 | 426.26 | 285.24 | LysoPE 14:0(2n isomer)* | 3 | 305052 | 328012 | 302191 | 163801 | 223369 | 188207 | 352484 | 349096 | 353196 | 242993 | 278830 | 274739 |
| 287 | 495.04 | 300.99 | 4,6-O-(S)-Hexahydroxydiphenoyl-β-D-glucuronide | 1 | 78093 | 68940 | 56364 | 145136 | 172117 | 177937 | 82373 | 84668 | 62205 | 80585 | 112560 | 76788 |
| 288 | 419.0973 | 383.07 | Luteolin-8-C-arabinoside | 3 | 337070 | 228683 | 335401 | 170033 | 130065 | 124196 | 334997 | 465765 | 373830 | 188465 | 265250 | 368815 |
| 289 | 435.09 | 303.05 | Morin-3-O-xyloside | 3 | 9E+06 | 4E+06 | 4E+06 | 3E+06 | 2E+06 | 1E+06 | 2E+06 | 3E+06 | 4E+06 | 3E+06 | 2E+06 | 4E+06 |
| 290 | 481.06 | 275 | 4,6-(S)-Hexahydroxydiphenoyl-D-glucose | 2 | 3E+07 | 3E+07 | 3E+07 | 6E+07 | 6E+07 | 6E+07 | 5E+07 | 5E+07 | 5E+07 | 4E+07 | 4E+07 | 4E+07 |
| 291 | 403.1 | 367.08 | Apigenin-6-C-arabinoside* | 2 | 6E+06 | 5E+06 | 6E+06 | 1E+06 | 2E+06 | 2E+06 | 3E+06 | 4E+06 | 4E+06 | 2E+06 | 4E+06 | 3E+06 |
| 292 | 403.1 | 367.08 | Apigenin-8-C-Arabinoside* | 2 | 5E+06 | 4E+06 | 5E+06 | 1E+06 | 2E+06 | 2E+06 | 3E+06 | 3E+06 | 3E+06 | 3E+06 | 4E+06 | 4E+06 |
| 293 | 435.09 | 168.01 | 1-Galloyl-6-O-Benzoyl Glucose | 2 | 8E+06 | 8E+06 | 8E+06 | 8E+06 | 7E+06 | 8E+06 | 5E+06 | 6E+06 | 6E+06 | 6E+06 | 7E+06 | 7E+06 |
| 294 | 467.08 | 423.09 | gentisic acid 5-O-β-D-(6'-O-galloyl)-gluco-pyranoside | 2 | 3E+06 | 3E+06 | 3E+06 | 3E+06 | 4E+06 | 3E+06 | 5E+06 | 5E+06 | 5E+06 | 4E+06 | 4E+06 | 4E+06 |
| 295 | 341.14 | 137.06 | 2,4,2',4'-tetrahydroxy-3'-prenylchalcone | 3 | 1E+06 | 865193 | 965567 | 953014 | 1E+06 | 941394 | 742013 | 626520 | 671837 | 811102 | 844373 | 989922 |
| 296 | 339.12 | 309.08 | (2S)-Abyssinone II | 3 | 417154 | 412151 | 427229 | 386831 | 441525 | 380938 | 319332 | 333132 | 334859 | 377811 | 385361 | 385288 |
| 297 | 339.12 | 309.08 | (E)-3-[(2S,3S)-2-(4-hydroxy-3-methoxyphenyl)-7-methoxy-3-methyl-2,3-dihydro-1-benzofuran-5-yl]prop-2-enal | 3 | 365170 | 408500 | 377211 | 380950 | 411956 | 398083 | 264362 | 327213 | 310555 | 312683 | 340679 | 365488 |
| 298 | 357.13 | 307.09 | Horsfieldin | 2 | 274563 | 284783 | 331789 | 374896 | 384491 | 435070 | 370518 | 316194 | 269521 | 319416 | 306335 | 319807 |
| 299 | 373.13 | 343.08 | [(1R,2S)-1-(1,3-benzodioxol-5-yl)-2-methyl-3-oxobutyl]4-hydroxy-3-methoxybenzoate | 3 | 218650 | 266220 | 247796 | 289878 | 301920 | 290608 | 165171 | 176601 | 174464 | 208578 | 216000 | 237909 |
| 300 | 373.13 | 343.08 | 3',4',5',5,7-Pentamethoxyflavone* | 3 | 242722 | 235786 | 264207 | 269754 | 308779 | 229755 | 158377 | 170152 | 142092 | 179479 | 214546 | 207990 |
| 301 | 373.13 | 343.09 | Tangeretin (4',5,6,7,8-Pentamethoxyflavone)* | 3 | 254317 | 301401 | 303218 | 348437 | 399475 | 369790 | 205129 | 219284 | 165479 | 272418 | 258094 | 254086 |
| 302 | 453.11 | 313.06 | 3-Hydroxy-5-Methoxyphenyl-6-O-Galloyl-Beta-D-Galactopyranoside | 3 | 2E+06 | 1E+06 | 2E+06 | 3E+06 | 2E+06 | 3E+06 | 3E+06 | 3E+06 | 4E+06 | 3E+06 | 3E+06 | 3E+06 |
| 303 | 341.14 | 311.09 | 4,9-Dihydroxy-17-methoxy-2-oxatricyclo[13.2.2.13,7]icoSa-1(17),3,5,7(20),15,18-hexaen-10-one | 3 | 2E+06 | 2E+06 | 2E+06 | 1E+06 | 1E+06 | 1E+06 | 1E+06 | 2E+06 | 2E+06 | 1E+06 | 1E+06 | 1E+06 |
| 304 | 359.15 | 137.06 | sanshodiol | 1 | 1E+06 | 1E+06 | 1E+06 | 2E+06 | 2E+06 | 2E+06 | 1E+06 | 1E+06 | 1E+06 | 1E+06 | 1E+06 | 1E+06 |
| 305 | 359.15 | 137.06 | Matairesinol | 1 | 1E+06 | 1E+06 | 1E+06 | 2E+06 | 2E+06 | 2E+06 | 1E+06 | 1E+06 | 1E+06 | 1E+06 | 1E+06 | 1E+06 |
| 306 | 405.12 | 153.11 | (2S,3R,4S,5S,6R)-2-(2,5-dihydroxy-4-((E)-3-hydroxystyryl)phenoxy)-6-(hydroxymethyl)tetrahydro-2H-pyran-3,4,5-triol | 3 | 1E+06 | 1E+06 | 2E+06 | 797071 | 840631 | 810572 | 1E+06 | 1E+06 | 1E+06 | 1E+06 | 1E+06 | 1E+06 |
| 307 | 325.19 | 307.18 | Quinidine | 3 | 123680 | 186795 | 308670 | 188594 | 132979 | 411449 | 440436 | 334874 | 474510 | 291329 | 332742 | 286490 |
| 308 | 457.13 | 163.04 | Apiosylskimmin (Adicardin) | 2 | 448556 | 487977 | 474766 | 473423 | 538729 | 437585 | 367294 | 417550 | 278670 | 455492 | 413437 | 403142 |
| 309 | 377.16 | 163.08 | guaiacylglycerol-β-coniferyl ether | 1 | 497804 | 584522 | 621947 | 434330 | 422797 | 485142 | 574370 | 572333 | 504657 | 402214 | 490209 | 519647 |
| 310 | 375.15 | 327.12 | Erythro-Guaiacylglycerol-β-Coniferyl Ether | 3 | 162501 | 96181 | 134283 | 313685 | 183455 | 150085 | 101710 | 108741 | 56685 | 95035 | 83975 | 105106 |
| 311 | 391.14 | 343.12 | Demethyl-erythro-Guaiacylglycerol β-Sinapyl Ether | 3 | 345795 | 343477 | 331852 | 239149 | 290995 | 269458 | 356638 | 357145 | 373316 | 277072 | 295349 | 260295 |
| 312 | 439.1048 | 409.0966 | Epicatechin Tetramethyl Ether 3-Methylsulfate | 3 | 3E+06 | 3E+06 | 3E+06 | 2E+06 | 2E+06 | 2E+06 | 3E+06 | 3E+06 | 3E+06 | 3E+06 | 3E+06 | 2E+06 |
| 313 | 313.18 | 255.1 | 3,4,4'-Trihydroxy-5,5'-diisopropyl-2,2'-dimethylbiphenyl | 1 | 56871 | 31111 | 56404 | 74991 | 58452 | 29797 | 84231 | 99187 | 113324 | 51617 | 74449 | 27031 |
| 314 | 361.1657 | 180.078 | Secoisolariciresinol | 3 | 91700 | 81695 | 51724 | 9151.1 | 1670 | 5902 | 8856.4 | 5715.1 | 13240 | 16242 | 22917 | 21409 |
| 315 | 377.1596 | 329.1389 | 4,7,9,9'-Tetrahydroxy-3,3'-dimethoxy-8-O-4'-neolignan | 3 | 5E+06 | 6E+06 | 6E+06 | 3E+06 | 4E+06 | 4E+06 | 6E+06 | 6E+06 | 5E+06 | 5E+06 | 5E+06 | 5E+06 |
| 316 | 331.19 | 249.19 | 2-hydroxy-3-(4,8,12-trimethyl-10-oxotrideca-3,7,11-trienyl)-2H-furan-5-one | 3 | 748648 | 779157 | 723088 | 530290 | 521155 | 487436 | 811490 | 750550 | 763234 | 865338 | 726983 | 746754 |
| 317 | 301.22 | 301.22 | Isopimaric acid | 3 | 5E+06 | 5E+06 | 5E+06 | 6E+06 | 6E+06 | 5E+06 | 9E+06 | 8E+06 | 7E+06 | 6E+06 | 5E+06 | 6E+06 |
| 318 | 319.23 | 301.22 | Hispanolone | 1 | 1E+06 | 1E+06 | 1E+06 | 1E+06 | 1E+06 | 1E+06 | 1E+06 | 1E+06 | 1E+06 | 1E+06 | 1E+06 | 1E+06 |
| 319 | 333.2 | 251.2 | Agathic acid | 3 | 943244 | 1E+06 | 987296 | 895225 | 883356 | 881170 | 1E+06 | 1E+06 | 1E+06 | 1E+06 | 1E+06 | 994999 |
| 320 | 333.2 | 251.2 | Epoxyeleganolactone | 3 | 1E+06 | 1E+06 | 1E+06 | 987726 | 963292 | 940284 | 1E+06 | 1E+06 | 1E+06 | 1E+06 | 1E+06 | 1E+06 |
| 321 | 611.14 | 306.08 | Oxiglutatione | 2 | 6E+06 | 6E+06 | 6E+06 | 1E+07 | 1E+07 | 9E+06 | 7E+06 | 7E+06 | 7E+06 | 6E+06 | 1E+07 | 7E+06 |
| 322 | 321.24 | 123.08 | Ent-16α,17-Dihydroxykauran-2-one | 2 | 658893 | 699324 | 769908 | 384126 | 363973 | 353190 | 770438 | 681634 | 669132 | 566379 | 630781 | 628126 |
| 323 | 321.2421 | 179.1404 | 14-Hydroxyvibsanin F | 3 | 923198 | 904014 | 907576 | 521935 | 506175 | 537541 | 1E+06 | 1E+06 | 989279 | 767325 | 757942 | 769696 |
| 324 | 335.22 | 253.22 | 3,9-Dihydroxy-13(14)-labden-16,15-olide | 1 | 2E+06 | 1E+06 | 1E+06 | 1E+06 | 1E+06 | 1E+06 | 1E+06 | 2E+06 | 2E+06 | 2E+06 | 1E+06 | 1E+06 |
| 325 | 339.25 | 121.1 | 5,6-DiHETrE[(±)5,6-dihydroxy-8Z,11Z,14Z-eicosatrienoic acid] | 3 | 519848 | 539027 | 509814 | 296925 | 263294 | 198573 | 646615 | 763959 | 705248 | 328860 | 380056 | 409288 |
| 326 | 324.29 | 62.06 | linoleoyl ethanolamine | 2 | 3E+06 | 3E+06 | 3E+06 | 4E+06 | 4E+06 | 4E+06 | 3E+06 | 3E+06 | 3E+06 | 3E+06 | 3E+06 | 3E+06 |
| 327 | 438.26 | 297.24 | LysoPE 15:1(2n isomer)* | 3 | 107008 | 132091 | 103913 | 54118 | 89230 | 60900 | 118699 | 105060 | 122519 | 87548 | 90360 | 88088 |
| 328 | 327.29 | 59.01 | Hydroxyicosanoic Acid | 3 | 163041 | 147019 | 143282 | 164481 | 151107 | 126515 | 110456 | 123107 | 106321 | 150150 | 104580 | 128579 |
| 329 | 440.28 | 299.26 | LysoPE 15:0* | 3 | 275694 | 248230 | 259854 | 149034 | 143165 | 141127 | 379183 | 348659 | 351807 | 219919 | 238737 | 235185 |
| 330 | 440.28 | 299.26 | LysoPE 15:0(2n isomer)* | 3 | 245928 | 143412 | 118676 | 99217 | 102536 | 144310 | 301351 | 284558 | 304783 | 238061 | 209376 | 241164 |
| 331 | 473.04 | 169.01 | Trigallic acid | 2 | 3E+06 | 3E+06 | 4E+06 | 2E+06 | 1E+06 | 574820 | 1E+06 | 2E+06 | 4E+06 | 1E+06 | 2E+06 | 2E+06 |
| 332 | 427.07 | 289.04 | Theaflavic acid | 2 | 4E+06 | 5E+06 | 7E+06 | 4E+06 | 4E+06 | 2E+06 | 7E+06 | 7E+06 | 6E+06 | 6E+06 | 6E+06 | 6E+06 |
| 333 | 477.06 | 169.01 | 3,4-Digalloylshikimic acid | 1 | 1E+06 | 2E+06 | 1E+06 | 427310 | 1E+06 | 739215 | 2E+06 | 2E+06 | 2E+06 | 2E+06 | 2E+06 | 2E+06 |
| 334 | 495.08 | 319.04 | Myricetin-3-O-glucuronide | 3 | 62816 | 61480 | 57874 | 273519 | 239165 | 383979 | 226850 | 245215 | 223419 | 152701 | 180955 | 194518 |
| 335 | 433.11 | 271.06 | Galangin-7-O-glucoside | 3 | 5E+06 | 5E+06 | 5E+06 | 3E+06 | 4E+06 | 4E+06 | 5E+06 | 6E+06 | 5E+06 | 4E+06 | 5E+06 | 5E+06 |
| 336 | 433.11 | 271.06 | Sophoricoside | 3 | 4E+06 | 5E+06 | 4E+06 | 3E+06 | 3E+06 | 3E+06 | 5E+06 | 4E+06 | 4E+06 | 4E+06 | 4E+06 | 4E+06 |
| 337 | 433.11 | 271.06 | Apigenin-5-O-glucoside | 3 | 3E+06 | 3E+06 | 2E+06 | 2E+06 | 2E+06 | 1E+06 | 3E+06 | 3E+06 | 3E+06 | 2E+06 | 2E+06 | 2E+06 |
| 338 | 433.11 | 271.06 | Genistein-7-O-galactoside | 3 | 4E+06 | 5E+06 | 4E+06 | 3E+06 | 3E+06 | 3E+06 | 5E+06 | 4E+06 | 4E+06 | 4E+06 | 4E+06 | 4E+06 |
| 339 | 433.11 | 271.07 | Apigenin-4'-O-glucoside | 3 | 5E+06 | 4E+06 | 6E+06 | 3E+06 | 3E+06 | 4E+06 | 5E+06 | 5E+06 | 6E+06 | 4E+06 | 5E+06 | 5E+06 |
| 340 | 433.11 | 283.06 | Genistein-8-C-glucoside | 3 | 9E+07 | 8E+07 | 8E+07 | 2E+07 | 2E+07 | 3E+07 | 5E+07 | 5E+07 | 3E+07 | 4E+07 | 6E+07 | 3E+07 |
| 341 | 431.1 | 285.04 | Kaempferol-3-O-rhamnoside (Afzelin)(Kaempferin) | 1 | 2E+06 | 2E+06 | 2E+06 | 2E+06 | 2E+06 | 2E+06 | 3E+06 | 2E+06 | 2E+06 | 2E+06 | 2E+06 | 2E+06 |
| 342 | 431.1 | 285.04 | Kaempferol-7-O-rhamnoside | 2 | 2E+06 | 2E+06 | 2E+06 | 2E+06 | 2E+06 | 2E+06 | 3E+06 | 2E+06 | 3E+06 | 2E+06 | 2E+06 | 2E+06 |
| 343 | 431.1 | 311.05 | Apigenin-6-C-glucoside (Isovitexin) | 1 | 1E+07 | 1E+07 | 1E+07 | 4E+06 | 4E+06 | 4E+06 | 7E+06 | 8E+06 | 8E+06 | 8E+06 | 8E+06 | 8E+06 |
| 344 | 433.11 | 313.07 | Apigenin-8-C-Glucoside (Vitexin) | 1 | 4E+07 | 4E+07 | 5E+07 | 2E+07 | 2E+07 | 2E+07 | 5E+07 | 4E+07 | 5E+07 | 4E+07 | 3E+07 | 2E+07 |
| 345 | 447.09 | 285.04 | Kaempferol-7-O-glucoside* | 1 | 7E+07 | 7E+07 | 7E+07 | 6E+07 | 6E+07 | 6E+07 | 5E+07 | 5E+07 | 5E+07 | 6E+07 | 6E+07 | 6E+07 |
| 346 | 449.1056 | 287.0563 | Kaempferol-4'-O-glucoside* | 1 | 9E+06 | 1E+07 | 1E+07 | 2E+07 | 4E+07 | 1E+07 | 6E+06 | 8E+06 | 9E+06 | 2E+07 | 2E+07 | 9E+06 |
| 347 | 449.11 | 299.05 | Luteolin-8-C-glucoside (Orientin)* | 1 | 6E+06 | 5E+06 | 6E+06 | 6E+06 | 5E+06 | 5E+06 | 5E+06 | 5E+06 | 5E+06 | 5E+06 | 5E+06 | 5E+06 |
| 348 | 449.11 | 303.05 | Quercetin-3-O-rhamnoside(Quercitrin) | 3 | 2E+06 | 3E+06 | 3E+06 | 904598 | 859076 | 954860 | 649316 | 512421 | 487744 | 1E+06 | 2E+06 | 1E+06 |
| 349 | 465.1 | 303.05 | Rhodiolgin | 1 | 6E+07 | 6E+07 | 6E+07 | 3E+07 | 3E+07 | 4E+07 | 3E+07 | 2E+07 | 2E+07 | 4E+07 | 2E+07 | 5E+07 |
| 350 | 465.1 | 303.05 | Quercetin-5-O-β-D-glucoside* | 1 | 2E+07 | 1E+07 | 8E+06 | 1E+07 | 6E+06 | 1E+07 | 6E+06 | 7E+06 | 6E+06 | 1E+07 | 7E+06 | 1E+07 |
| 351 | 465.1 | 333.07 | 6-Methoxyquercetin-3-O-Xyloside | 3 | 99749 | 200145 | 156760 | 32150 | 36208 | 27268 | 13753 | 70572 | 58525 | 78263 | 46625 | 74473 |
| 352 | 481.1 | 319.05 | Gossypetin-3-O-glucoside | 2 | 3E+07 | 2E+07 | 2E+07 | 2E+07 | 2E+07 | 2E+07 | 7E+06 | 8E+06 | 2E+07 | 2E+07 | 3E+07 | 2E+07 |
| 353 | 495.08 | 343.07 | 3-O-Digalloyl quinic acid | 1 | 1E+07 | 2E+07 | 1E+07 | 2E+07 | 2E+07 | 2E+07 | 2E+07 | 2E+07 | 2E+07 | 2E+07 | 2E+07 | 2E+07 |
| 354 | 417.12 | 381.1 | Apigenin-6-C-rhamnoside | 3 | 255669 | 296363 | 274926 | 197586 | 115050 | 177691 | 312509 | 279739 | 279217 | 230961 | 248415 | 217563 |
| 355 | 433.11 | 271.06 | Choerospondin | 2 | 1E+06 | 1E+06 | 1E+06 | 539045 | 1E+06 | 653561 | 567951 | 560506 | 643581 | 713498 | 1E+06 | 661969 |
| 356 | 435.13 | 273.07 | Rubrofusarin-6-O-glucoside | 2 | 191597 | 300275 | 301342 | 462602 | 509213 | 456601 | 243712 | 270285 | 401594 | 313389 | 423343 | 363666 |
| 357 | 435.13 | 273.08 | 3,4-Dihydro-4-(4'-hydroxyphenyl)-5,7-dihydroxycoumarin glucoside | 2 | 273049 | 268357 | 341723 | 622483 | 572231 | 530753 | 418265 | 363745 | 335639 | 294497 | 290694 | 343741 |
| 358 | 449.11 | 287.06 | Dihydrokaempferol-7-O-glucoside | 3 | 9 | 9 | 9 | 348256 | 1E+06 | 466149 | 459667 | 668991 | 480215 | 463246 | 398588 | 609935 |
| 359 | 451.12 | 289.07 | Eriodictyol-7-O-glucoside | 3 | 3E+06 | 6E+06 | 7E+06 | 2E+07 | 8E+06 | 5E+06 | 2E+06 | 5E+06 | 1E+06 | 3E+06 | 7E+06 | 5E+06 |
| 360 | 465.1034 | 313.0554 | 1-Methoxybenzoyl-6-O-Galloyl-D-Glucose | 3 | 3E+06 | 4E+06 | 4E+06 | 2E+06 | 2E+06 | 2E+06 | 3E+06 | 3E+06 | 3E+06 | 2E+06 | 2E+06 | 2E+06 |
| 361 | 481.14 | 319.08 | Dihydromyricetin-3-O-glucoside | 3 | 240752 | 250129 | 270874 | 122080 | 207274 | 180032 | 255496 | 234846 | 245375 | 218351 | 249431 | 253439 |
| 362 | 403.14 | 373.09 | 3,5,6,7,8,4'-Hexamethoxyflavone | 3 | 349125 | 297063 | 384080 | 314800 | 338456 | 320009 | 155439 | 171123 | 139415 | 266621 | 249082 | 238747 |
| 363 | 403.14 | 373.1 | Nobiletin (5,6,7,8,3',4'-Hexamethoxyflavone) | 3 | 512392 | 522742 | 507274 | 500409 | 496072 | 474267 | 308324 | 298814 | 289426 | 391208 | 407882 | 396539 |
| 364 | 419.13 | 383.1 | O-MethylNaringenin-8-C-arabinoside | 3 | 623030 | 410595 | 495471 | 248555 | 256905 | 258575 | 332545 | 398893 | 512424 | 243545 | 369012 | 306928 |
| 365 | 435.13 | 273.08 | Dihydrocharcone-4'-O-glucoside | 3 | 2E+06 | 2E+06 | 2E+06 | 1E+06 | 1E+06 | 1E+06 | 1E+06 | 2E+06 | 1E+06 | 1E+06 | 1E+06 | 1E+06 |
| 366 | 435.1297 | 315.0884 | Phloretin-4'-O-glucoside (Trilobatin) | 3 | 808669 | 835346 | 791997 | 695030 | 461429 | 350446 | 360271 | 382386 | 433249 | 795555 | 835785 | 758685 |
| 367 | 451.12 | 289.07 | Catechin-5-O-glucoside | 3 | 169234 | 200978 | 245503 | 608025 | 636116 | 519728 | 628347 | 791220 | 423638 | 580543 | 463771 | 402871 |
| 368 | 469.13 | 153.02 | (3,4-dihydroxyphenethyl alcohol 4-O-D-(6'-O-galloyl)-glucopyranoside | 2 | 917474 | 937397 | 1E+06 | 1E+06 | 870055 | 926970 | 1E+06 | 1E+06 | 1E+06 | 1E+06 | 935953 | 1E+06 |
| 369 | 387.14 | 289.07 | Medioresinol | 1 | 1E+06 | 1E+06 | 1E+06 | 1E+06 | 1E+06 | 1E+06 | 1E+06 | 1E+06 | 1E+06 | 1E+06 | 1E+06 | 1E+06 |
| 370 | 664.12 | 136.06 | Nicotinic acid adenine dinucleotide | 2 | 1E+06 | 2E+06 | 2E+06 | 2E+06 | 3E+06 | 2E+06 | 1E+06 | 2E+06 | 1E+06 | 2E+06 | 2E+06 | 2E+06 |
| 371 | 407.17 | 165.06 | 1-(4'-Hydroxy-3'-methoxyphenyl)-2-[4''-(3-hydroxypropyl)-2'',6''-dimethoxyphenyl]-propane-1,3-Diol | 1 | 691966 | 610789 | 834720 | 390356 | 436243 | 395957 | 553689 | 648512 | 599155 | 614465 | 523429 | 582273 |
| 372 | 425.18 | 147.04 | Grandidentatin | 3 | 1E+06 | 2E+06 | 1E+06 | 2E+06 | 3E+06 | 2E+06 | 2E+06 | 1E+06 | 1E+06 | 937204 | 2E+06 | 2E+06 |
| 373 | 427.2 | 247.13 | Abscisic acid D-glucopyranosyl ester | 3 | 1E+06 | 323705 | 427905 | 1E+06 | 1E+06 | 1E+06 | 937642 | 1E+06 | 1E+06 | 1E+06 | 1E+06 | 2E+06 |
| 374 | 443.19 | 59.01 | Ebuloside | 3 | 263529 | 254692 | 292103 | 284855 | 255247 | 277590 | 326767 | 315150 | 318507 | 329606 | 264620 | 272835 |
| 375 | 351.25 | 147.12 | 1-Stearidonoyl-Glycerol | 1 | 2E+06 | 2E+06 | 2E+06 | 3E+06 | 3E+06 | 3E+06 | 2E+06 | 2E+06 | 2E+06 | 2E+06 | 2E+06 | 2E+06 |
| 376 | 351.25 | 259.2 | 15-Hydroxysessilifol F | 3 | 4E+06 | 4E+06 | 4E+06 | 6E+06 | 7E+06 | 6E+06 | 4E+06 | 4E+06 | 4E+06 | 4E+06 | 4E+06 | 5E+06 |
| 377 | 337.27 | 263.24 | (Oxiran-2-yl)methyl octadeca-9,12-dienoate | 1 | 646197 | 647327 | 735725 | 201290 | 231982 | 252475 | 1E+06 | 1E+06 | 1E+06 | 667059 | 695277 | 765840 |
| 378 | 337.27 | 263.24 | Glycidyl Linoleate | 1 | 223377 | 228533 | 267625 | 238569 | 218287 | 230771 | 343119 | 316929 | 331916 | 232008 | 255974 | 248860 |
| 379 | 353.27 | 261.22 | (S)-2,3-dihydroxypropyl (9Z,12Z,15Z)-octadeca-9,12,15-trienoate | 3 | 4E+07 | 4E+07 | 4E+07 | 1E+07 | 1E+07 | 1E+07 | 4E+07 | 5E+07 | 5E+07 | 3E+07 | 3E+07 | 3E+07 |
| 380 | 353.27 | 261.22 | 1-Monolinolenoyl-Rac-Glycerol | 1 | 2E+07 | 2E+07 | 2E+07 | 2E+07 | 2E+07 | 2E+07 | 2E+07 | 2E+07 | 2E+07 | 2E+07 | 2E+07 | 2E+07 |
| 381 | 353.27 | 261.22 | 1-α-Linolenoyl-glycerol* | 1 | 2E+07 | 2E+07 | 2E+07 | 2E+07 | 2E+07 | 2E+07 | 2E+07 | 2E+07 | 2E+07 | 2E+07 | 2E+07 | 2E+07 |
| 382 | 353.27 | 261.22 | Glycerol 9(E),11(Z),13(E)-octadecatrienoyl ester | 1 | 2E+07 | 2E+07 | 2E+07 | 2E+07 | 2E+07 | 2E+07 | 2E+07 | 2E+07 | 2E+07 | 2E+07 | 2E+07 | 2E+07 |
| 383 | 353.27 | 261.22 | Monolinolenin* | 1 | 2E+07 | 2E+07 | 2E+07 | 2E+07 | 2E+07 | 2E+07 | 2E+07 | 2E+07 | 2E+07 | 2E+07 | 2E+07 | 2E+07 |
| 384 | 353.2686 | 261.2213 | 2-α-Linolenoyl-glycerol* | 1 | 2E+07 | 2E+07 | 2E+07 | 2E+07 | 2E+07 | 2E+07 | 2E+07 | 3E+07 | 2E+07 | 2E+07 | 2E+07 | 2E+07 |
| 385 | 448.25 | 307.23 | LysoPE 16:3 | 3 | 60134 | 41065 | 49021 | 13147 | 16579 | 28355 | 61608 | 45662 | 51142 | 41169 | 22310 | 45029 |
| 386 | 477.27 | 161.04 | Hydroxypentadecenoic acid glucoside | 2 | 966509 | 1E+06 | 1E+06 | 1E+06 | 1E+06 | 1E+06 | 1E+06 | 1E+06 | 1E+06 | 1E+06 | 1E+06 | 1E+06 |
| 387 | 357.3 | 265.25 | 1-Oleoyl-Sn-Glycerol | 1 | 983148 | 543972 | 426794 | 350728 | 200058 | 253065 | 624531 | 2E+06 | 416415 | 553499 | 454934 | 438811 |
| 388 | 452.28 | 311.26 | LysoPE 16:1* | 1 | 1E+06 | 1E+06 | 1E+06 | 790452 | 788851 | 788695 | 2E+06 | 2E+06 | 2E+06 | 1E+06 | 1E+06 | 1E+06 |
| 389 | 452.28 | 311.26 | LysoPE 16:1(2n isomer)* | 1 | 1E+06 | 2E+06 | 1E+06 | 792737 | 766921 | 715428 | 2E+06 | 2E+06 | 2E+06 | 1E+06 | 1E+06 | 1E+06 |
| 390 | 454.29 | 313.27 | LysoPE 16:0 | 2 | 1E+06 | 1E+06 | 1E+06 | 1E+06 | 1E+06 | 1E+06 | 1E+06 | 2E+06 | 2E+06 | 2E+06 | 1E+06 | 1E+06 |
| 391 | 454.29 | 313.27 | LysoPE 16:0(2n isomer) | 3 | 1E+06 | 1E+06 | 1E+06 | 1E+06 | 1E+06 | 907889 | 2E+06 | 1E+06 | 1E+06 | 1E+06 | 2E+06 | 1E+06 |
| 392 | 344.13 | 147.04 | N-Phenylethylcrinasiadine | 3 | 2E+06 | 3E+06 | 2E+06 | 2E+06 | 5E+06 | 2E+06 | 707959 | 1E+06 | 1E+06 | 2E+06 | 1E+06 | 2E+06 |
| 393 | 441.08 | 289.07 | 7-O-Galloyltricetiflavan | 2 | 1E+07 | 1E+07 | 1E+07 | 9E+06 | 8E+06 | 8E+06 | 1E+07 | 1E+07 | 1E+07 | 8E+06 | 1E+07 | 1E+07 |
| 394 | 459.09 | 139.04 | Gallocatechin gallate* | 1 | 3E+07 | 3E+07 | 5E+07 | 2E+07 | 1E+07 | 2E+07 | 2E+07 | 2E+07 | 3E+07 | 4E+07 | 2E+07 | 3E+07 |
| 395 | 473.0736 | 319.0462 | 3-Dehydroshikimic Acid 5-(3-Vanilloyl)Gallate | 3 | 358538 | 364116 | 375483 | 322485 | 350127 | 298165 | 452029 | 494721 | 460444 | 360949 | 365249 | 390438 |
| 396 | 473.0721 | 321.0617 | Elephantorrhizol 3-Gallate | 3 | 89887 | 114393 | 109657 | 63867 | 52550 | 52990 | 78834 | 83406 | 89854 | 77289 | 74178 | 86505 |
| 397 | 461.11 | 315.05 | 8-Methoxykaempferol-7-O-rhamnoside | 3 | 345126 | 322653 | 351243 | 297037 | 241598 | 246153 | 387182 | 439180 | 345884 | 352413 | 348441 | 336054 |
| 398 | 493.1 | 331.05 | Mearnsetin-3-O-glucoside | 2 | 1E+06 | 1E+06 | 1E+06 | 531134 | 480648 | 595621 | 804391 | 788527 | 757236 | 824947 | 960575 | 809244 |
| 399 | 495.11 | 333.07 | Laricitrin-3-O-glucoside | 2 | 3E+06 | 1E+06 | 2E+06 | 846876 | 983214 | 956078 | 648713 | 1E+06 | 538951 | 891357 | 2E+06 | 999547 |
| 400 | 449.14 | 287.1 | Isosakuranetin-7-O-glucoside (Isosakuranin) | 3 | 1E+06 | 1E+06 | 1E+06 | 674472 | 804806 | 779820 | 1E+06 | 1E+06 | 1E+06 | 1E+06 | 2E+06 | 1E+06 |
| 401 | 481.13 | 319.05 | 3',5',5,7-Tetrahydroxy-4'-methoxyflavanone-3'-O-glucoside | 1 | 2E+07 | 2E+07 | 2E+07 | 3E+07 | 9E+06 | 2E+07 | 7E+06 | 9E+06 | 1E+07 | 2E+07 | 2E+07 | 2E+07 |
| 402 | 433.15 | 403.11 | 3,5,6,7,8,3',4'-Heptamethoxyflavone | 3 | 61972 | 63325 | 78653 | 132029 | 129390 | 142305 | 27738 | 21972 | 15464 | 71178 | 59774 | 71089 |
| 403 | 499.15 | 163.2 | 3-O-p-Coumaroylquinic acid-O-glucoside | 2 | 2E+06 | 2E+06 | 2E+06 | 2E+06 | 2E+06 | 1E+06 | 2E+06 | 2E+06 | 2E+06 | 2E+06 | 2E+06 | 2E+06 |
| 404 | 425.22 | 365.19 | Scutebarbolide C | 3 | 1E+07 | 1E+07 | 1E+07 | 1E+07 | 1E+07 | 1E+07 | 1E+07 | 1E+07 | 1E+07 | 1E+07 | 1E+07 | 1E+07 |
| 405 | 377.23 | 295.23 | Negundoin A | 3 | 2E+07 | 2E+07 | 2E+07 | 2E+07 | 2E+07 | 3E+07 | 2E+07 | 2E+07 | 2E+07 | 3E+07 | 2E+07 | 3E+07 |
| 406 | 377.23 | 295.23 | Vitexilactone | 1 | 2E+07 | 2E+07 | 2E+07 | 2E+07 | 2E+07 | 2E+07 | 2E+07 | 2E+07 | 2E+07 | 3E+07 | 3E+07 | 3E+07 |
| 407 | 425.22 | 365.2 | Cinnzeylanine | 3 | 81647 | 78773 | 99634 | 128555 | 138259 | 119502 | 95023 | 87836 | 64795 | 69492 | 86059 | 108148 |
| 408 | 349.27 | 123.08 | Anacardic acid | 2 | 4E+06 | 4E+06 | 4E+06 | 3E+06 | 3E+06 | 3E+06 | 4E+06 | 4E+06 | 4E+06 | 3E+06 | 3E+06 | 3E+06 |
| 409 | 349.27 | 179.14 | Ent-3β-Acetoxyisopimar-15-8β-ol | 1 | 2E+07 | 2E+07 | 2E+07 | 1E+07 | 1E+07 | 1E+07 | 2E+07 | 2E+07 | 2E+07 | 1E+07 | 1E+07 | 1E+07 |
| 410 | 338.34 | 338.34 | octadecanoic acid pyrrolidide | 2 | 3E+06 | 3E+06 | 3E+06 | 2E+06 | 2E+06 | 2E+06 | 4E+06 | 4E+06 | 4E+06 | 3E+06 | 3E+06 | 3E+06 |
| 411 | 481.2578 | 253.2177 | LysoPG 16:1 | 3 | 560619 | 901670 | 724381 | 134018 | 139371 | 144020 | 920953 | 869276 | 738194 | 278857 | 454378 | 484967 |
| 412 | 466.29 | 325.27 | LysoPE 17:1* | 3 | 474225 | 582080 | 579648 | 191179 | 215697 | 229453 | 413447 | 432555 | 407904 | 344453 | 341844 | 448177 |
| 413 | 466.29 | 325.27 | LysoPE 17:1(2n isomer)* | 3 | 435071 | 314160 | 345789 | 130419 | 265780 | 218684 | 457072 | 400312 | 463151 | 332767 | 373039 | 441609 |
| 414 | 483.2736 | 255.2336 | LysoPG 16:0 | 3 | 228785 | 257887 | 325185 | 102855 | 170859 | 167404 | 309694 | 449401 | 408963 | 165973 | 247495 | 269753 |
| 415 | 468.31 | 184.07 | LysoPC 14:0 | 1 | 5E+06 | 5E+06 | 5E+06 | 4E+06 | 4E+06 | 3E+06 | 6E+06 | 6E+06 | 6E+06 | 4E+06 | 4E+06 | 4E+06 |
| 416 | 455.1 | 289.07 | Epicatechin-3-(3''-O-methyl)gallate | 2 | 1E+07 | 1E+07 | 6E+06 | 3E+06 | 4E+06 | 3E+06 | 5E+06 | 1E+07 | 6E+06 | 5E+06 | 5E+06 | 5E+06 |
| 417 | 471.0951 | 183.0299 | Epigallocatechin 3-O-(3-O-Methyl)Gallate | 3 | 318251 | 565184 | 492031 | 147690 | 268357 | 179207 | 349217 | 807285 | 303938 | 447740 | 330113 | 260779 |
| 418 | 475.12 | 271.06 | Apigenin-7-O-(6''-acetyl)glucoside | 3 | 19502 | 14237 | 9351.1 | 14581 | 26675 | 8340 | 91553 | 269376 | 103558 | 52625 | 51075 | 45802 |
| 419 | 489.11 | 285.04 | Kaempferol-3-O-(6''-O-acetyl)glucoside | 1 | 3E+07 | 3E+07 | 3E+07 | 3E+07 | 3E+07 | 3E+07 | 2E+07 | 2E+07 | 2E+07 | 3E+07 | 2E+07 | 2E+07 |
| 420 | 505.1 | 300.03 | Quercetin-3-O-(6''-O-acetyl)galactoside | 3 | 5E+06 | 3E+06 | 3E+06 | 2E+06 | 2E+06 | 2E+06 | 2E+06 | 2E+06 | 2E+06 | 2E+06 | 2E+06 | 2E+06 |
| 421 | 507.12 | 303.05 | Quercetin-3-O-(6''-O-acetyl)glucoside | 3 | 599600 | 409850 | 600131 | 166770 | 126730 | 210160 | 163430 | 266920 | 136679 | 156760 | 216835 | 293640 |
| 422 | 493.13 | 331.08 | Flavoyadorinin A | 3 | 2E+07 | 2E+07 | 2E+07 | 8E+06 | 8E+06 | 7E+06 | 1E+07 | 1E+07 | 9E+06 | 1E+07 | 9E+06 | 1E+07 |
| 423 | 493.1341 | 331.0812 | Tricin-4'-O-glucoside | 3 | 6E+06 | 5E+06 | 6E+06 | 3E+06 | 2E+06 | 3E+06 | 3E+06 | 5E+06 | 5E+06 | 4E+06 | 5E+06 | 5E+06 |
| 424 | 493.1326 | 331.0817 | Iristectorin A | 3 | 8E+06 | 5E+06 | 1E+07 | 3E+06 | 3E+06 | 4E+06 | 5E+06 | 8E+06 | 6E+06 | 4E+06 | 6E+06 | 9E+06 |
| 425 | 509.13 | 347.08 | 5,6,3',4'-Tetrahydroxy-3,7-dimethoxyflavone-6-O-glucoside | 3 | 405647 | 311299 | 454150 | 330564 | 231551 | 58799 | 9 | 9 | 9 | 287795 | 244333 | 110045 |
| 426 | 509.13 | 347.08 | Limocitrin 3-Glucoside | 3 | 405647 | 311299 | 454150 | 330564 | 231551 | 58799 | 9 | 9 | 9 | 287795 | 244333 | 110045 |
| 427 | 409.18 | 259.11 | p-Coumaroylferuloylputrescine | 3 | 142473 | 144913 | 181824 | 173089 | 184945 | 213308 | 148355 | 140120 | 115567 | 150122 | 156102 | 167913 |
| 428 | 477.14 | 315.09 | Persicoside | 3 | 173161 | 59950 | 167189 | 52711 | 51471 | 46651 | 140003 | 138151 | 66613 | 153012 | 106710 | 159416 |
| 429 | 581.17 | 239.06 | 6'-O-α-D-galactosylsesamoside | 2 | 673567 | 742684 | 579455 | 693733 | 721892 | 532729 | 391283 | 332060 | 382713 | 447900 | 442851 | 516291 |
| 430 | 476.28 | 335.26 | LysoPE 18:3 | 1 | 2E+06 | 2E+06 | 2E+06 | 649319 | 752515 | 716633 | 2E+06 | 2E+06 | 2E+06 | 2E+06 | 1E+06 | 1E+06 |
| 431 | 476.28 | 335.26 | LysoPE 18:3(2n isomer) | 1 | 1E+06 | 2E+06 | 1E+06 | 568033 | 547616 | 529353 | 2E+06 | 2E+06 | 2E+06 | 1E+06 | 1E+06 | 1E+06 |
| 432 | 478.29 | 337.27 | LysoPE 18:2 | 2 | 3E+06 | 3E+06 | 3E+06 | 1E+06 | 1E+06 | 850040 | 3E+06 | 3E+06 | 2E+06 | 3E+06 | 2E+06 | 2E+06 |
| 433 | 478.29 | 337.27 | LysoPE 18:2(2n isomer) | 2 | 2E+06 | 2E+06 | 2E+06 | 881636 | 801698 | 967330 | 3E+06 | 3E+06 | 3E+06 | 2E+06 | 2E+06 | 2E+06 |
| 434 | 480.31 | 184.07 | LysoPC 15:1 | 2 | 4E+06 | 3E+06 | 3E+06 | 2E+06 | 2E+06 | 3E+06 | 5E+06 | 5E+06 | 5E+06 | 2E+06 | 2E+06 | 2E+06 |
| 435 | 480.31 | 339.29 | LysoPE 18:1* | 3 | 8E+06 | 9E+06 | 9E+06 | 4E+06 | 4E+06 | 4E+06 | 1E+07 | 1E+07 | 1E+07 | 8E+06 | 8E+06 | 9E+06 |
| 436 | 480.31 | 339.29 | LysoPE 18:1(2n isomer)* | 3 | 5E+06 | 6E+06 | 7E+06 | 3E+06 | 3E+06 | 3E+06 | 8E+06 | 7E+06 | 8E+06 | 6E+06 | 6E+06 | 5E+06 |
| 437 | 482.32 | 184.07 | LysoPC 15:0* | 1 | 3E+06 | 4E+06 | 4E+06 | 2E+06 | 2E+06 | 2E+06 | 4E+06 | 4E+06 | 4E+06 | 3E+06 | 3E+06 | 3E+06 |
| 438 | 482.32 | 184.07 | LysoPC 15:0(2n isomer)* | 1 | 4E+06 | 4E+06 | 4E+06 | 2E+06 | 2E+06 | 2E+06 | 4E+06 | 5E+06 | 5E+06 | 3E+06 | 4E+06 | 3E+06 |
| 439 | 482.32 | 341.31 | LysoPE 18:0(2n isomer) | 3 | 96688 | 103030 | 71916 | 28037 | 36576 | 51994 | 78164 | 128520 | 126001 | 68124 | 80636 | 81780 |
| 440 | 535.11 | 287.05 | Kaempferol-3-O-(6''-malonyl)glucoside* | 1 | 2E+06 | 2E+06 | 5E+06 | 3E+06 | 2E+06 | 3E+06 | 810755 | 1E+06 | 1E+06 | 2E+06 | 2E+06 | 3E+06 |
| 441 | 535.11 | 287.05 | Eriodictyol-7-O-(6''-malonyl)glucoside | 2 | 13913 | 10511 | 12061 | 21675 | 23349 | 36675 | 24263 | 12726 | 24265 | 31587 | 14524 | 28165 |
| 442 | 507.15 | 345.1 | Rhamnazine-4'-O-β-D-glucoside | 2 | 2E+06 | 2E+06 | 2E+06 | 2E+06 | 2E+06 | 2E+06 | 3E+06 | 3E+06 | 3E+06 | 2E+06 | 2E+06 | 2E+06 |
| 443 | 507.15 | 345.1 | Rhamnazine-5-O-β-D-glucoside | 2 | 2E+06 | 2E+06 | 2E+06 | 2E+06 | 2E+06 | 2E+06 | 3E+06 | 2E+06 | 2E+06 | 2E+06 | 2E+06 | 2E+06 |
| 444 | 381.2 | 299.2 | (Z)-3,8-Dihydro 6,6·;7,3'a-diligustilide | 1 | 2E+06 | 3E+06 | 3E+06 | 3E+06 | 3E+06 | 3E+06 | 2E+06 | 3E+06 | 3E+06 | 3E+06 | 3E+06 | 3E+06 |
| 445 | 492.31 | 184.07 | LysoPC 16:2(2n isomer) | 1 | 4E+06 | 4E+06 | 4E+06 | 2E+06 | 2E+06 | 2E+06 | 5E+06 | 5E+06 | 5E+06 | 3E+06 | 3E+06 | 3E+06 |
| 446 | 494.32 | 184.07 | LysoPC 16:1* | 1 | 3E+07 | 3E+07 | 3E+07 | 1E+07 | 1E+07 | 1E+07 | 3E+07 | 3E+07 | 3E+07 | 2E+07 | 2E+07 | 2E+07 |
| 447 | 494.32 | 184.07 | LysoPC 16:1(2n isomer)* | 1 | 3E+07 | 3E+07 | 3E+07 | 1E+07 | 1E+07 | 1E+07 | 3E+07 | 3E+07 | 3E+07 | 2E+07 | 2E+07 | 2E+07 |
| 448 | 496.34 | 184.07 | LysoPC 16:0(2n isomer)* | 1 | 5E+06 | 4E+06 | 5E+06 | 4E+06 | 4E+06 | 4E+06 | 5E+06 | 5E+06 | 5E+06 | 5E+06 | 4E+06 | 4E+06 |
| 449 | 451.1392 | 147.0447 | 3-Cinnamoyl-5-p-Coumaroylshikimic acid | 3 | 3E+07 | 3E+07 | 3E+07 | 3E+07 | 2E+07 | 2E+07 | 4E+07 | 3E+07 | 3E+07 | 3E+07 | 3E+07 | 3E+07 |
| 450 | 467.1332 | 147.0441 | 3,4-O-Di-p-Coumaroylshikimic acid | 3 | 8E+06 | 2E+07 | 1E+07 | 5E+06 | 5E+06 | 8E+06 | 6E+06 | 1E+07 | 1E+07 | 6E+06 | 6E+06 | 6E+06 |
| 451 | 467.133 | 163.0385 | 4-O-Cinnamoyl-5-O-caffeoylshikimic acid* | 3 | 1E+07 | 1E+07 | 1E+07 | 2E+07 | 1E+07 | 1E+07 | 2E+07 | 2E+07 | 2E+07 | 2E+07 | 2E+07 | 1E+07 |
| 452 | 467.1345 | 163.0394 | 3-O-Cinnamoyl-5-O-caffeoylshikimic acid* | 3 | 1E+07 | 1E+07 | 1E+07 | 1E+07 | 1E+07 | 1E+07 | 2E+07 | 2E+07 | 2E+07 | 1E+07 | 2E+07 | 1E+07 |
| 453 | 483.1305 | 321.0988 | 3-Cinnamoyl-5-Caffeoylquinic acid | 3 | 2E+06 | 2E+06 | 2E+06 | 2E+06 | 2E+06 | 2E+06 | 2E+06 | 2E+06 | 2E+06 | 2E+06 | 2E+06 | 2E+06 |
| 454 | 565.12 | 317.08 | Isorhamnetin-3-O-(6''-malonyl)glucoside | 3 | 9 | 9 | 9 | 35010 | 49895 | 120874 | 9 | 9 | 9 | 57824 | 143410 | 90754 |
| 455 | 467.1346 | 321.0993 | 3-Cinnamoyl-5-p-Coumaroylquinic acid | 3 | 2E+07 | 2E+07 | 2E+07 | 1E+07 | 1E+07 | 1E+07 | 2E+07 | 2E+07 | 2E+07 | 2E+07 | 2E+07 | 1E+07 |
| 456 | 535.14 | 481.11 | Apigenin-6-C-xyloside-8-C-arabinoside | 2 | 3E+07 | 3E+07 | 3E+07 | 1E+07 | 2E+07 | 1E+07 | 3E+07 | 3E+07 | 2E+07 | 2E+07 | 2E+07 | 1E+07 |
| 457 | 535.14 | 499.12 | Apigenin-6,8-di-C-arabinoside* | 1 | 5E+07 | 4E+07 | 5E+07 | 2E+07 | 2E+07 | 3E+07 | 4E+07 | 4E+07 | 4E+07 | 3E+07 | 3E+07 | 3E+07 |
| 458 | 535.14 | 499.12 | Apigenin-6-C-arabinoside-8-C-xyloside* | 1 | 2E+06 | 2E+06 | 2E+06 | 1E+06 | 1E+06 | 913965 | 2E+06 | 2E+06 | 2E+06 | 2E+06 | 1E+06 | 1E+06 |
| 459 | 551.14 | 497.1 | Luteolin-6,8-di-C-arabinoside | 3 | 644933 | 506827 | 546050 | 235546 | 220175 | 233530 | 360460 | 283620 | 1E+06 | 230919 | 428985 | 650357 |
| 460 | 567.13 | 303.05 | Quercetin-3-O-xylosyl(1→2)arabinoside | 3 | 9 | 9 | 9 | 9 | 9 | 9 | 17948 | 25285 | 18340 | 29886 | 26675 | 35010 |
| 461 | 438.24 | 292.2 | N1,N10-Bis(p-coumaroyl)spermidine | 3 | 241553 | 245856 | 272218 | 288879 | 184445 | 226102 | 141572 | 126517 | 170687 | 176672 | 208570 | 216581 |
| 462 | 523.18 | 291.09 | Excelsioside | 3 | 15901 | 3741.9 | 12810 | 11625 | 6144.6 | 17407 | 2E+06 | 940257 | 635544 | 327316 | 491472 | 441221 |
| 463 | 523.18 | 291.1 | Isoligustroside | 3 | 5243.3 | 10005 | 8690.6 | 14824 | 11670 | 16230 | 3E+06 | 895202 | 726405 | 346436 | 353408 | 408867 |
| 464 | 412.25 | 412.25 | 1,2,9,10-tetraethoxy-6-methyl-5,6,6a,7-tetrahydro-4H-dibenzo[de,g]quinoline | 2 | 2E+06 | 2E+06 | 3E+06 | 2E+06 | 2E+06 | 2E+06 | 1E+06 | 1E+06 | 2E+06 | 2E+06 | 2E+06 | 2E+06 |
| 465 | 525.2 | 179.07 | 7R,8S-erythro-3',4,9,9'-tetrahydroxy-3-methoxy-8-O-4'-neolignan-7-O-glucopyr-anoside, | 3 | 121725 | 79376 | 98853 | 41680 | 21029 | 42479 | 298650 | 137966 | 136059 | 74740 | 93998 | 58209 |
| 466 | 504.31 | 363.29 | LysoPE 20:3* | 3 | 183501 | 157395 | 166449 | 105630 | 108851 | 100886 | 198431 | 194610 | 206955 | 147220 | 156065 | 138402 |
| 467 | 504.31 | 363.29 | LysoPE 20:3(2n isomer)* | 3 | 162565 | 147838 | 190660 | 98300 | 103511 | 93069 | 223856 | 236375 | 217794 | 129298 | 137369 | 124188 |
| 468 | 506.32 | 184.07 | LysoPC 17:2 | 2 | 1E+06 | 1E+06 | 1E+06 | 860993 | 791073 | 729723 | 2E+06 | 2E+06 | 2E+06 | 1E+06 | 1E+06 | 1E+06 |
| 469 | 504.31 | 279.23 | 1-Linoleoyl-2-Lysophosphatidic Acid Monobutylamine Ester | 1 | 1E+06 | 2E+06 | 1E+06 | 576689 | 579519 | 587382 | 2E+06 | 2E+06 | 2E+06 | 1E+06 | 1E+06 | 1E+06 |
| 470 | 506.32 | 365.31 | LysoPE 20:2 | 3 | 222090 | 217405 | 225032 | 154050 | 166744 | 155168 | 297652 | 309059 | 283889 | 206076 | 210713 | 249137 |
| 471 | 506.32 | 365.31 | LysoPE 20:2(2n isomer) | 3 | 195005 | 225326 | 228806 | 164564 | 169879 | 146662 | 238485 | 315053 | 287350 | 208748 | 225476 | 219943 |
| 472 | 555.29 | 225 | LysoPI 16:0 | 1 | 9595.8 | 7730.6 | 5190.9 | 5342.5 | 4216.7 | 3470.1 | 11848 | 7813 | 12585 | 8141.6 | 7290.9 | 6670.4 |
| 473 | 508.34 | 184.07 | LysoPC 17:1 | 1 | 4E+06 | 4E+06 | 4E+06 | 2E+06 | 2E+06 | 2E+06 | 5E+06 | 5E+06 | 5E+06 | 3E+06 | 3E+06 | 3E+06 |
| 474 | 510.36 | 184.07 | LysoPC 17:0(2n isomer) | 1 | 3E+06 | 3E+06 | 3E+06 | 2E+06 | 2E+06 | 2E+06 | 3E+06 | 4E+06 | 4E+06 | 3E+06 | 3E+06 | 3E+06 |
| 475 | 563.15 | 353.07 | Vicenin-3 | 2 | 2E+07 | 2E+07 | 2E+07 | 1E+07 | 1E+07 | 1E+07 | 2E+07 | 2E+07 | 2E+07 | 2E+07 | 2E+07 | 2E+07 |
| 476 | 565.1552 | 367.1 | Isovitexin-8-O-xyloside | 3 | 5E+06 | 4E+06 | 5E+06 | 2E+06 | 2E+06 | 2E+06 | 4E+06 | 4E+06 | 4E+06 | 3E+06 | 3E+06 | 3E+06 |
| 477 | 565.16 | 409.09 | Isoschaftoside | 2 | 2E+06 | 2E+06 | 1E+06 | 1E+06 | 1E+06 | 758538 | 1E+06 | 1E+06 | 2E+06 | 2E+06 | 1E+06 | 1E+06 |
| 478 | 565.1552 | 433.1 | Genistein-8-C-apiosyl(1→6)glucoside | 3 | 4E+06 | 4E+06 | 5E+06 | 1E+06 | 1E+06 | 2E+06 | 4E+06 | 2E+06 | 6E+06 | 2E+06 | 5E+06 | 5E+06 |
| 479 | 565.1552 | 433.11 | Apigenin-6-C-(2''-xylosyl)glucoside | 3 | 6E+06 | 1E+07 | 5E+06 | 3E+06 | 3E+06 | 3E+06 | 6E+06 | 1E+07 | 6E+06 | 5E+06 | 5E+06 | 5E+06 |
| 480 | 565.15 | 433.11 | Isovitexin-2''-O-xyloside* | 3 | 8E+06 | 8E+06 | 8E+06 | 4E+06 | 3E+06 | 4E+06 | 7E+06 | 7E+06 | 7E+06 | 6E+06 | 6E+06 | 5E+06 |
| 481 | 565.156 | 433.1142 | Vitexin-2''-O-xyloside* | 3 | 3E+07 | 2E+07 | 3E+07 | 1E+07 | 1E+07 | 2E+07 | 2E+07 | 2E+07 | 3E+07 | 2E+07 | 2E+07 | 2E+07 |
| 482 | 581.15 | 287.06 | Kaempferol-3-O-sambubioside | 3 | 423335 | 327045 | 359895 | 231914 | 310403 | 220072 | 149749 | 150266 | 226850 | 376653 | 330208 | 145029 |
| 483 | 581.15 | 383.07 | Luteolin-6-C-arabinoside-7-O-glucoside | 3 | 863305 | 985107 | 825038 | 1E+06 | 1E+06 | 1E+06 | 792666 | 974777 | 685203 | 661072 | 722809 | 938564 |
| 484 | 581.1527 | 239.057 | Dehydroepigallocatechin 6-C-Glucoside-3'-C-arabinoside | 3 | 3E+07 | 3E+07 | 3E+07 | 3E+07 | 3E+07 | 3E+07 | 2E+07 | 2E+07 | 2E+07 | 3E+07 | 2E+07 | 3E+07 |
| 485 | 521.2 | 131.05 | Dehydrodiconiferyl alcohol-gamma'-O-glucoside* | 3 | 149766 | 157944 | 211404 | 779854 | 395784 | 235980 | 219123 | 457837 | 231860 | 249155 | 375915 | 651490 |
| 486 | 484.24 | 220.1 | N1-Caffeoyl-N10-Feruloylspermidine | 3 | 257453 | 251642 | 295149 | 120406 | 106027 | 100530 | 131190 | 138021 | 219668 | 171323 | 167888 | 97929 |
| 487 | 521.2 | 89.02 | Urolignoside | 2 | 345407 | 308734 | 445700 | 961575 | 420332 | 1E+06 | 681630 | 946475 | 751985 | 695030 | 800580 | 795555 |
| 488 | 459.23 | 295.22 | Vibsanin J | 3 | 521385 | 491797 | 583951 | 659011 | 797628 | 565415 | 486670 | 431909 | 434930 | 835232 | 665855 | 618529 |
| 489 | 518.32 | 184.07 | LysoPC 18:3 | 2 | 5E+06 | 5E+06 | 5E+06 | 2E+06 | 2E+06 | 2E+06 | 5E+06 | 5E+06 | 5E+06 | 5E+06 | 4E+06 | 4E+06 |
| 490 | 518.32 | 184.07 | LysoPC 18:3(2n isomer) | 1 | 3E+06 | 4E+06 | 4E+06 | 2E+06 | 3E+06 | 2E+06 | 4E+06 | 3E+06 | 4E+06 | 3E+06 | 3E+06 | 3E+06 |
| 491 | 518.32 | 184.08 | 1-18:3-LysoPC | 3 | 4E+06 | 3E+06 | 3E+06 | 2E+06 | 3E+06 | 2E+06 | 3E+06 | 3E+06 | 4E+06 | 3E+06 | 3E+06 | 3E+06 |
| 492 | 520.34 | 184.07 | LysoPC 18:2(2n isomer) | 2 | 5E+06 | 5E+06 | 6E+06 | 2E+06 | 2E+06 | 2E+06 | 4E+06 | 6E+06 | 6E+06 | 4E+06 | 4E+06 | 4E+06 |
| 493 | 520.34 | 184.08 | 1-(9Z,12Z-Octadecadienoyl)-Sn-Glycero-3-Phosphocholine | 3 | 4E+06 | 4E+06 | 4E+06 | 3E+06 | 2E+06 | 2E+06 | 3E+06 | 4E+06 | 4E+06 | 3E+06 | 3E+06 | 3E+06 |
| 494 | 522.35 | 184.07 | 1-Vaccenoyl-Glycero-3-Phosphocholine | 3 | 2E+07 | 2E+07 | 2E+07 | 7E+06 | 6E+06 | 7E+06 | 3E+07 | 3E+07 | 3E+07 | 2E+07 | 2E+07 | 2E+07 |
| 495 | 522.36 | 184.07 | LysoPC 18:1* | 1 | 3E+07 | 3E+07 | 3E+07 | 1E+07 | 1E+07 | 1E+07 | 4E+07 | 4E+07 | 4E+07 | 2E+07 | 2E+07 | 2E+07 |
| 496 | 522.36 | 184.07 | LysoPC 18:1(2n isomer)* | 1 | 3E+07 | 2E+07 | 3E+07 | 1E+07 | 1E+07 | 1E+07 | 2E+07 | 2E+07 | 2E+07 | 2E+07 | 2E+07 | 2E+07 |
| 497 | 552.33 | 253.22 | 1-(2,3-dihydroxypropoxy)-3-(((2-(dimethylamino)ethoxy)(hydroxy)phosphoryl)oxy)propan-2-yl (E)-hexadec-9-enoate | 1 | 360862 | 438833 | 125567 | 46262 | 269355 | 78218 | 592872 | 556279 | 520471 | 137467 | 174528 | 147032 |
| 498 | 552.33 | 253.22 | 2-(2,3-dihydroxypropoxy)-3-(((2-(dimethylamino)ethoxy)(hydroxy)phosphoryl)oxy)propan-2-yl (E)-hexadec-9-enoate | 2 | 443705 | 506354 | 509571 | 263723 | 202795 | 236873 | 686560 | 584547 | 598891 | 410784 | 489426 | 413454 |
| 499 | 524.37 | 184.07 | LysoPC 18:0 | 1 | 2E+07 | 2E+07 | 2E+07 | 1E+07 | 1E+07 | 1E+07 | 2E+07 | 3E+07 | 2E+07 | 2E+07 | 2E+07 | 2E+07 |
| 500 | 524.37 | 184.07 | LysoPC 18:0(2n isomer) | 1 | 3E+06 | 3E+06 | 3E+06 | 2E+06 | 2E+06 | 2E+06 | 3E+06 | 5E+06 | 3E+06 | 3E+06 | 3E+06 | 3E+06 |
| 501 | 633.07 | 301 | Phyllanemblinin B | 3 | 2E+06 | 2E+06 | 3E+06 | 2E+06 | 2E+06 | 2E+06 | 3E+06 | 3E+06 | 3E+06 | 3E+06 | 3E+06 | 3E+06 |
| 502 | 541.13 | 313.06 | Resveratrol-4'-O-β-D-(6''-O-galloyl)-glucopyranoside | 3 | 80293 | 59032 | 93030 | 113842 | 171775 | 140829 | 178444 | 171273 | 169486 | 119158 | 98198 | 140317 |
| 503 | 557.13 | 405.1 | 2,3,5,4'-Tetrahydroxystilbene-2-O-(2'-gallic acyl)-D-glucoside | 2 | 125866 | 131215 | 174445 | 127658 | 153420 | 76524 | 378840 | 313685 | 181384 | 213500 | 173707 | 157146 |
| 504 | 589.12 | 437.11 | 4',6'-O-Digalloylsalicin | 2 | 2E+06 | 2E+06 | 2E+06 | 1E+06 | 1E+06 | 1E+06 | 2E+06 | 2E+06 | 2E+06 | 2E+06 | 2E+06 | 2E+06 |
| 505 | 579.17 | 433.11 | Vitexin-2''-O-rhamnoside | 1 | 2E+06 | 3E+06 | 3E+06 | 975543 | 1E+06 | 933604 | 3E+06 | 3E+06 | 2E+06 | 2E+06 | 1E+06 | 2E+06 |
| 506 | 595.1663 | 271.0617 | Apigenin-7-O-Gentiobioside | 3 | 1E+07 | 1E+07 | 1E+07 | 5E+06 | 5E+06 | 5E+06 | 1E+07 | 1E+07 | 1E+07 | 9E+06 | 1E+07 | 1E+07 |
| 507 | 593.15 | 285.04 | Kaempferol-3-O-robinobioside(Biorobin)* | 1 | 3E+06 | 3E+06 | 4E+06 | 3E+06 | 2E+06 | 3E+06 | 2E+06 | 2E+06 | 2E+06 | 3E+06 | 3E+06 | 3E+06 |
| 508 | 593.15 | 285.04 | Kaempferol-3-O-rutinoside(Nicotiflorin)* | 1 | 4E+07 | 3E+07 | 3E+07 | 2E+07 | 2E+07 | 3E+07 | 2E+07 | 2E+07 | 2E+07 | 2E+07 | 2E+07 | 2E+07 |
| 509 | 595.16 | 287.05 | Kaempferol-3-O-rhamnosyl(1→2)glucoside* | 1 | 3E+06 | 2E+06 | 3E+06 | 4E+06 | 4E+06 | 5E+06 | 3E+06 | 2E+06 | 2E+06 | 2E+06 | 2E+06 | 2E+06 |
| 510 | 595.166 | 287.0598 | Kaempferol-3-O-glucorhamnoside* | 1 | 5E+07 | 5E+07 | 7E+07 | 1E+07 | 3E+07 | 3E+07 | 9 | 9 | 9 | 4E+07 | 4E+07 | 6E+07 |
| 511 | 595.17 | 287.06 | Cimicifugic acid E-glucose | 1 | 2E+06 | 2E+06 | 2E+06 | 3E+06 | 2E+06 | 3E+06 | 2E+06 | 1E+06 | 1E+06 | 5E+06 | 2E+06 | 3E+06 |
| 512 | 595.16 | 313.07 | Vitexin-2''-O-glucoside | 1 | 4E+07 | 5E+07 | 5E+07 | 2E+07 | 2E+07 | 2E+07 | 4E+07 | 4E+07 | 5E+07 | 3E+07 | 4E+07 | 3E+07 |
| 513 | 595.17 | 313.07 | Isosaponarin(Isovitexin-4'-O-glucoside) | 1 | 3E+07 | 4E+07 | 4E+07 | 2E+07 | 2E+07 | 2E+07 | 4E+07 | 4E+07 | 4E+07 | 4E+07 | 3E+07 | 3E+07 |
| 514 | 595.17 | 433.11 | Isovitexin-7-O-glucoside(Saponarin)* | 1 | 2E+07 | 3E+07 | 3E+07 | 1E+07 | 1E+07 | 1E+07 | 3E+07 | 3E+07 | 3E+07 | 2E+07 | 2E+07 | 2E+07 |
| 515 | 595.17 | 433.12 | Vitexin-2''-O-galactoside* | 1 | 4E+06 | 3E+06 | 4E+06 | 2E+06 | 1E+06 | 2E+06 | 3E+06 | 3E+06 | 4E+06 | 3E+06 | 3E+06 | 3E+06 |
| 516 | 595.17 | 433.12 | 4'-O-Glucosylvitexin | 3 | 4E+06 | 3E+06 | 3E+06 | 1E+06 | 1E+06 | 2E+06 | 3E+06 | 3E+06 | 3E+06 | 3E+06 | 3E+06 | 3E+06 |
| 517 | 595.16 | 449.11 | Orientin-2''-O-rhamnoside | 2 | 3E+06 | 3E+06 | 3E+06 | 1E+06 | 1E+06 | 1E+06 | 3E+06 | 3E+06 | 3E+06 | 2E+06 | 2E+06 | 2E+06 |
| 518 | 595.17 | 449.11 | Luteolin-6-C-glucoside-7-O-rhamnoside | 3 | 3E+06 | 2E+06 | 3E+06 | 1E+06 | 1E+06 | 1E+06 | 3E+06 | 3E+06 | 3E+06 | 2E+06 | 2E+06 | 2E+06 |
| 519 | 595.16 | 577.16 | Vitexin-7-O-glucoside | 2 | 2E+06 | 2E+06 | 2E+06 | 2E+06 | 3E+06 | 2E+06 | 1E+06 | 1E+06 | 2E+06 | 2E+06 | 2E+06 | 2E+06 |
| 520 | 611.16 | 287.05 | Kaempferol-3,7-O-diglucoside | 3 | 221692 | 312622 | 329624 | 159248 | 176813 | 132168 | 261889 | 305748 | 269117 | 276841 | 270135 | 236108 |
| 521 | 611.1607 | 449.107 | Orientin-2''-O-galactoside | 3 | 2E+06 | 3E+06 | 2E+06 | 968412 | 1E+06 | 1E+06 | 3E+06 | 3E+06 | 2E+06 | 1E+06 | 2E+06 | 1E+06 |
| 522 | 611.16 | 491.12 | Kaempferol-6,8-di-C-glucoside | 3 | 242663 | 168339 | 217107 | 164442 | 119580 | 174315 | 214551 | 236402 | 243626 | 158933 | 156117 | 195882 |
| 523 | 627.16 | 303.05 | 6-Hydroxykaempferol-6,7-O-Diglucoside* | 2 | 178450 | 248555 | 122077 | 529320 | 146030 | 229185 | 362580 | 641445 | 327826 | 198277 | 168736 | 251373 |
| 524 | 627.16 | 303.06 | Quercetin-3-O-sophoroside (Baimaside) | 2 | 706958 | 423442 | 502037 | 181790 | 298593 | 458239 | 1E+06 | 542705 | 476022 | 295200 | 213500 | 298650 |
| 525 | 611.16 | 371.08 | Eriodictyol-8-C-glucoside-4'-O-glucoside | 1 | 729729 | 811473 | 777681 | 792491 | 834238 | 745054 | 486989 | 417610 | 472231 | 693273 | 531425 | 746794 |
| 526 | 599.2 | 431.13 | Phloretin 3',5'-Di-C-Glucoside | 1 | 293230 | 305950 | 477895 | 507994 | 419983 | 620769 | 836528 | 1E+06 | 435474 | 258565 | 435648 | 401606 |
| 527 | 515.32 | 279.23 | 1-O-Linoleoyl-3-O-galactopyranosyl-L-glycerol | 1 | 212167 | 225166 | 96052 | 34525 | 42362 | 44903 | 398768 | 377427 | 195279 | 268202 | 154755 | 235784 |
| 528 | 534.36 | 184.07 | LysoPC 19:2(2n isomer) | 2 | 1E+06 | 1E+07 | 1E+07 | 980423 | 992711 | 1E+06 | 1E+07 | 2E+06 | 1E+07 | 8E+06 | 9E+06 | 8E+06 |
| 529 | 536.37 | 184.07 | LysoPC 19:1 | 2 | 2E+06 | 2E+06 | 2E+06 | 1E+06 | 1E+06 | 1E+06 | 2E+06 | 2E+06 | 2E+06 | 1E+06 | 2E+06 | 2E+06 |
| 530 | 615.1 | 153.02 | Kaempferol-3-O-(2''-O-galloyl)glucuronide | 3 | 16700 | 21561 | 31388 | 58756 | 50015 | 53389 | 68482 | 40442 | 75670 | 45329 | 64182 | 43345 |
| 531 | 601.12 | 287.06 | Kaempferol-3-O-(2''-galloyl)galactoside* | 1 | 9E+06 | 1E+07 | 7E+06 | 2E+07 | 1E+07 | 7E+07 | 3E+07 | 2E+07 | 4E+07 | 1E+07 | 2E+07 | 2E+07 |
| 532 | 601.12 | 287.06 | Kaempferol-3-O-(6''-galloyl)galactoside* | 1 | 9E+06 | 1E+07 | 7E+06 | 2E+07 | 1E+07 | 7E+07 | 3E+07 | 2E+07 | 4E+07 | 1E+07 | 2E+07 | 2E+07 |
| 533 | 593.18 | 447.13 | Chrysoeriol-6-C-rhamnoside-7-O-rhamnoside | 3 | 82950 | 81033 | 49451 | 27841 | 21717 | 43360 | 76136 | 58304 | 42271 | 56728 | 69023 | 30267 |
| 534 | 625.17 | 301.07 | Chrysoeriol-5,7-di-O-glucoside | 3 | 88109 | 137998 | 75892 | 25681 | 55015 | 47178 | 85655 | 97990 | 95256 | 57611 | 85030 | 77808 |
| 535 | 625.18 | 317.06 | 6-C-Methylquercetin-3-O-rutinoside | 3 | 715155 | 687710 | 642067 | 336998 | 295398 | 379017 | 379875 | 314933 | 327105 | 374294 | 417910 | 691677 |
| 536 | 625.18 | 317.07 | Isorhamnetin-3-O-glucoside-7-O-rhamnoside | 3 | 819021 | 757235 | 684955 | 278731 | 365684 | 395978 | 552215 | 451465 | 492791 | 366531 | 439104 | 504162 |
| 537 | 625.18 | 463.13 | Chrysoeriol-6-C-glucoside-4'-O-glucoside | 3 | 570468 | 627239 | 489919 | 365773 | 493601 | 155090 | 905839 | 703431 | 644719 | 365475 | 439858 | 607779 |
| 538 | 625.18 | 487.12 | Chrysoeriol-6,8-di-C-glucoside | 3 | 161222 | 131735 | 194734 | 9 | 9 | 9 | 229336 | 218883 | 229517 | 103375 | 181245 | 119131 |
| 539 | 627.19 | 465.1 | Hesperetin-8-C-glucoside-3'-O-glucoside | 3 | 9 | 9 | 9 | 321701 | 225175 | 394846 | 352257 | 306397 | 362985 | 223901 | 225151 | 323383 |
| 540 | 627.19 | 465.2 | Hesperetin-6-C-glucoside-7-O-glucoside | 3 | 158425 | 71690 | 126730 | 195099 | 116720 | 304634 | 221396 | 267416 | 316239 | 220175 | 97324 | 150434 |
| 541 | 581.22 | 329.14 | Tortoside B | 2 | 190195 | 180427 | 219986 | 161088 | 213433 | 258672 | 119641 | 137306 | 135365 | 147691 | 174287 | 138617 |
| 542 | 546.36 | 184.07 | LysoPC 20:3 | 2 | 2E+06 | 2E+06 | 2E+06 | 1E+06 | 1E+06 | 1E+06 | 2E+06 | 2E+06 | 2E+06 | 1E+06 | 2E+06 | 2E+06 |
| 543 | 576.33 | 277.22 | 2-(2,3-dihydroxypropoxy)-3-(((2-(dimethylamino)ethoxy)(hydroxy)phosphoryl)oxy)propyl (8E,11Z,14Z)-octadeca-8,11,14-trienoate | 1 | 8E+06 | 9E+06 | 1E+07 | 5E+06 | 3E+06 | 3E+06 | 2E+07 | 2E+07 | 1E+07 | 8E+06 | 1E+07 | 9E+06 |
| 544 | 578.34 | 279.23 | 2-(2,3-dihydroxypropoxy)-3-(((2-(dimethylamino)ethoxy)(hydroxy)phosphoryl)oxy)propyl (11Z,14Z)-octadeca-11,14-dienoate | 1 | 9E+06 | 1E+07 | 6E+06 | 4E+06 | 4E+06 | 5E+06 | 8E+06 | 1E+07 | 1E+07 | 1E+07 | 9E+06 | 9E+06 |
| 545 | 550.39 | 184.07 | LysoPC 20:1 | 2 | 4E+06 | 4E+06 | 4E+06 | 3E+06 | 3E+06 | 3E+06 | 6E+06 | 6E+06 | 5E+06 | 3E+06 | 4E+06 | 4E+06 |
| 546 | 580.36 | 281.25 | 1-(2,3-dihydroxypropoxy)-3-(((2-(dimethylamino)ethoxy)(hydroxy)phosphoryl)oxy)propan-2-yl (Z)-14-Octadecenoic Acid | 1 | 491135 | 323036 | 411346 | 187303 | 219434 | 179520 | 322313 | 305626 | 264377 | 378835 | 447648 | 255092 |
| 547 | 580.36 | 281.25 | 2-(2,3-dihydroxypropoxy)-3-(((2-(dimethylamino)ethoxy)(hydroxy)phosphoryl)oxy)propan-2-yl (Z)-14-Octadecenoic Acid | 1 | 1E+06 | 684474 | 585771 | 436619 | 288167 | 499060 | 1E+06 | 1E+06 | 936801 | 1E+06 | 1E+06 | 947431 |
| 548 | 593.09 | 289.07 | 7,3'-Di-O-gallyoltricetiflavan | 1 | 1E+07 | 1E+07 | 1E+07 | 1E+07 | 1E+07 | 1E+07 | 2E+07 | 2E+07 | 2E+07 | 1E+07 | 1E+07 | 1E+07 |
| 549 | 593.09 | 289.07 | 7,4'-Di-O-galloyltricetiflavan | 1 | 1E+07 | 1E+07 | 1E+07 | 1E+07 | 2E+07 | 2E+07 | 2E+07 | 2E+07 | 2E+07 | 2E+07 | 2E+07 | 2E+07 |
| 550 | 593.0956 | 441.0835 | Epicatechin 3,5-Digallate* | 3 | 3E+07 | 3E+07 | 4E+07 | 4E+07 | 4E+07 | 4E+07 | 5E+07 | 5E+07 | 5E+07 | 4E+07 | 4E+07 | 4E+07 |
| 551 | 623.2 | 161.02 | Magnoloside D | 1 | 11670 | 6670 | 16675 | 6670 | 11670 | 30002 | 6E+06 | 3E+06 | 3E+06 | 1E+06 | 1E+06 | 1E+06 |
| 552 | 623.2 | 161.02 | Forsythoside H* | 3 | 9 | 9 | 9 | 15005 | 13340 | 9359.3 | 6E+06 | 3E+06 | 3E+06 | 1E+06 | 1E+06 | 1E+06 |
| 553 | 623.2 | 161.03 | Forsythoside I* | 3 | 8838 | 5005 | 20752 | 14064 | 16675 | 15005 | 5E+06 | 3E+06 | 3E+06 | 1E+06 | 1E+06 | 1E+06 |
| 554 | 623.2 | 461.17 | Acteoside; Verbascoside | 2 | 13291 | 15005 | 7603.8 | 6013 | 15005 | 11079 | 202380 | 148340 | 125534 | 58323 | 47984 | 63489 |
| 555 | 88.99 | 70.98 | Oxalic acid | 3 | 66075 | 86941 | 69088 | 33817 | 28541 | 43696 | 45273 | 44808 | 54202 | 60404 | 63372 | 66570 |
| 556 | 125 | 78.96 | 2-Hydroxyethylphosphonic acid | 3 | 326932 | 335653 | 259429 | 394268 | 309823 | 326685 | 514292 | 483866 | 495685 | 440660 | 469788 | 435032 |
| 557 | 140.01 | 78.96 | O-Phosphorylethanolamine | 3 | 512138 | 555557 | 575258 | 820671 | 827693 | 806969 | 634021 | 581871 | 583096 | 657986 | 621022 | 649027 |
| 558 | 623.107 | 169.0139 | Epigallocatechin 3-O-gallate 3'-O-(3-O-Methyl)Gallate | 3 | 3E+06 | 3E+06 | 2E+06 | 2E+06 | 2E+06 | 2E+06 | 2E+06 | 3E+06 | 2E+06 | 2E+06 | 2E+06 | 2E+06 |
| 559 | 623.1053 | 183.0282 | Epigallocatechin 3'-O-gallate 3-O-(3-O-Methyl)Gallate | 3 | 303719 | 276633 | 293337 | 184287 | 183465 | 166457 | 266847 | 281020 | 234898 | 230427 | 234044 | 215237 |
| 560 | 579.15 | 271.06 | Apigenin-7-O-(6''-p-Coumaryl)glucoside | 3 | 3E+07 | 2E+07 | 2E+07 | 1E+07 | 1E+07 | 2E+07 | 1E+07 | 1E+07 | 1E+07 | 1E+07 | 1E+07 | 1E+07 |
| 561 | 577.14 | 407.08 | Procyanidin B2 | 1 | 1E+07 | 1E+07 | 1E+07 | 8E+06 | 1E+07 | 1E+07 | 2E+07 | 1E+07 | 1E+07 | 1E+07 | 1E+07 | 1E+07 |
| 562 | 577.14 | 407.08 | Procyanidin B3 | 1 | 1E+07 | 1E+07 | 1E+07 | 1E+07 | 9E+06 | 1E+07 | 1E+07 | 1E+07 | 2E+07 | 1E+07 | 1E+07 | 1E+07 |
| 563 | 577.14 | 407.08 | Procyanidin B4 | 3 | 2E+06 | 2E+06 | 2E+06 | 2E+06 | 2E+06 | 2E+06 | 3E+06 | 3E+06 | 3E+06 | 3E+06 | 2E+06 | 3E+06 |
| 564 | 593.13 | 300.03 | Quercetin 7-O-p-coumaroyl rhamnoside | 1 | 107667 | 81482 | 89611 | 54215 | 72605 | 69204 | 104209 | 100846 | 86343 | 84739 | 83372 | 74395 |
| 565 | 611.14 | 147.04 | Quercetin-3-O-(6''-O-p-Coumaroyl)galactoside | 1 | 3E+06 | 3E+06 | 3E+06 | 1E+06 | 2E+06 | 2E+06 | 3E+06 | 3E+06 | 2E+06 | 3E+06 | 2E+06 | 1E+06 |
| 566 | 611.14 | 287.05 | Gallocatechin-(4α→8)-gallocatechin | 3 | 287696 | 328375 | 272837 | 263580 | 166068 | 216317 | 312760 | 350435 | 328661 | 253154 | 221866 | 343745 |
| 567 | 565.17 | 337.1 | Ferulic-piceid | 3 | 100742 | 93871 | 108539 | 67913 | 89250 | 73481 | 98248 | 104471 | 88862 | 89845 | 85225 | 88181 |
| 568 | 441.37 | 441.37 | 3-Hydroxylup-20(29)-en-28-al (Betulinaldehyde) | 2 | 2E+06 | 2E+06 | 2E+06 | 2E+06 | 2E+06 | 2E+06 | 3E+06 | 3E+06 | 3E+06 | 2E+06 | 2E+06 | 2E+06 |
| 569 | 455.35 | 455.35 | 3-Hydroxyurs-12-en-28-oic acid (Ursolic acid) | 3 | 44540 | 48821 | 38629 | 51031 | 40044 | 24147 | 357807 | 229319 | 244442 | 159705 | 124077 | 107911 |
| 570 | 471.35 | 471.34 | 16,23:16,30-Diepoxydammar-24-ene-3,20-diol (Jujubogenin) | 2 | 149673 | 232795 | 213983 | 225325 | 242909 | 236534 | 2E+06 | 2E+06 | 2E+06 | 1E+06 | 873713 | 997042 |
| 571 | 471.35 | 471.34 | 2,3-Dihydroxy-12-ursen-28-oic acid | 2 | 149673 | 232795 | 213983 | 225325 | 242909 | 236534 | 2E+06 | 2E+06 | 2E+06 | 1E+06 | 873713 | 997042 |
| 572 | 585.24 | 537.21 | 23-Hydroxytoonacilide | 3 | 1E+06 | 1E+06 | 1E+06 | 748073 | 828666 | 742188 | 943936 | 907264 | 930463 | 1E+06 | 929865 | 939441 |
| 573 | 585.23 | 537.21 | 7,8-Dihydro-Buddlenol B(threo) | 1 | 994989 | 1E+06 | 1E+06 | 695199 | 797207 | 763830 | 892232 | 898422 | 948424 | 922100 | 891898 | 910521 |
| 574 | 585.23 | 537.21 | Dihydrobuddlenol B | 1 | 1E+06 | 1E+06 | 1E+06 | 352510 | 773184 | 372959 | 489045 | 966987 | 951994 | 476101 | 553186 | 519399 |
| 575 | 675.28 | 163.04 | inerminoside A | 2 | 920217 | 755350 | 1E+06 | 201941 | 231891 | 301210 | 675815 | 412374 | 351719 | 346835 | 428417 | 405248 |
| 576 | 653.38 | 397.14 | 1-Palmitoyl-Sn-Glycerol 3-O-Diglucoside | 2 | 2E+06 | 2E+06 | 2E+06 | 716805 | 701544 | 934337 | 3E+06 | 2E+06 | 3E+06 | 2E+06 | 2E+06 | 2E+06 |
| 577 | 653.38 | 397.14 | 2-Palmitoyl-Sn-Glycerol 3-O-Diglucoside | 2 | 2E+06 | 2E+06 | 2E+06 | 906735 | 712698 | 752496 | 2E+06 | 3E+06 | 3E+06 | 2E+06 | 2E+06 | 2E+06 |
| 578 | 517.39 | 425.34 | 3-(Acetyloxy)-13,15-dihydroxyoleanan-12-one (Rubiprasin A)(iso-01) | 3 | 156306 | 154781 | 157075 | 130231 | 140592 | 144653 | 114361 | 121487 | 102171 | 154536 | 127146 | 132775 |
| 579 | 797.14 | 645.1 | 2-O-Trigalloyl-glucose-glucose | 3 | 292463 | 250063 | 136735 | 9 | 9 | 9 | 304662 | 363738 | 266885 | 267753 | 247547 | 321850 |
| 580 | 741.22 | 287.06 | Kaempferol-3-O-rutinoside-7-O-rhamnoside | 2 | 216615 | 198699 | 160550 | 144558 | 235420 | 191539 | 9 | 9 | 9 | 119968 | 73355 | 187943 |
| 581 | 741.23 | 433.12 | Luteolin-7-O-(2''-O-rhamnosyl)rutinoside | 2 | 398005 | 253107 | 392005 | 605704 | 504195 | 412306 | 265595 | 325373 | 198667 | 171948 | 158848 | 213442 |
| 582 | 757.22 | 577.2 | Apigenin-6,8-di-C-glucoside-4'-O-glucoside | 3 | 201858 | 83411 | 129580 | 103606 | 82799 | 180305 | 84002 | 62989 | 59093 | 85743 | 77984 | 69668 |
| 583 | 773.21 | 303.05 | Quercetin-3-O-sophoroside-7-O-rhamnoside | 3 | 80410 | 53350 | 33340 | 53350 | 25005 | 43678 | 14512 | 20005 | 38266 | 30010 | 25982 | 18340 |
| 584 | 575.35 | 279.2 | Dendrocrepine* | 1 | 320813 | 316299 | 667307 | 148438 | 79002 | 143808 | 385042 | 359559 | 223908 | 369052 | 294293 | 126915 |
| 585 | 575.35 | 279.2 | Isodendrocrepine* | 1 | 275556 | 240312 | 696665 | 36658 | 124373 | 245280 | 442160 | 331717 | 315092 | 288322 | 169173 | 322203 |
| 586 | 677.37 | 261.22 | 1-α-Linolenoyl-glycerol-2,3-di-O-glucoside | 2 | 40901 | 36676 | 28267 | 13955 | 9430.8 | 7687.4 | 68370 | 45344 | 59680 | 27784 | 25719 | 28343 |
| 587 | 677.37 | 261.22 | 2-α-Linolenoyl-glycerol-1,3-di-O-glucoside | 1 | 28620 | 24204 | 25802 | 9282.9 | 4387 | 6651.9 | 53525 | 51207 | 35023 | 38290 | 32291 | 22598 |
| 588 | 675.36 | 397.14 | Gingerglycolipid A | 3 | 4E+06 | 4E+06 | 6E+06 | 904282 | 2E+06 | 2E+06 | 8E+06 | 7E+06 | 6E+06 | 5E+06 | 6E+06 | 5E+06 |
| 589 | 677.38 | 397.13 | Gingerglycolipid B | 3 | 925534 | 1E+06 | 1E+06 | 202355 | 182748 | 200939 | 1E+06 | 1E+06 | 1E+06 | 907089 | 727237 | 720875 |
| 590 | 679.39 | 397.13 | Gingerglycolipid C | 3 | 356632 | 175700 | 130310 | 114374 | 108261 | 95222 | 509213 | 528021 | 556298 | 316423 | 231377 | 335930 |
| 591 | 584.28 | 438.24 | Tri-p-coumaroyl Spermidine | 3 | 2E+06 | 2E+06 | 2E+06 | 2E+06 | 2E+06 | 2E+06 | 2E+06 | 2E+06 | 2E+06 | 2E+06 | 2E+06 | 2E+06 |
| 592 | 717.145 | 579.1133 | Theaflavin-3'-Gallate* | 1 | 7E+06 | 8E+06 | 8E+06 | 8E+06 | 6E+06 | 7E+06 | 4E+06 | 5E+06 | 5E+06 | 5E+06 | 7E+06 | 4E+06 |
| 593 | 729.14 | 407.07 | 3-O-galloylprocyanidin B1 | 1 | 8E+06 | 8E+06 | 9E+06 | 9E+06 | 9E+06 | 9E+06 | 1E+07 | 1E+07 | 1E+07 | 1E+07 | 1E+07 | 1E+07 |
| 594 | 729.15 | 407.08 | Procyanidin B2 3'-O-Gallate | 1 | 8E+06 | 9E+06 | 9E+06 | 9E+06 | 9E+06 | 9E+06 | 1E+07 | 1E+07 | 9E+06 | 1E+07 | 1E+07 | 9E+06 |
| 595 | 787.21 | 339.11 | Kaempferol-3-O-(6''-Feruloyl)glucosyl-(1→4)-galactoside | 3 | 77505 | 103007 | 110976 | 35186 | 30832 | 21675 | 40010 | 31675 | 31675 | 95035 | 45411 | 58964 |
| 596 | 803.2 | 339.11 | Quercetin-3-O-(2'''-O-feruloyl)sophoroside | 3 | 59597 | 61685 | 46703 | 15005 | 58350 | 13143 | 24774 | 15566 | 25005 | 24526 | 51106 | 33340 |
| 597 | 699.35 | 699.35 | 10-Hydroxymethyllycaconitine | 1 | 8E+06 | 8E+06 | 8E+06 | 2E+06 | 2E+06 | 2E+06 | 1E+07 | 1E+07 | 9E+06 | 7E+06 | 7E+06 | 7E+06 |
| 598 | 701.37 | 701.37 | Puberaconitidine | 2 | 4E+07 | 4E+07 | 4E+07 | 8E+06 | 8E+06 | 8E+06 | 4E+07 | 4E+07 | 4E+07 | 3E+07 | 3E+07 | 3E+07 |
| 599 | 103 | 59.01 | Malonic acid | 3 | 4E+06 | 4E+06 | 4E+06 | 3E+06 | 3E+06 | 3E+06 | 5E+06 | 5E+06 | 5E+06 | 4E+06 | 3E+06 | 4E+06 |
| 600 | 103 | 59.01 | Hydroxypyruvic acid | 3 | 506102 | 524759 | 535971 | 403683 | 415204 | 402577 | 615962 | 582506 | 578419 | 508463 | 456631 | 473644 |
| 601 | 118.01 | 74.02 | Aminomalonic acid | 3 | 4E+06 | 4E+06 | 4E+06 | 3E+06 | 3E+06 | 4E+06 | 4E+06 | 4E+06 | 4E+06 | 4E+06 | 4E+06 | 3E+06 |
| 602 | 89.02 | 59.01 | 3-Hydroxypropanoic acid | 3 | 65339 | 79195 | 203398 | 39305 | 38728 | 38738 | 71742 | 60107 | 100563 | 67608 | 81908 | 60116 |
| 603 | 88.04 | 88.04 | N-Methylglycine | 3 | 930356 | 952506 | 951509 | 951718 | 886753 | 895091 | 743274 | 750729 | 752049 | 622460 | 824250 | 744924 |
| 604 | 903.26 | 147.04 | Vitexin-2''-O-(6'''-p-coumaroyl)glucoside-4'-O-glucoside | 2 | 5005 | 14983 | 15005 | 58350 | 23340 | 68355 | 25005 | 45010 | 33340 | 21675 | 44945 | 26832 |
| 605 | 849.1703 | 697.1596 | Epiafzelechin-(4b->8)-Epiafzelechin 3,3'-Digallate | 3 | 4E+06 | 4E+06 | 4E+06 | 8E+06 | 8E+06 | 7E+06 | 7E+06 | 8E+06 | 8E+06 | 7E+06 | 6E+06 | 6E+06 |
| 606 | 89.11 | 72.08 | Putrescine | 3 | 216975 | 205834 | 237381 | 194813 | 193742 | 190645 | 217239 | 234243 | 220704 | 210950 | 209732 | 192131 |
| 607 | 112.05 | 70.03 | Isocytosine | 2 | 2E+06 | 2E+06 | 2E+06 | 2E+06 | 2E+06 | 2E+06 | 2E+06 | 2E+06 | 2E+06 | 2E+06 | 2E+06 | 2E+06 |
| 608 | 117.0193 | 73.0295 | Methylmalonic acid* | 1 | 5E+06 | 5E+06 | 5E+06 | 5E+06 | 5E+06 | 5E+06 | 5E+06 | 6E+06 | 6E+06 | 5E+06 | 5E+06 | 5E+06 |
| 609 | 117.02 | 73.03 | D-Erythronolactone | 3 | 2E+06 | 2E+06 | 2E+06 | 2E+06 | 2E+06 | 1E+06 | 2E+06 | 2E+06 | 2E+06 | 2E+06 | 2E+06 | 2E+06 |
| 610 | 117.02 | 73.03 | Succinic acid* | 1 | 5E+06 | 5E+06 | 5E+06 | 4E+06 | 5E+06 | 5E+06 | 5E+06 | 6E+06 | 5E+06 | 5E+06 | 4E+06 | 5E+06 |
| 611 | 102.06 | 56.05 | 1-Aminocyclopropane-1-carboxylic acid | 2 | 865445 | 854603 | 857181 | 1E+06 | 1E+06 | 1E+06 | 947834 | 901200 | 922728 | 858862 | 915282 | 849999 |
| 612 | 102.05 | 56.05 | L-Azetidine-2-carboxylic acid | 2 | 2E+06 | 2E+06 | 2E+06 | 3E+06 | 2E+06 | 3E+06 | 2E+06 | 2E+06 | 2E+06 | 2E+06 | 2E+06 | 2E+06 |
| 613 | 132.03 | 88.04 | L-Aspartic Acid | 1 | 2E+07 | 2E+07 | 2E+07 | 3E+07 | 3E+07 | 3E+07 | 2E+07 | 2E+07 | 2E+07 | 2E+07 | 2E+07 | 2E+07 |
| 614 | 132.0303 | 88.0403 | Iminodiacetic acid | 3 | 2E+06 | 2E+06 | 2E+06 | 3E+06 | 3E+06 | 3E+06 | 2E+06 | 3E+06 | 2E+06 | 3E+06 | 3E+06 | 3E+06 |
| 615 | 131.05 | 70.03 | 3-Ureidopropionic Acid | 1 | 2E+06 | 2E+06 | 2E+06 | 2E+06 | 2E+06 | 2E+06 | 2E+06 | 2E+06 | 2E+06 | 2E+06 | 2E+06 | 2E+06 |
| 616 | 133.06 | 74.02 | L-Asparagine | 1 | 1E+07 | 1E+07 | 1E+07 | 1E+07 | 1E+07 | 1E+07 | 1E+07 | 1E+07 | 1E+07 | 1E+07 | 1E+07 | 1E+07 |
| 617 | 103.04 | 59.01 | 3-Hydroxybutyric acid | 3 | 3E+06 | 3E+06 | 3E+06 | 2E+06 | 2E+06 | 2E+06 | 4E+06 | 4E+06 | 4E+06 | 2E+06 | 2E+06 | 3E+06 |
| 618 | 121.05 | 93.06 | D-Threose | 3 | 935496 | 959428 | 1E+06 | 504516 | 520179 | 378277 | 552176 | 471033 | 949947 | 671887 | 504828 | 670234 |
| 619 | 135.03 | 75.01 | D-Threonic Acid | 1 | 5E+06 | 5E+06 | 4E+06 | 3E+06 | 3E+06 | 3E+06 | 5E+06 | 4E+06 | 5E+06 | 4E+06 | 4E+06 | 4E+06 |
| 620 | 104.07 | 58.07 | 2-Aminoisobutyric acid | 2 | 5E+06 | 5E+06 | 5E+06 | 5E+06 | 5E+06 | 5E+06 | 5E+06 | 5E+06 | 5E+06 | 4E+06 | 5E+06 | 5E+06 |
| 621 | 104.07 | 59.01 | Methyl 3-aminopropanoate | 2 | 3E+07 | 3E+07 | 3E+07 | 4E+07 | 3E+07 | 3E+07 | 3E+07 | 3E+07 | 3E+07 | 3E+07 | 3E+07 | 3E+07 |
| 622 | 104.07 | 69.03 | γ-Aminobutyric acid | 3 | 5E+06 | 6E+06 | 6E+06 | 8E+06 | 8E+06 | 8E+06 | 6E+06 | 6E+06 | 6E+06 | 6E+06 | 7E+06 | 6E+06 |
| 623 | 147.08 | 84.04 | L-Glutamine | 1 | 853131 | 873245 | 795008 | 967549 | 944118 | 915990 | 649934 | 634395 | 603801 | 926108 | 791230 | 735544 |
| 624 | 149.05 | 75.01 | D-Ribose | 3 | 886589 | 895434 | 887328 | 1E+06 | 1E+06 | 1E+06 | 1E+06 | 1E+06 | 948205 | 1E+06 | 1E+06 | 1E+06 |
| 625 | 146.09 | 87.04 | 4-Guanidinobutyric acid | 2 | 2E+07 | 2E+07 | 2E+07 | 2E+07 | 2E+07 | 2E+07 | 3E+07 | 3E+07 | 3E+07 | 2E+07 | 2E+07 | 2E+07 |
| 626 | 150.06 | 61.01 | DL-Methionine | 3 | 272018 | 259829 | 255392 | 1E+06 | 1E+06 | 1E+06 | 474230 | 430616 | 466920 | 577733 | 606087 | 625268 |
| 627 | 133.1 | 116.07 | L-Ornithine | 3 | 1E+06 | 1E+06 | 2E+06 | 2E+06 | 2E+06 | 2E+06 | 1E+06 | 1E+06 | 1E+06 | 1E+06 | 2E+06 | 1E+06 |
| 628 | 103.12 | 86.1 | Cadaverine | 3 | 387925 | 373660 | 395295 | 372567 | 332607 | 344746 | 321690 | 265280 | 289604 | 352046 | 344437 | 322070 |
| 629 | 131.13 | 114.1 | Agmatine | 3 | 1E+07 | 1E+07 | 1E+07 | 9E+06 | 9E+06 | 9E+06 | 1E+07 | 1E+07 | 1E+07 | 9E+06 | 1E+07 | 1E+07 |
| 630 | 184.07 | 125 | O-Phosphocholine | 3 | 1E+07 | 1E+07 | 1E+07 | 1E+07 | 1E+07 | 1E+07 | 2E+07 | 1E+07 | 2E+07 | 1E+07 | 1E+07 | 2E+07 |
| 631 | 167.02 | 124.01 | Uric acid | 3 | 173906 | 166797 | 162850 | 123134 | 139579 | 130764 | 179201 | 177264 | 189284 | 149095 | 179825 | 129432 |
| 632 | 110.02 | 66.03 | Pyrrole-2-carboxylic acid | 3 | 380994 | 345614 | 404003 | 400634 | 386979 | 362531 | 279716 | 296624 | 281415 | 343841 | 311409 | 343170 |
| 633 | 127.05 | 81.04 | Imidazol-1-yl-acetic acid | 3 | 964964 | 1E+06 | 1E+06 | 1E+06 | 913063 | 917888 | 1E+06 | 1E+06 | 1E+06 | 913059 | 1E+06 | 1E+06 |
| 634 | 127.05 | 110.02 | Thymine | 2 | 308030 | 287053 | 234226 | 171732 | 147155 | 159148 | 223470 | 235983 | 223401 | 191007 | 213294 | 208315 |
| 635 | 131.03 | 85.03 | Itaconic acid | 2 | 4E+06 | 4E+06 | 4E+06 | 5E+06 | 5E+06 | 5E+06 | 4E+06 | 4E+06 | 4E+06 | 4E+06 | 4E+06 | 4E+06 |
| 636 | 161.01 | 73.03 | 4-Hydroxy-2-oxoglutaric acid | 3 | 1E+06 | 2E+06 | 2E+06 | 685346 | 598924 | 712864 | 927664 | 965177 | 2E+06 | 2E+06 | 2E+06 | 1E+06 |
| 637 | 128.04 | 82.03 | 5-Oxoproline | 1 | 1E+06 | 1E+06 | 1E+06 | 889856 | 909793 | 949160 | 1E+06 | 1E+06 | 1E+06 | 1E+06 | 1E+06 | 1E+06 |
| 638 | 147.0299 | 57.0344 | 3-Methylmalic acid | 3 | 2E+06 | 2E+06 | 2E+06 | 2E+06 | 2E+06 | 2E+06 | 1E+06 | 2E+06 | 2E+06 | 1E+06 | 2E+06 | 1E+06 |
| 639 | 147.03 | 87.01 | L-Citramalic acid | 2 | 2E+06 | 2E+06 | 2E+06 | 2E+06 | 2E+06 | 2E+06 | 2E+06 | 2E+06 | 2E+06 | 2E+06 | 2E+06 | 2E+06 |
| 640 | 144.08 | 84.06 | 2-Amino-4,5-dihydro-1H-imidazole-4-acetic acid | 3 | 4E+06 | 4E+06 | 4E+06 | 3E+06 | 3E+06 | 3E+06 | 4E+06 | 4E+06 | 4E+06 | 4E+06 | 4E+06 | 4E+06 |
| 641 | 116.07 | 70.06 | 3-hydroxy-1-methylpyrrolidin-2-one | 1 | 8E+06 | 8E+06 | 8E+06 | 8E+06 | 8E+06 | 8E+06 | 7E+06 | 7E+06 | 7E+06 | 8E+06 | 8E+06 | 7E+06 |
| 642 | 116.0708 | 70.0662 | 1-Amino-1-cyclobutane-carboxylic-acid | 3 | 8E+06 | 8E+06 | 8E+06 | 8E+06 | 8E+06 | 8E+06 | 7E+06 | 7E+06 | 7E+06 | 8E+06 | 8E+06 | 7E+06 |
| 643 | 132.07 | 86.06 | 5-Aminolevulinic Acid | 3 | 8E+06 | 8E+06 | 8E+06 | 8E+06 | 8E+06 | 8E+06 | 7E+06 | 7E+06 | 7E+06 | 7E+06 | 8E+06 | 7E+06 |
| 644 | 148.06 | 88.04 | O-Acetylserine | 3 | 3E+06 | 3E+06 | 3E+06 | 3E+06 | 3E+06 | 3E+06 | 2E+06 | 2E+06 | 2E+06 | 3E+06 | 3E+06 | 3E+06 |
| 645 | 162.04 | 144.03 | 4-Hydroxy-L-glutamic acid | 3 | 354607 | 405321 | 339295 | 440355 | 447197 | 391894 | 351400 | 355322 | 376725 | 372940 | 390927 | 340751 |
| 646 | 145.06 | 109.04 | 2,2-Dimethylsuccinic acid | 1 | 293849 | 299946 | 312789 | 450079 | 473095 | 455901 | 273109 | 283958 | 301459 | 359025 | 395639 | 363749 |
| 647 | 177.04 | 59.01 | D-Glucono-1,5-lactone | 3 | 1E+06 | 1E+06 | 1E+06 | 1E+06 | 1E+06 | 1E+06 | 989698 | 851399 | 934777 | 1E+06 | 892111 | 943926 |
| 648 | 130.1 | 56.05 | Pipecolic acid | 2 | 5E+07 | 5E+07 | 5E+07 | 4E+07 | 4E+07 | 4E+07 | 5E+07 | 5E+07 | 5E+07 | 4E+07 | 5E+07 | 4E+07 |
| 649 | 130.09 | 70.07 | Homoproline | 3 | 3E+06 | 2E+06 | 2E+06 | 3E+06 | 3E+06 | 3E+06 | 2E+06 | 2E+06 | 2E+06 | 3E+06 | 2E+06 | 2E+06 |
| 650 | 146.08 | 86.06 | 4-Acetamidobutyric acid | 3 | 3E+06 | 3E+06 | 3E+06 | 2E+06 | 2E+06 | 2E+06 | 3E+06 | 3E+06 | 3E+06 | 2E+06 | 2E+06 | 2E+06 |
| 651 | 144.07 | 126.06 | Allysine(6-Oxo DL-Norleucine) | 3 | 210020 | 222758 | 229567 | 333583 | 287875 | 745285 | 447375 | 504230 | 500885 | 238485 | 227989 | 230096 |
| 652 | 131.07 | 85.07 | 2-Hydroxyisocaproic acid | 3 | 2E+06 | 2E+06 | 2E+06 | 2E+06 | 2E+06 | 2E+06 | 2E+06 | 2E+06 | 2E+06 | 2E+06 | 2E+06 | 2E+06 |
| 653 | 147.07 | 59.01 | Mevalonic acid | 3 | 6E+06 | 6E+06 | 6E+06 | 3E+06 | 3E+06 | 3E+06 | 4E+06 | 4E+06 | 4E+06 | 4E+06 | 2E+06 | 4E+06 |
| 654 | 195.05 | 75.01 | Gluconic acid | 1 | 2E+07 | 2E+07 | 2E+07 | 2E+07 | 2E+07 | 2E+07 | 2E+07 | 2E+07 | 2E+07 | 2E+07 | 2E+07 | 2E+07 |
| 655 | 160.11 | 114.04 | δ-Guanidinovaleric acid | 2 | 1E+06 | 1E+06 | 1E+06 | 899100 | 905254 | 809081 | 1E+06 | 2E+06 | 1E+06 | 1E+06 | 1E+06 | 1E+06 |
| 656 | 132.1019 | 86.0964 | L-Leucine* | 1 | 9E+06 | 9E+06 | 9E+06 | 9E+06 | 8E+06 | 9E+06 | 8E+06 | 7E+06 | 8E+06 | 8E+06 | 8E+06 | 8E+06 |
| 657 | 132.1019 | 86.0964 | L-Isoleucine* | 1 | 9E+06 | 9E+06 | 9E+06 | 9E+06 | 9E+06 | 9E+06 | 8E+06 | 7E+06 | 8E+06 | 8E+06 | 8E+06 | 7E+06 |
| 658 | 132.1 | 86.1 | L-Norleucine* | 1 | 9E+06 | 9E+06 | 9E+06 | 9E+06 | 9E+06 | 1E+07 | 8E+06 | 7E+06 | 8E+06 | 8E+06 | 8E+06 | 8E+06 |
| 659 | 132.1 | 86.1 | D-Allo-Isoleucine* | 1 | 9E+06 | 9E+06 | 9E+06 | 9E+06 | 8E+06 | 9E+06 | 8E+06 | 7E+06 | 8E+06 | 7E+06 | 8E+06 | 8E+06 |
| 660 | 164.07 | 56.05 | L-Homomethionine | 3 | 427535 | 452925 | 453915 | 1E+06 | 1E+06 | 1E+06 | 601088 | 612268 | 554775 | 571489 | 618284 | 667882 |
| 661 | 131.12 | 114.09 | N-Acetylputrescine | 3 | 1E+07 | 1E+07 | 1E+07 | 1E+07 | 9E+06 | 1E+07 | 1E+07 | 1E+07 | 1E+07 | 1E+07 | 1E+07 | 1E+07 |
| 662 | 147.11 | 84.08 | L-Lysine | 1 | 707229 | 733795 | 778347 | 845727 | 876860 | 810347 | 573906 | 585345 | 604670 | 684588 | 637147 | 689326 |
| 663 | 258.04 | 78.96 | D-Glucosamine 1-phosphate | 3 | 260725 | 274226 | 242709 | 258382 | 277585 | 265326 | 355097 | 375881 | 384459 | 368143 | 286105 | 301742 |
| 664 | 165.08 | 59.02 | L-Fucitol | 2 | 7E+06 | 7E+06 | 7E+06 | 6E+06 | 6E+06 | 5E+06 | 7E+06 | 7E+06 | 7E+06 | 7E+06 | 7E+06 | 7E+06 |
| 665 | 245.04 | 153 | sn-glycero-3-phospho-(1'-sn-glycerol) | 1 | 9E+06 | 9E+06 | 1E+07 | 8E+06 | 9E+06 | 8E+06 | 1E+07 | 1E+07 | 1E+07 | 1E+07 | 1E+07 | 1E+07 |
| 666 | 122.02 | 78.03 | 2-Picolinic acid | 3 | 1E+06 | 1E+06 | 1E+06 | 2E+06 | 1E+06 | 1E+06 | 1E+06 | 1E+06 | 1E+06 | 1E+06 | 2E+06 | 1E+06 |
| 667 | 140.03 | 94.03 | 6-Hydroxynicotinic acid | 3 | 854217 | 2E+06 | 2E+06 | 953306 | 469231 | 650649 | 2E+06 | 1E+06 | 2E+06 | 2E+06 | 910729 | 1E+06 |
| 668 | 167.06 | 110.03 | 1-Methylxanthine | 3 | 238772 | 264995 | 327399 | 103408 | 64592 | 106655 | 279556 | 317142 | 294984 | 220821 | 217052 | 209725 |
| 669 | 109.03 | 81.03 | Hydroquinone | 3 | 349334 | 371656 | 354128 | 119226 | 128473 | 128056 | 229704 | 260641 | 213445 | 232585 | 237750 | 224225 |
| 670 | 125.02 | 79.02 | Methyl 2-furoate | 2 | 4E+07 | 4E+07 | 4E+07 | 3E+07 | 3E+07 | 3E+07 | 4E+07 | 4E+07 | 4E+07 | 4E+07 | 3E+07 | 4E+07 |
| 671 | 125.02 | 79.02 | Pyrogallol | 2 | 2E+06 | 2E+06 | 2E+06 | 1E+06 | 954283 | 1E+06 | 1E+06 | 1E+06 | 1E+06 | 1E+06 | 1E+06 | 1E+06 |
| 672 | 125.03 | 81.03 | 2,3-Dimethylmaleic anhydride | 3 | 125316 | 123687 | 146158 | 90600 | 86234 | 88276 | 103804 | 111517 | 136403 | 111259 | 97423 | 83516 |
| 673 | 143.03 | 69.03 | 5-hydroxymaltol | 2 | 5E+06 | 5E+06 | 5E+06 | 966937 | 1E+06 | 1E+06 | 3E+06 | 3E+06 | 3E+06 | 3E+06 | 3E+06 | 3E+06 |
| 674 | 173.01 | 71.03 | Dehydroascorbic acid | 3 | 411116 | 411210 | 364339 | 583351 | 544448 | 517555 | 306739 | 331052 | 335225 | 332448 | 337166 | 398131 |
| 675 | 173.01 | 129.02 | Cis-Aconitic acid | 3 | 2E+07 | 2E+07 | 2E+07 | 2E+07 | 2E+07 | 2E+07 | 2E+07 | 2E+07 | 2E+07 | 2E+07 | 2E+07 | 2E+07 |
| 676 | 150.08 | 133.05 | 1-Methyladenine | 3 | 641471 | 670571 | 580159 | 188727 | 286967 | 348328 | 191682 | 574477 | 301387 | 242240 | 298530 | 237968 |
| 677 | 127.04 | 55.02 | 4-Hydroxy-2,5-dimethyl-3(2H)furanone | 3 | 68560 | 78997 | 78991 | 112973 | 111671 | 76451 | 42042 | 33340 | 43155 | 73764 | 86226 | 51518 |
| 678 | 175.02 | 85.03 | D-Glucurono-6,3-lactone | 3 | 295054 | 268969 | 277025 | 67277 | 78405 | 90400 | 69525 | 205044 | 246477 | 245135 | 224198 | 233042 |
| 679 | 156.08 | 110.07 | 2-amino-3-(1H-pyrazol-1-yl)propanoic acid | 2 | 2E+06 | 2E+06 | 2E+06 | 3E+06 | 3E+06 | 3E+06 | 2E+06 | 3E+06 | 3E+06 | 2E+06 | 2E+06 | 2E+06 |
| 680 | 156.08 | 110.07 | L-Histidine | 2 | 5E+06 | 6E+06 | 7E+06 | 1E+07 | 8E+06 | 1E+07 | 6E+06 | 6E+06 | 6E+06 | 7E+06 | 7E+06 | 7E+06 |
| 681 | 188.06 | 128.04 | N-Acetyl-L-glutamic acid | 1 | 1E+06 | 1E+06 | 1E+06 | 3E+06 | 3E+06 | 3E+06 | 1E+06 | 1E+06 | 2E+06 | 2E+06 | 959154 | 2E+06 |
| 682 | 187.07 | 125.07 | N-Acetyl-L-Glutamine | 3 | 5E+06 | 6E+06 | 5E+06 | 4E+06 | 4E+06 | 4E+06 | 5E+06 | 5E+06 | 5E+06 | 5E+06 | 6E+06 | 4E+06 |
| 683 | 175.0612 | 115.0399 | 3-Isopropylmalic Acid* | 1 | 2E+07 | 2E+07 | 2E+07 | 2E+07 | 2E+07 | 2E+07 | 2E+07 | 2E+07 | 2E+07 | 2E+07 | 2E+07 | 2E+07 |
| 684 | 175.06 | 115.04 | 2-Isopropylmalic Acid | 1 | 2E+07 | 2E+07 | 2E+07 | 2E+07 | 2E+07 | 2E+07 | 2E+07 | 2E+07 | 2E+07 | 2E+07 | 2E+07 | 2E+07 |
| 685 | 192.07 | 56.05 | N-Acetyl-L-Methionine | 3 | 105450 | 28340 | 97923 | 371611 | 396637 | 426865 | 154925 | 171960 | 163807 | 237860 | 225729 | 176116 |
| 686 | 193.07 | 59.01 | D-Pinitol | 3 | 5E+06 | 5E+06 | 5E+06 | 5E+06 | 5E+06 | 5E+06 | 4E+06 | 4E+06 | 4E+06 | 5E+06 | 5E+06 | 5E+06 |
| 687 | 146.12 | 55.05 | N-Methylisoleucine | 2 | 1E+06 | 1E+06 | 1E+06 | 1E+06 | 1E+06 | 1E+06 | 1E+06 | 1E+06 | 1E+06 | 1E+06 | 1E+06 | 1E+06 |
| 688 | 289.03 | 96.97 | D-Sedoheptuiose 7-phosphate | 3 | 5E+06 | 3E+06 | 2E+06 | 1E+06 | 2E+06 | 777258 | 2E+06 | 1E+06 | 824536 | 1E+06 | 1E+06 | 1E+06 |
| 689 | 189.13 | 144.1 | Homoarginine | 3 | 1E+06 | 1E+06 | 1E+06 | 4E+06 | 4E+06 | 4E+06 | 1E+06 | 1E+06 | 2E+06 | 1E+06 | 1E+06 | 1E+06 |
| 690 | 211.06 | 96.96 | 6-methoxyhexyl hydrogen sulfate | 2 | 1E+06 | 1E+06 | 1E+06 | 850083 | 762077 | 806747 | 1E+06 | 1E+06 | 1E+06 | 975166 | 959363 | 1E+06 |
| 691 | 146.17 | 72.08 | Spermidine | 3 | 320227 | 373763 | 353585 | 341464 | 326874 | 323017 | 154462 | 143274 | 187499 | 329286 | 332820 | 359692 |
| 692 | 166.02 | 79.96 | Quinolinic Acid | 3 | 4E+06 | 3E+06 | 3E+06 | 3E+06 | 850450 | 2E+06 | 3E+06 | 944412 | 1E+06 | 3E+06 | 3E+06 | 3E+06 |
| 693 | 123.04 | 77.04 | 3-hydroxybenzaldehyde | 2 | 2E+06 | 2E+06 | 3E+06 | 3E+06 | 3E+06 | 3E+06 | 2E+06 | 2E+06 | 2E+06 | 2E+06 | 3E+06 | 3E+06 |
| 694 | 121.03 | 92.03 | 4-Hydroxybenzaldehyde | 1 | 1E+07 | 1E+07 | 1E+07 | 9E+06 | 9E+06 | 9E+06 | 1E+07 | 1E+07 | 1E+07 | 1E+07 | 1E+07 | 1E+07 |
| 695 | 137.02 | 93.03 | 2,5-Dihydroxybenzaldehyde | 2 | 7E+06 | 7E+06 | 7E+06 | 6E+06 | 7E+06 | 6E+06 | 9E+06 | 9E+06 | 8E+06 | 8E+06 | 7E+06 | 7E+06 |
| 696 | 137.02 | 93.03 | 4-Hydroxybenzoic acid | 2 | 1E+07 | 1E+07 | 1E+07 | 9E+06 | 1E+07 | 1E+07 | 1E+07 | 1E+07 | 1E+07 | 1E+07 | 1E+07 | 1E+07 |
| 697 | 137.02 | 93.04 | Protocatechualdehyde | 2 | 7E+06 | 7E+06 | 7E+06 | 7E+06 | 7E+06 | 6E+06 | 8E+06 | 9E+06 | 8E+06 | 8E+06 | 8E+06 | 8E+06 |
| 698 | 153.02 | 109.03 | 2,5-Dihydroxybenzoic acid; Gentisic Acid* | 1 | 3E+07 | 3E+07 | 2E+07 | 9E+06 | 8E+06 | 8E+06 | 2E+07 | 2E+07 | 2E+07 | 2E+07 | 2E+07 | 2E+07 |
| 699 | 153.02 | 109.03 | 3,4-Dihydroxybenzoic acid (Protocatechuic acid)* | 1 | 5E+07 | 5E+07 | 5E+07 | 2E+07 | 2E+07 | 2E+07 | 3E+07 | 3E+07 | 3E+07 | 4E+07 | 3E+07 | 3E+07 |
| 700 | 153.02 | 109.03 | Terreic acid | 1 | 3E+07 | 3E+07 | 3E+07 | 9E+06 | 9E+06 | 9E+06 | 1E+07 | 2E+07 | 2E+07 | 2E+07 | 2E+07 | 2E+07 |
| 701 | 153.02 | 109.03 | Methyl cumalate | 1 | 2E+07 | 2E+07 | 2E+07 | 9E+06 | 8E+06 | 8E+06 | 1E+07 | 2E+07 | 2E+07 | 2E+07 | 2E+07 | 2E+07 |
| 702 | 169.01 | 125.02 | Gallic acid | 1 | 4E+06 | 4E+06 | 4E+06 | 3E+06 | 3E+06 | 3E+06 | 4E+06 | 4E+06 | 4E+06 | 3E+06 | 3E+06 | 3E+06 |
| 703 | 169.01 | 151 | 2,3,4-Trihydroxybenzoic acid | 3 | 2E+06 | 1E+06 | 1E+06 | 1E+06 | 2E+06 | 1E+06 | 716525 | 901466 | 768532 | 1E+06 | 1E+06 | 1E+06 |
| 704 | 138.06 | 77.04 | Salicylamide | 3 | 276931 | 348714 | 320437 | 323957 | 253663 | 310545 | 210275 | 232864 | 240167 | 305529 | 294113 | 302030 |
| 705 | 138.05 | 78.03 | Nicotinic Acid Methyl Ester(Methyl Nicotinate) | 3 | 122697 | 119303 | 139813 | 172913 | 177757 | 156849 | 122535 | 102288 | 114976 | 114312 | 150361 | 136023 |
| 706 | 138.06 | 120.04 | 4-Aminobenzoic acid | 3 | 178405 | 205684 | 170339 | 170734 | 207184 | 160023 | 118496 | 76534 | 126867 | 127762 | 140845 | 147372 |
| 707 | 152.04 | 108.05 | 3-Aminosalicylic acid | 3 | 1E+06 | 1E+06 | 1E+06 | 406251 | 414089 | 412396 | 418031 | 386543 | 365078 | 715039 | 688578 | 753080 |
| 708 | 152.03 | 108.05 | 4-Aminosalicylic acid | 3 | 600463 | 286174 | 464664 | 107829 | 215659 | 95533 | 181121 | 179872 | 166409 | 232759 | 270126 | 329603 |
| 709 | 154.05 | 136.04 | 3-Hydroxyanthranilic acid | 3 | 1E+06 | 2E+06 | 1E+06 | 777488 | 683282 | 892433 | 657721 | 621285 | 603304 | 831060 | 880540 | 694175 |
| 710 | 153.07 | 108.04 | 1-Methyl-6-Oxo-1,6-Dihydropyridine-3-Carboxamide | 3 | 3E+06 | 3E+06 | 3E+06 | 4E+06 | 3E+06 | 3E+06 | 3E+06 | 3E+06 | 3E+06 | 2E+06 | 3E+06 | 3E+06 |
| 711 | 181.07 | 124.05 | 1,7-Dimethylxanthine | 2 | 3E+07 | 3E+07 | 3E+07 | 2E+07 | 2E+07 | 1E+07 | 3E+07 | 3E+07 | 3E+07 | 2E+07 | 2E+07 | 2E+07 |
| 712 | 171.03 | 109.03 | 3-Dehydroshikimic acid | 2 | 3E+06 | 3E+06 | 3E+06 | 3E+06 | 3E+06 | 3E+06 | 2E+06 | 2E+06 | 2E+06 | 3E+06 | 3E+06 | 3E+06 |
| 713 | 138.09 | 103.05 | L-Tyramine | 3 | 303490 | 254364 | 300035 | 136684 | 185324 | 137890 | 228115 | 214326 | 275116 | 204948 | 193740 | 196482 |
| 714 | 169.1 | 70.07 | Cyclo(L-Ala-L-Pro) | 3 | 820828 | 905678 | 918355 | 951019 | 869113 | 892299 | 1E+06 | 1E+06 | 1E+06 | 811827 | 928356 | 876686 |
| 715 | 296.07 | 104.11 | 5-Aminoimidazole ribonucleotide | 1 | 8E+06 | 8E+06 | 8E+06 | 7E+06 | 8E+06 | 8E+06 | 1E+07 | 1E+07 | 1E+07 | 9E+06 | 9E+06 | 1E+07 |
| 716 | 237.06 | 87.01 | Mucic acid Dimethyl Ester | 1 | 7E+06 | 7E+06 | 7E+06 | 1E+07 | 1E+07 | 1E+07 | 8E+06 | 8E+06 | 8E+06 | 9E+06 | 9E+06 | 9E+06 |
| 717 | 217.13 | 158.08 | N-Acetyl-L-Arginine | 2 | 2E+06 | 2E+06 | 3E+06 | 3E+06 | 4E+06 | 4E+06 | 2E+06 | 3E+06 | 2E+06 | 3E+06 | 3E+06 | 3E+06 |
| 718 | 207.09 | 59.01 | Dambonitol | 2 | 447439 | 510245 | 465326 | 534280 | 516566 | 580618 | 356824 | 453215 | 427734 | 493023 | 586076 | 474308 |
| 719 | 203.15 | 70.07 | N,N'-Dimethylarginine;SDMA* | 3 | 4E+06 | 4E+06 | 4E+06 | 5E+06 | 5E+06 | 5E+06 | 4E+06 | 4E+06 | 4E+06 | 4E+06 | 4E+06 | 5E+06 |
| 720 | 203.15 | 70.07 | NG,NG-Dimethyl-L-arginine* | 3 | 3E+06 | 4E+06 | 4E+06 | 4E+06 | 4E+06 | 4E+06 | 4E+06 | 4E+06 | 4E+06 | 3E+06 | 4E+06 | 4E+06 |
| 721 | 258.11 | 104.11 | Choline Alfoscerate | 3 | 6E+06 | 6E+06 | 6E+06 | 6E+06 | 6E+06 | 6E+06 | 1E+07 | 1E+07 | 1E+07 | 7E+06 | 8E+06 | 8E+06 |
| 722 | 165.02 | 77.04 | Phthalic acid | 3 | 207733 | 205781 | 191857 | 185879 | 198479 | 174636 | 205317 | 219038 | 221947 | 199073 | 202083 | 182546 |
| 723 | 118.06 | 91.05 | m-Aminophenylacetylene | 1 | 2E+07 | 2E+07 | 2E+07 | 2E+07 | 2E+07 | 2E+07 | 2E+07 | 2E+07 | 2E+07 | 2E+07 | 2E+07 | 2E+07 |
| 724 | 118.07 | 91.05 | Indole | 2 | 2E+07 | 2E+07 | 2E+07 | 2E+07 | 2E+07 | 2E+07 | 2E+07 | 2E+07 | 2E+07 | 2E+07 | 2E+07 | 2E+07 |
| 725 | 121.06 | 77.04 | Phenylacetaldehyde | 3 | 9 | 9 | 9 | 7853.1 | 11670 | 8884.9 | 125303 | 52678 | 78623 | 40715 | 24602 | 25634 |
| 726 | 135.05 | 92.03 | 4-Hydroxyacetophenone | 3 | 2E+06 | 2E+06 | 1E+06 | 1E+06 | 1E+06 | 2E+06 | 2E+06 | 2E+06 | 2E+06 | 3E+06 | 1E+06 | 2E+06 |
| 727 | 153.05 | 123.04 | Methyl salicylate | 3 | 77421 | 44034 | 68292 | 52080 | 40768 | 87166 | 29985 | 23155 | 39513 | 79227 | 57135 | 39341 |
| 728 | 167.03 | 121.03 | 3-Hydroxymandelate | 3 | 51306 | 65055 | 47251 | 27160 | 17742 | 22517 | 50015 | 51009 | 39850 | 21220 | 53176 | 42456 |
| 729 | 167.04 | 123.05 | 2',4',6'-Trihydroxyacetophenone | 1 | 411826 | 375377 | 411953 | 511652 | 515515 | 472883 | 258212 | 311958 | 279329 | 359935 | 356142 | 351061 |
| 730 | 167.03 | 123.05 | 3,4-Dihydroxybenzeneacetic acid | 3 | 2E+06 | 2E+06 | 2E+06 | 1E+06 | 1E+06 | 1E+06 | 485830 | 1E+06 | 682652 | 1E+06 | 2E+06 | 2E+06 |
| 731 | 183.03 | 124.02 | 3-O-Methylgallic acid | 1 | 2E+07 | 2E+07 | 2E+07 | 1E+07 | 1E+07 | 1E+07 | 2E+07 | 2E+07 | 2E+07 | 2E+07 | 2E+07 | 2E+07 |
| 732 | 183.03 | 139.04 | 3,5-Dihydroxy-4-methoxybenzoic acid; 4-O-Methylgallic Acid | 2 | 695489 | 691290 | 714083 | 413167 | 394615 | 408509 | 1E+06 | 1E+06 | 1E+06 | 595771 | 693907 | 641628 |
| 733 | 120.08 | 103.05 | N-Benzylmethylene isomethylamine | 1 | 1E+07 | 1E+07 | 1E+07 | 1E+07 | 1E+07 | 1E+07 | 7E+06 | 9E+06 | 8E+06 | 1E+07 | 9E+06 | 9E+06 |
| 734 | 168.07 | 105.03 | 4,5,6-Trihydroxy-2-cyclohexen-1-ylideneacetonitrile | 2 | 3E+06 | 3E+06 | 3E+06 | 4E+06 | 3E+06 | 3E+06 | 3E+06 | 3E+06 | 3E+06 | 3E+06 | 3E+06 | 3E+06 |
| 735 | 149.06 | 131.05 | p-Coumaryl alcohol | 3 | 21173 | 36262 | 32919 | 70007 | 55847 | 71678 | 32330 | 47120 | 43628 | 39379 | 48524 | 44530 |
| 736 | 165.06 | 106.04 | 3-Hydroxyphenylacetic Acid Methyl Ester | 3 | 164903 | 150165 | 166557 | 106349 | 103588 | 110647 | 153880 | 124176 | 156703 | 140924 | 122243 | 120971 |
| 737 | 165.06 | 119.05 | 2,6-Dimethoxybenzaldehyde | 3 | 692866 | 734615 | 731129 | 465136 | 475338 | 482339 | 489003 | 238540 | 305634 | 533066 | 370108 | 525846 |
| 738 | 165.06 | 119.05 | 3-(4-Hydroxyphenyl)-propionic acid | 3 | 659725 | 630945 | 586475 | 497925 | 479778 | 525676 | 666421 | 633740 | 694481 | 621634 | 581351 | 552955 |
| 739 | 181.05 | 135.04 | Methyl 2,4-dihydroxyphenylacetate | 1 | 398217 | 457407 | 463456 | 274450 | 242122 | 223962 | 392447 | 442165 | 466558 | 434690 | 300803 | 387171 |
| 740 | 181.05 | 135.04 | 4-Hydroxyphenyllactic Acid | 1 | 446744 | 448008 | 458511 | 252769 | 244687 | 224121 | 478305 | 428781 | 456525 | 362429 | 410778 | 380418 |
| 741 | 197.05 | 123.01 | Syringic acid | 1 | 908975 | 1E+06 | 919556 | 661173 | 510588 | 621561 | 947008 | 909370 | 886926 | 848445 | 820784 | 840083 |
| 742 | 197.05 | 153.06 | 4-Hydroxy-3-methoxymandelate | 3 | 615134 | 571294 | 623117 | 568361 | 560515 | 624774 | 697941 | 653232 | 665509 | 609526 | 569632 | 545619 |
| 743 | 166.09 | 120.08 | L-Phenylalanine | 1 | 5E+06 | 5E+06 | 5E+06 | 5E+06 | 5E+06 | 5E+06 | 4E+06 | 4E+06 | 4E+06 | 4E+06 | 5E+06 | 5E+06 |
| 744 | 180.07 | 119.05 | DL-O-tyrosine | 2 | 997446 | 939078 | 907041 | 1E+06 | 976142 | 865339 | 696333 | 720942 | 775441 | 812708 | 574209 | 797459 |
| 745 | 151.08 | 106.04 | 3-(4-Hydroxyphenyl)-1-propanol | 3 | 11387 | 17156 | 28340 | 14927 | 15603 | 23485 | 10069 | 11136 | 5108.5 | 26450 | 35010 | 12210 |
| 746 | 306.05 | 110.04 | 2'-Deoxycytidine-5'-monophosphate | 3 | 504423 | 460084 | 442597 | 530756 | 559057 | 535549 | 444450 | 501134 | 419404 | 437837 | 468910 | 415539 |
| 747 | 171.1 | 99.08 | 9-Oxononanoic acid | 3 | 498499 | 504372 | 491731 | 607435 | 586001 | 525254 | 441719 | 468299 | 434565 | 508050 | 487629 | 499128 |
| 748 | 204.13 | 85.03 | O-Acetyl-L-carnitine | 2 | 3E+06 | 3E+06 | 3E+06 | 3E+06 | 3E+06 | 3E+06 | 3E+06 | 3E+06 | 3E+06 | 3E+06 | 3E+06 | 3E+06 |
| 749 | 218.1 | 88.04 | 6-(((S)-1-carboxyethyl)amino)-4-hydroxyhexanoicacid | 1 | 6E+06 | 6E+06 | 6E+06 | 7E+06 | 7E+06 | 7E+06 | 5E+06 | 5E+06 | 6E+06 | 6E+06 | 6E+06 | 6E+06 |
| 750 | 220.12 | 90.05 | D-Pantothenic Acid | 1 | 407766 | 410145 | 381241 | 430023 | 434778 | 418038 | 344211 | 377542 | 333458 | 392727 | 310203 | 379883 |
| 751 | 220.12 | 90.06 | Casuarine Analogue | 3 | 9E+06 | 9E+06 | 9E+06 | 1E+07 | 1E+07 | 1E+07 | 9E+06 | 9E+06 | 8E+06 | 9E+06 | 9E+06 | 9E+06 |
| 752 | 266.09 | 134.05 | S-Ribosyl-L-homocysteine | 3 | 222390 | 214090 | 237210 | 239701 | 281755 | 245161 | 295359 | 381288 | 393614 | 245719 | 313318 | 299807 |
| 753 | 163.04 | 89.04 | 7-hydroxy-2H-1-benzopyran-2-one | 2 | 6E+06 | 7E+06 | 7E+06 | 7E+06 | 7E+06 | 7E+06 | 9E+06 | 9E+06 | 9E+06 | 6E+06 | 8E+06 | 7E+06 |
| 754 | 177.02 | 89 | 5,7-Dihydroxychromone | 2 | 2E+06 | 2E+06 | 2E+06 | 1E+06 | 1E+06 | 1E+06 | 2E+06 | 2E+06 | 2E+06 | 2E+06 | 1E+06 | 2E+06 |
| 755 | 179.03 | 123.04 | aesculetine | 2 | 2E+06 | 2E+06 | 2E+06 | 2E+06 | 2E+06 | 2E+06 | 1E+06 | 1E+06 | 1E+06 | 1E+06 | 2E+06 | 2E+06 |
| 756 | 146.06 | 91.05 | 8-hydroxyquinoline | 1 | 1E+07 | 9E+06 | 1E+07 | 9E+06 | 1E+07 | 9E+06 | 8E+06 | 9E+06 | 8E+06 | 8E+06 | 8E+06 | 9E+06 |
| 757 | 146.06 | 91.05 | Indole-3-carboxaldehyde | 1 | 4E+06 | 4E+06 | 4E+06 | 3E+06 | 3E+06 | 3E+06 | 4E+06 | 4E+06 | 4E+06 | 3E+06 | 4E+06 | 4E+06 |
| 758 | 146.06 | 105.04 | 2-Hydroxyquinoline | 3 | 11670 | 45010 | 28340 | 126102 | 49630 | 79422 | 67224 | 73355 | 60549 | 58606 | 56722 | 62134 |
| 759 | 163.04 | 119.05 | 2-Hydroxycinnamic acid* | 1 | 3E+06 | 3E+06 | 3E+06 | 3E+06 | 3E+06 | 3E+06 | 3E+06 | 3E+06 | 3E+06 | 3E+06 | 4E+06 | 3E+06 |
| 760 | 148.08 | 103.06 | (E)-Cinnamamide | 3 | 500324 | 544406 | 467601 | 302811 | 270474 | 232038 | 446759 | 471238 | 373559 | 396397 | 324885 | 357703 |
| 761 | 94.98 | 79.96 | Methanesulfonic acid | 3 | 4E+06 | 4E+06 | 4E+06 | 3E+06 | 3E+06 | 3E+06 | 6E+06 | 5E+06 | 6E+06 | 4E+06 | 4E+06 | 4E+06 |

a. Important non-volatile metabolites serial number detected by UPLC-MS/MS, b. Molecular weight of precursor ions after the addition of ions by an electrospray ion source; c. Characteristic fragment ions; d. Metabolite identification criteria, in which 1 indicates that the secondary mass spectrometry of the sample substance (all fragment ions of the substance), RT and database substance matching score is more than 0.7 points; 2 indicates that the secondary mass spectrometry of the sample substance (all fragment ions of the substance), RT and database substance matching score is 0.5-0.7 points; 3 indicates that the sample substance Q1, Q3, RT, DP, CE and the database substance are checked consistently; e. Abundance values of non-volatile metabolites in different black tea samples, wherein, CK indicates congou black tea, CH indicates chloranthus spicatus black tea, OF indicates osmanthus black tea; f. QC is a mixture of 9 sample extracts and is mainly used to analyze the repeatability of samples under the same treatment method. 1, 2, 3 indicates three replicates of samples. Metabolites with * indicate the possible presence of isomers that cannot be distinguished.

**TABLE S2.** Comparison of the abundance of important volatile differential metabolites in three samples.

| Index^a^ | Quan.^b^ | Qual. ^c^ | Compounds | RI^d^ | CK_1^e^ | CK_2 ^e^ | CK_3 ^e^ | OF_1 ^e^ | OF_2 ^e^ | OF_3 ^e^ | CH_1 ^e^ | CH_2 ^e^ | CH_3 ^e^ | QC01^f^ | QC02 ^f^ | QC03^f^ |
| --- | --- | --- | --- | --- | --- | --- | --- | --- | --- | --- | --- | --- | --- | --- | --- | --- |
| 1 | 85 | 29 | 2(3H)-Furanone, 5-hexyldihydro- | 1471 | 7533.223 | 5562.3 | 4761.925 | 8425.291 | 7939.046 | 5775.522 | 2437453 | 2482377 | 2239617 | 780823.8 | 817898.8 | 768706.6 |
| 2 | 121 | 161 | 2-Butanone, 4-(2,6,6-trimethyl-1-cyclohexen-1-yl)- | 1433 | 109294.7 | 115725.4 | 115025.5 | 115949.3 | 124027.5 | 103433.3 | 5190300 | 5354204 | 4791397 | 1799681 | 1883319 | 1890804 |
| 3 | 165 | 105 | Benzoic acid, 2-(methylamino)-, methyl ester | 1408 | 8337.429 | 9947.746 | 8352.122 | 18820.93 | 19769.89 | 15912.44 | 11587.26 | 10500.97 | 8360.825 | 11643.67 | 11531.67 | 11894.79 |
| 4 | 41 | 69 | 1,6,10-Dodecatrien-3-ol, 3,7,11-trimethyl- | 1563 | 28813.63 | 28003.12 | 27427.04 | 20806.67 | 23124.14 | 17698.43 | 27710.52 | 28005.06 | 26216.18 | 25642.48 | 26207.54 | 22790.43 |
| 5 | 98 | 41 | 2-Furanmethanol | 851 | 47368.12 | 40553.77 | 45307.02 | 98863.74 | 96586.61 | 83692.07 | 38780.42 | 36031.7 | 35046.95 | 52201.07 | 58009.55 | 49484.68 |
| 6 | 69 | 41 | Geraniol | 1255 | 32436086 | 32535836 | 30837238 | 46003696 | 48489408 | 42390782 | 34773952 | 32526549 | 30300574 | 36022781 | 36362036 | 35101165 |
| 7 | 137 | 152 | Phenol, 4-ethyl-2-methoxy- | 1282 | 8917.017 | 9863.445 | 7026.084 | 35299.22 | 36252.86 | 27612.08 | 31391.95 | 30289.36 | 29453.59 | 22955.05 | 22453.41 | 19416.54 |
| 8 | 161 | 134 | Naphthalene, 1,2,3,5,6,8a-hexahydro-4,7-dimethyl-1-(1-methylethyl)-, (1S-cis)- | 1524 | 28819.72 | 31257.53 | 30914.27 | 35943.27 | 36512.32 | 26482.85 | 60923.83 | 55922.5 | 54654.42 | 34268.92 | 35347.29 | 36842.98 |
| 9 | 41 | 93 | .alpha.-Farnesene | 1508 | 2940.663 | 2928.749 | 3365.586 | 2259.664 | 2577.274 | 1165.901 | 46513.73 | 47635 | 47055.8 | 11323.75 | 15020.62 | 14605.75 |
| 10 | 71 | 82 | Hotrienol | 1106 | 3217512 | 3120653 | 3113567 | 4088372 | 4196361 | 3565278 | 2307515 | 2209053 | 2107245 | 3019357 | 3213743 | 2894949 |
| 11 | 69 | 41 | 2,6-Octadien-1-ol, 3,7-dimethyl-, (Z)- | 1228 | 802625.3 | 799054.1 | 767193.3 | 986215.5 | 1057334 | 871298.2 | 647444.3 | 606379.3 | 534562.3 | 764642.2 | 800953.9 | 729751.4 |
| 12 | 57 | 72 | 1-Octen-3-ol | 982 | 1135853 | 1102787 | 1109512 | 635372.2 | 649212.3 | 557381.1 | 993106.5 | 1010094 | 1117464 | 867541.5 | 928114.5 | 834400.9 |
| 13 | 93 | 91 | .beta.-Ocimene | 1037 | 1612002 | 1682472 | 1739509 | 1901804 | 1996161 | 1567849 | 1086167 | 1209922 | 1093254 | 1241530 | 1478960 | 1365229 |
| 14 | 94 | 109 | Ethanone, 1-(1H-pyrrol-2-yl)- | 1063 | 678428.3 | 626717.4 | 648694.8 | 318250.4 | 356092.5 | 332115.5 | 582227.6 | 545094.8 | 489525.1 | 464703.9 | 535478.5 | 460298.5 |
| 15 | 71 | 93 | Linalool | 1099 | 10128280 | 9970092 | 10201705 | 13881955 | 14323011 | 12160172 | 9472582 | 9258593 | 8904454 | 10133414 | 10979229 | 10261703 |
| 16 | 69 | 41 | Geranic acid | 1355 | 168787.9 | 131121.4 | 101972.1 | 134993.6 | 289315.1 | 580569.4 | 408690.8 | 441497.4 | 257887.4 | 155740.6 | 226187.8 | 193743.6 |
| 17 | 104 | 105 | .beta.-Phenylethyl butyrate | 1444 | 59563.97 | 63393.21 | 61698.9 | 88426.6 | 103224 | 79641.46 | 129680.1 | 127358.3 | 113190.3 | 91813.97 | 98033.09 | 92084.71 |
| 18 | 95 | 150 | Cedrol | 1600 | 290.5685 | 354.8966 | 193.7901 | 350.7316 | 624.5515 | 544.1881 | 14377.66 | 15752.04 | 15468.48 | 5074.85 | 5196.033 | 4215.034 |
| 19 | 94 | 68 | Acetonitrile, 2,2'-iminobis- | 1108 | 1720768 | 1721186 | 1757815 | 2303761 | 2396549 | 1991244 | 1648028 | 1581059 | 1537908 | 1753587 | 1868912 | 1726717 |
| 20 | 91 | 92 | Benzeneacetamide | 1402 | 65204.52 | 68267.47 | 65263.54 | 148986 | 159653 | 141480.3 | 113915.7 | 114328.1 | 96024.2 | 100976.3 | 104075.8 | 99133.26 |
| 21 | 69 | 41 | 2-Butenoic acid, ethyl ester, (Z)- | 830 | 26825.26 | 26235.7 | 25137.89 | 108968.3 | 110169.9 | 95827.19 | 11264.86 | 9885.169 | 9310.604 | 37234.45 | 37627.22 | 39314.42 |
| 22 | 81 | 82 | Furan, 2-pentyl- | 993 | 444297.4 | 447986.4 | 446643.4 | 573923 | 577419.3 | 495512.1 | 247398.9 | 243488.5 | 243837.3 | 445440.5 | 489248.3 | 361540 |
| 23 | 55 | 84 | 2-Butenal, 2-methyl-, (E)- | 745 | 27049.34 | 31193.43 | 30717.23 | 49715.53 | 55931.73 | 46377.29 | 28617.69 | 23303.4 | 23510.47 | 36484.94 | 39355.74 | 30009.81 |
| 24 | 161 | 176 | 1-(2,3,4,5-Tetramethylphenyl)ethanone | 1482 | 58221.28 | 60987.42 | 62642.36 | 72871.62 | 89895.66 | 62122.45 | 288774.6 | 296695.3 | 302287.7 | 116386.9 | 116689.3 | 132127.9 |
| 25 | 105 | 119 | (1R,3aS,8aS)-7-Isopropyl-1,4-dimethyl-1,2,3,3a,6,8a-hexahydroazulene | 1440 | 17923.68 | 18420.73 | 17581.57 | 22977.77 | 24778.25 | 20005.22 | 1308377 | 1324391 | 1213420 | 447224.2 | 475104.6 | 457955.7 |
| 26 | 105 | 77 | 1,2-Ethanediol, monobenzoate | 1402 | 26226.64 | 27587.75 | 27317.32 | 61173.28 | 64166.83 | 58533.98 | 49397.74 | 44754.4 | 41284.59 | 40625.24 | 42353 | 41023.1 |
| 27 | 134 | 149 | BenzenAmine, 2,6-diethyl- | 1342 | 4757.897 | 4415.45 | 6403.165 | 9661.543 | 9721.937 | 7805.885 | 13584.16 | 13569.51 | 11337.13 | 8429.988 | 8243.121 | 7856.328 |
| 28 | 67 | 95 | cis-Dihydrocarvone | 1195 | 159579.8 | 133793.5 | 144373.1 | 171807.2 | 180551.7 | 145623.2 | 135895.8 | 121991.6 | 114714.7 | 153621.6 | 149441.4 | 129687.2 |
| 29 | 130 | 115 | 1H-Indene, 3-methyl- | 1155 | 1457.21 | 1424.184 | 1710.966 | 2301.372 | 2311.861 | 1976.316 | 38578.87 | 37492.83 | 33663.35 | 14831.38 | 15562.6 | 14848.33 |
| 30 | 67 | 82 | Butanoic acid, 3-hexenyl ester, (E)- | 1185 | 97547.61 | 99965.92 | 94585.41 | 112918.9 | 127292.6 | 88804.04 | 206357.1 | 201973.2 | 175287.1 | 129483.5 | 135580.4 | 130624 |
| 31 | 55 | 70 | 1-Butanol, 3-methyl-, formate | 792 | 354698.5 | 344003.2 | 335753 | 530511.8 | 524830.7 | 427363 | 183128.9 | 197742.3 | 174059.3 | 314401.8 | 322754.9 | 300754.1 |
| 32 | 41 | 105 | Naphthalene, decahydro-4a-methyl-1-methylene-7-(1-methylethenyl)-, [4aR-(4a.alpha.,7.alpha.,8a.beta.)]- | 1486 | 146162.8 | 145397.3 | 147973.7 | 155095.7 | 177667.3 | 138487.9 | 1208720 | 1227607 | 1146508 | 568171 | 557981.8 | 577607 |
| 33 | 161 | 81 | Azulene, 1,2,3,3a,4,5,6,7-octahydro-1,4-dimethyl-7-(1-methylethenyl)-, [1R-(1.alpha.,3a.beta.,4.alpha.,7.beta.)]- | 1473 | 223958.2 | 241122.1 | 248315.3 | 8841.04 | 10589.31 | 6554.784 | 89282.98 | 69220.52 | 56736.52 | 94903.31 | 97343.2 | 103114.3 |
| 34 | 83 | 84 | 1,5-Diazabicyclo[3.1.0]hexane | 951 | 169040.3 | 164312.8 | 165762.7 | 192902.5 | 202693.1 | 170092.5 | 156654.3 | 149848.3 | 143898.2 | 167497.3 | 170594.5 | 152990 |
| 35 | 99 | 42 | n-Pentyl methylphosphonofluoridate | 1073 | 52285.63 | 50942.34 | 50665.6 | 60794.3 | 68403.67 | 61931.07 | 50492.53 | 47789.29 | 48202.38 | 50963.83 | 56711.49 | 49565.32 |
| 36 | 58 | 57 | 2-Hexanone | 792 | 227285.6 | 212802.1 | 219162.1 | 371302.6 | 371684.2 | 311995.9 | 127884.6 | 122552.8 | 115091.3 | 195845 | 218562.2 | 202297.2 |
| 37 | 107 | 91 | D-Verbenone | 1228 | 33847.79 | 35120.19 | 33462.47 | 41837.28 | 40771.9 | 33778.1 | 27174.97 | 22758.92 | 20204.58 | 33444.24 | 29463.13 | 31960.16 |
| 38 | 91 | 92 | (5-bromopentyl)-Benzene | 1487 | 416290.9 | 410834.8 | 420780.2 | 430592.5 | 498275.7 | 391175.7 | 4098847 | 4180239 | 3863658 | 1772960 | 1762991 | 1780501 |
| 39 | 41 | 69 | 2,6-Octadienal, 3,7-dimethyl-, (Z)- | 1240 | 281791.6 | 290724.2 | 285227.4 | 492627.3 | 517914.1 | 405044.2 | 276450.1 | 281971.8 | 243488.7 | 324981.1 | 341709.7 | 310465.7 |
| 40 | 187 | 145 | aR-Himachalene | 1542 | 9 | 9 | 9 | 9 | 9 | 9 | 50286.65 | 53896.57 | 56267.04 | 18080.71 | 18322.46 | 15656.38 |
| 41 | 42 | 45 | 5-Methyloxazolidine | 795 | 213061.2 | 196167 | 201982.1 | 353113.4 | 334309.2 | 265625.9 | 102030.5 | 116364.5 | 112539.8 | 191207 | 194858.3 | 187279.5 |
| 42 | 83 | 55 | 3-Penten-2-one, 4-methyl- | 798 | 202526.4 | 197316.7 | 196285.3 | 186823.8 | 194589.6 | 165656 | 90156.22 | 84061.14 | 81826.59 | 147300.3 | 153362.6 | 138518.1 |
| 43 | 95 | 96 | 3-FurAldehyde | 831 | 732928 | 684629 | 681716 | 701251 | 736820 | 667976 | 182237.9 | 145628 | 147261 | 468344 | 513279 | 446257 |
| 44 | 70 | 69 | 1-Hexanol, 4-methyl- | 953 | 36565.6 | 36131.8 | 36064.31 | 46748.08 | 49457.62 | 42625.9 | 29606.91 | 24631.51 | 23888.45 | 31637.27 | 33320.98 | 31513.06 |
| 45 | 70 | 56 | 1-Heptanol | 970 | 43068.57 | 41576.75 | 39803.02 | 33202.99 | 34943 | 29442.35 | 11870.69 | 10508.6 | 9463.625 | 24964.34 | 26373.35 | 21018.66 |
| 46 | 100 | 85 | 3-Penten-2-one, 4-(acetyloxy)-, (Z)- | 1005 | 10648 | 14494 | 13089 | 17348 | 19333 | 15855 | 8851 | 9584 | 9210 | 12797 | 15401 | 11153 |
| 47 | 97 | 96 | Pyridine, 2,3,4,5-tetrahydro-6-propyl- | 1028 | 15591.75 | 13322.52 | 13585.14 | 45912.68 | 46624.54 | 37982.73 | 8766.714 | 9516.201 | 8597.396 | 28159.97 | 26296.96 | 16122.57 |
| 48 | 98 | 87 | 2-Heptanol, acetate | 1043 | 12805.27 | 12420.65 | 12222.55 | 13300.16 | 14642.96 | 12465.23 | 7988.393 | 7203.851 | 7738.761 | 9797.269 | 11881.71 | 9499.749 |
| 49 | 69 | 41 | 6-Octenal, 7-methyl-3-methylene- | 1147 | 83043.4 | 83752.94 | 86153.12 | 147462.8 | 152779.2 | 120809.2 | 72588.88 | 72268.23 | 66019.29 | 93985.94 | 94374.91 | 90066.63 |
| 50 | 57 | 71 | Undecane, 3,5-dimethyl- | 1207 | 76503.21 | 79076.25 | 77631.61 | 144016.8 | 160751.7 | 117072 | 69095.9 | 69735.62 | 59019.15 | 79033.57 | 86670.16 | 84700.68 |
| 51 | 81 | 94 | 2-Furanpropanoic acid, ethyl ester | 1192 | 197349.4 | 192648.9 | 194747.8 | 270671.7 | 288743.3 | 243049.8 | 219692.4 | 212906.3 | 181139.8 | 216345 | 227609 | 212384.3 |
| 52 | 57 | 85 | Decane, 3-ethyl-3-methyl- | 1229 | 282055.4 | 303905.7 | 294803.3 | 451931.7 | 504947.2 | 350692.7 | 342771 | 331010.3 | 295456.6 | 311984.5 | 324396.3 | 326632 |
| 53 | 121 | 136 | Ascaridole | 1244 | 33481.92 | 39873.31 | 37683.51 | 48868.74 | 53725.37 | 36843.91 | 33103.35 | 30987.87 | 29453.67 | 36706.67 | 36102.57 | 36000.45 |
| 54 | 126 | 57 | Dodecane, 5-methyl- | 1255 | 49215.35 | 53214.45 | 46496.96 | 77156.44 | 79294.67 | 65300.36 | 57278.58 | 50354.65 | 47275.99 | 54462.02 | 54570.07 | 54490.43 |
| 55 | 134 | 133 | Phenol, 4-(2-propenyl)- | 1255 | 25554.97 | 28232.29 | 27002.4 | 43738.3 | 44067.3 | 35804.09 | 34330.96 | 31110.76 | 28166.69 | 30785.2 | 29287.5 | 34862.37 |
| 56 | 41 | 55 | 2-Decenal, (Z)- | 1252 | 12201610 | 12356055 | 11715651 | 16869677 | 17599391 | 15763170 | 13178829 | 12354515 | 11563673 | 13633291 | 13692408 | 13383563 |
| 57 | 95 | 93 | Bornyl acetate | 1285 | 4485.857 | 6198.024 | 4811.639 | 12022.26 | 13410.73 | 8820.272 | 3322.456 | 3001.511 | 2959.059 | 3567.898 | 4248.332 | 5363.473 |
| 58 | 135 | 107 | Phenol, m-tert-butyl- | 1296 | 135238.3 | 144235.8 | 138601.4 | 65176.2 | 66554.31 | 56336.9 | 363606.5 | 362089.2 | 347017.5 | 207342.2 | 206210 | 182342.7 |
| 59 | 164 | 132 | Benzoic acid, 2-(dimethylamino)-, methyl ester | 1404 | 117999.4 | 141722 | 134370.6 | 309545.5 | 295372.1 | 282850.6 | 216644.4 | 217441.6 | 200306.9 | 200283.4 | 188431.7 | 193895.4 |
| 60 | 161 | 122 | .alpha.-Maaliene | 1443 | 8908.992 | 8465.942 | 9210.909 | 10316.92 | 10242.69 | 7449.706 | 4014787 | 4008270 | 3733545 | 1309340 | 1399052 | 1397341 |
| 61 | 41 | 70 | 2-Dodecenal, (E)- | 1468 | 68070.56 | 71283.19 | 69849.35 | 6014.765 | 9422.592 | 6568.749 | 147530.8 | 145330.1 | 128968.7 | 65352.51 | 68425.67 | 67453.55 |
| 62 | 57 | 41 | Dodecane, 5,8-diethyl- | 1572 | 28278.23 | 28557.65 | 27412.63 | 33038.22 | 37384.41 | 28682.39 | 45336.86 | 47162.85 | 43064.61 | 31972.08 | 30283.46 | 30449.82 |
| 63 | 71 | 95 | Butanoic acid, 1,7,7-trimethylbicyclo[2.2.1]hept-2-yl ester, endo- | 1476 | 9 | 9 | 9 | 9 | 9 | 9 | 38765.73 | 38819.99 | 35363.66 | 18308.68 | 17964.08 | 17572.07 |
| 64 | 97 | 41 | Dodecanenitrile | 1490 | 41084.09 | 42647.43 | 42216.23 | 46238.79 | 55069.71 | 41750.93 | 249969.7 | 252925.4 | 240893.5 | 117883.4 | 115964.2 | 120194.7 |
| 65 | 161 | 105 | (3S,3aR,3bR,4S,7R,7aR)-4-Isopropyl-3,7-dimethyloctahydro-1H-cyclopenta[1,3]cyclopropa[1,2]benzen-3-ol | 1515 | 8824.57 | 9922.397 | 8973.252 | 9827.841 | 10510.33 | 8500.16 | 18020.89 | 17102.3 | 17289.61 | 9410.044 | 9158.787 | 8539.692 |
| 66 | 119 | 105 | Cubenene | 1532 | 155323.6 | 173783.2 | 158007.6 | 195391.8 | 217359.9 | 157054.8 | 299405.4 | 326331.3 | 293105.8 | 208898.4 | 195901.5 | 201294.8 |
| 67 | 131 | 103 | 2-Propenoic acid, 3-phenyl-, methyl ester, (E)- | 1379 | 59001.19 | 68774.8 | 58113.15 | 7406.949 | 8602.611 | 6087.565 | 88304.32 | 91442.43 | 87523.9 | 49841.15 | 54032.21 | 52693.55 |
| 68 | 93 | 55 | 2,6,11-Dodecatrienal, 2,6-dimethyl-10-methylene- | 1695 | 2651.237 | 2797.059 | 2861.892 | 1615.02 | 1778.961 | 1612.428 | 25732.6 | 29002.15 | 22195.05 | 8163.028 | 8829.393 | 7330.088 |
| 69 | 183 | 198 | Naphthalene, 1,6-dimethyl-4-(1-methylethyl)- | 1674 | 33623.93 | 35993.32 | 37531.96 | 37980.93 | 39276.85 | 29642.17 | 67448.75 | 71052.29 | 62770.95 | 39342.33 | 44525.34 | 44292.72 |
| 70 | 74 | 87 | Hexadecanoic acid, methyl ester | 1926 | 31328.68 | 29675.3 | 27979.33 | 11665.76 | 15024.44 | 14379.96 | 23543.17 | 26597.39 | 19157.59 | 22207.68 | 24884.36 | 20427.93 |
| 71 | 69 | 39 | 3-Hexenal, (Z)- | 800 | 39811.2 | 37551.88 | 38832.93 | 65890.41 | 66147.06 | 54982.27 | 23438.31 | 23353.73 | 22942.99 | 34965.7 | 39206.68 | 35748.91 |
| 72 | 71 | 41 | Octane | 800 | 227053.4 | 221265 | 217972.9 | 356579.5 | 364159.2 | 292653.7 | 122762.1 | 128406.8 | 113583.7 | 203558.2 | 218310.4 | 200369.4 |
| 73 | 44 | 56 | Hexanal | 801 | 1829892 | 1704762 | 1706729 | 3070616 | 3013356 | 2473852 | 985562.8 | 980208.7 | 945477.6 | 1654163 | 1746400 | 1605255 |
| 74 | 41 | 69 | 3-Methylpenta-1,3-diene-5-ol, (E)- | 836 | 62741.68 | 72245.01 | 66300.62 | 190338.1 | 187696.4 | 154296.8 | 38916.13 | 29795.98 | 35331.83 | 78944.28 | 76952.18 | 81817.63 |
| 75 | 93 | 91 | .alpha.-Phellandrene 1 | 1006 | 261427.7 | 280881.1 | 304580.6 | 512760.7 | 441000.9 | 368651.6 | 251404.1 | 315042.8 | 304545.2 | 274876.5 | 311570.7 | 293820.6 |
| 76 | 68 | 93 | D-Limonene | 1030 | 1022861 | 826415.1 | 784050.9 | 1153469 | 1389015 | 1245891 | 835330.4 | 731433.8 | 645091.1 | 736404.1 | 885964 | 755770.9 |
| 77 | 71 | 57 | Nonane, 4,5-dimethyl- | 1046 | 4905.481 | 6352.137 | 6392.914 | 6979.867 | 7826.82 | 6515.958 | 18598.24 | 18936.19 | 16762.07 | 8329.752 | 9016.318 | 8811.296 |
| 78 | 154 | 95 | Citronellal | 1153 | 50646.59 | 50566.04 | 49773.1 | 76965.54 | 77295.09 | 63597.08 | 40023.47 | 38843.1 | 35375.17 | 49385.28 | 50876.17 | 49429.4 |
| 79 | 105 | 77 | Benzoic acid, ethyl ester | 1172 | 95500.27 | 96194.13 | 101254.6 | 100102.3 | 105025.2 | 80862.97 | 79898.79 | 77516.68 | 64985.41 | 85447.39 | 89886.1 | 86929.95 |
| 80 | 79 | 91 | Bicyclo[3.1.1]hept-2-ene-2-methanol, 6,6-dimethyl- | 1195 | 53265.67 | 56504.78 | 57121.41 | 85428.45 | 85615.84 | 71926.11 | 62010.32 | 60978.5 | 55830.17 | 65855.46 | 68305.23 | 65266.33 |
| 81 | 80 | 123 | Pyrazinamide | 1250 | 2645218 | 2666388 | 2521250 | 3894696 | 4117907 | 3498571 | 2852295 | 2659831 | 2481939 | 2987138 | 3017700 | 2894740 |
| 82 | 71 | 67 | 1-methyl-4-(1-methylethenyl)-1,2-Cyclohexanediol | 1321 | 45782.77 | 50975.04 | 51566.7 | 81441.07 | 94063.56 | 62701.69 | 40463.34 | 40257.23 | 34612.85 | 44649.56 | 46431.84 | 49151 |
| 83 | 91 | 104 | Hydrocinnamic Acid | 1356 | 44714.77 | 41437.4 | 47115.37 | 76723.13 | 93995.53 | 81303.63 | 166467.3 | 75257.11 | 60550.24 | 51787.72 | 60727.37 | 50462.07 |
| 84 | 131 | 103 | Vinyl trans-cinnamate | 1357 | 13743.82 | 14053.97 | 13016.43 | 13349.79 | 17535.61 | 14359.69 | 25606.48 | 29416.15 | 31421.26 | 16538.14 | 17941.07 | 18104.87 |
| 85 | 69 | 93 | 2,6-Octadien-1-ol, 3,7-dimethyl-, acetate, (Z)- | 1362 | 17712.87 | 18016.49 | 18143.33 | 30449.52 | 35588.67 | 32732.4 | 17613.97 | 16831.44 | 19640.44 | 17614.24 | 17797.05 | 18719.23 |
| 86 | 156 | 141 | Naphthalene, 1,4-dimethyl- | 1436 | 9752.158 | 10665.46 | 10022.99 | 16134.52 | 18561.34 | 15008.88 | 21572.17 | 20764.65 | 18737.5 | 15521.74 | 14187.24 | 15287.7 |
| 87 | 59 | 149 | 2-Naphthalenemethanol, 1,2,3,4,4a,5,6,8a-octahydro-.alpha.,.alpha.,4a,8-tetramethyl-, [2R-(2.alpha.,4a.alpha.,8a.beta.)]- | 1653 | 6224.02 | 6125.613 | 6409.493 | 750.6328 | 971.4915 | 690.3648 | 50835.66 | 52603.96 | 44075.14 | 19798.21 | 19363.23 | 17363.93 |
| 88 | 200 | 185 | Benzene, 1-methoxy-4-phenoxy- | 1632 | 6115.384 | 7479.304 | 6258.115 | 8168.511 | 8249.218 | 6296.386 | 10958.41 | 11067.21 | 10167.66 | 9002.636 | 7040.199 | 8052.304 |
| 89 | 161 | 105 | Bicyclo[4.4.0]dec-1-ene, 2-isopropyl-5-methyl-9-methylene- | 1507 | 61683.59 | 64318.37 | 62716.96 | 125041.7 | 149201.7 | 113966.9 | 304854 | 318174.6 | 312566.3 | 138459.1 | 131654 | 142850.2 |
| 90 | 69 | 41 | 1-Propanone, 1-cyclopropyl- | 756 | 52508.29 | 51251.26 | 49813.94 | 53439.98 | 57059.64 | 46745.87 | 21612.37 | 15145.1 | 13772.39 | 34298.3 | 39583.89 | 35449.88 |
| 91 | 68 | 59 | 2H-Pyran-3-ol, 6-ethenyltetrahydro-2,2,6-trimethyl- | 1173 | 7523615 | 7640353 | 7348016 | 8568872 | 8934320 | 7931368 | 8123615 | 7683822 | 7427218 | 7613631 | 8124051 | 7446454 |
| 92 | 70 | 42 | Cyclobutanone, 2,2,3-trimethyl- | 847 | 45313.47 | 35482.68 | 37260.68 | 99941.87 | 99152.26 | 85285.82 | 29745.9 | 26017.44 | 26226.94 | 50061.72 | 53810.39 | 44149.32 |
| 93 | 45 | 69 | 2-Hexanol | 801 | 349072.9 | 357735.2 | 346563.1 | 581969.7 | 591109.5 | 492783 | 198742.3 | 197279.9 | 191080.1 | 347026.3 | 338141.9 | 307941 |
| 94 | 67 | 45 | E-1-Methoxy-4-hexene | 801 | 315068.5 | 313694.1 | 305559.7 | 512824.3 | 529576.9 | 439087.9 | 176834.1 | 165690.3 | 164362.9 | 286090.7 | 315929.5 | 272834.8 |
| 95 | 91 | 106 | Benzene, 1,3-dimethyl- | 866 | 213309.4 | 209273.7 | 212511.9 | 212782.1 | 228976.4 | 175570.8 | 150684.5 | 135691.6 | 135086.8 | 153945.9 | 163729 | 153952.4 |
| 96 | 81 | 53 | Furan, 2-propyl- | 791 | 64316.72 | 64598.4 | 61408.92 | 102444.8 | 101070.6 | 89355.23 | 35046.54 | 32889.28 | 31778.4 | 56818.76 | 62212.54 | 55856.33 |
| 97 | 83 | 55 | 2-Butenoic acid, 3-methyl-, methyl ester | 842 | 8232.14 | 8857.767 | 7551.903 | 14013.26 | 16949.33 | 16719.08 | 5748.399 | 5975.665 | 6034.555 | 8967.701 | 8778.432 | 7973.999 |
| 98 | 67 | 54 | Norbornadieone | 913 | 24580.29 | 25575.56 | 23484.24 | 45923.64 | 46697.15 | 40249.46 | 10915.56 | 6621.906 | 6878.444 | 20498.24 | 24249.71 | 20647.44 |
| 99 | 108 | 111 | 5-Hepten-2-one, 6-methyl- | 986 | 384182.6 | 370759.3 | 381038.4 | 430231 | 448641.4 | 378326.7 | 252053.5 | 241353.5 | 240985.6 | 320472.1 | 346589 | 314897.6 |
| 100 | 121 | 136 | 2,4,6-Octatriene, 2,6-dimethyl- | 1131 | 77277.44 | 84232.45 | 93214.95 | 129519.9 | 123876.3 | 91897.6 | 62872.65 | 67533.97 | 61858.61 | 66473.27 | 79621.49 | 77019.29 |
| 101 | 93 | 91 | (+)-3-Carene | 1010 | 119027 | 96855 | 106046 | 148294 | 162814.3 | 125552.1 | 83834.92 | 87990.61 | 83384.35 | 88994.44 | 95786.44 | 93617 |
| 102 | 111 | 41 | 1-Nonene, 4,6,8-trimethyl- | 1012 | 14797.27 | 19048 | 15363 | 32620 | 32977 | 27998 | 12544 | 10672 | 9431 | 18624 | 21182 | 20533 |
| 103 | 67 | 95 | 1,3-Hexadiene, 3-ethyl-2-methyl- | 1031 | 592790.6 | 586139.9 | 565627.5 | 781240.5 | 867485.2 | 652071.2 | 496247.3 | 505829.9 | 427716 | 504641.7 | 580829 | 528378 |
| 104 | 107 | 79 | Furan, 2-(1-pentenyl)-, (E)- | 1000 | 98291.31 | 101838.6 | 108913.7 | 156200 | 173898.9 | 124101.7 | 61790.6 | 66253.24 | 59161.71 | 89040.6 | 103619.6 | 92015.06 |
| 105 | 121 | 136 | Pyrazine, 2-methyl-5-(1-methylethyl)- | 1059 | 36124.06 | 37094.83 | 39977.06 | 49218.18 | 55595.89 | 39334.2 | 29133.91 | 34647.71 | 32287.62 | 30866.79 | 32822.14 | 35934.51 |
| 106 | 58 | 43 | 2-Nonanone, 3-(hydroxymethyl)- | 1093 | 314246.3 | 314694.5 | 320889.9 | 399315.6 | 413343.6 | 351832.8 | 307078.3 | 297232.3 | 290140.9 | 324752.2 | 333729.2 | 314134.9 |
| 107 | 58 | 42 | 1,6-HexanediAmine, N,N,N',N'-tetramethyl- | 1206 | 11280.82 | 11430.16 | 10957 | 14996.99 | 17151.68 | 12068.76 | 8230.878 | 9047.716 | 9258.094 | 10676.17 | 11811.04 | 10168.28 |
| 108 | 55 | 41 | 3-Nonen-1-ol, (E)- | 1143 | 31015.5 | 34575.56 | 29687.92 | 36550.86 | 36177.16 | 30699.45 | 69845.3 | 66084.16 | 65371.64 | 42271.45 | 47717.59 | 46353 |
| 109 | 103 | 57 | Butanoic acid, 2-methyl-, hexyl ester | 1236 | 157800.2 | 171546.7 | 163654.1 | 345546.3 | 387557.8 | 262156.6 | 190523.1 | 182334.2 | 162159.7 | 177451.9 | 186936.9 | 196164.3 |
| 110 | 69 | 41 | 2,6-Octadienoic acid, 3,7-dimethyl-, methyl ester | 1323 | 1214154 | 1278229 | 1275856 | 1287445 | 1423263 | 1043176 | 927932.4 | 953575.1 | 847669.7 | 1035879 | 1089877 | 1070617 |
| 111 | 82 | 54 | Carvone | 1242 | 279143.9 | 277330.5 | 269601.5 | 361681.7 | 388349.7 | 309040.2 | 291248.5 | 290405.5 | 263744.7 | 290606.1 | 290179.8 | 274958.9 |
| 112 | 92 | 91 | Bicyclo[3.1.0]hexan-3-ol, 4-methylene-1-(1-methylethyl)-, acetate | 1292 | 8901.056 | 7857.386 | 8253.688 | 7666.635 | 6378.252 | 3770.774 | 14096.78 | 15444.08 | 14817.37 | 10216.58 | 12221.98 | 9356.937 |
| 113 | 91 | 65 | Thiocyanic acid, phenylmethyl ester | 1339 | 9 | 9 | 9 | 9 | 9 | 9 | 49992.37 | 50943.61 | 47390.82 | 22520.7 | 22638.14 | 18898.25 |
| 114 | 70 | 113 | L-Proline, 1-acetyl- | 1366 | 19323.84 | 19135.05 | 18776.64 | 21524.51 | 24187.62 | 17681.32 | 27584.23 | 28038.25 | 27381.57 | 19085.26 | 19620.17 | 19397.23 |
| 115 | 121 | 136 | 2-Acetoxyacetophenone | 1409 | 34473.48 | 38405.58 | 37918.06 | 86120.27 | 87305.46 | 82019.26 | 68831.2 | 60013.58 | 55017.75 | 55580.25 | 53431.36 | 53009.01 |
| 116 | 105 | 91 | (4R,4aS,6S)-4,4a-Dimethyl-6-(prop-1-en-2-yl)-1,2,3,4,4a,5,6,7-octahydronaphthalene | 1476 | 29262.31 | 28980.6 | 28674.28 | 30759.3 | 35352.72 | 24748.54 | 283130.2 | 290647.7 | 276312.5 | 89427.24 | 96033.47 | 95794.31 |
| 117 | 71 | 135 | N,N-Dimethyl-1,2,3-trithian-5-Amine | 1491 | 42045.52 | 41617.14 | 41850.4 | 45567.24 | 54704.73 | 40840.94 | 519450.1 | 527627.8 | 483585.3 | 211150.5 | 211998.4 | 215801 |
| 118 | 157 | 142 | 4-Isopropyl-6-methyl-1-methylene-1,2,3,4-tetrahydronaphthalene | 1563 | 134881.4 | 143541.9 | 136381.4 | 129191.2 | 146732.2 | 99799.09 | 247667.3 | 260941.2 | 227221.9 | 183192.3 | 174480 | 159927.5 |
| 119 | 161 | 122 | (4aR,8aR)-5,8a-dimethyl-3-propan-2-ylidene-1,2,4,4a,7,8-hexahydronaphthalene | 1542 | 5214 | 5932 | 5030 | 4683 | 4278 | 3243 | 3803347 | 4078269 | 4121607 | 1150992 | 1180457 | 982960 |
| 120 | 161 | 105 | (1.alpha.,4a.beta.,8a.alpha.)-1,2,3,4,4a,5,6,8a-octahydro-7-methyl-4-methylene-1-(1-methylethyl)-Naphthalene | 1513 | 3232.34 | 3581.91 | 2980.193 | 1474.637 | 1235.289 | 1637.305 | 45288.51 | 44147.14 | 49021.58 | 13680.71 | 13769.42 | 12931.16 |
| 121 | 107 | 79 | Benzenemethanol, .alpha.-2-cyclohexen-1-yl- | 1598 | 697.324 | 1134.339 | 1221.28 | 1495.837 | 1140.243 | 854.972 | 15300.58 | 15687.77 | 14609.58 | 5751.261 | 6829.603 | 4009.815 |
| 122 | 121 | 93 | (3R,3aR,7S,8aS)-3,6,8,8-Tetramethyl-4,7,8,8a-tetrahydro-1H-3a,7-methanoazulen-2(3H)-one | 1640 | 5875.35 | 5689.315 | 4920.509 | 4710.177 | 5778.493 | 4908.204 | 10422.37 | 11885.89 | 11203.04 | 7059.736 | 6998.891 | 6494.741 |
| 123 | 93 | 69 | Lavandulyl caproate | 1650 | 75749.61 | 75998.97 | 77666.88 | 22817.62 | 21012.91 | 16893.52 | 519892.2 | 550948.6 | 478752.1 | 205220.1 | 205376.7 | 185549.1 |
| 124 | 41 | 98 | Furan, 2,5-dihydro-3,4-dimethyl- | 731 | 25443.01 | 30590.26 | 25908.66 | 39864.02 | 41500.48 | 46266.84 | 26885.07 | 27914.58 | 25004.56 | 17959.58 | 23287 | 26279.21 |
| 125 | 55 | 42 | Pyridine, 2,3,4,5-tetrahydro- | 740 | 62719.73 | 70136.05 | 67288.68 | 86869.21 | 99375.77 | 65516.89 | 53207.04 | 53218.57 | 48165.47 | 52002.27 | 55963.04 | 58229.07 |
| 126 | 79 | 77 | 2,4-Heptadien-1-ol, (E,E)- | 1004 | 124743.8 | 137450.9 | 137122.3 | 283584.9 | 288373.7 | 250379.3 | 93239.93 | 89697.5 | 86132.09 | 146021.1 | 160482.8 | 152197.4 |
| 127 | 121 | 93 | 1,3-Cyclohexadiene, 1-methyl-4-(1-methylethyl)- | 1012 | 159694.4 | 182501.2 | 176917.8 | 235481.3 | 256093.5 | 202394.6 | 131348.7 | 144469 | 137882.2 | 140565.4 | 182935.1 | 162415.6 |
| 128 | 105 | 120 | Benzene, 1,2,3-trimethyl- | 1013 | 28224.36 | 30931.05 | 31385.1 | 42276.53 | 46041.18 | 37156.56 | 22270.51 | 24232.6 | 23440.83 | 23197.61 | 29405.53 | 27937.9 |
| 129 | 76 | 91 | Carbonochloridodithioic acid, methyl ester | 1033 | 271037 | 276820.5 | 278971 | 303519 | 313667.4 | 290856.4 | 277672.8 | 263050.4 | 252732.5 | 264850.7 | 294391.9 | 259920.1 |
| 130 | 42 | 103 | 1H-1,2,4-Triazole, 3-chloro- | 1237 | 23363.09 | 24570.99 | 22801.84 | 39619.82 | 46563.02 | 30415.56 | 26677.7 | 27066.12 | 23947.36 | 24382.75 | 26528.47 | 26844 |
| 131 | 71 | 99 | Bicyclo[3.1.1]heptan-3-one, 2-hydroxy-2,6,6-trimethyl- | 1256 | 2323143 | 2352469 | 2226243 | 3331356 | 3533670 | 3042259 | 2512953 | 2334866 | 2171485 | 2593260 | 2623082 | 2526877 |
| 132 | 95 | 121 | Bicyclo[2.2.1]heptan-2-ol, 1,7,7-trimethyl-, formate, endo- | 1226 | 41275.51 | 39733.32 | 39439.99 | 50625.34 | 48387.73 | 40728.56 | 31667.64 | 27170.13 | 24030.28 | 37110.21 | 39608.13 | 36568.42 |
| 133 | 57 | 41 | 2-Decen-1-ol, (E)- | 1257 | 622457.3 | 618560.9 | 594941.3 | 888404.3 | 930237.4 | 803849.7 | 676372.8 | 635567 | 593447.4 | 692647.1 | 694936.2 | 680957.2 |
| 134 | 142 | 69 | 4H-Pyran-4-one, 5-hydroxy-2-(hydroxymethyl)- | 1462 | 5502.243 | 6479.982 | 6050.564 | 3549.334 | 4357.278 | 3051.386 | 9176.961 | 8194.476 | 7918.64 | 5957.891 | 5914.922 | 5874.11 |
| 135 | 182 | 75 | TriSulfur compounds, dipropyl | 1328 | 9024.616 | 5414.338 | 6973.663 | 3615.983 | 3081.4 | 2502.41 | 2655.872 | 3002.682 | 3131.06 | 6583.571 | 8993.011 | 4950.365 |
| 136 | 69 | 55 | 8-Methyl-6-nonenoic Acid | 1373 | 16949.84 | 16743.53 | 16875.04 | 38567.39 | 41053.43 | 31176.7 | 13361.32 | 13425.02 | 12437.6 | 17796.9 | 18770.59 | 17427.06 |
| 137 | 81 | 80 | (-)-.beta.-Bourbonene | 1384 | 87374.62 | 93331.91 | 89174.77 | 86117.08 | 95459.31 | 69892.16 | 212862.7 | 223225.9 | 213555.8 | 114082.9 | 121711.5 | 115402 |
| 138 | 41 | 69 | (E)-.beta.-Famesene | 1457 | 19119.57 | 19738.55 | 18455.77 | 16258.45 | 19259.92 | 16758.56 | 47130.66 | 47970.44 | 46021.41 | 26569.93 | 27587.18 | 26557.12 |
| 139 | 95 | 82 | 2H-Pyran-2-one, 6-pentyl- | 1453 | 66693.47 | 65900.32 | 72906.74 | 44323.83 | 50799.34 | 45309.7 | 76066.2 | 70176.72 | 77530.65 | 73489.51 | 72595.95 | 66194.51 |
| 140 | 91 | 119 | (6,6-dimethyl-2-bicyclo[3.1.1]hept-2-enyl)methyl 2-methylpropanoate | 1466 | 223554.1 | 226826.6 | 225292.4 | 19624.66 | 23278.45 | 18463.22 | 86418.69 | 81264.34 | 59522.17 | 100136.3 | 103584.7 | 105338.2 |
| 141 | 97 | 68 | 2H-Pyran-2-one, 5,6-dihydro-6-pentyl- | 1501 | 4509.99 | 4460.901 | 4066.043 | 2537.847 | 2951.287 | 2061.959 | 24434.88 | 24617.61 | 24708.94 | 8509.257 | 8787.63 | 8666.082 |
| 142 | 119 | 93 | 1,3-Cyclohexadiene, 5-(1,5-dimethyl-4-hexenyl)-2-methyl-, [S-(R*,S*)]- | 1495 | 24291.13 | 25415.11 | 25141.14 | 8043.355 | 8066.833 | 7028.659 | 142173.9 | 142480.1 | 149758.9 | 48774.92 | 51324.01 | 50248.68 |
| 143 | 121 | 93 | (1S,2E,6E,10R)-3,7,11,11-Tetramethylbicyclo[8.1.0]undeca-2,6-diene | 1495 | 29394.35 | 29333.04 | 29007.19 | 4566.018 | 4935.114 | 3929.541 | 209036.8 | 206339 | 224680.1 | 69177.98 | 69952.53 | 70055.1 |
| 144 | 69 | 71 | Butanoic acid, 3,7-dimethyl-2,6-octadienyl ester, (E)- | 1562 | 78399.92 | 78079.53 | 73940.69 | 51663.3 | 63352.6 | 51378.31 | 73090.22 | 75849.18 | 63300.38 | 70324.05 | 70380.56 | 60111.11 |
| 145 | 119 | 132 | Benzene, 1-(1,5-dimethyl-4-hexenyl)-4-methyl- | 1483 | 15165.99 | 16856.5 | 16800.01 | 19754.02 | 23479.33 | 16056.02 | 127633.9 | 132824.6 | 130758.6 | 44832.52 | 48020.23 | 50751.84 |
| 146 | 158 | 157 | 6-Quinolinamine, 2-methyl- | 1650 | 3301.697 | 4069.937 | 4494.046 | 3430.722 | 2586.92 | 2178.802 | 9632.812 | 10835.31 | 9255.472 | 6376.78 | 5021.326 | 5449.847 |
| 147 | 139 | 67 | Hexahydro-3-butylphthalide | 1653 | 1857.219 | 1647.385 | 2358.188 | 9 | 9 | 9 | 16746.94 | 16533.54 | 15541.48 | 5830.682 | 6106.468 | 5352.059 |
| 148 | 57 | 44 | Cyclopentanol | 768 | 199815.6 | 205631.5 | 182755.1 | 256497.6 | 261776.3 | 235129.6 | 73206.57 | 26261.95 | 107431.7 | 149750.7 | 182395 | 146629.7 |
| 149 | 55 | 70 | 2-Propenoic acid, pentyl ester | 974 | 118207.4 | 116224.9 | 116205.8 | 66030.21 | 65070.37 | 60963.99 | 105730.6 | 104108.9 | 106701.9 | 85744 | 97565.88 | 79007.22 |
| 150 | 82 | 39 | Dehydromevalonic lactone | 1169 | 72178.44 | 79064.79 | 72156.87 | 114235.5 | 120212.4 | 103829.6 | 82980.02 | 81494.08 | 77478.73 | 84786.07 | 92005.28 | 80236.58 |
| 151 | 94 | 109 | 3-Acetyl-1H-pyrroline | 1035 | 157850.2 | 168516.3 | 184272.1 | 204634.7 | 208859.4 | 166589 | 108502.5 | 123292.5 | 115936 | 124857.9 | 152303.6 | 142120.3 |
| 152 | 57 | 71 | Nonane, 3-methyl-5-propyl- | 1185 | 11173.81 | 9496.039 | 10647.75 | 11817.23 | 14839.71 | 12615.1 | 10311.3 | 11400.87 | 10229.17 | 8892.214 | 9619.995 | 11013.52 |
| 153 | 67 | 109 | 3-Oxatricyclo[4.1.1.0(2,4)]octane, 2,7,7-trimethyl- | 1095 | 2265034 | 2236142 | 2289683 | 3063032 | 3159085 | 2653374 | 2121130 | 2084399 | 2008141 | 2303443 | 2444481 | 2293600 |
| 154 | 161 | 119 | Benzene, 2-methyl-1,4-bis(1-methylethyl)- | 1289 | 89393.94 | 94375.58 | 91886.92 | 9 | 9 | 9 | 331121.2 | 335926.1 | 325497.1 | 153682.1 | 162156.3 | 140138.8 |
| 155 | 95 | 124 | 1-(Furan-2-yl)-2-methylpentan-1-one | 1268 | 73277.95 | 80527.48 | 77809.42 | 137733.6 | 146235.5 | 118646.3 | 73510.15 | 70565.51 | 68112.46 | 89319.38 | 91073.89 | 86647.92 |
| 156 | 119 | 134 | Bicyclo[3.1.1]hept-2-en-6-ol, 2,7,7-trimethyl-, acetate, [1S-(1.alpha.,5.alpha.,6.beta.)]- | 1239 | 54619.8 | 57112.75 | 60794.65 | 119099.4 | 121170.7 | 91766.43 | 58157.77 | 55126.8 | 46236.55 | 68324.96 | 70885.49 | 67504.2 |
| 157 | 79 | 107 | 4-Isopropylcyclohexa-1,3-dienecarbAldehyde | 1283 | 9125.776 | 7947.183 | 7757.895 | 19369.98 | 23547.53 | 19949.44 | 4865.087 | 5142.67 | 3313.455 | 7169.923 | 9231.314 | 9579.83 |
| 158 | 105 | 77 | 1-Pentanone, 1-phenyl- | 1374 | 32102.74 | 38208.52 | 33561.47 | 15403.65 | 17257.54 | 14661.44 | 28561.01 | 30526.37 | 26972.89 | 22772.26 | 24249.25 | 23476.08 |
| 159 | 161 | 119 | Copaene | 1376 | 124955.6 | 146920.6 | 130795.4 | 73164.94 | 81123.14 | 55962.72 | 908228.7 | 922191.6 | 924253.4 | 302771.6 | 344425.1 | 324041 |
| 160 | 91 | 73 | .beta.-Phenylethylmethylethylcarbinol | 1382 | 47990.55 | 50207.34 | 45422.49 | 38373.81 | 43740.28 | 30700.51 | 279388.4 | 298469.8 | 289242.9 | 106124.2 | 116431.8 | 110717.5 |
| 161 | 161 | 69 | 1H-3a,7-Methanoazulene, octahydro-3,8,8-trimethyl-6-methylene-, [3R-(3.alpha.,3a.beta.,7.beta.,8a.alpha.)]- | 1421 | 6706.367 | 7404.515 | 8330.479 | 44980.58 | 44886.18 | 34704.61 | 35841.83 | 28690.07 | 26370.61 | 25094.31 | 23304.67 | 23495.27 |
| 162 | 93 | 80 | Humulene | 1454 | 112123.6 | 113885.1 | 124712.4 | 76867.27 | 90286.28 | 77825.16 | 167813.7 | 161884.8 | 165118.1 | 135916.7 | 137709 | 129208.4 |
| 163 | 175 | 190 | Benzene, 1,2-diethyl-3,4,5,6-tetramethyl- | 1558 | 958 | 814 | 772.5942 | 507.4472 | 767 | 526.0284 | 272042 | 291891 | 296169 | 81938 | 83938 | 70852 |
| 164 | 69 | 41 | 2,6,10-Dodecatrienal, 3,7,11-trimethyl- | 1656 | 27481.29 | 31987.65 | 30990.25 | 15286.91 | 16808.63 | 11341.17 | 147931 | 158310.8 | 137949.3 | 67455.76 | 66311.73 | 63338.37 |
| 165 | 83 | 55 | Dicyclohexyldisulphide | 1863 | 7968.358 | 7765.547 | 7178.666 | 8549.451 | 7916.307 | 10937.26 | 16519.82 | 15885.37 | 12964.28 | 5100.513 | 9196.62 | 7706.262 |
| 166 | 55 | 45 | 2-Propenoic acid, 2-methoxyethyl ester | 851 | 317614.3 | 299426.2 | 303576.5 | 390657.4 | 363984.3 | 333079 | 232694.9 | 196885.3 | 202937.5 | 282772.6 | 284702.7 | 261121.4 |
| 167 | 56 | 43 | 1-Hexanol | 868 | 353560 | 333642 | 346621 | 517918 | 479773 | 422229 | 199249 | 154707 | 178020 | 296406 | 312198 | 286308 |
| 168 | 57 | 71 | 3-Hexanone, 2,2-dimethyl- | 868 | 215086 | 192545 | 203623 | 254421 | 291280 | 240795 | 110620 | 94208.1 | 88712 | 173586 | 190797 | 161202 |
| 169 | 60 | 73 | Hexanoic Acid | 990 | 4805550 | 4476681 | 4124037 | 5659312 | 5938954 | 6003590 | 4523836 | 3791892 | 3787427 | 4262585 | 4972290 | 4267504 |
| 170 | 91 | 122 | Benzene, (methoxymethyl)- | 969 | 66241.11 | 68825.2 | 64140.04 | 9 | 9 | 9 | 512847.6 | 549093.8 | 522912 | 147430.3 | 167932.1 | 139624.3 |
| 171 | 81 | 69 | Fenchone | 1096 | 1607615 | 1591586 | 1634468 | 2204764 | 2290824 | 1930528 | 1518606 | 1462150 | 1425608 | 1624165 | 1743107 | 1637940 |
| 172 | 96 | 95 | 1H-Pyrazole-1-carboximidamide, 3,5-dimethyl- | 1096 | 2339308 | 2388176 | 2453407 | 2817646 | 2960199 | 2469428 | 2343749 | 2254065 | 2201385 | 2402522 | 2524709 | 2311516 |
| 173 | 83 | 55 | 2-Methyl-6-methyleneocta-2,7-dien-4-one | 1145 | 24242.84 | 25988.82 | 25813.28 | 48402.18 | 48821.88 | 39034.76 | 21182.05 | 18017.66 | 19439.26 | 33660.17 | 30619.26 | 26817.99 |
| 174 | 84 | 41 | 4-Decenal, (E)- | 1198 | 53385.51 | 56927.28 | 54537.38 | 54423.1 | 63097.63 | 45288.63 | 97906.19 | 99087.43 | 88458.34 | 60603.4 | 62246.73 | 61330.81 |
| 175 | 69 | 41 | 2,6-Octadienenitrile, 3,7-dimethyl-, (Z)- | 1231 | 784469.5 | 781397.2 | 748348 | 960996.1 | 1031819 | 855564.1 | 637895.9 | 593537.6 | 526535.5 | 749657.3 | 782937.8 | 711261.5 |
| 176 | 135 | 108 | Benzothiazole | 1228 | 27148.51 | 33304.64 | 32458.58 | 33417.85 | 33661.46 | 31033.93 | 24946.58 | 23427.27 | 22152.69 | 29233.59 | 28070.36 | 27326.16 |
| 177 | 93 | 80 | Linalyl acetate | 1257 | 6652023 | 6818058 | 6414375 | 10325372 | 10610981 | 9046626 | 7308131 | 6785269 | 6284603 | 7615403 | 7627851 | 7498342 |
| 178 | 41 | 55 | 2,4-Decadien-1-ol | 1274 | 526585.7 | 554790.8 | 539100.2 | 1036666 | 1072573 | 879486.1 | 493640.9 | 486326.3 | 448582.8 | 631276.9 | 628443.3 | 642836 |
| 179 | 119 | 120 | Di-epi-.alpha.-cedrene-(I) | 1382 | 76665.9 | 87281.45 | 77854.77 | 65381.74 | 74304.41 | 51393.92 | 719882.8 | 746132.9 | 755865 | 237881.4 | 269077.7 | 254625 |
| 180 | 107 | 122 | Ethanol, 2-(4-ethylphenoxy)- | 1424 | 9 | 9 | 9 | 9 | 9 | 9 | 38917.57 | 40298.89 | 40640.4 | 9268.911 | 10890.56 | 9390.295 |
| 181 | 119 | 134 | 1-Pentanone, 1-(4-methylphenyl)- | 1440 | 3781.921 | 4000.908 | 4061.889 | 6410.253 | 8131.091 | 6893.285 | 1789221 | 1805215 | 1672054 | 593486.2 | 642158.8 | 624564.8 |
| 182 | 123 | 124 | 6-Methyl-6-(5-methylfuran-2-yl)heptan-2-one | 1433 | 1496.778 | 2561.066 | 2623.816 | 2353.875 | 2057.875 | 1485.602 | 79410.84 | 82753.95 | 73730.18 | 28611.79 | 28994.13 | 29217.9 |
| 183 | 135 | 77 | 1-Propanone, 1-(4-methoxyphenyl)- | 1513 | 2641.813 | 2608.604 | 2401.123 | 1937.07 | 2234.637 | 2053.902 | 17429.59 | 17269.53 | 20174.1 | 6024.845 | 5669.62 | 5807.224 |
| 184 | 57 | 71 | Tetradecane, 2,6,10-trimethyl- | 1539 | 10172.29 | 11093.57 | 10335.9 | 15745.1 | 19119.76 | 13457.03 | 10779.38 | 10799.55 | 10580.39 | 10865.07 | 11420.09 | 12306.86 |
| 185 | 161 | 122 | Ledol | 1565 | 26623.65 | 28052.19 | 27599.11 | 17175.68 | 22399.54 | 17541.28 | 34155.27 | 33582.47 | 28866.45 | 27935.11 | 24878.11 | 21328.79 |
| 186 | 162 | 161 | 4-MethoxycinnamAldehyde | 1566 | 5499.325 | 5640.116 | 5656.007 | 3734.15 | 4943.65 | 4001.264 | 6893.742 | 8081.839 | 6709.482 | 6387.453 | 4654.834 | 3997.317 |
| 187 | 133 | 119 | Acetic acid, (4,6,8,9-tetramethyl-3-oxabicyclo[3.3.1]non-6-en-1-yl)methyl ester | 1667 | 52684.43 | 57017.55 | 59577.6 | 9930.594 | 10326.93 | 8449.311 | 430311.4 | 459722 | 399767.6 | 167582.9 | 167899.9 | 152110.9 |
| 188 | 55 | 82 | cis-9-Tetradecen-1-ol | 1666 | 34088.19 | 34593.36 | 36525.66 | 7706.725 | 7095.239 | 6398.601 | 268030.9 | 282320.2 | 244042.9 | 104345.9 | 105682.7 | 94294.78 |
| 189 | 131 | 103 | 2-Propenoic acid, 3-phenyl-, ethyl ester, (E)- | 1463 | 44990.92 | 48316.74 | 47715.78 | 13996.8 | 19304.25 | 14922.37 | 27281.62 | 25733.8 | 24603.81 | 28722.91 | 30353.17 | 30193.6 |
| 190 | 91 | 92 | Toluene | 763 | 171581 | 161345 | 166150 | 155161 | 162402 | 134797 | 59717 | 56011 | 55404 | 108545 | 117726 | 107785 |
| 191 | 57 | 41 | 2-Hexen-1-ol, (E)- | 862 | 128079.6 | 105614.8 | 107879.6 | 237341 | 263135.4 | 185943.1 | 136101.8 | 93554.95 | 101624.1 | 89347.15 | 99032.57 | 80816.19 |
| 192 | 98 | 83 | 2-Hexenal, (E)- | 854 | 723024.3 | 750782.7 | 679775.6 | 771837.4 | 673641.6 | 656410.3 | 647123 | 575286.3 | 578290.5 | 729506.1 | 689303.6 | 549477.3 |
| 193 | 69 | 41 | 4-Hexen-3-one | 855 | 243906.9 | 218059 | 235205.9 | 288932.4 | 289663.4 | 246304.3 | 169520.6 | 136205.8 | 141284.9 | 205637.9 | 215816.3 | 192521.2 |
| 194 | 44 | 45 | Ethanediamide | 1039 | 17952.59 | 20909.1 | 22986.42 | 26519.78 | 25661.25 | 25051.67 | 17654.68 | 20521.29 | 18794.64 | 16875.22 | 22432.9 | 21084.79 |
| 195 | 79 | 108 | Benzyl Alcohol | 1036 | 11009490 | 11050446 | 10930456 | 11838371 | 12172857 | 11470491 | 10990890 | 10281447 | 9775506 | 10427216 | 11594551 | 10006632 |
| 196 | 55 | 81 | 3-Octen-1-ol, (Z)- | 1055 | 116951.9 | 130549.6 | 134953.6 | 180838.4 | 183187.8 | 155326.9 | 115696.1 | 111212.4 | 108750.8 | 127512.8 | 134540.6 | 126941.5 |
| 197 | 135 | 150 | 3-Methyl-2-(2-methyl-2-butenyl)-furan | 1093 | 15512.31 | 16185.58 | 17522.65 | 22158.24 | 25404.4 | 18115.63 | 14927.12 | 15805.79 | 15180.66 | 15866 | 16941.02 | 15798.53 |
| 198 | 136 | 79 | Adamantane | 1116 | 9922.236 | 10408.84 | 11352.58 | 4959.965 | 5688.054 | 4743.667 | 6283.702 | 6319.115 | 5370.516 | 6634.724 | 7969.514 | 8105.19 |
| 199 | 149 | 150 | 2,3,5-Trimethyl-6-ethylpyrazine | 1163 | 14820.66 | 13629.51 | 15277.42 | 4347.154 | 4513.345 | 4935.459 | 8154.905 | 7718.864 | 8590.703 | 10540.98 | 9288.681 | 10308.5 |
| 200 | 59 | 93 | L-.alpha.-Terpineol | 1190 | 349960.5 | 343458.3 | 348937.6 | 483623.5 | 510906.4 | 423537.6 | 386703.8 | 375272.3 | 344261 | 394887.1 | 405686.3 | 372576.5 |
| 201 | 131 | 132 | 2-Propenal, 3-phenyl- | 1274 | 43874.93 | 51083.18 | 43505.92 | 53585.36 | 53525.22 | 52010.42 | 32270.94 | 33647.03 | 28945.04 | 40619.4 | 42956.18 | 36270.14 |
| 202 | 69 | 41 | Geranyl formate | 1301 | 152176.8 | 147255 | 141144.2 | 353866.2 | 399711.9 | 305977.2 | 184309.8 | 160660 | 147394.2 | 185217.8 | 211095.6 | 194177.9 |
| 203 | 98 | 70 | 1,3-Cyclopentanedione, 4-(3-methylbutyl)- | 1337 | 18922.98 | 18144.24 | 20788.18 | 24652.77 | 30452.68 | 28191.38 | 20818.43 | 22170.75 | 20750.52 | 21006.37 | 20557.74 | 19967.19 |
| 204 | 56 | 57 | Butyl caprylate | 1389 | 7332.111 | 7344.861 | 7001.281 | 8593.131 | 10446.85 | 7291.175 | 13648.4 | 12696.52 | 11841.03 | 7574.696 | 7699.596 | 6978.594 |
| 205 | 91 | 105 | 3-Hexanone, 1-phenyl- | 1427 | 26101.83 | 27988.96 | 26931.04 | 33624.65 | 40615.67 | 29796.4 | 1388165 | 1419549 | 1285637 | 487172.4 | 513095.2 | 499710 |
| 206 | 99 | 57 | (2E)-2-(Acetylhydrazono)propanoic acid | 1468 | 10044.18 | 10072.64 | 10266.78 | 6173.217 | 7186.76 | 4814.977 | 20475.4 | 20504.48 | 18415.18 | 10877.62 | 12067.67 | 10860.71 |
| 207 | 161 | 204 | Epizonarene | 1501 | 65126.79 | 66004.76 | 62926.11 | 60509.93 | 68091.78 | 54701.54 | 147707.5 | 147748.4 | 152319.8 | 94367.71 | 94436.1 | 77834.02 |
| 208 | 161 | 105 | .beta.-Guaiene | 1490 | 70122.8 | 67831.71 | 72115.12 | 78761.05 | 93819.48 | 74615.69 | 673641 | 649876.6 | 652598.6 | 259777.3 | 246281 | 259009.2 |
| 209 | 91 | 92 | (3-Bromo-1-methylpropoxymethyl)benzene | 1499 | 13974.97 | 14689.74 | 13665.9 | 10504.06 | 11959.48 | 9276.249 | 61015 | 69210.31 | 62865.72 | 29495.86 | 32260.72 | 23802.28 |
| 210 | 205 | 57 | Butylated Hydroxytoluene | 1513 | 9793.267 | 9900.758 | 9315.322 | 18297.35 | 19794.68 | 15252.23 | 58235.39 | 56315.38 | 60367.03 | 22975.2 | 22949.82 | 24419.36 |
| 211 | 198 | 183 | Dibenzofuran, 2-methoxy- | 1672 | 17954.57 | 18318.37 | 20641.19 | 20518.47 | 21327.92 | 16062.72 | 35752.29 | 36622.02 | 32463.84 | 20835.69 | 22988.54 | 21902 |
| 212 | 41 | 93 | .beta.-Myrcene | 991 | 1667295 | 1719215 | 1818689 | 2772665 | 2990540 | 2310187 | 1342162 | 1411779 | 1256264 | 1567300 | 1739784 | 1730654 |
| 213 | 98 | 70 | 2-Piperidinimine | 824 | 415922.2 | 418019.3 | 421073.7 | 540545 | 598760.4 | 520111.4 | 354692.2 | 309375.4 | 331602.8 | 438413.2 | 459607.9 | 421515.8 |
| 214 | 91 | 106 | Ethylbenzene | 855 | 70426.97 | 68703.63 | 66624.92 | 71992.15 | 76952.76 | 57317.13 | 52718.89 | 48344.15 | 43853.57 | 51123.28 | 53846.48 | 50479.55 |
| 215 | 74 | 57 | Butanoic Acid, 2-methyl- | 861 | 529110.7 | 501849.8 | 521500.3 | 288889.2 | 296544.3 | 290706.9 | 597073.2 | 462272.8 | 476111.9 | 434356.8 | 481987.2 | 406133.1 |
| 216 | 74 | 87 | Octanoic acid, methyl ester | 1126 | 42991.62 | 42426.54 | 44748.64 | 46031.79 | 50653.32 | 38355.23 | 32276.22 | 32155.08 | 27523.4 | 36612.8 | 38773.66 | 37085.72 |
| 217 | 69 | 55 | 1-Octyl trifluoroacetate | 1059 | 78211.93 | 90764.32 | 88557.58 | 119740.1 | 123527.6 | 109695.2 | 74597.55 | 73844.47 | 69831.84 | 84169.92 | 93027 | 84826.3 |
| 218 | 138 | 95 | Benzene, 1,2-dimethoxy- | 1148 | 10342.59 | 11264.94 | 10902.14 | 41365.92 | 37081.1 | 32390.68 | 27721.76 | 25207.51 | 24594.81 | 21251.46 | 24806.52 | 22782.3 |
| 219 | 93 | 121 | Cyclohexanol, 1-methyl-4-(1-methylethylidene)- | 1197 | 3836987 | 3937956 | 3890784 | 4559007 | 4970248 | 4128498 | 2781970 | 2774898 | 2536943 | 3445064 | 3719796 | 3584165 |
| 220 | 41 | 69 | OxiranecarboxAldehyde, 3-methyl-3-(4-methyl-3-pentenyl)- | 1234 | 103441 | 111579.2 | 106988.9 | 175261.6 | 199816.2 | 142024.8 | 118699.6 | 124374.8 | 102101.5 | 108975.6 | 114192.6 | 118842 |
| 221 | 97 | 98 | Thiophene, 2-hexyl- | 1277 | 3843.006 | 4203.024 | 4323.929 | 9493.782 | 10635.4 | 8350.175 | 2197.312 | 3148.976 | 1546.723 | 4333.96 | 4519.476 | 4913.11 |
| 222 | 112 | 42 | 1,2,4,5-Tetrazine-3,6-diAmine | 1279 | 5831.066 | 7413.651 | 5685.361 | 10940.44 | 11209.36 | 9455.985 | 4297.619 | 5101.028 | 4175.637 | 4178.373 | 5504.012 | 5315.675 |
| 223 | 81 | 68 | Cyclohexane, 1-ethenyl-1-methyl-2,4-bis(1-methylethenyl)-, (1.alpha.,2.beta.,4.beta.)- | 1398 | 9074.316 | 10052.75 | 8655.699 | 9806.154 | 10247.92 | 7192.148 | 16759.84 | 16555.53 | 15875.25 | 9754.204 | 10908.48 | 11265.7 |
| 224 | 91 | 136 | Benzeneacetic acid, 2-methylpropyl ester | 1393 | 15633 | 17894.75 | 16809.86 | 15741.73 | 18678.09 | 14372.66 | 101786.3 | 105530.4 | 92316.79 | 41888.61 | 44945.14 | 44384.93 |
| 225 | 44 | 58 | Mexiletine | 1397 | 2347.747 | 4091.153 | 2209.112 | 4067.398 | 6306.627 | 2910.615 | 12235.02 | 10960.19 | 10471.11 | 6282.219 | 6048.821 | 6466.902 |
| 226 | 118 | 105 | (1R,1aR,2aS,5R,6R,6aS,7aS)-1,6,6a-trimethyldecahydro-1,2a-methanocyclopropa[b]naphthalen-5-ol | 1431 | 527.3263 | 438.0335 | 699.3416 | 813.8616 | 722.7281 | 736.8183 | 34256.12 | 35075.23 | 31452.78 | 11771.16 | 12737.98 | 13220.73 |
| 227 | 119 | 93 | trans-.alpha.-Bergamotene | 1435 | 2892.201 | 3654.159 | 3502.319 | 6072.632 | 7760.065 | 5457.684 | 218650 | 227171.2 | 203056.5 | 79508.29 | 83858.82 | 82693.05 |
| 228 | 85 | 71 | 1-iodo-Decane | 1433 | 9 | 9 | 9 | 1478.861 | 2280.884 | 1511.855 | 9737.842 | 10338.41 | 8211.625 | 3414.439 | 3451.69 | 3970.245 |
| 229 | 161 | 105 | (1S,4S,4aS)-1-Isopropyl-4,7-dimethyl-1,2,3,4,4a,5-hexahydronaphthalene | 1458 | 50766.15 | 53579.91 | 52119.36 | 48908.91 | 60870.27 | 43076.91 | 68702.31 | 72979.14 | 68628.9 | 50836.64 | 59493.43 | 59155.03 |
| 230 | 99 | 71 | 2,5-Octanedione | 984 | 48495.02 | 47708.18 | 48123.61 | 52752.94 | 56353.99 | 49305.6 | 29379.9 | 28681.55 | 29385.97 | 39489.34 | 44341.03 | 40181.24 |
| 231 | 42 | 55 | 1-Pentanol | 766 | 399221 | 394264.3 | 365160.2 | 412926.7 | 416577.9 | 352438.2 | 147377.5 | 98541.61 | 120720.3 | 272669.6 | 271181.4 | 258328 |
| 232 | 94 | 67 | Pyrazine, methyl- | 829 | 522205.2 | 466471 | 462489.7 | 248763.1 | 255171.7 | 204727.5 | 316985.3 | 295442.7 | 302363.7 | 330958.9 | 366498.7 | 315871.3 |
| 233 | 55 | 42 | Cyclohexanone | 894 | 112550.1 | 108665.6 | 109834 | 185328 | 191163.5 | 159096.8 | 90268.29 | 83774.01 | 82199.29 | 113368.8 | 118341.7 | 111991.2 |
| 234 | 57 | 85 | Nonane | 900 | 69864.08 | 70120.98 | 69416.13 | 118376.8 | 125023.8 | 98531.02 | 59298.17 | 54820.79 | 53410.04 | 65727.38 | 71466.25 | 70622.44 |
| 235 | 139 | 121 | 3,6-Heptadien-2-ol, 2,5,5-trimethyl-, (E)- | 1000 | 38044.22 | 39784.3 | 39705.8 | 47715.57 | 57793.41 | 37871.09 | 21129.45 | 21871.6 | 19441.76 | 28219.56 | 33416.91 | 29574.29 |
| 236 | 41 | 55 | (E)-2-Octenal | 1060 | 78122.65 | 104045.9 | 101545.3 | 140488.2 | 143704.3 | 118371 | 81071.38 | 83146.47 | 79256.55 | 86232.14 | 107566.8 | 99711.5 |
| 237 | 91 | 90 | Formic acid, phenylmethyl ester | 1079 | 30754.46 | 29885.46 | 32109.94 | 43156.77 | 50078.03 | 38656.1 | 33802.7 | 33312.99 | 36043.11 | 39469.15 | 29875.75 | 32291.59 |
| 238 | 123 | 138 | 3-Acetyl-2,5-dimethyl furan | 1099 | 105918.1 | 110190.7 | 116238.2 | 127592.9 | 133102.7 | 109924.4 | 102134.5 | 98936.4 | 97896.9 | 108122.5 | 117743.1 | 104933.5 |
| 239 | 57 | 71 | Undecane | 1100 | 342772.6 | 347075.9 | 347409 | 445076.4 | 469722 | 392700.6 | 359029 | 365294.7 | 339612 | 347535.4 | 362537.4 | 345972.5 |
| 240 | 107 | 135 | Bicyclo[3.1.1]hept-3-en-2-one, 4,6,6-trimethyl-, (1S)- | 1204 | 247156.8 | 257774.3 | 261459.4 | 195390.4 | 206559 | 172211.2 | 255874.1 | 254671.3 | 238561.9 | 224506.8 | 245559.1 | 227525.8 |
| 241 | 121 | 93 | Formamide, N-phenyl- | 1221 | 9359.815 | 13015.69 | 11998.39 | 18031.78 | 18042.82 | 16277.31 | 11379.37 | 11448.86 | 9516.638 | 10410.87 | 13884.62 | 11364.64 |
| 242 | 95 | 41 | 2,6-Dimethyl-1-nonen-3-yn-5-ol | 1300 | 3202.613 | 2581.312 | 2378.394 | 6544.783 | 7686.509 | 6481.755 | 3916.129 | 2657.639 | 2878.883 | 4394.495 | 4269.774 | 3625.293 |
| 243 | 121 | 136 | 3-Cyclohexene-1-methanethiol, .alpha.,.alpha.,4-trimethyl- | 1283 | 8621.968 | 11561.6 | 9989.434 | 9801.753 | 13397.9 | 8589.486 | 4231.405 | 5168.159 | 4411.109 | 6006.311 | 6014.471 | 7609.098 |
| 244 | 133 | 91 | (3S,3aS,6R,8aS)-3,8,8-Trimethyl-7-methyleneoctahydro-1H-3a,6-methanoazulene | 1444 | 2692.308 | 1603.726 | 1012.158 | 1530.196 | 2335.525 | 1718.194 | 385948.7 | 388066.8 | 354934.9 | 126888.4 | 136560.3 | 133187.1 |
| 245 | 95 | 55 | Cyclohexanepropanoic Acid, 2-propenyl ester | 1435 | 6047.621 | 6427.589 | 5560.914 | 7957.701 | 10028 | 8173.034 | 188354.6 | 193547 | 173308.6 | 66933.83 | 71552.28 | 71274.38 |
| 246 | 81 | 41 | Naphthalene, decahydro-1,6-bis(methylene)-4-(1-methylethyl)-, (4.alpha.,4a.alpha.,8a.alpha.)- | 1459 | 56691.06 | 58556.97 | 60570.13 | 39105.82 | 48643.57 | 39274.56 | 79716.79 | 80470.56 | 81971.22 | 70297.96 | 70845.98 | 66131.6 |
| 247 | 68 | 67 | 1-Cyclohexene-1-carboxylic acid, 4-(1-methylethenyl)- | 1484 | 2387.883 | 1417.339 | 1441.033 | 1141.552 | 1757.799 | 1425.134 | 11339.06 | 11258.86 | 10992.89 | 4460.722 | 5394.308 | 4378.984 |
| 248 | 191 | 131 | Benzeneacetic acid, 4-(1,1-dimethylethyl)-, methyl ester | 1491 | 8289.989 | 7422.906 | 8932.65 | 10297.71 | 10776.2 | 9227.079 | 396146.2 | 378386.7 | 385652.5 | 123261 | 119340.2 | 123217.7 |
| 249 | 57 | 41 | Tridecanal | 1513 | 18910.26 | 19225.73 | 18212.68 | 20216.4 | 22930.59 | 16893.1 | 37767.4 | 38054.37 | 34284.95 | 19836.46 | 19969.51 | 21330.21 |
| 250 | 93 | 119 | (Z)-1-Methyl-4-(6-methylhept-5-en-2-ylidene)cyclohex-1-ene | 1515 | 18045.9 | 17517.99 | 17466.78 | 10841.61 | 11099.91 | 9168.833 | 143161 | 145738.6 | 148292.3 | 47477.04 | 49431.06 | 47417.61 |
| 251 | 55 | 69 | 1-Dodecanol | 1474 | 47336.84 | 46578.84 | 48060.21 | 8050.498 | 8114.726 | 6530.798 | 193474.9 | 189441.7 | 171651.3 | 74069.51 | 81592.8 | 78424.6 |
| 252 | 157 | 142 | .alpha.-Calacorene | 1542 | 9759.582 | 11017.2 | 9862.008 | 13156.16 | 13671.72 | 10476.26 | 20834.56 | 22800.36 | 22570.14 | 16265.03 | 15074.7 | 13943.97 |
| 253 | 57 | 43 | Decane | 1000 | 39588.6 | 54749.09 | 69225.5 | 89135.34 | 100358.2 | 98852.78 | 36642.09 | 57987.59 | 54826.11 | 48357.43 | 62263.91 | 66807.51 |
| 254 | 57 | 71 | Undecane, 2,9-dimethyl- | 1233 | 276350.8 | 288104.2 | 282856.9 | 493337.2 | 569438.4 | 377115.7 | 327666 | 335639.3 | 288943.8 | 302541.4 | 316192.4 | 327502.7 |
| 255 | 42 | 55 | 2-Oxepanone | 1065 | 36523.66 | 39779.6 | 38739.84 | 50682.63 | 60381.29 | 50194.7 | 15251.17 | 14843.86 | 13957.82 | 34468.44 | 34945 | 29735.5 |
| 256 | 139 | 43 | 2H-Pyran, 2-ethenyltetrahydro-2,6,6-trimethyl- | 972 | 11619.01 | 12184.44 | 11356.79 | 10681.77 | 11548.59 | 8641.522 | 7625.644 | 7826.719 | 7342.261 | 8568.26 | 9828.11 | 9179.984 |
| 257 | 120 | 61 | 1,4-Dithiane | 1068 | 54324.25 | 60212.53 | 58431.63 | 52042.48 | 54501.86 | 44491.85 | 62015.96 | 62988.88 | 59101.18 | 53711.59 | 60491.34 | 56530.14 |
| 258 | 135 | 136 | Pyrazine, 3-ethyl-2,5-dimethyl- | 1081 | 162703.6 | 175940.5 | 180230.4 | 81988.72 | 78966.46 | 64631.98 | 108998.6 | 101779.7 | 99324.59 | 111228.6 | 128329.9 | 110641.7 |
| 259 | 105 | 77 | Benzoic acid, methyl ester | 1094 | 705948.8 | 704792.5 | 732945.5 | 980545.8 | 1020232 | 854948.6 | 675483.9 | 659212.6 | 643408.8 | 724143.2 | 792326.7 | 717893.9 |
| 260 | 121 | 77 | Benzene, (1-methoxypropyl)- | 1104 | 2816916 | 2835660 | 2925753 | 4027284 | 4178874 | 3485202 | 2660026 | 2553612 | 2444097 | 2801727 | 3203660 | 2917652 |
| 261 | 41 | 43 | 2-Isopropyl-5-methylhex-2-enal | 1106 | 403835.5 | 403397.9 | 414170.1 | 523701.3 | 535437.7 | 441966.5 | 374642.1 | 361987.6 | 337907 | 410181.8 | 435144.6 | 414376.2 |
| 262 | 70 | 85 | Butanoic acid, 3-methyl-, 2-methylbutyl ester | 1107 | 10670.53 | 10248.7 | 10241.82 | 18738.02 | 16225.18 | 12609.57 | 78885.03 | 80628.53 | 74123.08 | 32917.19 | 36334.73 | 34462.36 |
| 263 | 55 | 69 | Bicyclo[3.1.1]heptan-3-one, 2,6,6-trimethyl-, (1.alpha.,2.beta.,5.alpha.)- | 1173 | 838959.7 | 854344.1 | 816743.8 | 962093.4 | 1004798 | 906187.8 | 903664.5 | 854889.6 | 809738.5 | 856015.3 | 905833.1 | 836712.2 |
| 264 | 82 | 138 | Isophorone | 1124 | 17093.95 | 16974.79 | 17335.04 | 16726.92 | 16355.86 | 13668.06 | 19934.06 | 19071.27 | 18011.04 | 16762.65 | 18845.92 | 16844.47 |
| 265 | 98 | 71 | Azacyclohexan-3-one, 1,5,6-trimethyl- | 1126 | 35910.14 | 35368.32 | 37919.68 | 64661.69 | 66423.53 | 50859.74 | 32164.92 | 29712.19 | 28727.65 | 35776.27 | 42261.15 | 39181.32 |
| 266 | 70 | 43 | Acetic acid, 2-ethylhexyl ester | 1129 | 10928.89 | 9812.151 | 9978.349 | 31432.85 | 33433.32 | 24833.79 | 8572.487 | 7207.409 | 6490.251 | 12376.27 | 13742.44 | 12489.23 |
| 267 | 79 | 81 | (Z)-2,2-Dimethyl-3-(3-methylpenta-2,4-dien-1-yl)oxirane | 1133 | 57519.94 | 60505.59 | 61927.07 | 90098.82 | 96915.33 | 72638.66 | 54878.95 | 59950.2 | 51803.76 | 52597.2 | 61800.85 | 63140.03 |
| 268 | 70 | 55 | 1-Decen-3-one | 1141 | 17279.2 | 17372.01 | 16869.46 | 21349.88 | 22350.41 | 20121.91 | 64511.17 | 62345.51 | 62809.4 | 31794.08 | 36165.58 | 32749.98 |
| 269 | 70 | 55 | 1-Undecene, 9-methyl- | 1152 | 54951.49 | 58077.03 | 56124.89 | 90885.28 | 91923.06 | 75852.88 | 46395.93 | 42211.5 | 36889.22 | 56475.17 | 61191.13 | 56791.02 |
| 270 | 68 | 67 | 2H-Pyran, 3,6-dihydro-4-methyl-2-(2-methyl-1-propenyl)- | 1154 | 93604.58 | 93488.38 | 96133.29 | 111023.2 | 115751.2 | 95526.29 | 73724.64 | 70420.42 | 64672.12 | 84237.88 | 89385.66 | 82319.39 |
| 271 | 56 | 55 | 1-Nonanol | 1173 | 193894.5 | 206904.1 | 196004.8 | 234284 | 250732.5 | 223614.2 | 210785.1 | 201927.6 | 196733.6 | 196307.8 | 211486.2 | 203393.5 |
| 272 | 106 | 78 | 4-Pyridinecarboxylic acid, ethyl ester | 1201 | 38780.63 | 40550.68 | 40671.99 | 35770.61 | 35839.38 | 32046.69 | 40854.15 | 40644.57 | 41738.44 | 38739.46 | 42245.65 | 36310.44 |
| 273 | 57 | 85 | Butanethioic acid, 3-methyl-, S-(1-methylpropyl) ester | 1174 | 335527.7 | 327007 | 336450.7 | 388824 | 410509.7 | 361349.1 | 335630.5 | 360074.1 | 326141.8 | 342238.6 | 361932.3 | 341614.2 |
| 274 | 95 | 107 | 2-Methylisoborneol | 1198 | 418862.2 | 443897.8 | 438447 | 474852.6 | 504333.4 | 428463.8 | 398179.5 | 388470.8 | 359043.9 | 416546.5 | 434479.5 | 417187.8 |
| 275 | 109 | 96 | 3,5-Dimethyl-1-butylpyrazole | 1192 | 108227 | 112429 | 115880 | 134175 | 151372 | 103044 | 77390 | 72018 | 69028 | 97269 | 102239 | 106746 |
| 276 | 108 | 67 | 2-Nonenenitrile | 1194 | 10105.67 | 10691.96 | 10993.54 | 16865.54 | 20479.98 | 17110.03 | 11946.76 | 10769.49 | 10657 | 13425.86 | 14669.46 | 11134.37 |
| 277 | 82 | 42 | Tropinone | 1198 | 51620.64 | 55527.13 | 54888.49 | 101916.6 | 112223 | 84103.21 | 48126.15 | 48141.71 | 40586.85 | 54595.62 | 59908.48 | 57067.06 |
| 278 | 93 | 81 | o-Mentha-1(7),8-dien-3-ol | 1201 | 216547.8 | 226333.9 | 224841.9 | 231120.8 | 251128.7 | 202131.5 | 114214.4 | 135973.4 | 122518.1 | 168534.8 | 180300.1 | 176194.2 |
| 279 | 69 | 41 | 6-Octen-1-ol, 3,7-dimethyl-, (R)- | 1220 | 94230.96 | 95458.74 | 92073.85 | 112669.4 | 126267.6 | 101465.7 | 89560.73 | 85442.8 | 75859.71 | 92586.81 | 97461.39 | 88158.06 |
| 280 | 94 | 151 | Amantadine | 1231 | 23878.65 | 25843.76 | 24741.73 | 26151.4 | 25719.67 | 22889.24 | 30758.95 | 30989.61 | 28894.51 | 25792.81 | 25781.07 | 24936.37 |
| 281 | 70 | 55 | 1-Butanol, 3-methyl-, acetate | 876 | 17407.72 | 17505.32 | 17464.22 | 47767.92 | 48062.09 | 40346.37 | 28468.71 | 23690.24 | 25118.3 | 25103.64 | 26832.09 | 24480.38 |
| 282 | 41 | 68 | 4-Heptenal, (Z)- | 900 | 94696.05 | 99467.51 | 98716.43 | 157168.1 | 164989.4 | 129680.9 | 68993.96 | 65180.89 | 61979.24 | 89490.17 | 99183.84 | 91264.1 |
| 283 | 70 | 41 | Heptanal | 903 | 154637.2 | 149698.7 | 154185.5 | 258855.1 | 264601.1 | 222745.4 | 126382.8 | 118000.6 | 114588.1 | 156656.3 | 173344.7 | 159815.8 |
| 284 | 93 | 91 | .alpha.-Pinene | 937 | 107787.8 | 82857.01 | 72445.41 | 21532.41 | 21295.24 | 14906.03 | 469316.6 | 454937.5 | 427198.3 | 200357.7 | 212042.5 | 121556.5 |
| 285 | 80 | 79 | Bicyclo(3.3.1)non-2-ene | 964 | 24715.5 | 24688.65 | 24519.8 | 38079.81 | 41229.68 | 43166.51 | 16214.22 | 16938.65 | 16620.93 | 24545.14 | 24149.35 | 22793.74 |
| 286 | 110 | 40 | Pyrazine, methoxy- | 904 | 81214.63 | 79239.47 | 76984.77 | 34304.43 | 31620.48 | 30115.26 | 38420.72 | 30592.47 | 31192.38 | 52170.32 | 56894.94 | 44041.27 |
| 287 | 91 | 106 | Cyclobutanespiro-2'-bicyclo[1.1.0]butane-4'-spirocyclobutane | 930 | 46012.38 | 35352.55 | 31475.18 | 11317.59 | 10010.22 | 6965.693 | 201243.2 | 197248.9 | 184880 | 86520.86 | 94277.67 | 53202.4 |
| 288 | 41 | 69 | Cyclopentane, butyl- | 937 | 18712.16 | 13374.79 | 11638.61 | 3672.569 | 4148.801 | 3371.397 | 31979.9 | 28067.15 | 26003.66 | 17101.11 | 16753.4 | 10300.59 |
| 289 | 68 | 79 | 1,7-Octadiene, 2-methyl-6-methylene- | 984 | 102450.2 | 98758.07 | 101104 | 117669.7 | 122985.9 | 104810.4 | 96496.94 | 79195.39 | 80740.97 | 94302.73 | 95900.61 | 86768.12 |
| 290 | 67 | 41 | 3-Hexen-1-ol, (Z)- | 858 | 827668.1 | 759754.4 | 785528.3 | 925069.2 | 967705 | 864044.3 | 521211.9 | 433549.8 | 434716.6 | 674852.9 | 713739.3 | 648089.5 |
| 291 | 93 | 66 | Pyridine, 2-methyl- | 816 | 18299.9 | 15540.24 | 15115.72 | 6501.946 | 7588.358 | 6819.177 | 11325.21 | 9593.868 | 9892.667 | 10869.2 | 11660.16 | 9688.294 |
| 292 | 58 | 71 | 2-Heptanone | 895 | 255890.1 | 246368.9 | 254741.7 | 307642.2 | 319958.5 | 267427.9 | 91210.62 | 85580.61 | 81421.19 | 185890.1 | 203914.1 | 183073.4 |
| 293 | 42 | 41 | 1,2,4,5-Tetrazin-3-Amine | 967 | 16931.27 | 15397.52 | 10624.39 | 10463.61 | 10637.49 | 9930.033 | 6407.868 | 3999.848 | 3239.708 | 10066.42 | 7964.581 | 6051.497 |
| 294 | 45 | 41 | Diallyl Sulfur compounds | 861 | 4737.349 | 7151.253 | 9002.662 | 12768.79 | 14869.98 | 12302.47 | 5430.756 | 4222.7 | 5971.267 | 5625.795 | 5274.155 | 5640.143 |
| 295 | 55 | 56 | 2-Propenoic acid, butyl ester | 861 | 175078.5 | 163219.9 | 180921.3 | 247664.1 | 229142.2 | 207629.8 | 92008.6 | 78982.98 | 77797.3 | 146279.9 | 156538.8 | 139716.4 |
| 296 | 84 | 68 | Dicyandiamide | 866 | 25358.89 | 26283.82 | 24516.06 | 33658.88 | 32736.26 | 27742.52 | 12919.35 | 11635.64 | 12733.24 | 19943.26 | 25493.9 | 20125.81 |
| 297 | 55 | 70 | 1-Hepten-3-one | 881 | 8398.17 | 10506.99 | 11546.01 | 33969.84 | 32641.93 | 29591.75 | 18808 | 19153.33 | 14953.58 | 13880.05 | 19123.83 | 16441.45 |
| 298 | 74 | 43 | Pentanoic acid, 4-methyl-, methyl ester | 881 | 21404 | 19109 | 17958.99 | 16021 | 17392 | 18333 | 16136 | 11364 | 10307 | 16983 | 14240.89 | 13263 |
| 299 | 91 | 106 | o-Xylene | 888 | 76981.04 | 74904.11 | 75483.74 | 77790.93 | 87076.41 | 64275.21 | 57511 | 54614.12 | 47998.47 | 57209.9 | 63333.45 | 57564.31 |
| 300 | 95 | 110 | Ethanone, 1-(2-furanyl)- | 910 | 182168.2 | 171949.3 | 167031 | 88411.1 | 89803.21 | 82715.32 | 83635.46 | 66490.36 | 69811.29 | 117141.6 | 127530.4 | 101432.4 |
| 301 | 104 | 103 | Styrene | 893 | 442506 | 433478.5 | 428078.8 | 422310.5 | 446671.9 | 368149.1 | 314431.8 | 289422.2 | 271270.5 | 337114.1 | 371767.6 | 328039.1 |
| 302 | 68 | 43 | 4-Pentenyl Acetate | 896 | 51559.24 | 50528.77 | 53445.93 | 83531.37 | 85269.74 | 74049.79 | 40346.87 | 36735.42 | 34285.67 | 53869.13 | 57888.65 | 52413.51 |
| 303 | 67 | 68 | 5-Hexenal, 4-methylene- | 897 | 33225.13 | 31636.96 | 33122.7 | 52973.11 | 52885.89 | 46369.65 | 24970.33 | 22324.7 | 21232.22 | 33775.08 | 36560.31 | 32566.26 |
| 304 | 71 | 43 | Propanoic acid, 2-methyl-, 2-methylpropyl ester | 910 | 64905.7 | 65365.23 | 65650.26 | 91339.26 | 92227.2 | 79066.12 | 44486.94 | 41884.22 | 39273.92 | 56445.77 | 62874.17 | 59087.26 |
| 305 | 81 | 39 | 2,4-Hexadienal, (E,E)- | 910 | 106622.2 | 100635.2 | 89554.66 | 162475.1 | 157147.3 | 151633.6 | 60363.22 | 46189.88 | 49722.06 | 91407.66 | 107051.6 | 76223.66 |
| 306 | 70 | 61 | Acetic acid, pentyl ester | 910 | 46678.31 | 46280.48 | 45498.29 | 105178.9 | 106574.6 | 89527.94 | 17584.95 | 17201.93 | 16240.49 | 46988.91 | 50186.69 | 45028.88 |
| 307 | 57 | 71 | 3-Ethyl-3-methylheptane | 953 | 45107.32 | 38771.25 | 38754.88 | 43585.95 | 45980.85 | 37878.64 | 36154.2 | 31473.41 | 30321.73 | 33556.37 | 35310.38 | 32452.84 |
| 308 | 42 | 86 | Butanoic Acid, 4-hydroxy- | 933 | 110525.3 | 108398.5 | 100415.5 | 39309.21 | 37884.51 | 45789.28 | 92617.43 | 68927.81 | 73187.08 | 87035.86 | 88231.71 | 69858.25 |
| 309 | 105 | 120 | Benzene, 1,2,4-trimethyl- | 990 | 103203.5 | 107286 | 111978.6 | 142795.3 | 159205.8 | 118006.4 | 86964.12 | 97030.03 | 93597.76 | 89776.33 | 108521 | 100700.7 |
| 310 | 41 | 69 | 2-Pentenoic Acid, 2-methyl- | 974 | 10895.74 | 10150.35 | 11561.52 | 9 | 9 | 9 | 233326.5 | 253034.6 | 234368.6 | 32510.65 | 41403.05 | 35619.4 |
| 311 | 61 | 43 | Glycerin | 967 | 6991.533 | 6665.727 | 7894.12 | 11511.37 | 12220.86 | 11134.76 | 5128.803 | 5576.332 | 4980.304 | 6777.603 | 8058.864 | 7304.309 |
| 312 | 121 | 105 | 1,3-Cyclohexadiene, 1,5,5,6-tetramethyl- | 976 | 29811.73 | 30814.79 | 29739.19 | 6114.437 | 7518.976 | 5186.027 | 227583.5 | 242229.7 | 232872.7 | 64753.7 | 76369.88 | 62509.64 |
| 313 | 69 | 41 | 5-Methyl-(E)-2-hepten-4-one | 972 | 20238.83 | 18996.53 | 19039.72 | 17540.08 | 18246.52 | 14524.46 | 4498.653 | 5165.691 | 4474.119 | 11126.6 | 12198.29 | 9878.005 |
| 314 | 93 | 91 | Bicyclo[3.1.0]hexane, 4-methylene-1-(1-methylethyl)- | 972 | 220596.9 | 226707.1 | 202561.4 | 9 | 9 | 9 | 1867895 | 2026206 | 1918635 | 493088.4 | 585079.8 | 476569.9 |
| 315 | 126 | 45 | Dimethyl triSulfur compounds | 971 | 159817.6 | 164425.7 | 167026.7 | 256224.3 | 277748.4 | 269730.3 | 116542.1 | 120328.8 | 121215.5 | 155755.8 | 161315.5 | 153425.4 |
| 316 | 107 | 51 | 4-Pyridinecarboxaldehyde | 976 | 16006.47 | 14472.56 | 15072.88 | 5699.216 | 6750.579 | 4589.342 | 94847.49 | 104110.9 | 94599.95 | 28060.86 | 33246.76 | 26628.1 |
| 317 | 69 | 41 | 2-Propenoic acid, 2-methyl-, 2-chloroethyl ester | 977 | 63108.26 | 62312.54 | 59790.88 | 9 | 9 | 9 | 415267.4 | 447946.2 | 425227.3 | 128192 | 145242.9 | 126079.6 |
| 318 | 93 | 41 | .beta.-Pinene | 981 | 86057.52 | 78995.99 | 82016.87 | 96138.99 | 99916 | 82863.44 | 53559.24 | 49590.65 | 48164.87 | 71681.6 | 73681.83 | 67067.1 |
| 319 | 74 | 87 | Heptanoic acid, methyl ester | 1023 | 92161.67 | 97803.34 | 87137.7 | 88204.18 | 103037.3 | 108344.4 | 85502.89 | 80733.69 | 70758.8 | 71210.49 | 92809.87 | 80960.78 |
| 320 | 136 | 94 | DiSulfur compounds, ethyl 1-methylethyl | 985 | 123441 | 130244.6 | 147444 | 200328.6 | 211824.1 | 162646.4 | 112914.8 | 135961.6 | 118547.1 | 125977.8 | 146676.2 | 129696.1 |
| 321 | 69 | 41 | cis-2,6-Dimethyl-2,6-octadiene | 985 | 251338 | 255154.2 | 261997.5 | 294434.2 | 311227.3 | 284836.1 | 191106.8 | 191621.4 | 193310.1 | 229203.9 | 241181.1 | 228437.4 |
| 322 | 111 | 126 | Isomaltol | 989 | 29800.36 | 28193.01 | 32680.9 | 60033.77 | 64488.72 | 50839.24 | 16727.78 | 18638.14 | 16957.7 | 28896.58 | 34898.28 | 28758.65 |
| 323 | 69 | 41 | 2,6-Dimethyl-2-trans-6-octadiene | 993 | 1873534 | 1959476 | 2087691 | 3157729 | 3322439 | 2550635 | 1605975 | 1900531 | 1749530 | 1842116 | 2072537 | 2065320 |
| 324 | 109 | 79 | 2,3-Dehydro-1,8-cineole | 992 | 63083.09 | 64266.38 | 58412.65 | 76109.55 | 89022.09 | 61072.31 | 37566.54 | 38781.09 | 36196.05 | 50132.54 | 57648.03 | 51783.56 |
| 325 | 105 | 134 | Benzene, (1-methylpropyl)- | 1001 | 12194.35 | 13379.21 | 14847.36 | 18737.79 | 16948.71 | 13644.2 | 11800.81 | 12729.34 | 11409.51 | 12731.76 | 15818.47 | 12333.29 |
| 326 | 109 | 79 | Ethanone, 1-(2-methyl-1-cyclopenten-1-yl)- | 996 | 16467.18 | 17658.9 | 19856.42 | 23832.37 | 23354.98 | 18080.38 | 8532.353 | 10912.06 | 10480.22 | 14789.2 | 18849.58 | 13351.13 |
| 327 | 128 | 58 | 2-Octanone | 991 | 8697 | 9377 | 8287.753 | 10847.48 | 12000 | 10122.45 | 8450.301 | 8096.567 | 7298.21 | 8813 | 10290.35 | 8364.574 |
| 328 | 74 | 87 | Hexanoic acid, methyl ester | 925 | 136843.7 | 130742.6 | 130143 | 82602.34 | 82980.18 | 70938.15 | 42652.78 | 37577.28 | 39643.16 | 76112.28 | 82747.1 | 75131.02 |
| 329 | 122 | 107 | Benzene, 1-methoxy-2-methyl- | 1008 | 12443.83 | 12254.11 | 12846.8 | 18292.12 | 20898.78 | 16154.77 | 10245.06 | 10893.35 | 10036.52 | 10422.45 | 13857.99 | 11281.22 |
| 330 | 67 | 68 | 2-Cyclopentylethanol | 1003 | 124945.5 | 88214.78 | 88236.29 | 56373.33 | 52679.04 | 40496.57 | 122424 | 65137.12 | 60605.11 | 65340.71 | 82592.7 | 67394.11 |
| 331 | 82 | 67 | 3-Hexen-1-ol, acetate, (E)- | 1005 | 31824.11 | 32561.8 | 32449.55 | 23576.93 | 23401.97 | 17892.08 | 30024.81 | 29889.16 | 28046.39 | 24038.04 | 27682.53 | 27378.23 |
| 332 | 82 | 67 | 3-Hexen-1-ol, acetate, (Z)- | 1009 | 75134.19 | 79253.16 | 80320.33 | 165594.6 | 167187 | 146375.7 | 57053.75 | 52998.61 | 53778.4 | 80392.38 | 94802.68 | 87385.19 |
| 333 | 83 | 42 | 2-Dimethylamino-4-methyl-pent-4-enenitrile | 1014 | 4149.601 | 5374.008 | 5101.767 | 11775.69 | 12654 | 10118.79 | 3279.9 | 3323.882 | 2937.989 | 4903.88 | 5756.982 | 5152.884 |
| 334 | 117 | 118 | Indane | 1029 | 44178.78 | 47745.36 | 45244.99 | 40625.26 | 43453.92 | 32851.05 | 51644.23 | 51035.43 | 47189.66 | 40498.12 | 48589.37 | 42737.84 |
| 335 | 57 | 41 | 1-Hexanol, 2-ethyl- | 1030 | 124908.2 | 128011.5 | 111409.2 | 134710.5 | 170373 | 162101.6 | 329211.2 | 306303.2 | 271397.9 | 160623.5 | 185833.1 | 166613.4 |
| 336 | 111 | 55 | 3-Octen-2-one, (E)- | 1035 | 194279.5 | 196734.5 | 193195.2 | 332089.6 | 342098.4 | 298885.3 | 100886.3 | 84526.98 | 80972.83 | 194640.9 | 216178.1 | 165359.8 |
| 337 | 74 | 75 | Acetic acid, (propylthio)-, methyl ester | 1036 | 325133.2 | 327623.9 | 324959 | 351362.1 | 360301 | 338901.2 | 329674.3 | 315941.6 | 299355.8 | 318248.8 | 345592.6 | 301658.3 |
| 338 | 55 | 111 | 3-Octen-2-one | 1040 | 355020.7 | 351540.6 | 349854.6 | 590819.6 | 610642.6 | 535461.9 | 207913 | 198800.8 | 183393.7 | 336033.8 | 360769.4 | 328441.4 |
| 339 | 92 | 65 | 1H-Pyrrole-3-carbonitrile | 1044 | 2027736 | 2058785 | 2024294 | 2874383 | 2962958 | 2625978 | 1623596 | 1593411 | 1511036 | 1964612 | 2253055 | 1867953 |
| 340 | 57 | 41 | 1-Hexanol, 3,5,5-trimethyl- | 1047 | 9843.949 | 10496.19 | 8647.38 | 10181.07 | 12343.19 | 7136.062 | 16232.32 | 15856.33 | 14115.22 | 7528.42 | 8299.098 | 9283.989 |
| 341 | 57 | 43 | Decane, 5-methyl- | 1057 | 128421.6 | 142796.6 | 139269.8 | 182100.7 | 203662.7 | 147978.2 | 137724.4 | 142013.7 | 123945.2 | 126529.8 | 132245.7 | 128569.5 |
| 342 | 93 | 77 | .beta.-Phellandrene | 1031 | 746911.5 | 799642.3 | 789879.1 | 1028294 | 1145530 | 821749.1 | 603527.8 | 648010.6 | 605347.2 | 683256.1 | 793778.8 | 694113.4 |
| 343 | 45 | 41 | 7-Octen-2-ol | 969 | 34924.5 | 35410.23 | 35841.19 | 54160.25 | 55568.76 | 54609.92 | 24584.14 | 25192.35 | 23840.42 | 34665.91 | 35432.37 | 32544.02 |
| 344 | 68 | 43 | 1H-Imidazole, 1-acetyl- | 950 | 4239.585 | 3765.375 | 3573.18 | 3550.04 | 3993.433 | 3267.075 | 7222.85 | 7261.731 | 6469.788 | 4994.901 | 6288.493 | 4212.788 |
| 345 | 42 | 41 | 2,5-Furandione, dihydro-3-methyl- | 1057 | 59129.23 | 63564.26 | 65706.94 | 80678.31 | 82811.21 | 70961.05 | 52538.25 | 53787.21 | 47847.03 | 58826.93 | 65213.26 | 58352.33 |
| 346 | 91 | 119 | BenzAldehyde, 2-methyl- | 1064 | 40201.82 | 42368.64 | 42265.67 | 63271.79 | 68492.66 | 56708.05 | 22703.66 | 26229.93 | 22467.58 | 37240.05 | 43798.11 | 40396.27 |
| 347 | 81 | 82 | 2-Ethoxy-3-methylpyrazine | 1065 | 440573 | 452711.2 | 455961.8 | 1128944 | 1172965 | 977386.6 | 270293.9 | 217203.9 | 206427.6 | 513689.6 | 562653.7 | 515077.1 |
| 348 | 93 | 91 | .gamma.-Terpinene | 1060 | 115037 | 105933 | 118030 | 150015 | 166163 | 115255 | 84830 | 100592 | 91403 | 92060 | 105248 | 99973 |
| 349 | 124 | 123 | 2,5-FurandicarboxAldehyde | 1076 | 321383.3 | 356909.6 | 345953.3 | 767552.2 | 797507.8 | 695274.3 | 207771.7 | 202475.6 | 197368.5 | 378033.3 | 432537.4 | 373644 |
| 350 | 41 | 55 | 1,10-Undecadiene | 1095 | 3091083 | 3113272 | 3155816 | 4102339 | 4197754 | 3616003 | 2966059 | 2895157 | 2791675 | 3182389 | 3296247 | 3176184 |
| 351 | 135 | 136 | Pyrazine, 2-ethyl-3,5-dimethyl- | 1084 | 26154 | 30402 | 29823 | 11275 | 10930 | 11047 | 22378 | 19197 | 17735 | 19163 | 20998 | 17637 |
| 352 | 83 | 101 | 2-Butenoic acid, 2-methyl-, 2-methylpropyl ester, (E)- | 1093 | 2456799 | 2454208 | 2515132 | 3183961 | 3305380 | 2784316 | 2363022 | 2290779 | 2216030 | 2477115 | 2646528 | 2453338 |
| 353 | 70 | 82 | 1-(2-Hydroxyethyl)-1,2,4-triazole | 1095 | 788908.9 | 782935 | 800717.7 | 1038780 | 1098498 | 909071.4 | 778128.1 | 758395.2 | 731745.9 | 823010.5 | 855102.3 | 805162.1 |
| 354 | 82 | 81 | 1H-Imidazole, 2-propyl- | 1095 | 1132626 | 1133646 | 1162050 | 1418331 | 1475978 | 1228479 | 1103217 | 1071986 | 1040618 | 1150213 | 1205049 | 1126613 |
| 355 | 117 | 99 | Hexanoic acid, propyl ester | 1097 | 25797.31 | 26967.61 | 29113.75 | 38548.38 | 39739.53 | 32992.12 | 29994.74 | 27946.13 | 28927.86 | 27823.3 | 32399.34 | 31676.88 |
| 356 | 68 | 69 | 7-Octen-4-ol, 2-methyl-6-methylene-, (S)- | 1097 | 1459412 | 1443269 | 1485027 | 1977889 | 2040898 | 1727457 | 1356218 | 1339048 | 1294024 | 1473499 | 1559040 | 1475651 |
| 357 | 88 | 43 | Heptanoic acid, ethyl ester | 1098 | 13871.27 | 13212.32 | 13606.71 | 16384.66 | 16642.39 | 14538.32 | 12343.96 | 12322.2 | 11538.14 | 14330.79 | 14327.58 | 12738.64 |
| 358 | 67 | 82 | 3-Cyclopentyl-1-propanol | 1102 | 1065886 | 1035047 | 1037792 | 1345406 | 1395284 | 1195766 | 754809.5 | 731410.2 | 693651.1 | 974506.3 | 1075227 | 975357.1 |
| 359 | 55 | 83 | 1-Nonen-4-ol | 1103 | 423397.5 | 409897.1 | 413179 | 551656.7 | 570900.1 | 464565.7 | 358440.3 | 351713.3 | 280108.2 | 381643.3 | 419530 | 390724.9 |
| 360 | 57 | 41 | 2-Octen-1-ol, (E)- | 1067 | 32300.75 | 36390.94 | 36119.28 | 34489.34 | 35382.92 | 30838.68 | 20875.06 | 19276.47 | 17676.84 | 29703.28 | 32783.55 | 27940.33 |
| 361 | 124 | 109 | Benzene, (methylthio)- | 1106 | 17759.51 | 16220.54 | 16667.21 | 12310.82 | 14168.54 | 10508.05 | 19451.81 | 18413.49 | 15525.88 | 15061.06 | 15561.48 | 15998.35 |
| 362 | 80 | 79 | 2,6,6-Trimethylbicyclo[3.2.0]hept-2-en-7-one | 1108 | 4045139 | 3987320 | 4094559 | 5672394 | 5860980 | 4960725 | 3847852 | 3771346 | 3646826 | 4094364 | 4447139 | 4099760 |
| 363 | 73 | 45 | 3-Hexene, 1-(1-ethoxyethoxy)-, (Z)- | 1102 | 12873.74 | 13293.9 | 12923.03 | 17678.94 | 16365.99 | 13877.71 | 9672.942 | 9936.995 | 10508.03 | 11833.93 | 13774.41 | 11013.17 |
| 364 | 117 | 132 | Benzene, 1-methyl-4-(1-methylethenyl)- | 1090 | 88991.38 | 95152.2 | 95304.78 | 110741.9 | 108866.3 | 86263.39 | 171473.4 | 170610.8 | 159359 | 112329.9 | 121407.8 | 115902.5 |
| 365 | 71 | 43 | Butanoic acid, anhydride | 1120 | 44857.94 | 44758.63 | 43758.64 | 96372.07 | 103756.3 | 82953.8 | 36452.17 | 33745.39 | 32702.31 | 46956.2 | 53274.2 | 53194.65 |
| 366 | 69 | 81 | Furan, 3-(4-methyl-3-pentenyl)- | 1101 | 211610.3 | 208870.6 | 205119.3 | 260410.2 | 266286.3 | 247917 | 214645.4 | 210790.7 | 196556.5 | 214329.3 | 230501.4 | 223372.9 |
| 367 | 106 | 121 | BenzenAmine, N-ethyl- | 1128 | 18043.93 | 18560.49 | 20119.57 | 26167.32 | 27178.16 | 22158.6 | 15976.89 | 17476.13 | 16736.78 | 15526.75 | 19344.89 | 17777.48 |
| 368 | 91 | 119 | 2,6-Dimethyl-1,3,5,7-octatetraene, E,E- | 1131 | 147034.8 | 139562.6 | 170136.1 | 221730 | 270529.5 | 189165.5 | 129194.7 | 187267.9 | 163680.1 | 131747.6 | 168646.1 | 153944.3 |
| 369 | 83 | 55 | Cyclohexanecarboxylic acid, ethyl ester | 1136 | 3214.759 | 5562.539 | 6150.613 | 5485.409 | 5368.713 | 5971.832 | 11302.81 | 9463.14 | 9320.3 | 5277.379 | 6012.571 | 6269.557 |
| 370 | 71 | 111 | Terpinen-4-ol | 1177 | 525850.7 | 566140.2 | 557559.4 | 654809.1 | 685816 | 602492.2 | 596248.3 | 578559.1 | 554199.9 | 573952.3 | 618412.5 | 569225.2 |
| 371 | 69 | 41 | 2,7-Nonadien-5-one, 4,6-dimethyl- | 1138 | 8856.991 | 11332.21 | 9174.597 | 8367.877 | 8979.089 | 7349.163 | 96520.85 | 88929.72 | 89513.72 | 34773.11 | 46437 | 42592.82 |
| 372 | 108 | 67 | Limonene oxide, trans- | 1138 | 51515.33 | 61618.81 | 53652.46 | 27392.29 | 26714.55 | 23358.51 | 39471.4 | 34443.91 | 30900.94 | 34938.91 | 38210.99 | 41670.64 |
| 373 | 68 | 96 | 2,6,6-Trimethyl-2-cyclohexene-1,4-dione | 1145 | 40190.71 | 39478.26 | 39983.38 | 48072.5 | 50506.31 | 41588.06 | 27094.77 | 28139.45 | 26916.63 | 38374.57 | 38796.46 | 36214.87 |
| 374 | 121 | 79 | 2,4,6-Octatriene, 2,6-dimethyl-, (E,E)- | 1144 | 82163.71 | 87920.14 | 89116.3 | 155415.3 | 143771.9 | 106317.8 | 69719.87 | 79784.93 | 77810.98 | 74337.8 | 90626.5 | 91324.87 |
| 375 | 108 | 91 | Acetic acid, phenylmethyl ester | 1162 | 176603.9 | 203887.4 | 187500.7 | 184017.6 | 191064.8 | 159496.5 | 371051.6 | 341504.1 | 338994.4 | 214141 | 234530 | 223284.4 |
| 376 | 71 | 67 | Butanoic acid, 5-hexenyl ester | 1183 | 68673.66 | 70801.09 | 68902.59 | 81954.51 | 92430.64 | 65546.29 | 133658 | 132636.8 | 112301 | 81364.39 | 89657.39 | 91217.51 |
| 377 | 70 | 84 | Bicyclo[3.1.1]heptan-3-ol, 2,6,6-trimethyl-, (1.alpha.,2.beta.,3.alpha.,5.alpha.)- | 1179 | 74725.34 | 81748.07 | 76605.71 | 69595.7 | 84166.84 | 55487.28 | 102077.3 | 104661.1 | 87594.64 | 60762.99 | 68033.28 | 68741.76 |
| 378 | 81 | 67 | Cyclohexanone, 5-methyl-2-(1-methylethenyl)- | 1179 | 826142 | 832243.5 | 798882.6 | 935611.3 | 980269.4 | 864945.7 | 877783.4 | 823740.3 | 781427.5 | 832359.3 | 885529.5 | 805174 |
| 379 | 45 | 69 | 2-Nonanol | 1102 | 56536.64 | 52449.62 | 52970.38 | 75204.56 | 78773.82 | 61579.77 | 57136.55 | 59337.5 | 55487.35 | 55226.82 | 60645.34 | 58326.7 |
| 380 | 82 | 71 | 3,7-Octadiene-2,6-diol, 2,6-dimethyl- | 1193 | 26332.3 | 20219.33 | 27924 | 40507 | 37807.76 | 39851 | 23392.68 | 21949 | 21213 | 28566.76 | 32684 | 23835 |
| 381 | 110 | 54 | 3-Octen-1-ol, acetate, (Z)- | 1195 | 5325 | 3652 | 3866.857 | 7571 | 9516 | 7101 | 4934 | 2870 | 4158 | 4098 | 5717 | 4002 |
| 382 | 105 | 133 | Benzene, 1-methyl-2-(1-ethylpropyl)- | 1240 | 24404.93 | 25109.75 | 22601.94 | 39945.84 | 41308.54 | 31531.07 | 24311.68 | 24803.81 | 23162.39 | 27557.35 | 29232.91 | 27042.58 |
| 383 | 104 | 105 | Benzene, (2-nitroethyl)- | 1304 | 13464.83 | 14905.78 | 14755.85 | 22944.03 | 22689.75 | 20128.68 | 12973.66 | 11253.07 | 10910.4 | 14443.02 | 14372.06 | 12396.2 |
| 384 | 57 | 71 | Undecane, 4,6-dimethyl- | 1196 | 341731.7 | 367264.1 | 355073.1 | 317459.8 | 372638.7 | 264032.7 | 790566.8 | 831435.3 | 709724.3 | 399235 | 413273.1 | 439383.8 |
| 385 | 83 | 55 | Cyclohexanol, 2-methyl-3-(1-methylethenyl)-, (1.alpha.,2.alpha.,3.alpha.)- | 1196 | 21720.51 | 21568.22 | 22236.99 | 21420.91 | 25406.44 | 16914.29 | 42169.66 | 41861.23 | 35308.3 | 22861.86 | 23516.5 | 27319.81 |
| 386 | 136 | 135 | BenzAldehyde, 3-methoxy- | 1196 | 100436.9 | 107380.7 | 106134.4 | 126405.9 | 132391.5 | 116312.2 | 80557.37 | 79864.6 | 70546.75 | 90090.21 | 100641.5 | 98529.18 |
| 387 | 67 | 82 | Butanoic acid, 4-hexenyl ester, (Z)- | 1203 | 30497.56 | 33326.11 | 32882.04 | 70217.67 | 79948.07 | 69599.24 | 33131.99 | 31977.01 | 24372.06 | 31121.23 | 34649.99 | 41792.25 |
| 388 | 84 | 83 | (-)-cis-Isopiperitenol | 1228 | 171483.2 | 171324.3 | 162765.8 | 209141.1 | 224736.6 | 185100.2 | 137218.5 | 127523.1 | 111799.3 | 159542.6 | 167970.1 | 150373.6 |
| 389 | 81 | 67 | Cyclohexanone, 5-methyl-2-(1-methylethylidene)- | 1212 | 3477.24 | 2933.373 | 2737.251 | 3550.761 | 2158.2 | 3677.78 | 68610.92 | 63957.09 | 62862.43 | 22261.5 | 18019.63 | 19319.98 |
| 390 | 70 | 71 | Isopentyl hexanoate | 1250 | 2742838 | 2767455 | 2616585 | 3902327 | 4138732 | 3581927 | 2944235 | 2744638 | 2555752 | 3039918 | 3093771 | 2966871 |
| 391 | 125 | 168 | Thiophene, 2-butyl-5-ethyl- | 1282 | 12316.54 | 14108.7 | 14038 | 33093.89 | 35193.01 | 24145.2 | 9581.891 | 8643.593 | 8022.324 | 13177.49 | 14712.93 | 15323.21 |
| 392 | 41 | 93 | (+)-neodihydrocarveol | 1226 | 332392.8 | 326715.5 | 318663.5 | 375210.2 | 389983.8 | 352379.2 | 234145.5 | 221562.7 | 197910.9 | 292684.5 | 304906.3 | 280081.3 |
| 393 | 99 | 71 | 2(3H)-Furanone, 5-(acetyloxy)dihydro-5-methyl- | 1226 | 3550.99 | 3678.959 | 3137.038 | 8065.598 | 8024.996 | 6734.601 | 3049.137 | 3105.559 | 2472.73 | 4054.151 | 3765.833 | 4757.169 |
| 394 | 81 | 53 | Furan, 2-[(methyldithio)methyl]- | 1226 | 52637.92 | 56551.79 | 54206.38 | 67060.61 | 69116.63 | 56643.17 | 41644.25 | 39316.1 | 34163.75 | 53339.66 | 51990.44 | 48961.39 |
| 395 | 85 | 57 | Undecane, 4,4-dimethyl- | 1229 | 153951.5 | 151987 | 151681.6 | 241011.8 | 261164.8 | 189256.3 | 170568.7 | 162765.7 | 144218.8 | 152958.4 | 166243.5 | 165562.6 |
| 396 | 94 | 79 | 3-Cyclohexene-1-acetAldehyde, .alpha.,4-dimethyl- | 1225 | 51215.96 | 52117.61 | 49541.27 | 63513.54 | 64315.8 | 54356.16 | 43607.6 | 36984.83 | 33856.46 | 50441.53 | 52083.34 | 47977.37 |
| 397 | 136 | 135 | Pyrazine, 2,3-dimethyl-5-(1-methylpropyl)- | 1229 | 35956.97 | 37902.47 | 35310.13 | 52903.78 | 49360.56 | 42335.2 | 30206.11 | 26203.28 | 24449.6 | 37034.2 | 34572.91 | 35369.06 |
| 398 | 99 | 71 | n-Caproic acid vinyl ester | 974 | 75514.44 | 72261.92 | 68615.88 | 40818.02 | 46383.23 | 40235.66 | 60889.31 | 61396.22 | 61332.9 | 57517.02 | 61204.08 | 50465.53 |
| 399 | 82 | 110 | 2-Cyclohexen-1-one, 3-methyl-6-(1-methylethyl)- | 1253 | 497876.9 | 508640.7 | 478317.2 | 735146.2 | 763856.5 | 660226.2 | 540332.7 | 505640.1 | 467493.7 | 558445.7 | 570468.8 | 550269.9 |
| 400 | 105 | 77 | Benzoic acid, 2-propenyl ester | 1254 | 504399.3 | 519524.3 | 491637 | 778899.5 | 795448.8 | 668869.2 | 576331.5 | 533403.9 | 491616 | 573386.3 | 580659.7 | 577744.7 |
| 401 | 110 | 95 | Carvenone | 1257 | 705737.6 | 750887.3 | 688601.1 | 1149355 | 1177150 | 957310.7 | 802694.6 | 708201.7 | 653988.9 | 789707 | 799534.1 | 799595.6 |
| 402 | 79 | 52 | Picolinamide | 1269 | 59101.38 | 59278.58 | 58058.04 | 119165.7 | 118022.6 | 90683.78 | 55031.67 | 50254.5 | 50266.7 | 67766.34 | 70074.68 | 66558.07 |
| 403 | 57 | 71 | Dodecane, 3-methyl- | 1271 | 11067.92 | 12248.22 | 11100.8 | 19186.39 | 20722.22 | 12736.27 | 9421.681 | 8453.446 | 8716.375 | 10616.68 | 10890.07 | 12188.1 |
| 404 | 68 | 79 | 1-Cyclohexene-1-carboxAldehyde, 4-(1-methylethenyl)- | 1274 | 42245.46 | 39446.8 | 37354.83 | 72237.59 | 75176.54 | 60144.69 | 40707.51 | 37835.81 | 34825.5 | 47570.85 | 47239.18 | 44990.03 |
| 405 | 136 | 121 | p-Menth-8-en-3-ol, acetate | 1259 | 1226066 | 1312405 | 1196234 | 2023231 | 2084437 | 1684126 | 1416275 | 1233185 | 1136706 | 1395214 | 1395890 | 1389135 |
| 406 | 83 | 55 | 6-Undecanol | 1277 | 12707.61 | 14541.81 | 13104.67 | 25076.04 | 27686.3 | 21110.91 | 11564.76 | 11353.73 | 10360.45 | 14575.68 | 15424.87 | 14285.33 |
| 407 | 57 | 71 | Dodecane, 4,6-dimethyl- | 1325 | 38936.34 | 41782.75 | 41788.68 | 65943.91 | 78489.52 | 51990.56 | 35065.28 | 36533.87 | 28927.55 | 37865.28 | 38455.95 | 41414.77 |
| 408 | 70 | 99 | Hexanoic acid, pentyl ester | 1287 | 64247.7 | 65739.66 | 63779.87 | 75625.26 | 79980.16 | 61894.38 | 54549.73 | 52573.1 | 47950.99 | 55676.45 | 59755.83 | 55782.71 |
| 409 | 95 | 69 | dl-Camphoroquinone | 1319 | 29708 | 34339 | 33165 | 38507 | 40186 | 31224 | 22131 | 25417.92 | 25143 | 29205 | 28534 | 29275 |
| 410 | 105 | 123 | Benzoic acid, 2-methylpropyl ester | 1321 | 20406.59 | 22051.69 | 20827.42 | 20373.64 | 26943.61 | 19959.19 | 16492.89 | 17125.21 | 13693.44 | 18440.29 | 17868.28 | 19916.66 |
| 411 | 135 | 150 | Phenol, 2-methyl-5-(1-methylethyl)- | 1299 | 22118.12 | 21867.74 | 21912.42 | 28475.27 | 31111.59 | 26654.72 | 26789.53 | 20788.19 | 21711.3 | 23234.57 | 24735.54 | 22193.97 |
| 412 | 41 | 69 | cis-.beta.-Farnesene | 1444 | 9 | 9 | 9 | 9 | 9 | 9 | 475057.3 | 479143.4 | 437212.7 | 162459.8 | 168877.8 | 170824.1 |
| 413 | 91 | 108 | Propanoic acid, 2-methyl-, phenylmethyl ester | 1299 | 18532.36 | 18895.69 | 16874.49 | 36955.84 | 42059.36 | 33879.21 | 38505.3 | 36673.98 | 34132.93 | 27795.32 | 27075.71 | 30437.18 |
| 414 | 122 | 135 | Pyrazine, 2,5-dimethyl-3-(3-methylbutyl)- | 1317 | 41963.16 | 44293.48 | 43647.51 | 15517.21 | 17702.16 | 13425.06 | 27069.16 | 26650.71 | 22829.09 | 28489.09 | 29129.78 | 27005.66 |
| 415 | 57 | 71 | Dodecane, 2,6,10-trimethyl- | 1366 | 166302.4 | 175329.8 | 172316 | 177965.7 | 208469 | 142168.2 | 253426.7 | 262357.3 | 237228 | 155327.8 | 159772.8 | 169262.3 |
| 416 | 107 | 122 | 5,8-Decadien-2-one, 5,9-dimethyl-, (E)- | 1320 | 81161.99 | 90900.97 | 94044.47 | 94168.96 | 100837 | 76878.42 | 62475.5 | 63637.7 | 59455.99 | 70566.51 | 73500.27 | 71580.3 |
| 417 | 128 | 56 | 1-Methyl-2,4,5-trioxoimidazolidine | 1324 | 4899.97 | 6158.446 | 5980.288 | 5635.642 | 6732.974 | 5015.615 | 3931.303 | 4067.624 | 3923.706 | 4515.972 | 4703.013 | 4561.862 |
| 418 | 99 | 41 | Hexanoic acid, cyclopentyl ester | 1325 | 51885.29 | 55374.59 | 55876.02 | 58618.3 | 66456.73 | 47981.03 | 40515.64 | 39867.62 | 36302.54 | 45149.42 | 47435.27 | 46460.77 |
| 419 | 103 | 75 | Benzene, (2,2-diethoxyethyl)- | 1328 | 11906.87 | 6154.636 | 9753.645 | 5383.809 | 3968.062 | 3925.272 | 4573.307 | 5769.744 | 4876.678 | 8817.137 | 11077.35 | 7554.831 |
| 420 | 108 | 91 | Butanoic acid, phenylmethyl ester | 1347 | 23833.15 | 21511.82 | 26309.16 | 35301.05 | 37398.76 | 37388.43 | 81917.41 | 82810.43 | 78793.92 | 44012.39 | 43155.75 | 43000.94 |
| 421 | 136 | 121 | Dihydrocarvyl acetate | 1306 | 24812.33 | 26340.17 | 22240.09 | 56360.19 | 62005.92 | 49490.09 | 30951.38 | 25638.87 | 24995.91 | 31114.12 | 32840.35 | 29449.85 |
| 422 | 108 | 41 | 1,4-Methano-1H-indene, octahydro-4-methyl-8-methylene-7-(1-methylethyl)-, [1S-(1.alpha.,3a.beta.,4.alpha.,7.alpha.,7a.beta.)]- | 1396 | 17282.9 | 18044.1 | 18384.75 | 17955.56 | 19793.7 | 16190.93 | 89155.06 | 87496.28 | 78724.02 | 35953.72 | 39778.83 | 40095.56 |
| 423 | 71 | 67 | 2,7-Octadiene-1,6-diol, 2,6-dimethyl-, (Z)- | 1361 | 24353.4 | 26924.57 | 25401.35 | 28423.57 | 33833.66 | 25220.14 | 38736.95 | 42545.18 | 37241.52 | 23819.17 | 26321.94 | 28099.02 |
| 424 | 161 | 160 | 2-(3-Thienyl)pyridine | 1362 | 1729.361 | 1742.673 | 2268.17 | 1056.232 | 1028.107 | 1160.614 | 7976.075 | 9210.237 | 8358.581 | 3247.234 | 3834.113 | 4079.531 |
| 425 | 41 | 70 | 2-Undecenal, E- | 1366 | 33716.7 | 34801.66 | 32343.15 | 38183.33 | 46186.7 | 28927.14 | 52176.81 | 50754.09 | 47425.97 | 32068.97 | 33619.64 | 34650.76 |
| 426 | 55 | 69 | 1-Undecanol | 1371 | 26801.69 | 28058.56 | 28791.28 | 63289.81 | 70114.03 | 50658.31 | 22402.86 | 24323.96 | 20269.93 | 27873.22 | 29347.1 | 30275.41 |
| 427 | 41 | 55 | 2-Octenal, 2-butyl- | 1378 | 67056 | 79689 | 78606 | 65602 | 75877 | 52882 | 32331.58 | 33212.27 | 28121.31 | 48153 | 50084 | 61795 |
| 428 | 117 | 99 | Hexanoic acid, hexyl ester | 1384 | 209794.3 | 238536.4 | 212905.7 | 345129 | 379404.1 | 275824.3 | 226533.7 | 232889.6 | 220687 | 218738.1 | 226336.3 | 227465.5 |
| 429 | 178 | 163 | Benzene, 1,2-dimethoxy-4-(1-propenyl)- | 1492 | 319410 | 334253.8 | 341164.1 | 347127.2 | 408644.3 | 325668.3 | 9114647 | 8780691 | 8958952 | 3049734 | 2923398 | 3071983 |
| 430 | 41 | 67 | 1H-Cycloprop[e]azulene, decahydro-1,1,4,7-tetramethyl-, [1aR-(1a.alpha.,4.beta.,4a.beta.,7.beta.,7a.beta.,7b.alpha.)]- | 1373 | 15663.9 | 17286.97 | 15917.92 | 36432.62 | 42851.97 | 29401.07 | 13281.61 | 14928.5 | 13074 | 16139.26 | 17668.06 | 17183.46 |
| 431 | 71 | 56 | Propanoic acid, 2-methyl-, 3-hydroxy-2,2,4-trimethylpentyl ester | 1374 | 22431.06 | 23286.97 | 24026.03 | 45278.12 | 50945.3 | 34313.42 | 20812.22 | 21763.67 | 20217.47 | 22672.78 | 23166.14 | 24334.94 |
| 432 | 82 | 67 | Hexanoic acid, 3-hexenyl ester, (Z)- | 1380 | 744172.7 | 781045.5 | 756045.8 | 719064.1 | 813533.9 | 585744.4 | 481579.2 | 498411.7 | 441830.8 | 587512.2 | 606676.4 | 620824.9 |
| 433 | 111 | 157 | Diethyl adipate | 1385 | 6532.219 | 6911.44 | 6618.079 | 8746.645 | 7512.681 | 6713.273 | 11355.77 | 11988.25 | 11104.13 | 6512.777 | 7307.356 | 6737.755 |
| 434 | 159 | 117 | 1,1,7,7a-Tetramethyl-1a,2,6,7,7a,7b-hexahydro-1H-cyclopropa[a]naphthalene | 1440 | 837.0401 | 877.0287 | 1037.71 | 644.0582 | 759.6807 | 462.8422 | 42417.61 | 41220.91 | 38120.71 | 13751.64 | 14166.04 | 15970.6 |
| 435 | 119 | 93 | 1H-3a,7-Methanoazulene, 2,3,4,7,8,8a-hexahydro-3,6,8,8-tetramethyl-, (3R,3aS,7S,8aR)- | 1389 | 11676.61 | 13325.4 | 11988.35 | 12104.82 | 12699.9 | 9184.337 | 7951.644 | 7548.938 | 6043.426 | 9427.365 | 10163.47 | 9384.119 |
| 436 | 119 | 93 | (1S,5S)-2-Methyl-5-((R)-6-methylhept-5-en-2-yl)bicyclo[3.1.0]hex-2-ene | 1394 | 5280.271 | 6420.336 | 6317.223 | 6706.24 | 8099.19 | 6630.98 | 8649.391 | 10596.16 | 10457.67 | 7396.231 | 6777.507 | 6680.857 |
| 437 | 58 | 104 | Nitroguanidine | 1392 | 7783.225 | 8078.002 | 8129.706 | 9617.581 | 11158.9 | 8026.282 | 27004.28 | 27446.16 | 23477.3 | 12559.73 | 12572.77 | 13910.38 |
| 438 | 83 | 55 | 2(5H)-Furanone, 5-ethyl- | 966 | 11230.27 | 11574.65 | 10355.04 | 11263.2 | 13813.81 | 11150.19 | 5877.366 | 5468.03 | 4742.565 | 8763.229 | 8578.441 | 7556.543 |
| 439 | 189 | 161 | 1H-Cyclopropa[a]naphthalene, 1a,2,3,3a,4,5,6,7b-octahydro-1,1,3a,7-tetramethyl-, [1aR-(1a.alpha.,3a.alpha.,7b.alpha.)]- | 1398 | 4162.081 | 5268.477 | 4357.544 | 4117.367 | 5203.631 | 3718.822 | 7557.173 | 7355.41 | 7128.955 | 4706.152 | 3985.356 | 4155.771 |
| 440 | 108 | 53 | 3-Amino-4-pyrazolecarbonitrile | 1399 | 8788.174 | 10563.22 | 9510.306 | 20689.55 | 22237.47 | 19316.78 | 19255.21 | 18685.08 | 17495.8 | 14027.96 | 13761.31 | 15063.83 |
| 441 | 135 | 107 | Phenol, 4-(1,1-dimethylpropyl)- | 1400 | 65961.2 | 76422.25 | 76708.61 | 173269.1 | 174904 | 166047 | 121536.3 | 124439.4 | 114333.5 | 113255 | 111127.1 | 111521.7 |
| 442 | 119 | 92 | 1-ButanAmine, N-(2-pyridinylmethylene)- | 1401 | 6015.192 | 6653.308 | 6790.811 | 13652.91 | 14653.88 | 13110.32 | 27066.82 | 10542.3 | 9326.455 | 9455.836 | 9333.751 | 9236.034 |
| 443 | 93 | 133 | Bicyclo[5.2.0]nonane, 2-methylene-4,8,8-trimethyl-4-vinyl- | 1407 | 57324.83 | 60661.35 | 59753.87 | 131259.1 | 139913 | 125629 | 131363.9 | 100105.6 | 88503.87 | 90166.23 | 90819.76 | 85895.67 |
| 444 | 112 | 41 | 4a(2H)-Naphthalenol, octahydro-4,8a-dimethyl-,(4.alpha.,4a.alpha.,8a.beta.)- | 1430 | 3836.223 | 4386.33 | 3343.661 | 4676.483 | 5353.079 | 3700.974 | 104470.5 | 106967.5 | 94853.15 | 37046.79 | 38267.97 | 38493.06 |
| 445 | 69 | 70 | 2,6-Dodecadien-1-al | 1449 | 9840.044 | 7654.45 | 6630.243 | 5814.112 | 8401.231 | 5662.899 | 34930.61 | 33507.48 | 30475.31 | 12354.91 | 12308.6 | 11509.27 |
| 446 | 121 | 93 | Ionone | 1422 | 2941.96 | 4331.919 | 5104.001 | 15785.19 | 14461.65 | 10986.25 | 49320.5 | 47500.89 | 42078.35 | 15922.98 | 17932.19 | 16422.56 |
| 447 | 81 | 41 | (E,E)-2,4-Undecadienal | 1430 | 5063.332 | 4291.828 | 3013.409 | 6321.167 | 7888.106 | 6240.007 | 205533 | 212554.7 | 186213.1 | 72096.82 | 76323.18 | 73969.99 |
| 448 | 93 | 41 | Bicyclo[3.1.1]hept-2-ene, 2,6-dimethyl-6-(4-methyl-3-pentenyl)- | 1435 | 69513.84 | 72614.21 | 70598.37 | 77140.62 | 81891.22 | 68435.26 | 3353604 | 3441073 | 3107455 | 1167669 | 1219526 | 1199681 |
| 449 | 161 | 41 | 1H-Cyclopropa[a]naphthalene, 1a,2,3,5,6,7,7a,7b-octahydro-1,1,7,7a-tetramethyl-, [1aR-(1a.alpha.,7.alpha.,7a.alpha.,7b.alpha.)]- | 1432 | 3641.701 | 4551.137 | 4050.908 | 27432.52 | 30684.21 | 22296.86 | 19079.93 | 20172.55 | 17748.47 | 12163.33 | 13095.03 | 15252.88 |
| 450 | 41 | 93 | Bicyclo[7.2.0]undec-4-ene, 4,11,11-trimethyl-8-methylene-,[1R-(1R*,4Z,9S*)]- | 1406 | 19216.25 | 19370.86 | 19037.91 | 45377.05 | 49118.85 | 41249.66 | 32955.54 | 32025.8 | 29174.26 | 28342.74 | 28531.1 | 29341.62 |
| 451 | 146 | 118 | Coumarin | 1440 | 737.0757 | 706.8944 | 1168.089 | 9 | 9 | 9 | 18812.62 | 19652.14 | 17585.88 | 6963.205 | 6802.213 | 7024.603 |
| 452 | 105 | 161 | [1α,4aα,8aα]-1,2,4a,5,6,8a-hexahydro-4-7-dimethyl-1-[1-methylethyl]naphthalene | 1496 | 62237.43 | 61423.31 | 59326.6 | 15814.06 | 24812.55 | 18183.05 | 336795.4 | 337156.9 | 357763.6 | 115101.3 | 122710.5 | 118530.2 |
| 453 | 137 | 180 | 2,5-Dihydroxy-4-isopropyl-2,4,6-cycloheptatrien-1-one | 1441 | 1004.833 | 1245.623 | 945.1495 | 1188.254 | 1268.093 | 1145.197 | 382593.2 | 381303.8 | 350637.8 | 124108.8 | 136155.5 | 131629.5 |
| 454 | 138 | 56 | CyclohexanAmine, N-cyclohexyl- | 1443 | 9 | 9 | 9 | 9 | 9 | 9 | 104678.7 | 105185.1 | 97905.89 | 34774.12 | 37910.8 | 36018.54 |
| 455 | 99 | 41 | 2(3H)-Furanone, 5-hexyldihydro-4-methyl-, trans- | 1444 | 9 | 9 | 9 | 9 | 9 | 9 | 31510.62 | 32057.18 | 28724.61 | 10870.24 | 10490.62 | 10746.73 |
| 456 | 115 | 134 | Acetic Acid, cinnamyl ester | 1446 | 1424.948 | 1368.204 | 873.1155 | 1632.069 | 1128.452 | 1242.307 | 56717.03 | 55941.51 | 53507 | 21452.03 | 21474.74 | 20846.35 |
| 457 | 91 | 83 | Benzyl angelate | 1446 | 4132.649 | 5234.372 | 4632.644 | 7379.497 | 8009.619 | 6234.625 | 1107205 | 1119533 | 1021547 | 375475.4 | 392687.8 | 381597.8 |
| 458 | 190 | 148 | Megastigmatrienone | 1473 | 15946.43 | 16163.28 | 17060.53 | 2927.924 | 2549.237 | 2335.904 | 9006.669 | 7736.264 | 7057.297 | 6801.063 | 9024.398 | 8439.473 |
| 459 | 189 | 133 | (4aR-trans)-decahydro-4a-methyl-1-methylene-7-(1-methylethylidene)-Naphthalene | 1479 | 10955.55 | 11585.31 | 12097.63 | 12530.74 | 15059.88 | 10623.7 | 722602.6 | 733305.8 | 784628.8 | 197109 | 204044 | 222964 |
| 460 | 97 | 96 | Cyclohexane, 1-(1,5-dimethylhexyl)-4-methyl- | 1461 | 20254.85 | 22085.99 | 22595.22 | 12430.06 | 14326.22 | 13135.59 | 20534.53 | 20544.54 | 19889.5 | 22086.37 | 24429.12 | 21626.9 |
| 461 | 95 | 109 | Iridomyrmecin | 1463 | 57883.52 | 58013.39 | 55414.94 | 5326.762 | 7660.124 | 5473.345 | 70397.76 | 65243.11 | 59089.31 | 39949.22 | 42895.97 | 41315.03 |
| 462 | 129 | 100 | Diisopropyl adipate | 1464 | 26929.69 | 29313.26 | 28737.54 | 10754.67 | 13703.66 | 10904.7 | 42141.37 | 39849.77 | 38886.84 | 27572.9 | 28037.11 | 27310.22 |
| 463 | 131 | 160 | 2H-1-Benzopyran-2-one, 3-methyl- | 1490 | 72134.28 | 72292.66 | 74647.16 | 76502.18 | 87221.4 | 71060.69 | 1034203 | 1023087 | 1000384 | 416232.2 | 402643.1 | 421346.7 |
| 464 | 152 | 108 | trans-O-Dithiane-4,5-diol | 1466 | 1482.058 | 1622.661 | 1922.571 | 9 | 9 | 9 | 17941.52 | 16613.76 | 16606.21 | 6038.105 | 7121.482 | 6418.643 |
| 465 | 175 | 190 | Precocene I | 1466 | 23677.6 | 25006.46 | 24990.25 | 2449.134 | 2919.008 | 1978.099 | 10993.56 | 8373.969 | 7363.883 | 10927.47 | 11011.58 | 12800.31 |
| 466 | 119 | 121 | Spiro[4.5]dec-7-ene, 1,8-dimethyl-4-(1-methylethenyl)-, [1S-(1.alpha.,4.beta.,5.alpha.)]- | 1471 | 124851.3 | 130728.1 | 134591.6 | 4339.324 | 5899.447 | 5150.876 | 51128.15 | 40358.89 | 31684.36 | 49120.59 | 55905.14 | 57594.44 |
| 467 | 107 | 161 | Eremophilene | 1494 | 41382.45 | 43498.28 | 41236.03 | 6193.156 | 7671.916 | 5141.645 | 286775.2 | 290289.6 | 302043.7 | 93303.8 | 102684.9 | 97036.74 |
| 468 | 131 | 119 | Eudesma-2,4,11-triene | 1479 | 3154.671 | 3953.287 | 2880.75 | 3617.496 | 3720.886 | 3023.397 | 40525.35 | 40294.62 | 40824.36 | 13744.84 | 13402.79 | 14591.96 |
| 469 | 85 | 69 | 1-Pentanol, 5-[(tetrahydro-2H-pyran-2-yl)oxy]- | 1486 | 30544.76 | 31831.32 | 29827.21 | 35306.51 | 42117.32 | 31050.54 | 128160 | 132318.5 | 119740.7 | 65150.1 | 61972.38 | 64912.05 |
| 470 | 69 | 93 | Propanoic acid, 2-methyl-, 3,7-dimethyl-2,6-octadienyl ester, (Z)- | 1475 | 71692.38 | 81632.42 | 78719.06 | 9 | 9 | 9 | 119724.2 | 115736.2 | 101557.5 | 63680.18 | 60801.86 | 56808.63 |
| 471 | 115 | 71 | 2-Phenoxyethyl isobutyrate | 1488 | 80585.41 | 79195.31 | 82723.73 | 82146.48 | 95768.34 | 74435.08 | 820409.8 | 840988.4 | 804935.9 | 346750.1 | 338476.3 | 349960.1 |
| 472 | 158 | 157 | Nicotyrine | 1488 | 3690.025 | 4430.815 | 4184.706 | 4037.142 | 4870.827 | 3580.959 | 65222.52 | 62373.73 | 61429.49 | 21106.26 | 20250.03 | 23462.83 |
| 473 | 120 | 41 | Bicyclo[7.2.0]undecane, 10,10-dimethyl-2,6-bis(methylene)-, [1S-(1R*,9S*)]- | 1489 | 51608 | 53046.48 | 55486.02 | 49864.18 | 59177.44 | 45305.41 | 378963.7 | 382507.7 | 372228.4 | 167816.6 | 162416.4 | 173239.3 |
| 474 | 120 | 69 | 10,10-Dimethyl-2,6-dimethylenebicyclo[7.2.0]undecane | 1440 | 1433.54 | 2020.009 | 1205.9 | 2214.885 | 2442.001 | 2551.22 | 490222.3 | 492173.7 | 455794.1 | 158499.7 | 176694.7 | 170413.7 |
| 475 | 107 | 93 | [1S-(1.alpha.,7.alpha.,8a.beta.)]-1,2,3,5,6,7,8,8a-octahydro-1,4-dimethyl-7-(1-methylethenyl)-Azulene | 1505 | 9754.685 | 12990.32 | 12084.45 | 21980.94 | 28547.98 | 20072.72 | 127457 | 129298.9 | 131898.5 | 41423.99 | 45337.9 | 44894.87 |
| 476 | 107 | 150 | 3-(4-Hydroxyphenyl)propanal | 1490 | 242791.6 | 246465.7 | 251436.4 | 259749.4 | 304621.5 | 237392.7 | 2717323 | 2742729 | 2614128 | 1123681 | 1108478 | 1148608 |
| 477 | 69 | 93 | (1S,5S,6R)-6-Methyl-2-methylene-6-(4-methylpent-3-en-1-yl)bicyclo[3.1.1]heptane | 1492 | 104854.1 | 106264.1 | 109040.1 | 115602 | 132642.8 | 102141 | 918712.5 | 936197.3 | 865619.6 | 408819.1 | 406840.6 | 416096.7 |
| 478 | 134 | 91 | (5R,10R)-10-Methyl-6-methylene-2-(propan-2-ylidene)spiro[4.5]dec-7-ene | 1495 | 9469.978 | 9623.136 | 10054.44 | 659.7945 | 604.7455 | 826.1981 | 63665.13 | 62946.14 | 70071.91 | 20721.14 | 21761.27 | 20530.47 |
| 479 | 192 | 64 | Hexathiane | 1500 | 172183.2 | 173444.4 | 175253.7 | 1217.701 | 1274.527 | 1344 | 1036544 | 1136883 | 1188999 | 452519 | 470731.6 | 345405.3 |
| 480 | 189 | 133 | 4a,8-Dimethyl-2-(prop-1-en-2-yl)-1,2,3,4,4a,5,6,7-octahydronaphthalene | 1492 | 58610.25 | 62060.26 | 59241.73 | 14057.97 | 17541.95 | 14440.46 | 322569.4 | 300067.7 | 346694.2 | 105117 | 109919.1 | 104871.7 |
| 481 | 105 | 91 | Benzene, (1-methyl-1-propylpentyl)- | 1504 | 90469.83 | 95506.04 | 89612.32 | 184614 | 223696 | 165331.2 | 447097.8 | 466399.5 | 432584 | 206188.1 | 208181.7 | 223081.1 |
| 482 | 69 | 68 | Geranyl isobutyrate | 1514 | 10848.85 | 10081.92 | 10224.57 | 3397.101 | 4933.573 | 4313.884 | 113682.4 | 113845.6 | 114269 | 33853.76 | 32385.94 | 34149.85 |
| 483 | 137 | 109 | 2,4,4-Trimethyl-3-(3-methylbutyl)cyclohex-2-enone | 1520 | 56791.97 | 63478.71 | 57079.1 | 62106.66 | 67105.97 | 61774.3 | 75583.78 | 81320.89 | 69327.93 | 66593.77 | 57276.8 | 57873.16 |
| 484 | 81 | 95 | Citronellyl butyrate | 1529 | 66342.67 | 71504.16 | 66387.84 | 80077.27 | 91399.79 | 68318.41 | 116546.1 | 123784.5 | 112236.8 | 83296.94 | 82958.27 | 85101.31 |
| 485 | 93 | 107 | (E)-1-Methyl-4-(6-methylhept-5-en-2-ylidene)cyclohex-1-ene | 1533 | 33321.41 | 35768.99 | 32939.7 | 37466.53 | 42472.41 | 32397.16 | 67614.94 | 73122.33 | 68223.92 | 43686.59 | 43399.13 | 45619.96 |
| 486 | 41 | 82 | (1S,4R,7S,8R,11R,13R)-4,7,11-Trimethyl-5-oxatetracyclo[5.4.2.01,8.04,13]tridecane | 1546 | 1527.2 | 1536.317 | 1572.502 | 2202.73 | 1403.251 | 1655.176 | 369206 | 387484.5 | 389312.7 | 106126 | 121398.3 | 107406.4 |
| 487 | 55 | 110 | Patulin | 1545 | 1710.346 | 1633.616 | 1606.242 | 1749.16 | 1517.974 | 1351.846 | 352417.9 | 369080.8 | 375118.1 | 102865 | 113637.3 | 100235.8 |
| 488 | 56 | 57 | n-Capric acid isobutyl ester | 1546 | 664.8552 | 706.8589 | 1074.512 | 595.066 | 1300.767 | 640.3471 | 28500.39 | 29463.67 | 30077.27 | 9194.602 | 9571.773 | 8728.997 |
| 489 | 105 | 123 | Benzoic acid, hexyl ester | 1580 | 76816.72 | 79472.09 | 73827.18 | 87084.13 | 97199.27 | 78718.27 | 66256.55 | 72288.08 | 59517.92 | 81750.18 | 72336.01 | 72620.53 |
| 490 | 55 | 41 | cis-5-Dodecenoic Acid | 1561 | 18348.45 | 18160.84 | 18816 | 11202.03 | 16384.67 | 12508.72 | 19714.03 | 19949.15 | 17979.51 | 18152.63 | 17270.37 | 14646.34 |
| 491 | 82 | 67 | Octanoic acid, 3-hexenyl ester, (Z)- | 1588 | 5564.891 | 6545.796 | 5416.453 | 5226.618 | 5579.473 | 4362.799 | 7857.306 | 8494.811 | 7117.366 | 5679.91 | 6557.315 | 4649.677 |
| 492 | 69 | 41 | d-Nerolidol | 1544 | 1702.5 | 1718.578 | 1098.99 | 621.9867 | 1465.342 | 1162.033 | 170663.4 | 177694.6 | 179462.9 | 49346.02 | 55106.98 | 48246.55 |
| 493 | 82 | 105 | 3-Hexen-1-ol, benzoate, (Z)- | 1570 | 182900 | 183201.6 | 178598.2 | 135155.9 | 155643.7 | 131296.6 | 170297 | 181698.2 | 155616.3 | 166218.8 | 165902.8 | 156628.8 |
| 494 | 41 | 55 | Palustrol (Ledum) | 1568 | 9799.466 | 9316.356 | 8051.123 | 9943.784 | 9565.536 | 8655.829 | 13686.4 | 15170.95 | 13130.59 | 10423.11 | 9539.861 | 9274.686 |
| 495 | 81 | 123 | (2E,4S,7E)-4-Isopropyl-1,7-dimethylcyclodeca-2,7-dienol | 1574 | 12473.8 | 12547.67 | 13506.26 | 9792.391 | 11916.57 | 9267.371 | 18753.66 | 16760.75 | 14537.39 | 12615.35 | 13443.32 | 11602.84 |
| 496 | 187 | 159 | Cadina-1(10),6,8-triene | 1528 | 3807.28 | 4189.138 | 3396.496 | 4072.491 | 4003.79 | 3651.926 | 5886.314 | 6476.692 | 5163.705 | 4704.378 | 3245.683 | 4045.515 |
| 497 | 85 | 95 | 1,4-Methanoazulen-3-ol, decahydro-1,5,5,8a-tetramethyl-, [1S-(1.alpha.,3.beta.,3a.beta.,4.alpha.,8a.beta.)]- | 1593 | 7819.001 | 7203.938 | 7371.109 | 10113.15 | 12257.56 | 8534.925 | 19857.44 | 19832.35 | 18233.3 | 11133.6 | 11194.26 | 10969.39 |
| 498 | 185 | 200 | .alpha.-Corocalene | 1623 | 12911.96 | 13791.52 | 14070.26 | 13279.69 | 14553.31 | 11787.48 | 16135.51 | 17422.87 | 17202.86 | 14400.1 | 14326.65 | 14267.33 |
| 499 | 123 | 41 | 3-Buten-2-one, 4-(2,2,6-trimethyl-7-oxabicyclo[4.1.0]hept-1-yl)- | 1473 | 9526.828 | 11253.25 | 10205.15 | 9 | 9 | 9 | 30554.86 | 27876.95 | 25812.88 | 12535.3 | 11450.81 | 12460.43 |
| 500 | 177 | 220 | 2,5-Cyclohexadiene-1,4-dione, 2,6-bis(1,1-dimethylethyl)- | 1472 | 5366.531 | 5469.534 | 6212.64 | 4908.272 | 6648.243 | 5474.37 | 7994.553 | 7722.496 | 7050.03 | 5704.043 | 6030.104 | 5958.764 |
| 501 | 83 | 82 | Cyclopentaneacetic acid, 3-oxo-2-pentyl-, methyl ester | 1656 | 213145.8 | 220557.8 | 221406 | 23473.81 | 20670.2 | 17584.81 | 1765703 | 1863004 | 1609790 | 654316.8 | 668427.5 | 602074.5 |
| 502 | 137 | 182 | Homovanillic Acid | 1657 | 7122.614 | 7397.996 | 9278.437 | 2113.13 | 2993.985 | 2670.567 | 48788.29 | 52026.8 | 44191.96 | 19637.01 | 19280.05 | 18725.51 |
| 503 | 81 | 83 | Citronellyl tiglate | 1658 | 54551.51 | 56801.53 | 57436.17 | 21046.45 | 18841.87 | 14889.63 | 360308.1 | 379630.1 | 327506.4 | 144107.5 | 145966.7 | 132541 |
| 504 | 79 | 67 | 1,8,11,14-Heptadecatetraene, (Z,Z,Z)- | 1664 | 73544.21 | 76072.33 | 76665.7 | 19163.8 | 19965.99 | 16500 | 546693.1 | 574739.6 | 494585.1 | 209869.8 | 213826.6 | 192973 |
| 505 | 82 | 93 | 3-Cyclohexen-1-ol, 1-(1,5-dimethyl-4-hexenyl)-4-methyl- | 1671 | 64242.37 | 66589.25 | 66151.12 | 7672.764 | 7678.017 | 6830.37 | 508528.6 | 531380.4 | 457807 | 191921.2 | 197876.4 | 179442.4 |
| 506 | 79 | 67 | Cyclobuta[1,2:3,4]dicyclooctene, 1,2,5,6,6a,6b,7,8,11,12,12a,12b-dodecahydro-, (6a.alpha.,6b.beta.,12a.alpha.,12b.beta.)- | 1687 | 4494.482 | 3919.614 | 4328.704 | 1882.943 | 1669.574 | 1403.839 | 29214.54 | 33195.32 | 27211.53 | 11054.72 | 11757.4 | 9288.787 |
| 507 | 109 | 135 | 4,8-Methanoazulen-9-ol, decahydro-2,2,4,8-tetramethyl-, stereoisomer | 1566 | 18896.48 | 18593.41 | 18730.04 | 13309.12 | 17259.15 | 12851.45 | 20211.87 | 21039.97 | 19004.56 | 18556.75 | 17265.08 | 15513.49 |
| 508 | 84 | 133 | (S)-3-(1-methyl-2-pyrrolidinyl)-Pyridine | 1361 | 11886.95 | 11914.29 | 11500.16 | 20819.71 | 22687.27 | 18755.98 | 10538.81 | 11930.8 | 11685.91 | 12217.13 | 12916.91 | 12610.97 |
| 509 | 100 | 71 | 2-methyl-3-Pentanone | 745 | 16406.93 | 17564.89 | 17782.32 | 25117.2 | 24734.88 | 21031.29 | 4984.101 | 4817.64 | 4650.635 | 12078.26 | 13375.25 | 11365.57 |

a. Important volatile metabolite serial number detected by GC-MS; b. Quan, quantitative ion; c. Qual, qualitative ion; d, the retention index of the compound on the non-polar column; e. Abundance values of volatile metabolites in different black tea samples, wherein, CK indicates congou black tea, CH indicates chloranthus spicatus black tea, OF indicates osmanthus black tea; f. QC is a mixture of 9 sample extracts and is mainly used to analyze the repeatability of samples under the same treatment method. 1, 2, 3 indicates three replicates of samples.
